# A unified photoredox-catalysis strategy for C(sp^3^)–H hydroxylation and amidation using hypervalent iodine†
†Electronic supplementary information (ESI) available. See DOI: 10.1039/c7sc02773g



**DOI:** 10.1039/c7sc02773g

**Published:** 2017-09-04

**Authors:** Guo-Xing Li, Cristian A. Morales-Rivera, Fang Gao, Yaxin Wang, Gang He, Peng Liu, Gong Chen

**Affiliations:** a State Key Laboratory and Institute of Elemento-Organic Chemistry , College of Chemistry , Collaborative Innovation Center of Chemical Science and Engineering (Tianjin) , Nankai University , Tianjin 300071 , China . Email: gongchen@nankai.edu.cn; b Department of Chemistry , University of Pittsburgh , Pittsburgh , PA 15260 , USA . Email: pengliu@pitt.edu; c Department of Chemistry , The Pennsylvania State University , 104 Chemistry Building , University Park , PA 16802 , USA . Email: guc11@psu.edu

## Abstract

We report a unified photoredox-catalysis strategy for both hydroxylation and amidation of tertiary and benzylic C–H bonds. Use of hydroxyl perfluorobenziodoxole (PFBl–OH) oxidant is critical for efficient tertiary C–H functionalization, likely due to the enhanced electrophilicity of the benziodoxole radical. Benzylic methylene C–H bonds can be hydroxylated or amidated using unmodified hydroxyl benziodoxole oxidant Bl–OH under similar conditions. An ionic mechanism involving nucleophilic trapping of a carbocation intermediate by H_2_O or CH_3_CN cosolvent is presented.

## Abstract 2

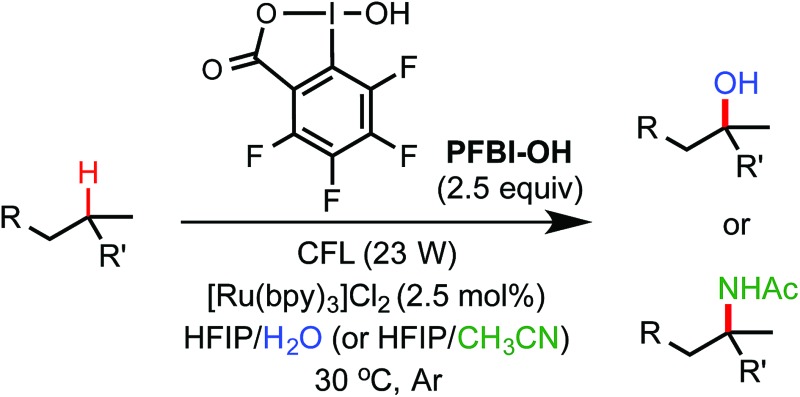
We report a unified photoredox-catalysis strategy for both hydroxylation and amidation of tertiary and benzylic C–H bonds.

## Introduction

**Scheme 1:**
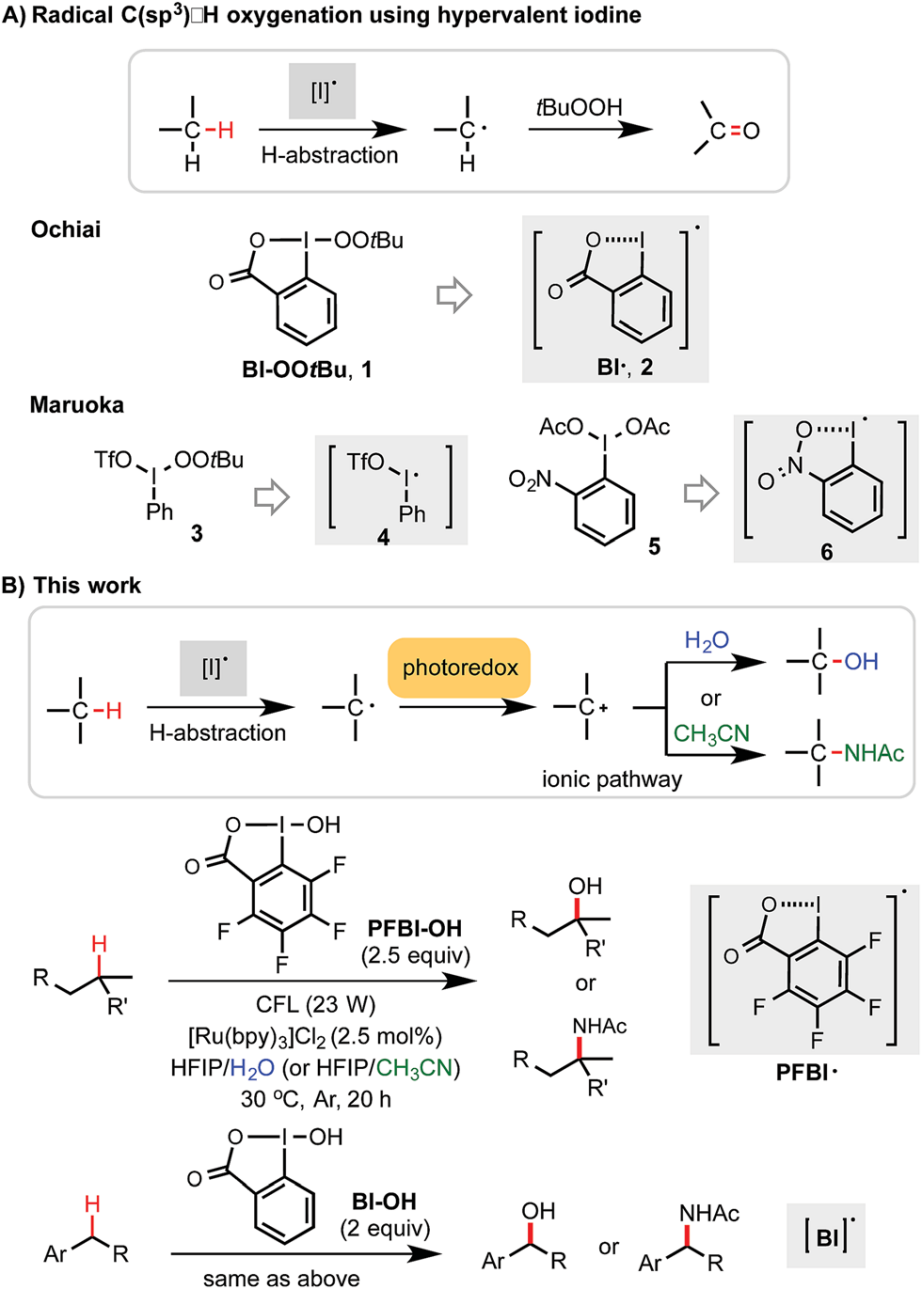
C(sp^3^)–H oxygenation and amination with hypervalent iodine(iii).

Methods for efficient and selective alkyl C–H oxidation could streamline the synthesis of fine chemicals, natural products, and drug metabolites.^1,2^ Despite rapid advances in the development of metal-catalyzed reactions^3^ and reagents,^4^ synthetically useful C(sp^3^)–H oxygenation chemistry is still in great demand.^5,6^ Recently, radical reactions mediated by hypervalent iodine(iii) reagents have emerged as viable means to oxygenate C(sp^3^)–H bonds under mild conditions.^7–11^ Ochiai first reported the oxidation of activated C(sp^3^)–H bonds of benzyl and allyl ethers to the corresponding esters using *t*-butylperoxy benziodoxole (Bl–OO*t*Bu, **1**) *via* H-abstraction by benziodoxole radical Bl˙ **2** (Scheme 1A).^9^ Maruoka elegantly demonstrated the use of acyclic iodane reagents **3** and **5** in the selective oxidation of unactivated methylene C–H bonds of simple alkanes to the corresponding ketones, effected by more reactive iodanyl radical intermediates **4** and **6**.^10^ Notably, Maruoka's oxygenation reactions proceed with a selectivity for secondary over tertiary C–H bonds. Herein, we report an efficient and broadly applicable photoredox-catalysis strategy for the selective hydroxylation of tertiary and benzylic C–H bonds using hydroxyl benziodoxoles as oxidant and H_2_O as cosolvent and hydroxylation reagent. This reaction system can be easily modulated to achieve tertiary and benzylic C–H amidation with high efficiency and selectivity using CH_3_CN as co-solvent and amidation reagent.

## Results and discussion

Previously, we discovered a visible light-promoted method for tertiary C–H azidation using Zhdankin reagent Bl–N_3_ **11** (see entry 1, Table 1), [Ru(bpy)_3_]Cl_2_ photosensitizer, and household compact fluorescent lamp (CFL) irradiation.^12–14^ We proposed a radical chain mechanism for this azidation reaction, beginning with formation of Bl radical **2** *via* single electron reduction of **11** by a photoexcited Ru(ii)* species. Bl˙ **2** then selectively abstracts a H atom from the substrate (*e.g.* 4-methylpentyl benzoate **7**), forming tertiary alkyl radical intermediate, which reacts with **11** to give C–H azidation product and regenerate radical **2**, propagating a radical chain reaction. Encouraged by these results, we questioned whether the reaction with the corresponding hydroxyl benziodoxole could offer C–H hydroxylation product under similar conditions.

**Table 1:** Tertiary C–H hydroxylation of **7** with hydroxyl benziodoxoles

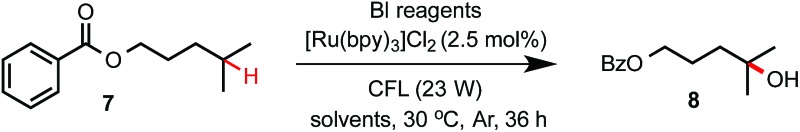			
Entry	Reagents (equiv.)	Solvents	Yield^*a*^ (%) **8**
1	Bl–N_3_ **11** (2)	HFIP	<1^*b*^
2	BI–OH **13** (2)	HFIP	<2
3	Bl–OH **13** (2)	HFIP/H_2_O (26/1)	29
4	4FBI–OH **14** (2)	HFIP/H_2_O (26/1)	32
5	4CF_3_BI–OH **15** (2)	HFIP/H_2_O (26/1)	38
6	TFBI–OH **16** (2)	HFIP/H_2_O (26/1)	46
7	PFBI–OH **17** (2)	HFIP/H_2_O (26/1)	51
8	4MOBI–OH **18** (2)	HFIP/H_2_O (26/1)	25
9	BI–OAc **12** (2)	HFIP/H_2_O (26/1)	18
**10**	****17**** **(2.5)**	**HFIP/H**_**2**_ **O (26/1)**	**64** ^*c*^
11	**17** (2.5)	HFIP/H_2_O (10/1)	55
12	**17** (2.5), O_2_ (1 atm)	HFIP/H_2_O (26/1)	29
13	**17** (2.5), Ir(ppy)_3_ (2.5 mol%)	HFIP/H_2_O (26/1)	<2
14	**17** (2.5), [Ru(bpz)_3_](PF_6_)_2_ (2.5 mol%)	HFIP/H_2_O (26/1)	<2
15	**17** (2.5), in darkness	HFIP/H_2_O (26/1)	<2
16	**17** (2.5), [Ru(bpy)_3_]Cl_2_ (1 mol%)	HFIP/H_2_O (26/1)	40
17	**17** (2.5)	HFIP	4
18	**17** (2.5)	DMSO/H_2_O (26/1)	<2
19	**17** (2.5)	HFIP/CH_3_CN (26/1)^*d*^	<2 (+10% of **10**)
**20**	****17**** **(2.5)**	**HFIP/CH**_**3**_ **CN (4/3)** ^*d*^	**<2 (+56% of 10)** ^*e*^
21	**13** (2.5)	HFIP/CH_3_CN (4/3)^*d*^	<2 (+8% of **10**)
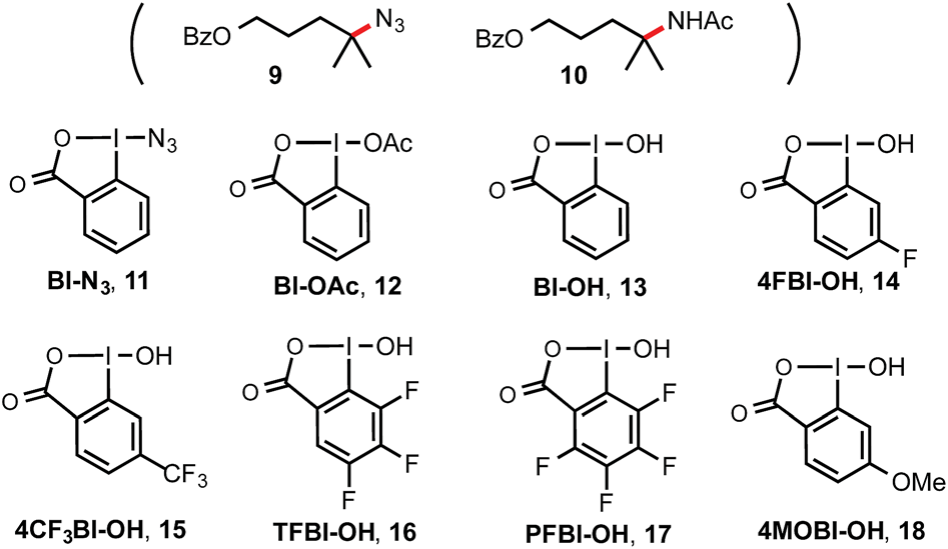			

^*a*^Isolated yield on a 0.2 mmol scale, *c* ∼ 50 mM, ACS grade of HFIP was used.

^*b*^58% of **9** was obtained.

^*c*^8% of **7** was recovered.

^*d*^Anhydrous HFIP and CH_3_CN dried over 4 Å molecular sieves were used.

^*e*^ *c* ∼ 30 mM, ∼10% of **7** was recovered. See ESI for more screening results.

As shown in Table 1, we commenced the investigation of tertiary C–H hydroxylation of **7** with Bl–OH **13** under the irradiation of CFL (23 W) using [Ru(bpy)_3_]Cl_2_ as photocatalyst in hexafluoroisopropanol (HFIP) at 30 °C. Our previous work has shown that Bl–OH **13** can be used to generate Bl˙ **2** under similar conditions for a Minisci-type C–H alkylation reaction of *N*-heteroarenes with alkyl boronic acids.^15,16^ However, subjecting **7**, **13**, and [Ru(bpy)_3_]Cl_2_ to CFL irradiation produced only trace amount of the desired hydroxylation product **8**, with **7** largely unconsumed (entry 2). However, adding H_2_O to the reaction increased the yield of **8** to 29% (entry 3). Our previous work has indicated that the spin density of Bl˙ is delocalized on both O and I atoms and that Bl˙ is more stable than benzoyloxy radical BzO˙.^15^ The stability of Bl˙ may explain the observed weak reactivity for H-abstraction and the low conversion of **7**.^17,18^ We speculated that installation of electron-withdrawing groups on the aryl motif of Bl would increase its electrophilicity, and enhance its H-abstraction reactivity. As shown in entries 4–7, Bl–OH analogs **14–17** with different electron-withdrawing groups were prepared and evaluated (see ESI† for more details).^19^ We were pleased to find that these Bl–OH analogs provided improved results, and hydroxyl perfluorobenziodoxole (PFBl–OH, **17**) gave the best yield.^20,21^ A 64% isolated yield of **8** was obtained when 2.5 equiv. of **17** was used (entry 10). Regarding the optimization of this hydroxylation reaction, we note: (1) addition of H_2_O is critical to obtain high yield (see entries 10 *vs.* 17). (2) **17** has high polarity and only dissolves well in polar solvents such as HFIP, DMSO, DMF; HFIP gives significantly better results than other solvent tested; (3) under O_2_ atmosphere, the reaction gave significantly diminished yield (entry 12); (4) in the dark, the reaction gave no product (entry 15); (5) only trace amount (<3% yield) of methylene C–H hydroxylation side product was detected. Interestingly, when the reaction was performed in mixed HFIP/CH_3_CN solvents (4/3) under similar conditions we obtained 56% yield of the C–H aminated product **10** with excellent selectivity (entry 20).

**Scheme 2:**
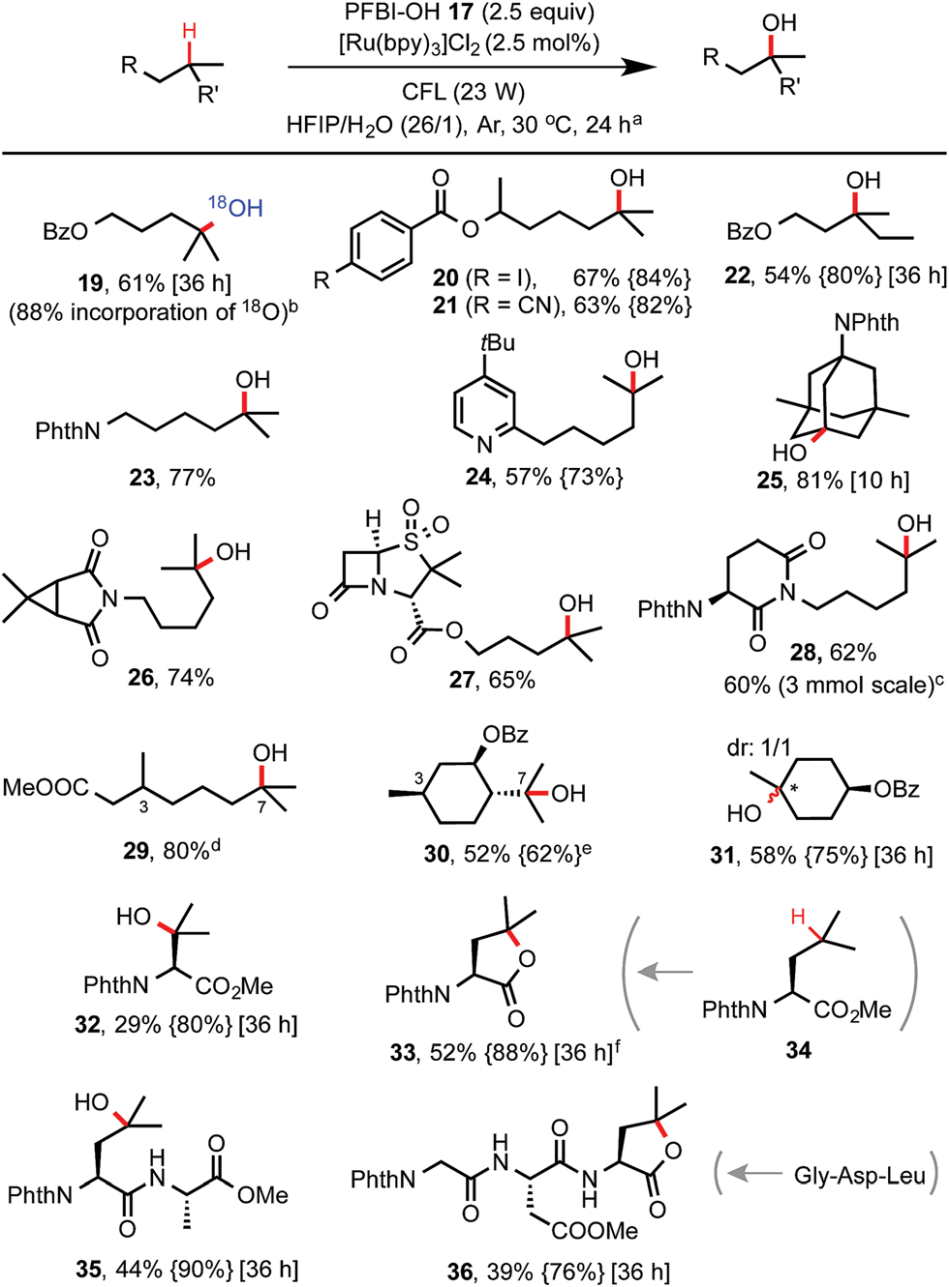
Substrate scope of tertiary C–H hydroxylation with PFBl–OH **17**. (a) Isolated yield on 0.2 mmol scale under the standard conditions, *c* ∼ 50 mM. For reaction with <85% conversion of starting material, yields based on recovered SM (BRSM) were given in braces. (b) H_2_^18^O (97% ^18^O) was used. (c) 3 mmol scale, 46 h. (d) C_3_ hydroxylation product <5%. (e) No C_3_ hydroxylation product was detected. (f) No free OH product was obtained.

With optimized conditions in hand, we investigated the substrate scope of this C–H hydroxylation reaction (Scheme 2). In general, the reaction proceeds with excellent selectivity for tertiary C–H bonds and in good yield. Common functional groups including CN, iodo, esters, amide, imide and pyridine moiety are tolerated. When reaction of **7** was performed in a mixture of HIFP and H_2_^18^O (97% of ^18^O), ^18^O-labelled product **19** was obtained. C–H hydroxylation of sulbactam and thalidomide derivatives (see **27** and **28**) bearing β-lactam and imide groups proceeded in good yield. **28** was obtained in 60% yield on a gram scale. Both steric and electronic factors influence the reactivity of tertiary C–H bonds. For instance, tertiary C–H hydroxylation took place selectively at the more distal 3° carbon of **29**. Hydroxylation of the sterically hindered and electron-poor tertiary C–H bond of phthaloyl valine methyl ester gave **32** in moderate yield. In comparison, C–H hydroxylation of leucine methyl ester **34** provided lactone product **33** in 52% yield. Moreover, short peptide substrates (see **35** and **36**) can be C–H hydroxylated on the Leu residue with excellent selectivity under standard conditions.

**Scheme 3:**
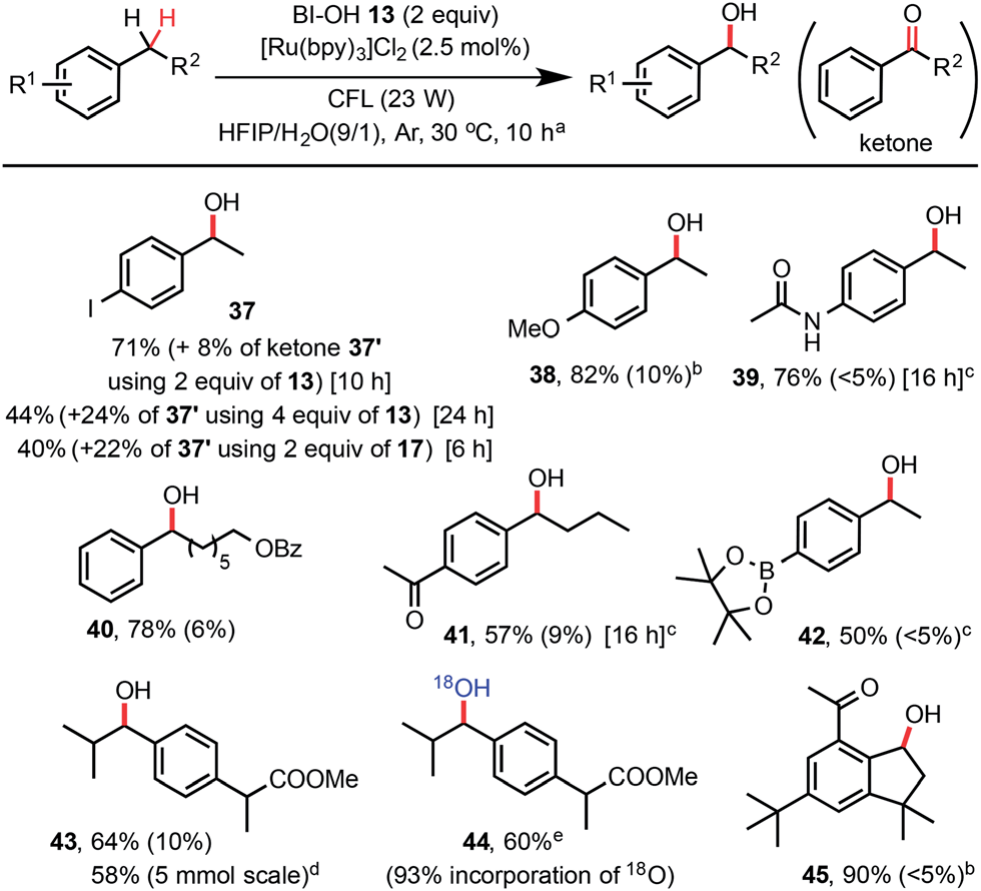
Substrate scope of benzylic C–H hydroxylation with Bl–OH **13**. (a) Isolated yield on a 0.2 mmol scale under standard conditions, *c* ∼ 45 mM, yield of ketone by-product was given in parentheses. (b) 1.5 equiv. of **13** was used. (c) 4 equiv. of **13** was used. (d) 5 mmol scale, 20 h. (e) H_2_^18^O (97% ^18^O) was used.

While a number of methods for oxidation of benzylic methylene groups to ketones have been developed,^22^ practical methods for C–H hydroxylation of these methylene groups to benzyl alcohols are sparse.^23^ As shown in Scheme 3, we subjected 4-ethylphenyliodide to our standard C–H hydroxylation conditions with PFBl-OH **17**, and obtained the alcohol product **37** in 40% yield along with 22% of ketone **37′** and other unidentified by-products. We were delighted to find that use of 2 equiv. of Bl–OH **13** under the same conditions gave **37** in 71% yield along with 8% of ketone. More ketone **37′** (24%) was obtained when 4 equiv. of Bl–OH was used for extended reaction time (24 h). This Bl–OH mediated benzylic C–H hydroxylation exhibited excellent chemo-selectivity and broad substrate scope. The reaction tolerates functional groups such as iodo, ketone, amide, even pinacolyl boronate ester (see **42**). Electron-deficient arenes are less reactive and require the use of 4 equiv. of Bl–OH **13** (see **41**). Electron-rich substrates give good yield with 1.5 equiv. of Bl–OH (see **38**). Reaction of ibuprofen methyl ester gave **43** in 64% yield without the formation of tertiary C–H hydroxylated product. The same reaction in H_2_^18^O gave ^18^O-labelled product **44**. Reaction of natural product celestolide gave product **45** in excellent yield.

**Scheme 4:**
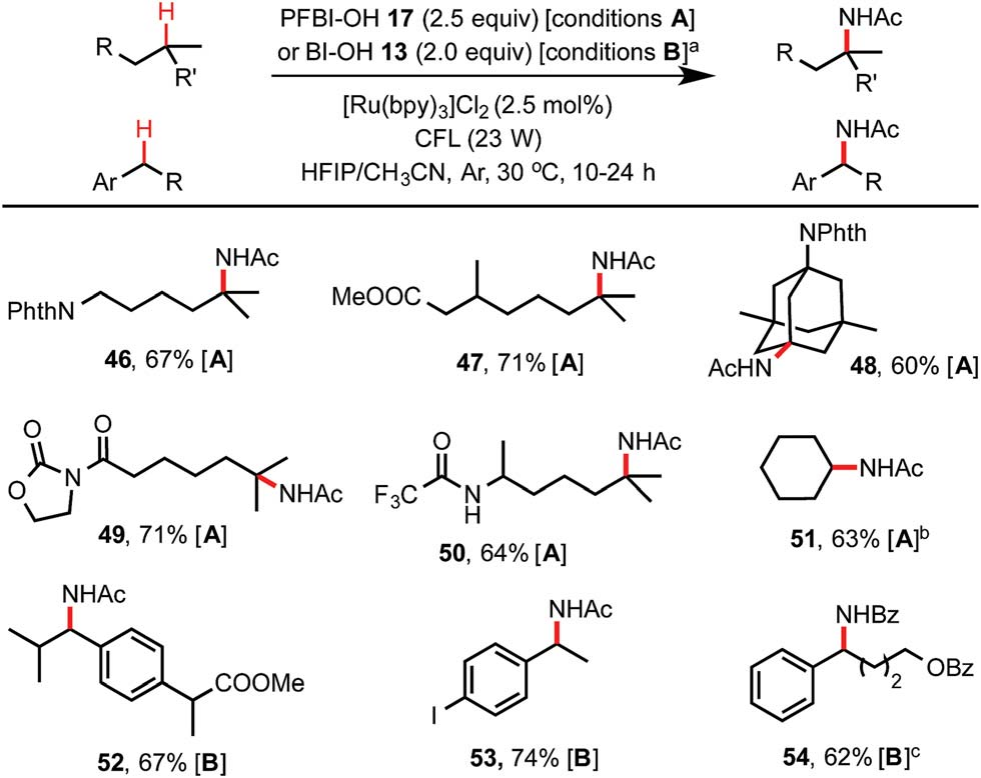
C(sp^3^)–H amidation with **13** or **17**. (a) Conditions **A** for tertiary C–H amidation, HFIP/CH_3_CN (4/3), *c* ∼ 30 mM, 24 h; conditions **B** for benzylic C–H amidation, HFIP/CH_3_CN (8/3), *c* ∼ 35 mM, 10 h. Anhydrous HFIP and CH_3_CN dried over 4 Å molecular sieves were used. Isolated yield on a 0.2 mmol scale. (b) 1 equiv. of cyclohexane was used, 0.5 mmol scale. (c) PhCN was used as cosolvent, HFIP/PhCN (8/5), *c* ∼ 30 mM, 10 h.

As shown in Scheme 4, by simply switching to the HFIP/CH_3_CN solvents, this hydroxyl benziodoxole-mediated reaction system provides an excellent method for C(sp^3^)–H amidation, which remains a challenging transformation for C–H functionalization chemistry.^24,25^ Tertiary C–H amidation with PFBl–OH **17** and benzylic C–H amidation with Bl–OH **13** proceeded with yields and regio-selectivity similar to the corresponding C–H hydroxylations carried out in HFIP/H_2_O solvents. Unactivated methylene C–H bonds were generally unreactive with either **13** or **17**. However, cycloalkanes such as cyclohexane were efficiently amidated with **17** (see **51**), probably due to their slightly more activated C–H bonds and more favorable kinetics.^25*b*^ Product **54** carrying a benzamide group was obtained in good yield using HFIP/PhCN solvent under similar conditions. Generally, the competing C–H hydroxylation reactions were well suppressed (<2% yield) in HFIP/nitrile solvents.

**Scheme 5:**
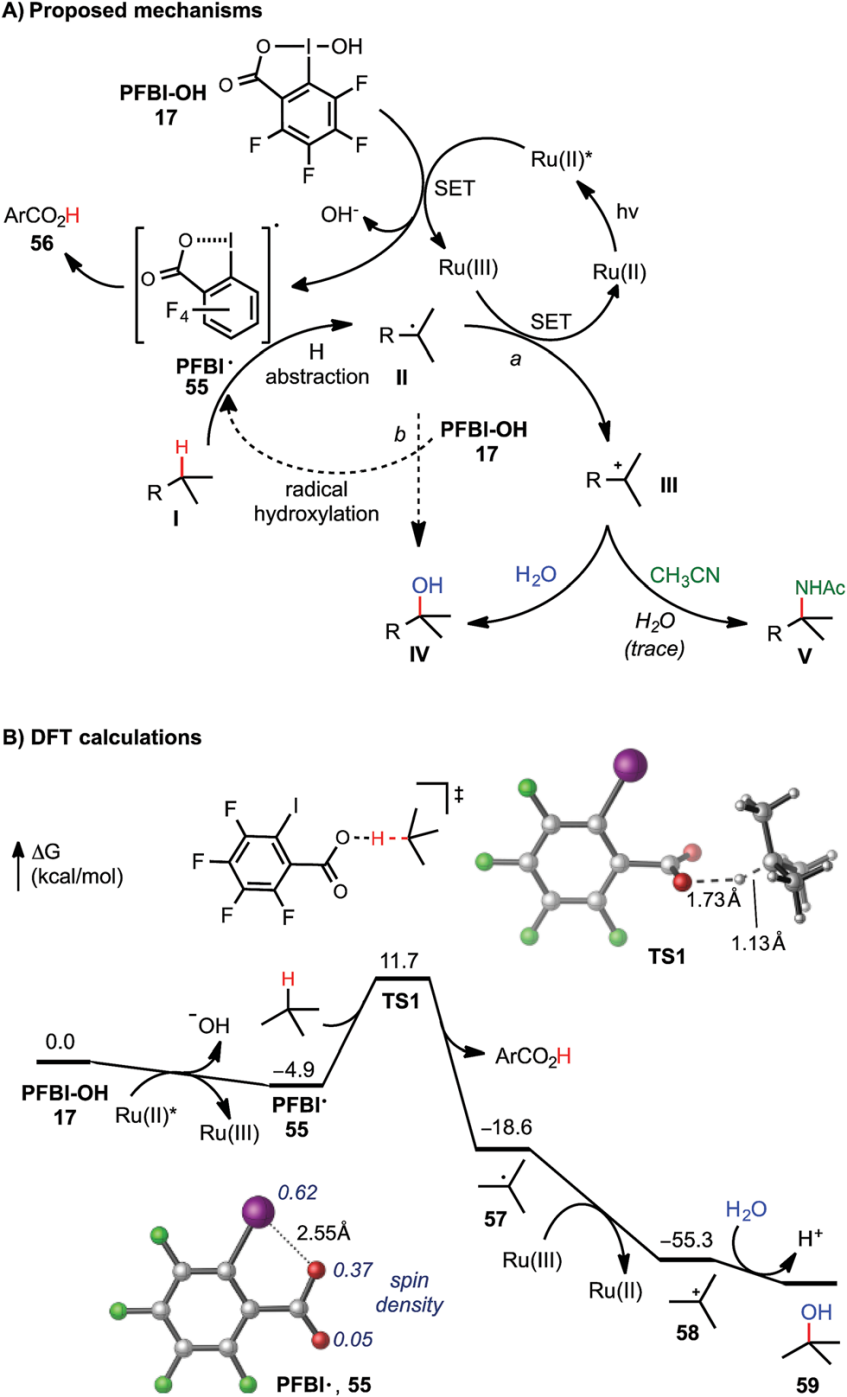
Mechanistic consideration of C(sp^3^)–H functionalization with PFBl–OH. DFT calculations were performed at the M06-2X/6-311++G(d,p)-SDD/SMD(HFIP)//M06-2X/6-31+G(*d*)-SDD level of theory. All energies are in kcal mol^–1^. See ESI† of DFT calculations with Bl–OH.

As shown in Scheme 5A, two C–O bond forming mechanisms were initially considered for this C–H hydroxylation reaction: nucleophilic trapping of a carbocation intermediate with H_2_O (pathway a) or a radical chain reaction with the hydroxyl benziodoxole reagents (pathway b).^23*c*^ In contrast to the large quantum yield *Φ* observed in our previously reported visible light-promoted C–H azidation reaction with Bl–N_3_ **11**,^12^ a small *Φ* (0.85, measured by Yoon's method^26^) of the C–H hydroxylation reaction of **7** with PFBl–OH **17** suggested a non-radical chain mechanism (see ESI† for details). The dependence of the reactivity on the H_2_O co-solvent and the formation of amidation product in the presence of CH_3_CN product strongly support ionic pathway a. Stern–Volmer experiments confirmed that the excited state of photocatalyst [Ru(bpy)_3_]Cl_2_ can be quenched by the addition of PFBl–OH **17**, while no obvious luminescence change of the photocatalyst was observed in the presence of substrate **7** (see ESI† for details).^27^

The mechanism of tertiary C–H hydroxylation with PFBl–OH **17** likely begins with single electron transfer (SET) from photoexcited Ru(ii)* to PFBl–OH **17**, generating radical PFBl˙ **55**. Radical **55** abstracts a H atom from alkane substrate **I**, forming tertiary carbon radical **II**. **II** can be oxidized by the Ru(iii) species, forming tertiary carbocation intermediate **III**, and regenerating the photocatalyst. Finally, tertiary carbocation intermediate **III** is attacked by H_2_O to give hydroxylated product **IV**. Trapping of **III** by CH_3_CN can give the amidated product **V** following a Ritter reaction-type mechanism.^25*b*^ We speculate that Bl–OH mediated benzylic C–H hydroxylation and amidation proceeds through a similar mechanism, involving cleavage of benzylic C–H bond with less electrophilic Bl˙ **2**.

This mechanism is supported by density functional theory (DFT) calculations using *t*-butane as a model substrate (Scheme 5B). The initial SET reduction of PFBl–OH **17** to PFBl˙ **55** is significantly more exergonic than the SET with Bl–OH **13** to Bl˙ **2** (Δ*G* = –4.9 kcal mol^–1^ with PFBl–OH **15** *vs.* –0.9 kcal mol^–1^ with Bl–OH **13**).^28,29^ With its spin density delocalized over the O and I atoms, PFBl˙ **55** undergoes facile H-abstraction of *t*-butane through an O-centered transition state (**TS1**) with a Δ*G* ^‡^ of 16.6 kcal mol^–1^ to give *t*Bu˙.^30^ This H-abstraction process is promoted by the electron-deficient perfluoroaryl group. The corresponding H-abstraction with Bl˙ **2** requires a noticeably higher barrier of 18.2 kcal mol^–1^ (see ESI†). The subsequent oxidation of *t*Bu˙ by Ru(iii) to *t*butyl cation is highly exothermic. Finally, the *t*butyl cation is trapped with H_2_O, providing *t*BuOH. Taken together, the DFT calculations indicated the perfluorinated analogue PFBl–OH promotes both the initial SET reduction and the H-abstraction steps in the catalytic cycle of the tertiary C–H hydroxylation.

## Conclusions

In summary, we have developed a unified photoredox-catalysis strategy for both C(sp^3^)–H hydroxylation and amidation using hydroxyl benziodoxole oxidant. This strategy allows the selective functionalization of tertiary and benzylic methylene C–H bonds under mild conditions. These reactions exhibit excellent substrate scope, and offer an efficient and convenient method for late-stage derivatization of complex substrates. Distinct from the radical chain mechanism invoked for our previous tertiary C–H azidation reaction with azido benziodoxole, we propose a new product-forming pathway: photoredox catalyzed formation of a carbocation intermediate, followed by nucleophilic trapping with H_2_O or nitrile cosolvent. Further expansion of the nucleophile scope and the functionalization of unactivated methylene C–H bonds using this reaction system are currently under investigation.

## Conflicts of interest

There are no conflicts to declare.
