# Supplementary material for: A unified photoredox-catalysis strategy for C(sp3)–H hydroxylation and amidation using hypervalent iodine
Source: Chem Sci. 2017 Sep 4;8(10):7180–5. doi: 10.1039/c7sc02773g (PMC5635418; doi:10.1039/c7sc02773g)
Supplement: Supplementary file 1 [file SC-008-C7SC02773G-s001.pdf]

Electronic Supplementary Material (ESI) for Chemical Science.
This journal is © The Royal Society of Chemistry 2017

Supporting Information

A Unified Photoredox-Catalysis Strategy for C(sp^3)−H Hydroxylation and Amidation Using Hypervalent Iodine

Guo-Xing Li,[a] Cristian A. Morales-Rivera,[b] Fang Gao,[a] Yaxin Wang,[a] Gang He,[a] Peng Liu,*[b] and Gong Chen*[a,c]

[a]State Key Laboratory and Institute of Elemento-Organic Chemistry, Collaborative Innovation Center of Chemical Science and Engineering (Tianjin), Nankai University, Tianjin 300071, China

[b]Department of Chemistry, University of Pittsburgh, Pittsburgh, Pennsylvania 15260, USA

[c]Department of Chemistry, The Pennsylvania State University, University Park, Pennsylvania 16802, USA

Content

1. Reagents, S2
2. Instruments, S2
3. Synthesis of hypervalent iodines, S3
4. Synthesis of substrates for hydroxylation and amidation, S6
5. Optimization for tertiary C-H hydroxylation, S18
6. General procedures and substrate scope of tertiary C-H hydroxylation with PFBl-OH, S18
7. General procedures and substrate scope of benzylic C-H hydroxylation with Bl-OH, S27
8. Scale-up reaction for hydroxylation, S33
9. General procedures and substrate scope of C(sp^3)-H amidation with PFBl-OH, S34
10. General procedures and substrate scope of benzylic C-H amidation with Bl-OH, S37
11. Measurement of quantum yield (Φ) for C-H hydroxylation of 7, S39
12. The luminescence quenching (Stern-Volmer) experiments, S42
13. DFT calculations, S43
14. References, S57
15. ^{1}H-NMR, ^{13}C-NMR and ^{19}F-NMR spectra, S60

1. Reagents

All commercial materials were used as received unless otherwise noted. DCM was dried by distillation over CaH_2. THF was dried by distillation over sodium/benzophenone. Anhydrous CH_3CN was purchased from Acros Organics and stored under nitrogen atmosphere. TLC were performed on silica gel Huanghai HSGF254 plates and visualization of the developed chromatogram was performed by fluorescence quenching (λ_{max} = 254 nm). Flash chromatography was performed using Silica gel (200-300 mesh) purchased from Qingdao Haiyang Chemical Co., China. Zhdankin reagent (azidobenziodoxole, BI-N_3, **11**)[1], acetoxybenziodoxole (BI-OAc, **12**)[1], hydroxylbenziodoxole (BI-OH, **13**)[1], 4-methoxyl hydroxylbenziodoxole (4MOBI-OH, **18**)[2], and $[Ru(bpz)_3](PF_6)_2$[3] were synthesized according to reported procedures and used as freshly prepared. $[Ru(bpy)_3]Cl_2$ (98%, Ru＞15.75%, Energy Chemical), HFIP (99.0%, ACS grade, J&K Chemical,) and $H_2{}^{18}O$ (97% ^{18}O, J&K Chemical) were used as received unless otherwise noted.

2. Instruments

NMR spectra were recorded on Bruker AVANCE AV 400 instruments and all NMR experiments were reported in units, parts per million (ppm). Peaks recorded are relative to internal standards: TMS (δ = 0.00) for ^1H and $CDCl_3$ (δ = 77.00) for ^{13}C spectra. Multiplicities are recorded as: s = singlet, d = doublet, t = triplet, dd = doublet of doublets, br s = broad singlet, m = multiplet. High resolution ESI mass experiments were operated on a Waters LCT Premier instrument. All reactions were carried out in a 22 mL glass vial (Thermo SCIENTIFIC National B7999-5, made from clear borosilicate glass), sealed with PTEF cap on bench top.

3. Synthesis of hypervalent iodines

3.1 Procedure for synthesis of 14 and 15:

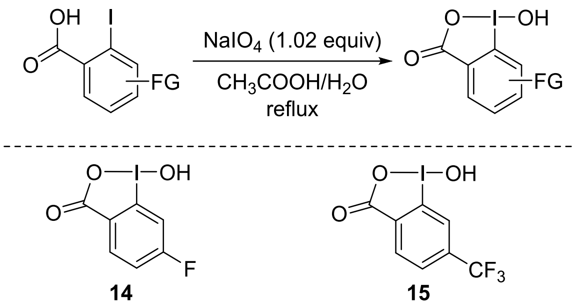

Scheme S1

To a solution of AcOH in water (30% v/v, 30 mL) $NaIO_4$ (20.4 mmol, 1.02 equiv) and corresponding benzoic acid (20 mmol, 1.00 equiv) were added at room temperature. The suspension mixture was vigorously stirred and refluxed for 4 h under air. The reaction mixture was cooled to room temperature then poured into cold water (50 mL). The mixture is then filtered and the filter cake was further washed with water and acetone. The residue was collected and dried under high vacuum in dark to give the desired product.

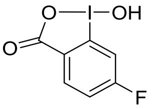

14

Compound **14** was obtained in 75% yield as white solid. M.p. = 222-223 ^{o}C (decomp.); IR (KBr) : ῡ = 3083, 2416, 1742, 1609, 1472, 1210, 885, 781, 668 cm^{-1}; **^{1}H NMR** (400 MHz, DMSO-*d*$_6$) δ 8.24 (br s, 1H), 8.00 (dd, *J* = 8.4, 5.3 Hz, 1H), 7.58-7.53 (m, 2H); **^{13}C NMR** (101 MHz, DMSO-*d*$_6$) δ 166.85, 166.07 (d, *J* = 255.3 Hz), 132.93, 128.33, 122.86, 118.06 (d, *J* = 20.8 Hz), 113.60 (d, *J* = 28.6 Hz); **^{19}F NMR** (376 MHz, DMSO-*d*$_6$) δ -105.53 (s, 1F); **HRMS** Calcd for $C_7H_5FIO_3$ $[M+H]^+$: 282.9267, Found: 282.9260.

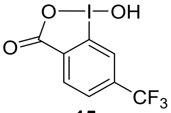

15

Compound **15** was obtained in 67% yield as white solid. M.p. = 244-245 ^{o}C (decomp.);

IR (KBr) : ῦ = 3050, 2440, 1830, 1562, 1398, 1321, 1186, 1133, 782, 700 cm^{-1}; **^{1}H NMR** (400 MHz, DMSO-*d_6*) δ 8.40 (br s, 1H), 8.18 (d, *J* = 7.9 Hz, 1H), 8.09 (d, *J* = 8.1 Hz, 1H), 8.04 (br s, 1H); **^{13}C NMR** (101 MHz, DMSO-*d_6*) δ 166.99, 135.83, 134.42 (q, *J* = 32.5 Hz), 132.38, 128.08 (q, *J* = 7.1 Hz), 123.88 (q, *J* = 273.3 Hz), 123.65 (q, *J* = 3.8 Hz), 122.52; **^{19}F NMR** (376 MHz, DMSO-*d_6*) δ -61.24 (s, 3F); **HRMS** Calcd for $C_8H_5F_3IO_3$ $[M+H]^+$: 332.9235, Found: 332.9228.

3.2 Procedure for synthesis of 16 and 17:

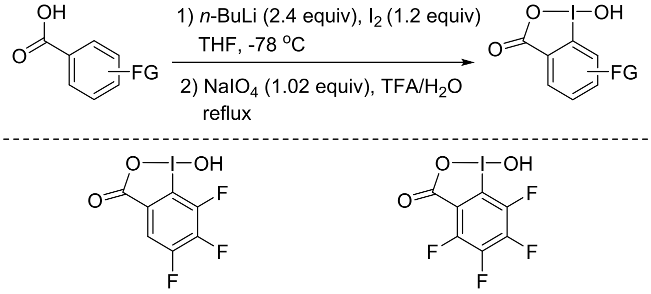

Scheme S2

A modified literature procedure was followed for the synthesis of hypervalent iodine **16** and **17**.[4] To a solution of the corresponding benzoic acid (77 mmol, 1.0 equiv) in anhydrous THF, *n*-Butyllithium (77 mL, 2.4 M in hexanes, 185 mmol, 2.4 equiv) was added dropwisely at -78 °C under Ar atmosphere. The resulting suspension was stirred at -78 °C for 3 h. Then a solution of iodine (93 mmol, 1.2 equiv) in anhydrous THF (50 mL) was added slowly until the brown color of the iodine persisted in the solution. After the addition was completed, the reaction mixture was warmed to room temperature. The reaction was carefully quenched by dropwise addition of saturated aqueous solution of $NaHSO_3$ until the brown color just faded (Note: excessive addition of $NaHSO_3$ could cause low yield, or even no product was obtained), and the reaction mixture was concentrated *in vacuo*. To the residue, water (100 mL) and ethyl acetate (80 mL) was added. The resulting mixture was acidified with aqueous 2 M H_2SO_4 to pH 1-2 under vigorous stirring at 0 °C. The organic phase was separated, and the aqueous phase was extracted with ethyl acetate (80 mL x 2). The combined organic phase was washed with saturated aqueous $NaHSO_3$ solution (5 mL), water (50 mL), brine (20 mL), dried over Na_2SO_4 and filtrated. The filtrate was concentrated *in vacuo* and the residue was precipitated with petroleum ether (60 mL) for 4 h. The resultant precipitate was collected by filtration, washed with petroleum ether (20 mL), the

desired product was obtained as a white solid and used without further purification . The corresponding 2-iodobenzoic acid (50 mmol) and $NaIO_4$ (10.9 g, 51 mmol) were added into a solution of TFA (trifluoroacetic acid, 100 mL) in water (100 mL), the resulting mixture was heated at reflux for 3 h then cooled to 35 ^{o}C. The resultant precipitate was collected by filtration, carefully washed with water (400 mL) and petroleum ether (50 mL). The residue was collected and dried under high vacuum to give the desired product.

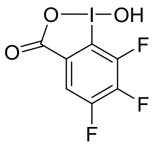

16

Compound **16** was obtained in 62% yield (for 2 steps) as white solid. M.p. = 219-220 ^{o}C (decomp.); IR (KBr) : $\tilde{\nu}$ = 3652, 2994, 1649, 1499, 1354, 1128, 1059, 793, 739 cm^{-1}; **^{1}H NMR** (400 MHz, DMSO-d_6) δ 13.87 (br s, 1H), 7.79 (ddd, *J* = 10.7, 7.3, 2.1 Hz, 1H); **^{13}C NMR** (101 MHz, DMSO-d_6) δ 165.65, 152.25 (dd, *J* = 253.2, 10.6 Hz), 151.59 (dd, *J* = 259.0, 12.7 Hz), 143.87 (dt, *J* = 257.2, 16.4 Hz), 130.49 (dd, *J* = 5.9, 2.0 Hz), 114.62 (dd, *J* = 18.6, 2.3 Hz), 100.23 (dd, *J* = 12.3, 1.8 Hz); **^{19}F NMR** (376 MHz, DMSO-d_6) δ -129.90 (dd, *J* = 20.4, 8.8 Hz, 1F), -133.67 (dd, *J* = 20.8, 8.8 Hz, 1F), -151.51 (dd, *J* = 20.4, 20.8 Hz, 1F); **HRMS** Calcd for $C_7HF_3IO_3$ $[M\text{-}H]^-$: 316.8922, Found: 316.8925.

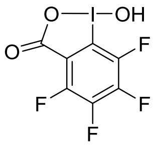

17

Compound **17** was obtained in 58% yield (for 2 steps) following the above procedure. Spectra data are consistent with those reported in the literature.[4]

4. Synthesis of substrates for hydroxylation and amidation:

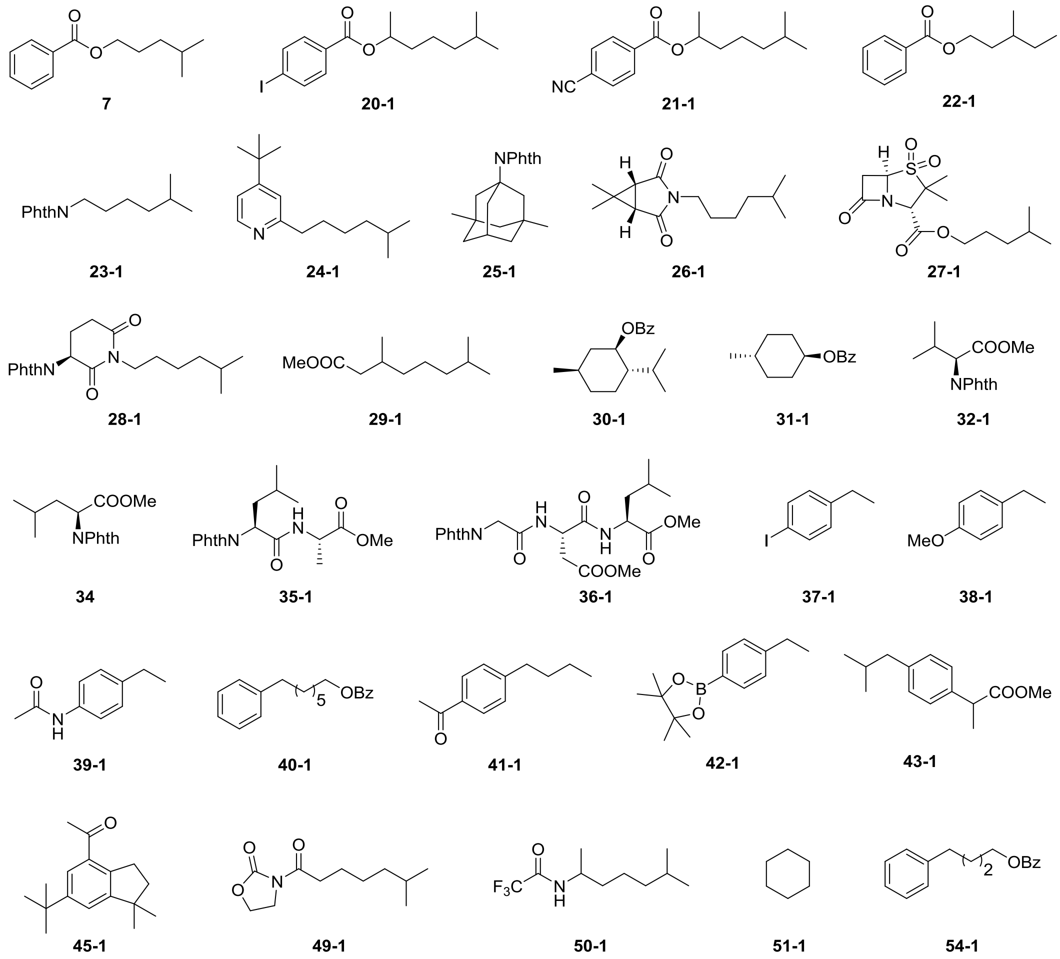

Scheme S3 List of all substrates used in this study

Compounds **37-1**, **38-1**, **39-1**, **41-1**, **42-1**, **45-1**, **51-1** were commercial available and used as received.

4.1 General procedure for synthesis 7, 20-1, 21-1, 22-1, 31-1, 40-1 and 54-1:

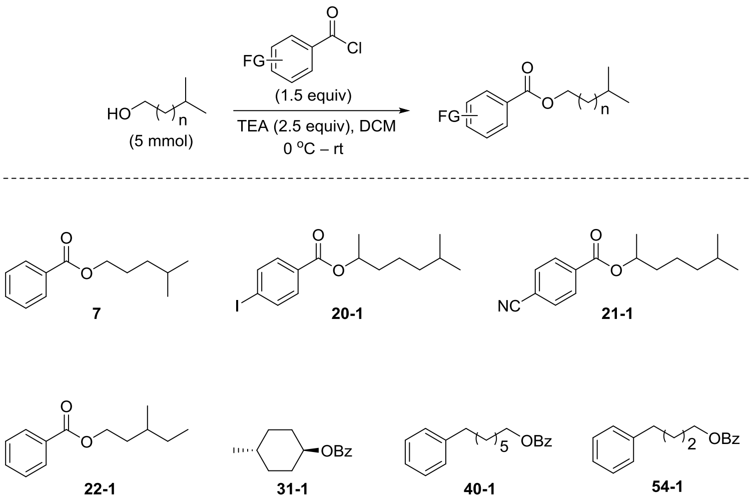

Scheme S4

To a solution of alcohols (5 mmol) and trimethylamine (TEA, 12.5 mmol, 2.5 equiv) in CH_2C1_2 (50 mL) was added appropriate acyl chloride (7.5 mmol, 1.5 equiv) dropwisely at 0 °C. The reaction mixture was warmed to room temperature and stirred until corresponding alcohol was completely consumed monitoring by TLC. The mixture was diluted with DCM (200 mL), washed with saturated aqueous $NaHCO_3$ solution (20 mL), 1 M HCl (20 mL), water (20 mL) and brine (20 mL). The organic layer was dried over Na_2SO_4 and filtrated. The filtrate was concentrated *in vacuo* and the residue was purified by flash chromatography on silica gel to afford desired product.

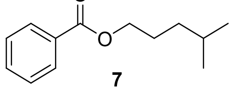

7

Compound **7** was prepared in 92% yield following the general procedure. Spectra data are consistent with those reported in the literature.[5]

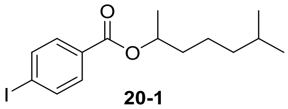

20-1

Compound **20-1** was prepared in 89% yield as a colorless oil following the general

procedure. IR (KBr) : ῦ = 3465, 2953, 1717, 1586, 1271, 1108, 1008, 753 cm^{-1}; **^{1}H NMR** (400 MHz, $CDCl_3$) δ 7.80 (d, *J* = 8.4 Hz, 2H), 7.74 (d, *J* = 8.4 Hz, 2H), 5.18-5.10 (m, 1H), 1.76-1.67 (m, 1H), 1.62-1.48 (m, 2H), 1.43-1.35 (m, 2H), 1.33 (d, *J* = 6.2 Hz, 3H), 1.24-1.16 (m, 2H), 0.86 (d, *J* = 6.6 Hz, 6H); **^{13}C NMR** (101 MHz, $CDCl_3$) δ 165.57, 137.53, 130.91, 130.29, 100.35, 72.02, 38.61, 36.12, 27.73, 23.12, 22.51, 22.47, 19.98; **HRMS** Calcd for $C_{15}H_{21}IO_2$ $[M]^+$: 360.0586, Found: 360.0581.

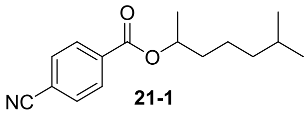

21-1

Compound **21-1** was prepared in 97% yield as a white solid following the general procedure. M.p. = 47-48 °C; IR (KBr) : ῦ = 2954, 2232, 1721, 1464, 1277, 1110, 862, 768 cm^{-1}; **^{1}H NMR** (400 MHz, $CDCl_3$) δ 8.14 (d, *J* = 8.2 Hz, 2H), 7.74 (d, *J* = 8.2 Hz, 2H), 5.26-5.09 (m, 1H), 1.80-1.67 (m, 1H), 1.66-1.48 (m, 2H), 1.43-1.29 (m, 5H), 1.27-1.14 (m, 2H), 0.86 (d, *J* = 6.6 Hz, 6H); **^{13}C NMR** (101 MHz, $CDCl_3$) δ 164.51, 134.68, 132.12, 129.98, 118.03, 116.11, 72.90, 38.62, 36.09, 27.76, 23.13, 22.50, 22.46, 19.94; **HRMS** Calcd for $C_{16}H_{21}NO_2$ $[M]^+$: 259.1572, Found: 259.0933.

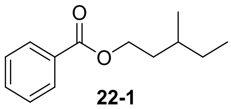

22-1

Compound **22-1** was prepared in 84% yield following the general procedure. Spectra data are consistent with those reported in the literature.[5]

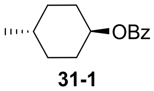

31-1

Compound **31-1** was prepared in 78% yield following the general procedure. Spectra data are consistent with those reported in the literature.[6]

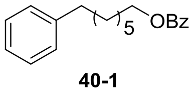

40-1

Compound **40-1** was prepared in 70% yield as a colorless oil following the general

procedure. IR (KBr) : ῡ = 3424, 2929, 1719, 1452, 1274, 1114, 747, 710 cm^{-1}; **^{1}H NMR** (400 MHz, $CDCl_3$) δ 8.06-8.03 (m, 2H), 7.58-7.53 (m, 1H), 7.46-7.42 (m, 2H), 7.29-7.25 (m, 2H), 7.19-7.15 (m, 3H), 4.31 (t, *J* = 6.6 Hz, 2H), 2.60 (t, *J* = 7.6 Hz, 2H), 1.79-1.72 (m, 2H), 1.66-1.59 (m, 2H), 1.46-1.34 (m, 6H); **^{13}C NMR** (101 MHz, $CDCl_3$) δ 166.46, 142.56, 132.64, 130.36, 129.38, 128.24, 128.16, 128.09, 125.46, 64.91, 35.79, 31.28, 29.03, 29.01, 28.57, 25.85; **HRMS** Calcd for $C_{20}H_{24}O_2Na$ $[M+Na]^+$: 319.1674, Found: 319.1661.

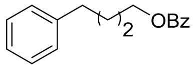

54-1

Compound **54-1** was prepared in 74% yield following the general procedure. Spectra data are consistent with those reported in the literature.[7]

4.2 Procedure for synthesis of phthalimide 23-1 and 25-1:

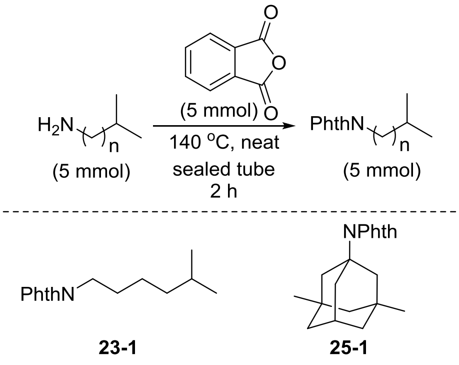

Scheme S5

The corresponding amine (5 mmol, 1.0 equiv) and phthalic anhydride (5mmol, 1.0 equiv) were heated at 140 ℃ in a sealed tube equipped with a stir bar for 2 h. After been cooled to room temperature, the reaction mixture was dissolved in ethyl acetate (200 mL), washed with water (20 mL), dried over Na_2SO_4 and filtrated. The filtrate was concentrated *in vacuo* and the residue was purified by flash chromatography on silica gel (ethyl acetate/petroleum ether = 5:95 (v/v)) to afford the desired product.

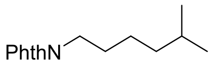

23-1

Compound **23-1** was prepared in 82% yield following the general procedure. Spectra data are consistent with those reported in the literature.[6]

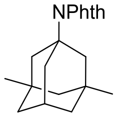

25-1

Compound **25-1** was prepared in 78% yield following the general procedure. Spectra data are consistent with those reported in the literature.[8]

4.3 Synthesis of compound 24-1:

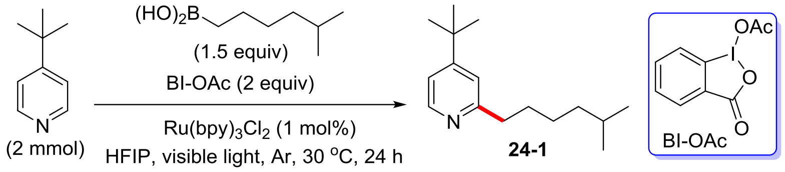

Scheme S6

4-(*tert*-butyl)pyridine (270 mg, 2 mmol, 1.0 equiv), (5-methylhexyl)boronic acid (432 mg, 3 mmol, 1.5 equiv) and BI-OAc (1.22 g, 4 mmol, 2.0 equiv) were added to a solution of $Ru(bpy)_3Cl_2$ (0.02 mmol, 0.01 equiv) in HFIP (5 mL). The reaction vial was purged with Ar for 1 min and then the mixture was stirred at 30 °C under the irradiation of fluorescent light for 48 h. The solvent was removed *in vacuo* and the residue was dissolved in DCM (30 mL). To the solution was added K_2CO_3 (approximate 1.5 g), and the resulting mixture was vigorously stirred for 5 min. Then the mixture was filtrated through a pad of Celite and washed with DCM. The filtrate was concentrated *in vacuo* and the residue was purified by flash chromatography on silica gel (eluted with ethyl acetate/petroleum ether = 5:95 (v/v)) to afford the desired product **24-1** as a colorless oil (289 mg, 62%). IR (KBr) : ṽ = 2957, 2867, 1600, 1465, 836, 743 cm^{-1}; ^{1}**H NMR** (400 MHz, $CDCl_3$) δ 8.42 (d, *J* = 5.3 Hz, 1H), 7.12 (d, *J* = 1.5 Hz, 1H), 7.08 (dd, *J* =

5.3, 1.8 Hz, 1H), 2.76 (t, J = 7.8 Hz, 2H), 1.74-1.67 (m, 2H), 1.59-1.49 (m, 1H), 1.40-1.33 (m, 2H), 1.30 (s, 9H), 1.24-1.19 (m, 2H), 0.86 (d, J = 6.6 Hz, 6H); **^{13}C NMR** (101 MHz, $CDCl_3$) δ 162.18, 160.10, 148.91, 119.49, 117.95, 38.75, 38.61, 34.50, 30.50, 30.28, 27.81, 27.16, 22.57; **HRMS** Calcd for $C_{16}H_{28}N$ $[M+H]^+$: 234.2222, Found: 234.2220.

4.4 Procedure for synthesis of 26-1 and 28-1:

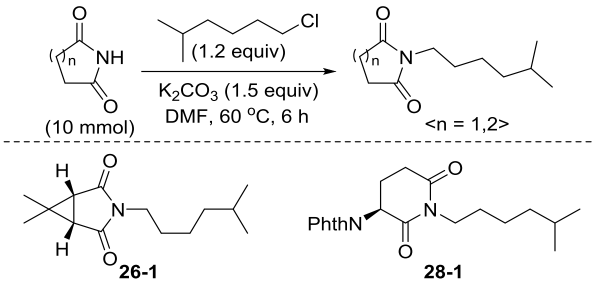

Scheme S7

The corresponding imine (10 mmol, 1.0 equiv), 1-chloro-5-methylhexane (1.62 g, 12 mmol, 1.2 equiv) and K_2CO_3 (2.07 g, 15 mmol, 1.5 equiv) were added into DMF (15 mL). The reaction mixture was heated at 60 ℃ for 6 h. After been cooled to room temperature, the reaction mixture was poured into brine (200 mL). The aqueous phase was extracted with ethyl ether (20 mL x 4). The combined organic phase was dried over Na_2SO_4 and filtrated. The filtrate was concentrated *in vacuo* and the residue was purified by flash chromatography on silica gel (ethyl acetate/petroleum ether = 10:90 (v/v)) to afford the desired product.

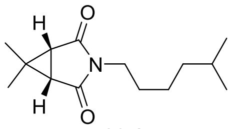

26-1

Compound **26-1** was prepared in 85% yield as a colorless oil following the general procedure. IR (KBr) : ῦ = 2955, 1768, 1703, 1403, 1177, 999, 685 cm^{-1}; **^{1}H NMR** (400 MHz, $CDCl_3$) δ 3.36 (t, J = 7.6 Hz, 2H), 2.31 (s, 3H), 1.55-1.46 (m, 3H), 1.32-1.26 (m, 2H), 1.24 (s, 3H), 1.21 (s, 3H), 1.19-1.14 (m, 2H), 0.85 (d, J = 6.6 Hz, 6H); **^{13}C NMR**

(101 MHz, $CDCl_3$) δ 173.84, 38.30, 38.16, 35.21, 33.42, 28.11, 27.72, 26.11, 24.69, 22.43, 15.55; **HRMS** Calcd for $C_{14}H_{24}NO_2$ $[M+H]^+$: 238.1807, Found: 238.1800.

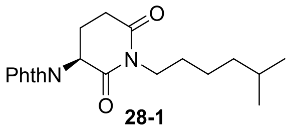

28-1

Compound **28-1** was prepared in 81% yield as a pale yellow oil following the general procedure. IR (KBr) : ῡ = 2954, 2916, 1782, 1718, 1680, 1390, 1266, 1132, 749, 615 cm^{-1}; **^{1}H NMR** (400 MHz, $CDCl_3$) δ 7.91-7.87 (m, 2H), 7.79-7.75 (m, 2H), 5.00-4.95 (m, 1H), 3.85-3.74 (m, 2H), 3.00-2.95 (m, 1H), 2.85-2.71 (m, 2H), 2.15-2.09 (m, 1H), 1.56-1.49 (m, 3H), 1.34-1.26 (m, 2H), 1.21-1.15 (m, 2H), 0.85 (d, J = 6.6 Hz, 6H); **^{13}C NMR** (101 MHz, $CDCl_3$) δ 170.79, 168.38, 167.41, 134.36, 131.76, 123.69, 50.15, 40.77, 38.42, 32.02, 27.98, 27.82, 24.63, 22.54, 22.50, 22.00; **HRMS** Calcd for $C_{20}H_{25}N_2O_4$ $[M+H]^+$: 357.1814, Found: 357.1807.

4.5 Synthesis of compound 27-1:

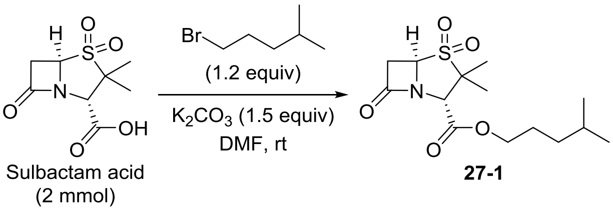

Scheme S8

Sulbactam acid (466 mg, 2.0 mmol, 1.0 equiv), 1-bromo-4-methylpentane (400 mg, 2.4 mmol, 1.2 equiv) and K_2CO_3 (415 mg, 3.0 mmol, 1.5 equiv) were added into DMF (5 mL). The reaction mixture was stirred overnight, then poured into brine (60 mL), the aqueous phase was extracted with ethyl ether (10 mL x 4). The combined organic phase was dried over Na_2SO_4 and filtrated. The filtrate was concentrated *in vacuo* and the residue was purified by flash chromatography on silica gel (ethyl acetate/petroleum ether = 10:90 (v/v)) to afford the desired product as a colorless oil (482 mg, 76%). IR (KBr) : ῡ = 3007, 1798, 1752, 1466, 1275, 1262, 751 cm^{-1}; **^{1}H NMR** (400 MHz, $CDCl_3$)

δ 4.62 (dd, J = 4.0, 2.3 Hz, 1H), 4.39 (s, 1H), 4.19 (t, J = 6.8 Hz, 2H), 3.53-3.42 (m, 2H), 1.72-1.65 (m, 2H), 1.62 (s, 3H), 1.60-1.55 (m, 1H), 1.42 (s, 3H), 1.27-1.21 (m, 2H), 0.91 (d, J = 6.6 Hz, 6H); **^{13}C NMR** (101 MHz, $CDCl_3$) δ 170.75, 166.98, 66.85, 63.18, 62.61, 61.03, 38.25, 34.81, 27.58, 26.26, 22.40, 22.37, 20.30, 18.52; **HRMS** Calcd for $C_{14}H_{23}NO_5SNa$ $[M+Na]^+$: 340.1195, Found: 340.1188.

4.6 Synthesis of compound 29-1:

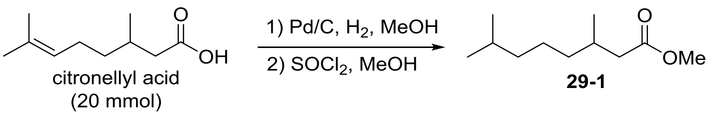

Scheme S9

To a solution of citronellyl acid (3.4 g, 20.0 mmol, 1.0 equiv) in 40 mL of MeOH was added Pd/C (0.6 g, 5% on carbon, wetted with ca. 55% water). The reaction mixture was stirred under H_2 for 24 h at room temperature. The reaction mixture was filtered through a pad of celite and the solvent was removed *in vacuo*. The residue was dissolved in MeOH (50 mL), thionyl chloride (9.5 g, 80 mmol, 4.0 equiv) was dropwisely added at 0 ^{o}C. After the addition was completed, the reaction mixture was warmed to room temperature and stirred for 2 h. The solvent was removed under reduced pressure and the resulting residue was purified by silica gel column chromatography (EtOAc/petroleum ether 1:100 (v/v)) to give compound **29-1** as a colorless oil (87% yield for 2 steps), spectra data are consistent with those reported in the literature.[5]

4.7 Synthesis of compound 30-1:

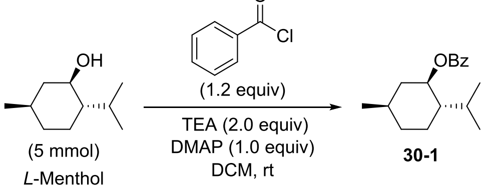

Scheme S10

Compound **30-1** was prepared following a reported procedure[9]: To a solution of DMAP (0.61 g, 5 mmol, 1.0 equiv) in CH_2C1_2 (25 mL) was added trimethylamine (1.01g, 10 mmol, 2.0 equiv), benzoyl chloride (0.84g, 6 mmol, 1.2 equiv), and finally *L*-Menthol (0.78 g, 5 mmol, 1.0 equiv). The mixture was stirred at room temperature for 2 h, and then diluted with ethyl ether (60 mL). The resulting mixture was washed with of 1N HCl (25 mL x 3), saturated aqueous $NaHCO_3$ solution (25 mL) and brine (25 mL). The organic layer was dried over Na_2SO_4 and filtrated. The filtrate was concentrated *in vacuo* and the residue was purified by flash chromatography on silica gel (ethyl acetate/petroleum ether = 5:95 (v/v)) to afford **30-1** (1.20 g, 92%) as a colorless oil. Spectra data are consistent with those reported in the literature.[8]

4.8 Procedure for synthesis of 32-1, 34 and 43-1:

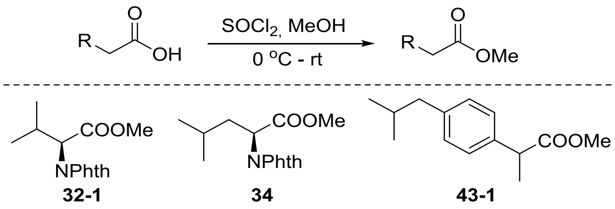

Scheme S11

To the solution of the carboxylic acid (10 mmol) in MeOH (100 mL) was added thionyl chloride (4.8 g, 40 mmol, 4.0 equiv) dropwisely at 0 °C. After the addition was completed, the reaction mixture was warmed to room temperature and stirred overnight. The solvent was removed under reduced pressure and the resulting residue was purified by silica gel column chromatography to give the desired product.

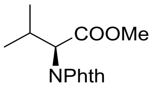

32-1

Compound **32-1** was prepared in 72% yield following the general procedure. Spectra data are consistent with those reported in the literature.[10]

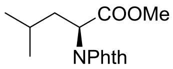

34

Compound **34** was prepared in 76% yield following the general procedure. Spectra data are consistent with those reported in the literature.[11]

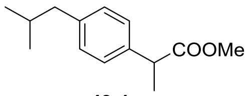

43-1

Compound **43-1** was prepared in 99% yield following the general procedure. Spectra data are consistent with those reported in the literature.[12]

4.9 Procedure for synthesis of 34 and 36-1:

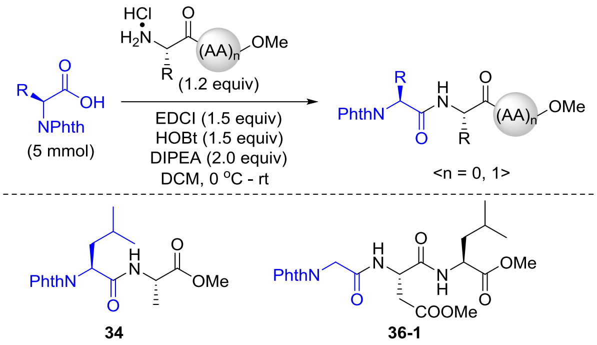

Scheme S12

To a suspension of the carboxylic acid (5 mmol) in 50 mL of DCM was added EDCI (1-ethyl-3-(3-dimethylaminopropyl)carbodiimide hydrochloride, 7.5 mmol, 1.5 equiv) and HOBt (1-hydroxybenzotriazole, 7.5 mmol, 1.5 equiv) at 0 °C. The reaction mixture was stirred for 20 min. at 0 °C. Then corresponding amine hydrochloride (6.0 mmol, 1.2 equiv) and DIPEA (10 mmol, 2.0 equiv) were added successively. The reaction mixture was warmed to room temperature and stirred overnight. Removed the solvent under reduced pressure, and the residue was dissolved in EtOAc (100 mL). The resulting solution was washed with aqueous 1M HCl (20 mL), water (20 mL), brine (20 mL), dried over Na_2SO_4 and filtrated. The solvent was removed under reduced pressure and the resulting residue was purified by silica gel column chromatography to give the desired product.

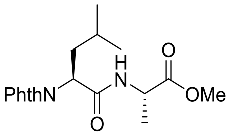

34

Compound **34** was prepared in 81% yield following the general procedure. Spectra data are consistent with those reported in the literature.[13]

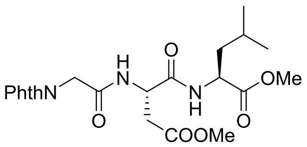

36-1

Compound **36-1** was prepared in 70% yield as a white oil following the general procedure. M.p. = 198-199 °C; IR (KBr) : ῦ = 3290, 3075, 2956, 2923, 1776, 1730, 1677, 1648, 1550, 1415, 1240, 1194, 743, 712 cm^{-1}; **^{1}H NMR** (400 MHz, $CDCl_3$) δ 7.90-7.86 (m, 2H), 7.78-7.74 (m, 2H), 7.24 (d, *J* = 7.7 Hz, 1H), 7.00 (d, *J* = 8.0 Hz, 1H), 4.87-4.83 (m, 1H), 4.54-4.49 (m, 1H), 4.39 (q, *J* = 16.2 Hz, 2H), 3.71 (s, 3H), 3.70 (s, 3H), 3.04 (dd, *J* = 17.2, 3.7 Hz, 1H), 2.66 (dd, *J* = 17.2, 7.2 Hz, 1H), 1.68-1.59 (m, 3H), 0.94 (t, *J* = 6.5 Hz, 3H); **^{13}C NMR** (101 MHz, $CDCl_3$) δ 172.65, 172.58, 169.85, 167.58, 166.21, 134.18, 131.85, 123.51, 52.16, 52.13, 51.07, 49.18, 40.62, 40.59, 35.50, 24.69, 22.70, 21.59; **HRMS** Calcd for $C_{14}H_{23}N_2NaO_6$ [M+Na^+]: 484.1696, Found: 484.1682.

4.10 Procedure for synthesis of 49-1:

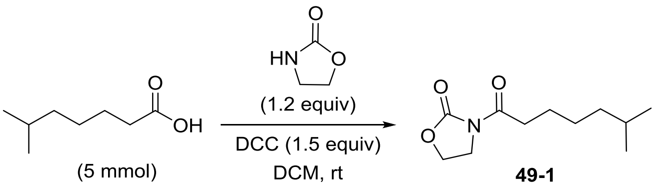

Scheme S13

To a solution of 6-methylheptanoic acid (1.44 g, 10 mmol) and oxazolidin-2-one (1.04 g, 12 mmol, 1.2 equiv) in 50 mL of DCM was added DCC (dicyclohexylcarbodiimide, 3.09 g, 15 mmol, 1.5 equiv). The reaction mixture was stirred overnight at room

temperature. The reaction mixture was washed with water (10 mL), brine (10 mL), dried over Na_2SO_4 and filtrated. The filtrate was concentrated *in vacuo* and the residue was purified by flash chromatography on silica gel (ethyl acetate/petroleum ether = 20:80 (v/v)) to afford **49-1** (1.34 g, 63%) as a white solid. M.p. = 45-46 ^oC; IR (KBr) : ῦ = 3453, 2953, 1765, 1699, 1386, 1042, 757, 701 cm^{-1}; **^{1}H NMR** (400 MHz, $CDCl_3$) δ 4.41 (t, *J* = 8.1 Hz, 2H), 4.02 (t, *J* = 8.1 Hz, 2H), 2.92 (t, *J* = 7.5 Hz, 2H), 1.68-1.59 (m, 2H), 1.57-1.49 (m, 2H), 1.39-1.32 (m, 2H), 1.23-1.17 (m, 2H), 0.87 (d, *J* = 6.6 Hz, 6H); **^{13}C NMR** (101 MHz, $CDCl_3$) δ 173.57, 153.52, 61.96, 42.49, 38.59, 35.12, 27.80, 26.89, 24.45, 22.54; **HRMS** Calcd for $C_{11}H_{19}O_3NNa$ [$M+Na^+$]: 236.1263, Found: 236.1252.

4.11 Synthesis of amide 50-1:

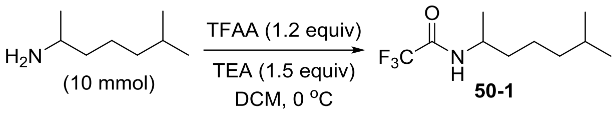

Scheme S14

To a solution of 6-methylheptan-2-amine (1.29 g, 10.0 mmol, 1.0 equiv) and TEA (1.52 g, 15.0 mmol, 1.5 equiv) in 50 mL of DCM was added a solution of TFAA (trifluoroacetic anhydride, 2.52 g, 12.0 mmol, 1.2 equiv) in DCM (10 mL) at 0 ^oC. The reaction mixture was stirred for 1 h. The reaction mixture was washed with aqueous 1M HCl (10 mL), water (10 mL), dried over Na_2SO_4 and filtrated. The solvent was removed under reduced pressure and the resulting residue was purified by flash chromatography on silica gel (eluted with EtOAc/petroleum ether 5:95 (v/v)) to give compound **50-1** as a colorless oil (1.89 g, 84% yield), spectra data are consistent with those reported in the literature.[5]

5. Optimization for tertiary C-H hydroxylation

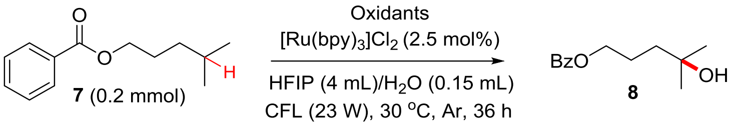

Oxidants	Equiv.	Conversion (%)[a]	Yield (%)[b]
PIDA	2.5	<5	<2
PIFA	2.5	<5	<2
BPO	2.5	22	11
$K_2S_2O_8$	2.5	<5	<2
$NaIO_4$	2.5	<5	<2

(a) Based on recovered starting material **7**. (b) Isolated yield.

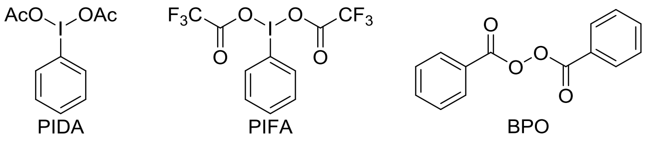

6. General procedures and substrate scope of tertiary C-H hydroxylation with PFBI-OH

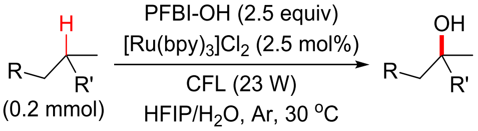

Scheme S15

General condition: To a solution of HFIP (4 mL) and water (0.15 mL) (Note: HFIP and water were bubbled with argon gas for 10 minutes to remove oxygen), were added substrate (0.2 mmol, 1.0 equiv), PFBI-OH (0.5 mmol, 2.5 equiv) and $[Ru(bpy)_3]Cl_2$ (0.005 mmol, 0.025 equiv). The reaction vial was purged with Ar for 1 min, then sealed with PTEF cap. The mixture was stirred at 30 °C under the irradiation of 23 W fluorescent light for 10-36 h. K_2CO_3 (approximate 150 mg) was added to the solution, and the resulting mixture was vigorously stirred for 5 min. The solvent was removed *in vacuo* and the residue was dissolved in DCM (5 mL). Then the mixture was filtrated through a pad of Celite and washed with DCM. The filtrate was concentrated *in vacuo* and the residue was purified by preparative thin layer chromatography or flash chromatography on silica gel to afford the desired product.

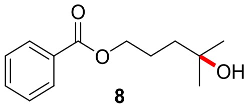

8

R_f = 0.26, 20% EtOAc in Hexane

Compound **8** was isolated in 64% yield as a colorless oil following the general procedure (36 h). **^{1}H NMR** (400 MHz, $CDCl_3$) δ 8.06-8.03 (m, 2H), 7.58-7.54 (m, 1H), 7.46-7.43 (m, 2H), 4.35 (t, *J* = 6.6 Hz, 2H), 1.91-1.84 (m, 2H), 1.67-1.60 (m, 2H), 1.27 (s, 6H). Spectra data are consistent with those reported in the literature.[5]

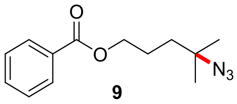

9

R_f = 0.50, 5% EtOAc in Hexane

Compound **9** was isolated in 58% yield as a colorless oil following the general procedure (36 h, 2 equiv of **11** ($BI-N_3$) was used, without water addition). IR (KBr) : ṽ = 3429, 2925, 2096, 1721, 1456, 1275, 1114, 712 cm^{-1}; **^{1}H NMR** (400 MHz, $CDCl_3$) δ 8.07-8.01 (m, 2H), 7.60-7.53 (m, 1H), 7.49-7.42 (m, 2H), 4.34 (t, *J* = 6.5 Hz, 2H), 1.90-1.82 (m, 2H), 1.66-1.62 (m, 2H), 1.31 (s, 6H); **^{13}C NMR** (101 MHz, $CDCl_3$) δ 166.53, 132.90, 130.22, 129.56, 129.51, 128.33, 64.81, 61.14, 37.80, 25.95, 23.79; **HRMS** Calcd for $C_{13}H_{17}N_3O_2Na$ [$M+Na^+$]: 270.1218, Found: 270.1212.

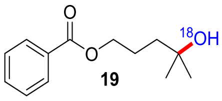

19

Compound **19** was isolated in 61% yield (88% incorporation of ^{18}O) as a colorless oil following the general procedure (Note: HFIP was dried over anhydrous Na_2SO_4 overnight, then bubbled with argon gas for 10 minutes to remove oxygen before the experiment. 36 h, 0.15 mL of $H_2{}^{18}O$ was used, 97% ^{18}O).

High Resolution Mass Spectrum (HRMS) of Compound 8:

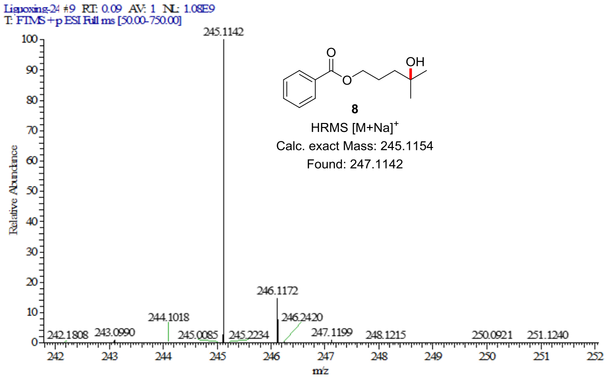

m/z	Abund (NL)
245.1154	$1.08*10^9$
247.1199	$1.05*10^7$

High Resolution Mass Spectrum (HRMS) of Compound 19:

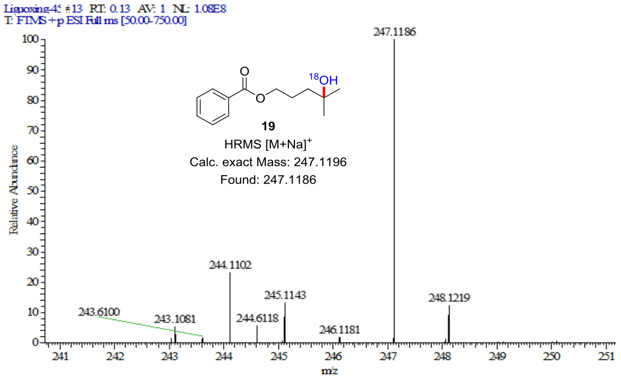

m/z	Abund (NL)
245.1143	$1.45*10^7$
247.1186	$1.08*10^8$

88% incorporation of ^{18}O was estimated based on the relative abundance (NL) of the peaks at 245.1143 and 247.1186.

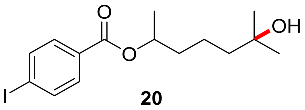

20

R_f = 0.27, 20% EtOAc in Hexane

Compound **20** was isolated in 67% yield as a colorless oil following the general procedure (24 h, conversion based on RSM **20-1**: 80%, yield based on RSM: 84%). IR (KBr) : ῡ = 3394, 2922, 1714, 1586, 1275, 1115, 1007, 754 cm^{-1}; ^{1}**H NMR** (400 MHz, $CDCl_3$) δ 7.80 (d, *J* = 8.4 Hz, 2H), 7.74 (d, *J* = 8.4 Hz, 2H), 5.20-5.13 (m, 1H), 1.80-1.72 (m, 1H), 1.66-1.44 (m, 6H), 1.34 (d, *J* = 6.3 Hz, 3H), 1.20 (s, 6H); ^{13}**C NMR** (101 MHz, $CDCl_3$) δ 165.72, 137.62, 130.96, 130.24, 100.45, 71.86, 70.80, 43.47, 36.40, 29.27, 29.19, 20.16, 20.03; **HRMS** Calcd for $C_{15}H_{21}O_3INa$ $[M+Na]^+$: 399.0433, Found: 399.0419.

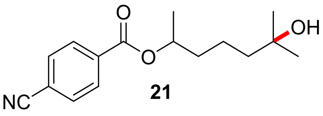

21

R_f = 0.20, 20% EtOAc in Hexane

Compound **21** was isolated in 63% yield as a colorless oil following the general procedure (24 h, conversion based on RSM **21-1**: 77%, yield based on RSM: 82%). IR (KBr) : ῡ = 3730, 2921, 2232, 1719, 1462, 1278, 1112, 863, 768, 691 cm^{-1}; **^{1}H NMR** (400 MHz, $CDCl_3$) δ 8.13 (d, *J* = 8.1 Hz, 2H), 7.74 (d, *J* = 8.1 Hz, 2H), 5.24-5.17 (m, 1H), 1.83-1.74 (m, 1H), 1.68-1.59 (m, 1H), 1.53-1.40 (m, 4H), 1.37 (d, *J* = 6.3 Hz, 3H), 1.21 (s, 6H); **^{13}C NMR** (101 MHz, $CDCl_3$) δ 164.55, 134.58, 132.20, 132.15, 130.00, 118.02, 116.15, 72.67, 70.77, 43.40, 36.34, 29.29, 29.23, 20.12, 19.98; **HRMS** Calcd for $C_{16}H_{21}O_3NNa$ $[M+Na]^+$: 298.1419, Found: 298.1407.

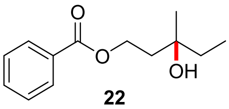

22

R_f = 0.28, 20% EtOAc in Hexane

Compound **22** was isolated in 54% yield as a colorless oil following the general procedure (36 h, conversion based on RSM **22-1**: 67%, yield based on RSM: 80%). **^{1}H NMR** (400 MHz, $CDCl_3$) δ 8.04-8.02 (m, 2H), 7.58-7.54 (m, 1H), 7.47-7.42 (m, 2H), 4.50 (t, *J* = 6.9 Hz, 2H), 1.97 (td, *J* = 6.9, 2.6 Hz, 2H), 1.59 (q, *J* = 7.5 Hz, 2H), 1.26 (s, 3H), 0.96 (t, *J* = 7.5 Hz, 3H). Spectra data are consistent with those reported in the literature.[5]

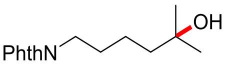

23

R_f = 0.13, 20% EtOAc in Hexane

Compound **23** was isolated in 77% yield as a white solid following the general procedure (24 h). M.p. = 54-55 °C; IR (KBr) : ῡ = 3396, 2923, 1770, 1710, 1398, 1186, 1038, 720 cm^{-1}; **^{1}H NMR** (400 MHz, $CDCl_3$) δ 7.85-7.82 (m, 2H), 7.74-7.69 (m, 2H), 3.71 (t, *J* = 7.2 Hz, 2H), 1.74-1.66 (m, 2H), 1.55-1.50 (m, 1H), 1.47-1.38 (m, 3H), 1.21 (s, 6H); **^{13}C NMR** (101 MHz, $CDCl_3$) δ 168.48, 133.87, 132.11, 123.17, 70.77, 43.13, 37.74, 29.20, 28.94, 21.40; **HRMS** Calcd for $C_{15}H_{19}O_3NNa$ $[M+Na]^+$: 284.1263, Found: 284.1250.

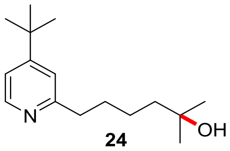

24 R_f = 0.22, 40% EtOAc in Hexane

Compound **24** was isolated in 57% yield as a colorless oil following the general procedure (24 h, conversion based on RSM **24-1**: 78%, yield based on RSM: 73%). IR (KBr) : ʋ̃ = 3358, 2925, 1724, 1632, 1467, 1210, 954, 736 cm^{-1}; **^{1}H NMR** (400 MHz, $CDCl_3$) δ 8.43 (d, *J* = 4.6 Hz, 1H), 7.14 (s, 1H), 7.12 (d, *J* = 4.6 Hz, 1H), 2.81 (t, *J* = 7.6 Hz, 2H), 1.79-1.71 (m, 2H), 1.55-1.51 (m, 2H), 1.48-1.42 (m, 2H), 1.31 (s, 9H), 1.21 (s, 6H); **^{13}C NMR** (101 MHz, $CDCl_3$) δ 161.73, 160.70, 148.61, 119.77, 118.25, 70.92, 43.52, 38.12, 34.64, 30.50, 30.42, 29.20, 23.97; **HRMS** Calcd for $C_{16}H_{28}NO$ $[M+H]^+$: 250.2171, Found: 250.2169.

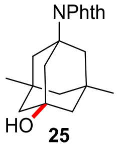

25 R_f = 0.36, 10% EtOAc in Hexane

Compound **25** was isolated e in 81% yield as a white solid following the general procedure (10 h). M.p. = 161-162 °C; IR (KBr) : ʋ̃ = 3454, 2948, 1769, 1706, 1505, 1456, 1353, 1114, 966, 720 cm^{-1}; **^{1}H NMR** (400 MHz, $CDCl_3$) δ 7.78-7.75 (m, 2H), 7.71-7.68 (m, 2H), 2.91 (s, 2H), 2.25-2.16 (m, 4H), 2.02 (s, 4H), 1.57 (br s, 1H), 1.28 (s, 2H), 1.04 (s, 6H); **^{13}C NMR** (101 MHz, $CDCl_3$) δ 169.40, 133.90, 131.67, 122.72, 85.75, 62.02, 49.07, 45.84, 44.97, 42.40, 34.50, 29.35; **HRMS** Calcd for $C_{20}H_{23}NO_3$ $[M]^+$: 325.1678, Found: 325.1675.

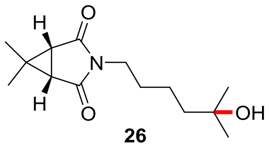

26 R_f = 0.46, 20% Acetone in Hexane

Compound **26** was isolated in 74% yield as a colorless oil following the general procedure (24 h). IR (KBr) : ʋ̃ = 3368, 2936, 1766, 1697, 1504, 1370, 1017, 751, 686 cm^{-1}; **^{1}H NMR** (400 MHz, $CDCl_3$) δ 3.39 (t, *J* = 7.5 Hz, 2H), 2.32 (s, 2H), 1.58-1.47

(m, 4H), 1.40-1.34 (m, 2H), 1.24 (s, 3H), 1.21 (s, 3H), 1.20 (s, 6H); **^{13}C NMR** (101 MHz, $CDCl_3$) δ 174.01, 70.70, 43.08, 37.93, 35.44, 33.50, 29.12, 28.29, 26.16, 21.55, 15.62; **HRMS** Calcd for $C_{14}H_{22}NO_2$ $[M-OH]^+$: 236.1651, Found: 236.1642.

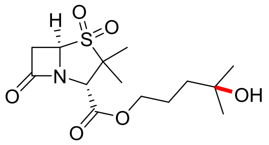

27 R_f = 0.23, 50% EtOAc in Hexane

To a solution of HFIP (4 mL) and water (0.15 mL), were added compound **27-1** (64.0 mg, 0.2 mmol, 1.0 equiv), PFBI-OH (168.8 mg, 0.5 mmol, 2.5 equiv) and $[Ru(bpy)_3]Cl_2$ (3.4 mg, 0.005 mmol, 0.025 equiv). The reaction vial was purged with Ar for 1 min and then the mixture was stirred at 30 °C under the irradiation of fluorescent light for 24 h. The solvent was removed *in vacuo* and the residue was purified by flash chromatography on silica gel (MeOH/DCM = 2:98 (v/v)) to afford compound **27** as a colorless oil (38.0 mg, 65%). IR (KBr) : ῦ = 3391, 2920, 1796, 1752, 1646, 1466, 1373, 1221, 1119, 951, 749 cm^{-1}; **^{1}H NMR** (400 MHz, $CDCl_3$) δ 4.60 (dd, *J* = 4.1, 2.3 Hz, 1H), 4.37 (s, 1H), 4.21 (t, *J* = 6.8 Hz, 2H), 3.50-3.40 (m, 2H), 1.81-1.74 (m, 2H), 1.60 (s, 3H), 1.52-1.48 (m, 2H), 1.40 (s, 3H), 1.22 (s, 6H); **^{13}C NMR** (101 MHz, $CDCl_3$) δ 170.78, 166.94, 70.36, 66.86, 63.22, 62.66, 61.07, 39.49, 38.28, 29.34, 29.30, 23.49, 20.29, 18.58; **HRMS** Calcd for $C_{14}H_{24}NO_6S$ $[M+H]^+$: 334.1324, Found: 334.1318.

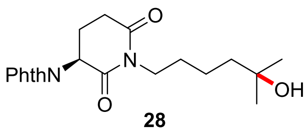

28 R_f = 0.68, 5% MeOH in DCM

Compound **28** was isolated in 62% yield as a colorless oil following the general procedure (24 h). IR (KBr) : ῦ = 3476, 2966, 1717, 1678, 1391, 1193, 1081, 890, 721 cm^{-1}; **^{1}H NMR** (400 MHz, $CDCl_3$) δ 7.90-7.88 (m, 2H), 7.78-7.76 (m, 2H), 5.01-4.97 (m, 1H), 3.84 (t, *J* = 7.3 Hz, 2H), 3.03-2.94 (m, 1H), 2.85-2.71 (m, 2H), 2.17-2.10 (m, 1H), 1.60-1.54 (m, 2H), 1.51-1.45 (m, 2H), 1.43-1.33 (m, 2H), 1.20 (s, 6H); **^{13}C NMR** (101 MHz, $CDCl_3$) δ 170.86, 168.53, 167.42, 134.41, 131.69, 123.70, 70.75, 50.11,

43.25, 40.42, 31.96, 29.16, 29.07, 28.11, 21.96, 21.34; **HRMS** Calcd for $C_{20}H_{23}N_2O_4$ $[M\text{-}OH]^+$: 355.1658, Found: 355.1651.

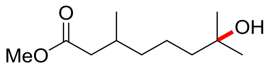
29 R_f = 0.32, 20% EtOAc in Hexane

Compound **29** was isolated in 80% yield as a colorless oil following the general procedure (24 h). **^{1}H NMR** (400 MHz, $CDCl_3$) δ 3.67 (s, 3H), 2.31 (dd, *J* = 14.7, 6.0 Hz, 1H), 2.13 (dd, *J* = 14.7, 8.0 Hz, 1H), 2.03-1.93 (m, 1H), 1.48-1.29 (m, 6H), 1.21 (s, 6H), 0.95 (d, *J* = 6.6 Hz, 3H). Spectra data are consistent with those reported in the literature.[5]

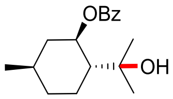
30 R_f = 0.45, 20% EtOAc in Hexane

Compound **30** was isolated in 52% yield as a colorless oil following the general procedure (24 h, conversion based on RSM **30-1**: 84%, yield based on RSM: 62%). **^{1}H NMR** (400 MHz, $CDCl_3$) δ 8.04-8.01 (m, 2H), 7.59-7.55 (m, 1H), 7.46-7.43 (m, 2H), 5.10 (td, *J* = 10.8, 4.2 Hz, 1H), 2.76 (br s, 1H), 2.19-2.14 (m, 1H), 2.00-1.94 (m, 1H), 1.90-1.83 (m, 1H), 1.78-1.70 (m, 1H), 1.67-1.55 (m, 1H), 1.19 (s, 3H), 1.17 (s, 3H), 1.14-0.96 (m, 3H), 0.94 (d, *J* = 6.5 Hz, 3H). Spectra data are consistent with those reported in the literature.[14]

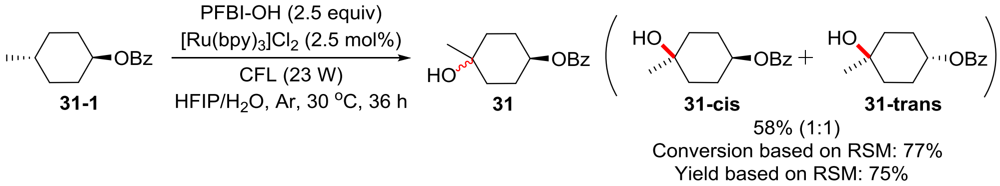

Scheme S16

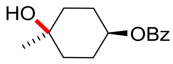
31-cis R_f = 0.2, 20% EtOAc in Hexane

Compound **31-cis** was isolated in 30% yield as a colorless oil following the general

procedure (36 h, conversion based on RSM: 77%, yield based on RSM: 39%). **^{1}H NMR** (400 MHz, $CDCl_3$) δ 8.05 (dd, *J* = 8.2, 1.0 Hz, 2H), 7.58-7.53 (m, 1H), 7.46-7.42 (m, 2H), 5.03-4.96 (m, 1H), 1.95-1.86 (m, 3H), 1.81-1.75 (m, 2H), 1.62-1.54 (m, 3H), 1.29 (s, 3H). Spectra data are consistent with those reported in the literature.[15]

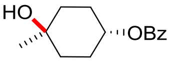

31-trans R_f = 0.3, 20% EtOAc in Hexane

Compound **31-trans** was isolated in 28% yield as a colorless oil following the general procedure (36 h, conversion based on RSM: 77%, yield based on RSM: 36%). **^{1}H NMR** (400 MHz, $CDCl_3$) δ 8.04 (d, *J* = 7.8 Hz, 2H), 7.58-7.54 (m, 1H), 7.47-7.43 (m, 2H), 5.23-5.20 (m, 1H), 2.06-2.00 (m, 2H), 1.84-1.78 (m, 4H), 1.60-1.57 (m, 2H), 1.32 (s, 3H). Spectra data are consistent with those reported in the literature.[15]

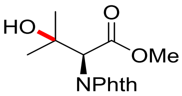

32 R_f = 0.26, 20% EtOAc in Hexane

Compound **32** was isolated in 29% yield as a colorless solid following the general procedure (36 h, conversion based on RSM: 36%, yield based on RSM: 80%). **^{1}H NMR** (400 MHz, $CDCl_3$) δ 7.92-7.90 (m, 2H), 7.80-7.78 (m, 2H), 4.90 (s, 1H), 4.40 (s, 1H), 3.76 (s, 3H), 1.53 (s, 3H), 1.30 (s, 3H). Spectra data are consistent with those reported in the literature.[16]

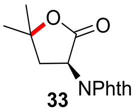

33 R_f = 0.38, 20% EtOAc in Hexane

Compound **33** was isolated in 52% yield as a white solid following the general procedure (36 h, conversion based on RSM: 59%, yield based on RSM: 88%). **^{1}H NMR** (400 MHz, $CDCl_3$) δ 7.90-7.86 (m, 2H), 7.78-7.74 (m, 2H), 5.24 (dd, *J* = 11.5, 9.6 Hz, 1H), 2.60 (t, *J* = 12.0 Hz, 1H), 2.44 (dd, *J* = 12.3, 9.6 Hz, 1H), 1.64 (s, 3H), 1.52 (s, 3H). Spectra data are consistent with those reported in the literature.[13]

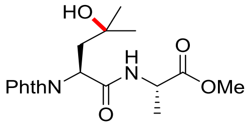

35 R$_f$ = 0.28, 60% EtOAc in Hexane

Compound **35** was isolated in 44% yield as a white solid following the general procedure (36 h, conversion based on RSM: 49%, yield based on RSM: 90%). **^{1}H NMR** (400 MHz, $CDCl_3$) δ 7.88-7.85 (m, 2H), 7.75-7.73 (m, 2H), 7.41 (d, *J* = 7.2 Hz, 1H), 5.12 (t, *J* = 5.6 Hz, 1H), 4.62-4.55 (m, 1H), 3.74 (s, 3H), 2.63 (dd, *J* = 15.3, 6.0 Hz, 1H), 2.20 (dd, *J* = 15.3, 5.3 Hz, 1H), 2.07 (br s, 1H), 1.40 (d, *J* = 7.2 Hz, 3H), 1.35 (s, 3H), 1.31 (s, 3H). Spectra data are consistent with those reported in the literature.[13]

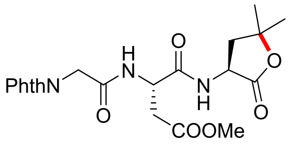

36 R$_f$ = 0.50, 5% MeOH in DCM

Compound **36** was isolated in 39% yield as a white solid following the general procedure (36 h, conversion based on RSM: 51%, yield based on RSM: 76%). M.p. = 188-190 ^oC; IR (KBr) : ῡ = 3435, 2922, 2852, 1772, 1721, 1652, 1547, 1419, 1377, 1193, 1160, 1113, 956, 712 cm^{-1}; **^{1}H NMR** (400 MHz, $CDCl_3$) δ 7.88-7.84 (m, 2H), 7.76-7.73 (m, 2H), 7.51 (d, *J* = 8.3 Hz, 1H), 7.42 (d, *J* = 7.5 Hz, 1H), 4.93-4.88 (m, 1H), 4.80-4.73 (m, 1H), 4.47-4.33 (m, 2H), 3.71 (s, 3H), 3.04 (dd, *J* = 17.1, 4.7 Hz, 1H), 2.77 (dd, *J* = 17.1, 6.0 Hz, 1H), 2.51 (dd, *J* = 12.5, 9.1 Hz, 1H), 2.14 (dd, *J* = 12.5, 11.7 Hz, 1H), 1.52 (s, 3H), 1.43 (s, 3H); **^{13}C NMR** (101 MHz, $CDCl_3$) δ 174.02, 172.52, 170.32, 167.77, 166.66, 134.27, 131.90, 123.61, 82.42, 52.26, 50.35, 49.40, 40.80, 35.15, 28.81, 27.16; **HRMS** Calcd for $C_{22}H_{27}O_8N_2Na$ [M+Na$^+$]: 468.1383, Found: 468.1367.

7. General procedures and substrate scope of benzylic C-H hydroxylation with BI-OH

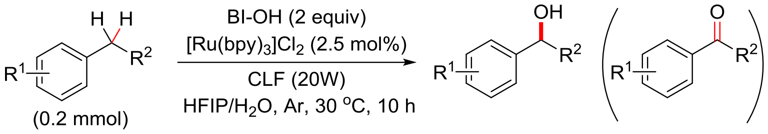

Scheme S17

General condition: To a solution of HFIP (4 mL) and water (0.45 mL) (Note: HFIP and water were bubbled with argon gas for 10 minutes to remove oxygen), were added substrate (0.2 mmol, 1.0 equiv), BI-OH (0.4 mmol, 2.0 equiv) and $[Ru(bpy)_3]Cl_2$ (0.005 mmol, 0.025 equiv). The reaction vial was purged with Ar for 1 min, then sealed with PTEF cap. The reaction mixture was stirred at 30 °C under the irradiation of fluorescent light for 10 h. K_2CO_3 (approximate 20 mg) was added to the solution, and the resulting mixture was vigorously stirred for 5 min. The solvent was removed *in vacuo* and the residue was dissolved in DCM (5 mL). Then the mixture was filtrated through a pad of Celite and washed with DCM. The filtrate was concentrated *in vacuo* and the residue was purified by preparative thin layer chromatography or flash chromatography on silica gel to afford the desired product.

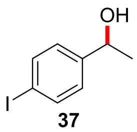

37 R_f = 0.44, 20% EtOAc in Hexane

Compound **37** was isolated in 71% yield as a colorless oil following the general procedure. **^{1}H NMR** (400 MHz, $CDCl_3$) δ 7.71-7.63 (m, 2H), 7.13-7.11 (m, 2H), 4.85 (q, J = 6.5 Hz, 1H), 1.84 (br s, 1H), 1.46 (d, J = 6.5 Hz, 3H). Spectra data are consistent with those reported in the literature.[17]

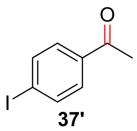

37' R_f = 0.44, 20% EtOAc in Hexane

Compound **37′** was isolated in 8% yield as a light yellow solid following the general procedure. **^{1}H NMR** (400 MHz, $CDCl_3$) δ7.85-7.82 (m, 2H), 7.69-7.65 (m, 2H), 2.58 (s, 3H). Spectra data are consistent with those reported in the literature.[18]

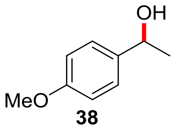

38 R_f = 0.32, 20% EtOAc in Hexane

Compound **38** was isolated in 82% yield as colorless oil following the general procedure (1.5 equiv. BI-OH was used). **^{1}H NMR** (400 MHz, $CDCl_3$) δ 7.32-7.28 (m, 2H), 6.90-6.86 (m, 2H), 4.85 (q, J = 6.4 Hz, 1H), 3.80 (s, 3H), 1.86 (br s, 1H), 1.48 (d, J = 6.4 Hz, 3H). Spectra data are consistent with those reported in the literature.[19]

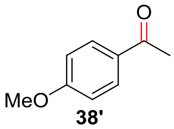

38' R_f = 0.32, 20% EtOAc in Hexane

Compound **38′** was isolated in 10% yield as a white solid following the general procedure (1.5 equiv. BI-OH was used). **^{1}H NMR** (400 MHz, $CDCl_3$) δ 7.96-7.93 (m, 2H), 6.96-6.92 (m, 2H), 3.88 (s, 3H), 2.57 (s, 3H). Spectra data are consistent with those reported in the literature.[20]

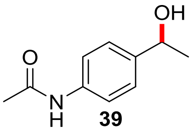

39 R_f = 0.17, 60% EtOAc in Hexane

Compound **39** was isolated in 76% yield as a colorless oil following the general procedure (16 h, 4.0 equiv. BI-OH was used). **^{1}H NMR** (400 MHz, $CDCl_3$) δ 7.49 (br s, 1H), 7.45 (d, J = 8.4 Hz, 2H), 7.30 (d, J = 8.4 Hz, 2H), 4.86 (q, J = 6.2 Hz, 1H), 2.15 (s, 3H), 2.10 (br s, 1H), 1.47 (d, J = 6.4 Hz, 3H). Spectra data are consistent with those reported in the literature.[21]

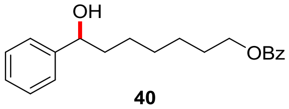

40 R_f = 0.43, 20% EtOAc in Hexane

Compound **40** was isolated in 78% yield as a colorless oil following the general procedure. IR (KBr) : ῦ = 3365, 2929, 1717, 1452, 1315, 1274, 1116, 742, 709 cm^{-1};**^{1}H NMR** (400 MHz, $CDCl_3$) δ 8.05-8.02 (m, 2H), 7.57-7.53 (m, 1H), 7.46-7.41 (m, 2H), 7.37-7.32 (m, 4H), 7.30-7.25 (m, 1H), 4.67 (dd, *J* = 7.1, 6.1 Hz, 1H), 4.29 (t, *J* = 6.6 Hz, 2H), 1.89 (br s, 1H), 1.86-1.67 (m, 4H), 1.49-1.27 (m, 6H); **^{13}C NMR** (101 MHz, $CDCl_3$) δ 166.63, 144.82, 132.74, 130.36, 129.45, 128.34, 128.24, 127.39, 125.80, 74.46, 64.96, 38.90, 29.07, 28.55, 25.87, 25.62; **HRMS** Calcd for $C_{20}H_{24}O_3Na$ $[M+Na]^+$: 335.1623, Found: 335.1609.

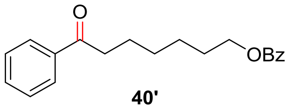

40' R_f = 0.77, 20% EtOAc in Hexane

Compound **40′** was isolated in 6% yield as a colorless oil following the general procedure. IR (KBr) : ῦ = 3416, 3062, 2932, 2857, 1967, 1912, 1718, 1685, 1451, 1276, 1116, 971, 804, 714cm^{-1}; **^{1}H NMR** (400 MHz, $CDCl_3$) δ 8.04 (dd, *J* = 8.0, 0.9 Hz, 2H), 7.96 (dd, *J* = 8.0, 0.9 Hz, 2H), 7.58-7.54 (m, 2H), 7.48-7.42 (m, 4H), 4.33 (t, *J* = 6.6 Hz, 2H), 2.99 (t, *J* = 7.3 Hz, 2H), 1.83-1.75 (m, 4H), 1.55-1.43 (m, 4H); **^{13}C NMR** (101 MHz, $CDCl_3$) δ 200.32, 166.66, 137.02, 132.91, 132.81, 130.45, 129.52, 128.56, 128.31, 128.02, 64.94, 38.42, 28.98, 28.58, 25.94, 24.13; **HRMS** Calcd for $C_{20}H_{22}O_3Na$ $[M+Na]^+$: 333.1467, Found: 333.1453.

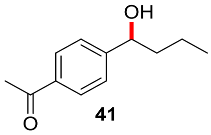

41 R_f = 0.19, 20% EtOAc in Hexane

Compound **41** was isolated in 57% yield as a pale yellow oil following the general procedure (16 h, 4.0 equiv. BI-OH was used). IR (KBr) : ῦ = 3440, 2958, 1931, 1679, 1607, 1414, 1360, 1271, 960, 836, 750 cm^{-1}; **^{1}H NMR** (400 MHz, $CDCl_3$) δ 7.94 (d, *J* = 8.2 Hz, 2H), 7.44 (d, *J* = 8.2 Hz, 2H), 4.76 (dd, *J* = 7.3, 5.8 Hz, 1H), 2.60 (s, 3H), 2.04 (br s, 1H), 1.82-1.65 (m, 2H), 1.49-1.28 (m, 2H), 0.94 (t, *J* = 7.4 Hz, 3H); **^{13}C**

NMR (101 MHz, $CDCl_3$) δ 197.85, 150.29, 136.28, 128.53, 125.95, 73.83, 41.27, 26.61, 18.83, 13.88; **HRMS** Calcd for $C_{12}H_{15}O_2$ $[M-H]^-$: 191.1072, Found: 191.1068.

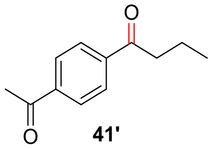

41'

R_f = 0.58, 20% EtOAc in Hexane

Compound **41′** was isolated in 9% yield as a white solid following the general procedure (16 h, 4.0 equiv. BI-OH was used). **^{1}H NMR** (400 MHz, $CDCl_3$) δ 8.04 (br s, 4H), 2.99 (t, J = 7.2 Hz, 2H), 2.65 (s, 3H), 1.83-1.74 (m, 2H), 1.02 (t, J = 7.4 Hz, 3H). Spectra data are consistent with those reported in the literature.[22]

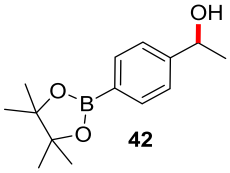

42

R_f = 0.38, 20% EtOAc in Hexane

Compound **42** was isolated in 50% yield as a colorless oil following the general procedure (4.0 equiv. BI-OH was used). **^{1}H NMR** (400 MHz, $CDCl_3$) δ 7.80 (d, J = 7.9 Hz, 2H), 7.38 (d, J = 7.9 Hz, 2H), 4.92 (q, J = 6.3 Hz, 1H), 1.82 (br s, 1H), 1.49 (d, J = 6.5 Hz, 3H), 1.34 (s, 12H). Spectra data are consistent with those reported in the literature.[23]

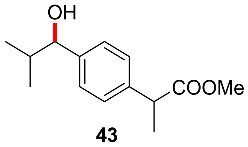

43

R_f = 0.36, 20% EtOAc in Hexane

Compound **43** was isolated in 64% yield as a colorless oil following the general procedure. **^{1}H NMR** (400 MHz, $CDCl_3$) δ 7.27 (s, 4H), 4.34 (d, J = 6.9 Hz, 1H), 3.72 (q, J = 7.2 Hz, 1H), 3.66 (s, 3H), 1.99-1.90 (m, 1H), 1.49 (d, J = 7.2 Hz, 3H), 1.00 (d, J = 6.7 Hz, 3H), 0.80 (d, J = 6.8 Hz, 3H). Spectra data are consistent with those reported in the literature.[24]

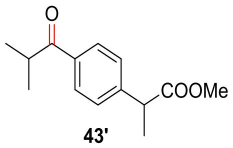

43'

R_f = 0.4, DCM

Compound **43′** was isolated in 10% yield as a colorless oil following the general procedure. **^{1}H NMR** (400 MHz, $CDCl_3$) δ 7.93 (d, *J* = 8.3 Hz, 2H), 7.39 (d, *J* = 8.3 Hz, 2H), 3.79 (q, *J* = 7.2 Hz, 1H), 3.68 (s, 3H), 3.59-3.48 (m, 1H), 1.53 (d, *J* = 7.2 Hz, 3H), 1.21 (d, *J* = 6.8 Hz, 6H). Spectra data are consistent with those reported in the literature.[25]

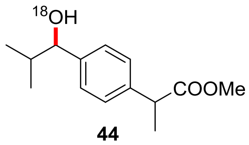

44

Compound **44** was isolated in 60% yield (93% incorporation of ^{18}O) as a colorless oil following the general procedure (Note: HFIP was dried over anhydrous Na_2SO_4 overnight, then bubbled with argon gas for 10 minutes to remove oxygen before the experiment. 10 h, 0.45 mL of $H_2{}^{18}O$ was used, 97% ^{18}O).

High Resolution Mass Spectrum (HRMS) of Compound 43:

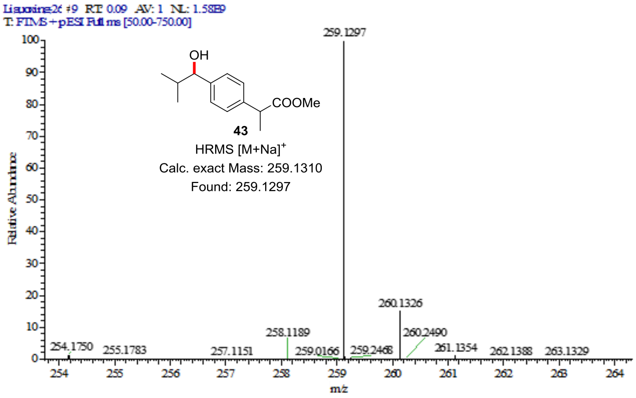

m/z	Abund (NL)
259.1297	$1.58*10^9$
261.1354	$1.85*10^7$

High Resolution Mass Spectrum (HRMS) of Compound 44:

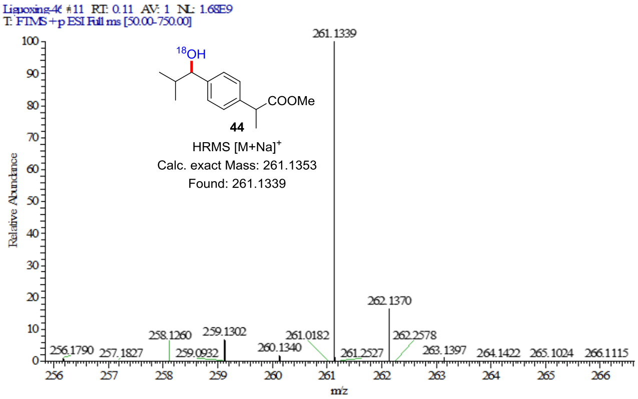

m/z	Abund (NL)
259.1302	$1.18*10^8$
261.1339	$1.68*10^9$

93% incorporation of ^{18}O was estimated based on the relative abundance (NL) of the peaks at 259.1302 and 261.1339.

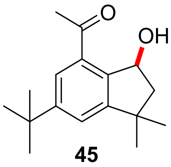

45 R_f = 0.63, 20% EtOAc in Hexane

Compound **45** was isolated in 90% yield as a white solid following the general procedure (1.5 equiv. BI-OH was used). M.p. = 59-60 °C; IR (KBr) : ῦ = 3494, 2954, 2865, 1792, 1664, 1460, 1362, 1238, 1060, 889, 778, 704 cm^{-1}; **^{1}H NMR** (400 MHz, $CDCl_3$) δ 7.78 (d, *J* = 1.6 Hz, 1H), 7.43 (d, *J* = 1.6 Hz, 1H), 5.38 (ddd, *J* = 7.6, 3.9, 1.6 Hz, 1H), 4.53 (d, *J* = 1.6 Hz, 1H), 2.68 (s, 3H), 2.30 (dd, *J* = 13.5, 7.6 Hz, 1H), 2.05 (dd, *J* = 13.6, 3.9 Hz, 1H), 1.40 (s, 3H), 1.37 (s, 9H), 1.26 (s, 3H); **^{13}C NMR** (101 MHz, $CDCl_3$) δ 202.70, 154.34, 152.22, 142.21, 133.62, 126.21, 124.63, 73.19, 48.71, 42.79, 34.88, 31.40, 30.92, 29.88, 27.96; **HRMS** Calcd for $C_{17}H_{24}NaO_2$ [M+Na]$^+$: 283.1674,

Found: 283.1660.

8. Scale-up reaction for hydroxylation

8.1 Scale-up reaction for hydroxylation of 28-1:

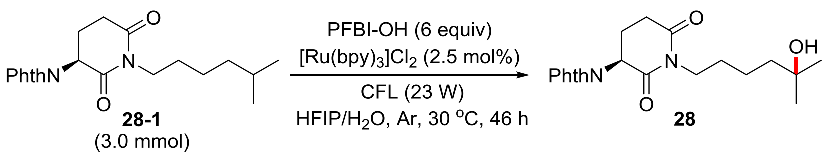

Scheme S18

Reaction condition: To a solution of HFIP (60 mL) and water (2.25 mL) (Note: HFIP and water were bubbled with argon gas for 10 minutes to remove oxygen), were added compound **28-1** (1.07 g, 3.0 mmol, 1.0 equiv), PFBI-OH (2.54 g, 7.5 mmol, 2.5 equiv) and $[Ru(bpy)_3]Cl_2$ (48.0 mg, 0.075 mmol, 0.025 equiv). The reaction vial was purged with Ar for 5 min, sealed with PTEF cap. The reaction mixture was stirred at 30 ^{o}C under the irradiation of fluorescent light for 46 h. K_2CO_3 (1.05 g) was added to the solution, and the resulting mixture was vigorously stirred for 10 min. The solvent was removed *in vacuo* and the residue was dissolved in MeOH (50 mL). Then silica gel (8.0 g) was added into the solution. The solvent was removed *in vacuo* and the residue was purified by flash chromatography on silica gel (ethyl acetate/petroleum ether = 50:50 (v/v)) to afford compound **28** as a colorless oil (667 mg, 60%). 119 mg of starting material **28-1** was recovered. Conversion based on RSM: 89%. Yield based on RSM: 67%.

8.2 Scale-up reaction for hydroxylation of 43-1:

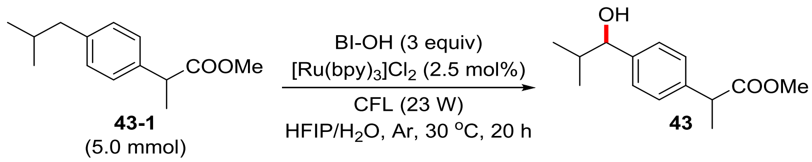

Scheme S19

Reaction condition: To a solution of HFIP (100 mL) and water (11.25 mL) (Note: HFIP and water were bubbled with argon gas for 10 minutes to remove oxygen), were added compound **43-1** (1.10 g, 5.0 mmol, 1.0 equiv), BI-OH (2.66 g, 10.0 mmol, 2.0 equiv) and $[Ru(bpy)_3]Cl_2$ (80.0 mg, 0.125 mmol, 0.025 equiv). The reaction vial was purged with Ar for 5 min, sealed with PTEF cap. The reaction mixture was stirred at 30 ^{o}C under the irradiation of fluorescent light for 20 h. K_2CO_3 (3.75 g) was added to the solution, and the resulting mixture was vigorously stirred for 10 min. The solvent was removed *in vacuo* and the residue was dissolved in MeOH (50 mL). Then silica gel (10.0 g) was added into the solution. The solvent was removed *in vacuo* and the residue was purified by flash chromatography on silica gel (ethyl acetate/petroleum ether = 15:85 (v/v)) to afford compound **43** as a colorless oil (680 mg, 58%). 137 mg of starting material **43-1** was recovered. Conversion based on RSM: 88%. Yield based on RSM: 66%.

9. General procedures and substrate scope of C(sp^3)-H amidation with PFBI-OH

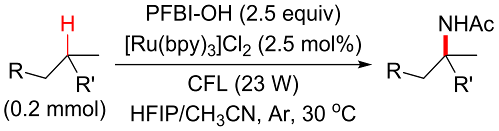

Scheme S20

General condition: To a solution of HFIP (4 mL) and acetonitrile (3 mL) (Note: HFIP and acetonitrile were dried over 4 Å molecular sieves overnight, then bubbled with argon gas for 10 minutes to remove oxygen before the experiment), were added substrate (0.2 mmol, 1.0 equiv), PFBI-OH (0.5 mmol, 2.5 equiv) and $[Ru(bpy)_3]Cl_2$ (0.005 mmol, 0.025 equiv). The reaction vial was purged with Ar for 1 min, then sealed with PTEF cap. The mixture was stirred at 30 ^{o}C under the irradiation of 23 W fluorescent light for 10-24 h. K_2CO_3 (approximate 150 mg) was added to the solution, and the resulting mixture was vigorously stirred for 5 min. The solvent was removed *in*

vacuo and the residue was dissolved in DCM (5 mL). Then the mixture was filtrated through a pad of Celite and washed with DCM. The filtrate was concentrated *in vacuo* and the residue was purified by preparative thin layer chromatography or flash chromatography on silica gel to afford the desired product.

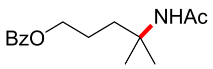

10 R_f = 0.38, 50% EtOAc in Hexane

Compound **10** was isolated in 56% yield as a white solid following the general procedure (36 h). **^{1}H NMR** (400 MHz, $CDCl_3$) δ 8.04 (dd, *J* = 8.3, 1.2 Hz, 2H), 7.61 – 7.53 (m, 1H), 7.46-7.43 (m, 2H), 5.30 (br s, 1H), 4.32 (t, *J* = 6.6 Hz, 2H), 1.94 (s, 3H), 1.91-1.86 (m, 2H), 1.78-1.72 (m, 2H), 1.33 (s, 6H). Spectra data are consistent with those reported in the literature.[26]

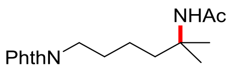

46 R_f = 0.20, 40% EtOAc in Hexane

Compound **46** was isolated in 67% yield as a white solid following the general procedure (24 h). M.p. = 142-143 °C; IR (KBr) : ῦ = 3303, 2928, 1771, 1712, 1657, 1545, 1439, 1397, 1369, 1041, 720 cm^{-1}; **^{1}H NMR** (400 MHz, $CDCl_3$) δ 7.86-7.83 (m, 2H), 7.74-7.71 (m, 2H), 5.42 (br s, 1H), 3.70 (t, *J* = 7.0 Hz, 2H), 1.94 (s, 3H), 1.73-1.66 (m, 4H), 1.35-1.26 (m, 2H), 1.31 (s, 6H), 0.96 (s, 6H); **^{13}C NMR** (101 MHz, $CDCl_3$) δ 169.57, 168.58, 133.94, 132.04, 123.18, 77.32, 77.00, 76.68, 53.41, 39.75, 37.44, 28.65, 26.73, 24.42, 21.01; **HRMS** Calcd for $C_{17}H_{22}N_2O_3Na$ [M+Na$^+$]: 325.1528, Found: 325.1522.

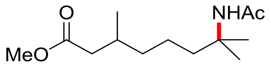

47 R_f = 0.22, 20% Acetone in Hexane

Compound **47** was isolated in 71% yield as a colorless oil following the general procedure (24 h). M.p. = 142-143 °C; IR (KBr) : ῦ = 3306, 3080, 2925, 1737, 1656, 1548, 1460, 1372, 1192 cm^{-1}; **^{1}H NMR** (400 MHz, $CDCl_3$) δ 5.19 (br s, 1H), 3.67 (s,

3H), 2.30 (dd, *J* = 14.7, 6.1 Hz, 1H), 2.12 (dd, *J* = 14.7, 8.0 Hz, 1H), 1.98-1.93 (m, 1H), 1.92 (s, 3H), 1.70-1.63 (m, 2H), 1.31-1.17 (m, 4H), 1.29 (s, 6H), 0.93 (d, *J* = 6.6 Hz, 3H); **^{13}C NMR** (101 MHz, $CDCl_3$) δ 173.74, 169.36, 53.66, 51.38, 41.62, 40.12, 36.91, 30.28, 26.95, 26.89, 24.51, 21.46, 19.74; **HRMS** Calcd for $C_{13}H_{25}NO_3Na$ [M+Na$^+$]: 266.1732, Found: 266.1725.

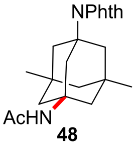

48 R_f = 0.19, 20% Acetone in Hexane

Compound **48** was isolated in 60% yield as a white solid following the general procedure (10 h). M.p. = 186-187 °C; IR (KBr) : ῦ = 3304, 3068, 2922, 1770, 1708, 1656, 1548, 1348, 1316, 720 cm^{-1}; **^{1}H NMR** (400 MHz, $CDCl_3$) δ 7.77-7.73 (m, 2H), 7.70-7.66 (m, 2H), 5.28 (br s, 1H), 2.61 (s, 2H), 2.14 (s, 4H), 1.92 (s, 3H), 1.78-1.70 (m, 4H), 1.22 (s, 2H), 0.96 (s, 6H); **^{13}C NMR** (101 MHz, $CDCl_3$) δ 169.56, 169.37, 133.79, 131.77, 122.60, 61.73, 54.29, 49.23, 46.27, 45.14, 42.59, 33.38, 29.53, 24.60; **HRMS** Calcd for $C_{22}H_{26}N_2O_3Na$ [M+Na$^+$]: 389.1841, Found: 389.1834.

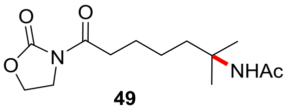

49 R_f = 0.32, EtOAc

Compound **49** was isolated in 71% yield as a white solid following the general procedure (24 h). M.p. = 86-87 °C; IR (KBr) : ῦ = 3386, 3081, 2923, 1778, 1698, 1656, 1545, 1386, 1221, 1037, 761 cm^{-1}; **^{1}H NMR** (400 MHz, $CDCl_3$) δ 5.19 (br s, 1H), 4.42 (t, *J* = 8.1 Hz, 2H), 4.02 (t, *J* = 8.1 Hz, 1H), 2.93 (t, *J* = 7.4 Hz, 1H), 1.92 (s, 3H), 1.74-1.62 (m, 4H), 1.37-1.25 (m, 2H), 1.30 (s, 6H); **^{13}C NMR** (101 MHz, $CDCl_3$) δ 173.28, 169.43, 153.53, 61.98, 53.57, 42.46, 39.72, 34.95, 26.88, 24.45, 23.61; **HRMS** Calcd for $C_{13}H_{22}N_2O_4Na$ [M+Na$^+$]: 293.1477, Found: 293.1469.

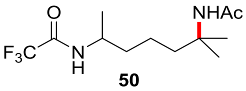

50 R_f = 0.54, 40% Acetone in Hexane

Compound **50** was isolated in 64% yield as a colorless oil following the general

procedure (24 h). IR (KBr) : ῦ = 3363, 3082, 2924, 1706, 1658, 1552, 1460, 1373, 1188, 1159 cm^{-1}; **^{1}H NMR** (400 MHz, $CDCl_3$) δ 6.28 (d, *J* = 6.9 Hz, 1H), 5.34 (br s, 1H), 4.11-4.00 (m, 1H), 1.93 (s, 3H), 1.76-1.69 (m, 2H), 1.54-1.43 (m, 2H), 1.33-1.20 (m, 2H), 1.29 (s, 3H), 1.28 (s, 3H), 1.23 (d, *J* = 6.6 Hz, 3H); **^{13}C NMR** (101 MHz, $CDCl_3$) δ 170.07, 156.85 (q, *J* = 36.5 Hz), 115.93 (q, *J* = 287.9 Hz), 53.59, 46.18, 39.31, 36.52, 27.28, 26.67, 24.10, 20.59, 20.42; **HRMS** Calcd for $C_{12}H_{21}F_3N_2O_2Na$ [M+Na$^+$]: 305.1453, Found: 305.1447.

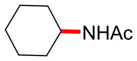

51 R_f = 0.21, 20% Acetone in Hexane

Compound **51** was isolated in 63% yield as a white solid following the general procedure (24 h, 0.5 mmol scale, 1.0 equiv of cyclohexane was used). **^{1}H NMR** (400 MHz, $CDCl_3$) δ 5.50 (br s, 1H), 3.79-3.70 (m, 1H), 1.98 (s, 3H), 1.93-1.89 (m, 2H), 1.73-1.68 (m, 2H), 1.64-1.60 (m, 1H), 1.41-1.30 (m, 2H), 1.20-1.06 (m, 3H). Spectra data are consistent with those reported in the literature.[27]

10. General procedures and substrate scope of benzylic C-H amidation with BI-OH

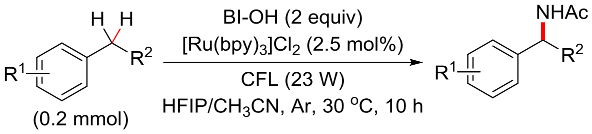

Scheme S21

General condition: To a solution of HFIP (4 mL) and acetonitrile (1.5 mL) (Note: HFIP and acetonitrile were dried over 4 Å molecular sieves overnight, then bubbled with argon gas for 10 minutes to remove oxygen before the experiment), were added substrate (0.2 mmol, 1.0 equiv), BI-OH (0.4 mmol, 2.0 equiv) and $[Ru(bpy)_3]Cl_2$ (0.005 mmol, 0.025 equiv). The reaction vial was purged with Ar for 1 min, then sealed with PTEF cap. The reaction mixture was stirred at 30 °C under the irradiation of fluorescent

light for 10 h. K_2CO_3 (approximate 20 mg) was added to the solution, and the resulting mixture was vigorously stirred for 5 min. The solvent was removed *in vacuo* and the residue was dissolved in DCM (5 mL). Then the mixture was filtrated through a pad of Celite and washed with DCM. The filtrate was concentrated *in vacuo* and the residue was purified by preparative thin layer chromatography or flash chromatography on silica gel to afford the desired product.

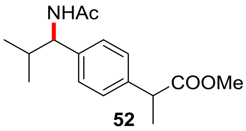

52

Compound **52** was isolated in 67% yield as a colorless oil following the general procedure. IR (KBr) : ῦ = 3292, 2926, 1737, 1646, 1546, 1460, 1375, 1210, 1166, 856 cm^{-1}; **^{1}H NMR** (400 MHz, $CDCl_3$) δ 7.24 (d, *J* = 8.1 Hz, 1H), 7.18 (d, *J* = 8.1 Hz, 1H), 5.94 (d, *J* = 8.9 Hz, 1H), 4.74 (dd, *J* = 8.9, 8.3 Hz, 1H), 3.71 (q, *J* = 7.2 Hz, 1H), 3.66 (s, 3H), 2.05-1.96 (m, 1H), 1.98 (s, 3H), 1.48 (d, *J* = 7.2 Hz, 3H), 0.96 (d, *J* = 6.7 Hz, 3H), 0.82 (d, *J* = 6.7 Hz, 3H); **^{13}C NMR** (101 MHz, $CDCl_3$) δ 174.99, 174.95, 169.29, 140.47, 139.18, 127.49, 127.18, 58.75, 51.99, 44.98, 44.96, 33.27, 23.43, 19.75, 18.78, 18.53, 18.50; **HRMS** Calcd for $C_{16}H_{23}NO_3Na$ [M+Na^+]: 300.1576, Found: 300.1568.

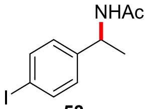

53

Compound **53** was isolated in 74% yield as a pale yellow solid following the general procedure. M.p. = 103-104 °C; IR (KBr) : ῦ = 3426, 3064, 2925, 1647, 1545, 1374, 1274, 821, 754 cm^{-1}; **^{1}H NMR** (400 MHz, $CDCl_3$) δ 7.65 (d, *J* = 8.3 Hz, 2H), 7.06 (d, *J* = 8.3 Hz, 2H), 5.85 (d, *J* = 5.4 Hz, 1H), 5.09-5.01 (m, 1H), 1.99 (s, 3H), 1.45 (d, *J* = 6.9 Hz, 3H), ; **^{13}C NMR** (101 MHz, $CDCl_3$) δ 169.48, 142.80, 137.67, 128.16, 92.68, 48.43, 23.29, 21.55; **HRMS** Calcd for $C_{10}H_{13}INO$ [M+H^+]: 290.0042, Found: 290.0033.

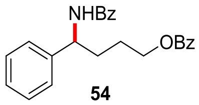

54

Compound **54** was isolated in 62% yield as a white solid following the general procedure (2.5 mL PhCN was used). M.p. = 146-147 °C; IR (KBr) : ṽ = 3315, 3062, 2924, 1717, 1637, 1533, 1276, 1116, 708 cm^{-1}; **^{1}H NMR** (400 MHz, $CDCl_3$) δ 8.02 (dd, *J* = 8.3, 1.2 Hz, 2H), 7.77 (dd, *J* = 8.4, 1.2 Hz, 2H), 7.58-7.52 (m, 1H), 7.51-7.47 (m, 1H), 7.45-7.34 (m, 8H), 7.31-7.27 (m, 1H), 6.40 (d, *J* = 8.0 Hz, 1H), 5.29-5.23 (m, 1H), 4.35 (td, *J* = 6.4, 0.9 Hz, 2H), 2.17-2.01 (m, 2H), 1.96-1.85 (m, 1H), 1.85-1.74 (m, 1H); **^{13}C NMR** (101 MHz, $CDCl_3$) δ 166.77, 166.55, 141.74, 134.43, 132.88, 131.47, 130.19, 129.51, 128.82, 128.50, 128.31, 127.62, 126.92, 126.62, 64.48, 53.66, 32.55, 25.85; **HRMS** Calcd for $C_{24}H_{23}NO_3Na$ [M+Na^+]: 396.1576, Found: 396.1566.

11. Measurement of quantum yield (Φ) for C-H hydroxylation of 7[28]

11. 1 Determination of light intensity

11. 1. 1 Determination of the light intensity at 436 nm:

A 0.15 M solution of ferrioxalate was prepared by dissolving 2.3848 g of potassium ferrioxalate hydrate ($K_3Fe(C_2O_4)_3 \cdot 3H_2O$) in 30.00 mL of 0.05 M H_2SO_4. A buffer of phenanthroline was prepared by dissolving 50.0 mg of phenanthroline and 11.25 g of sodium acetate in 50.00 mL of 0.5 M H_2SO_4. The above solutions were both stored in the dark after preparation. To determine the photon flux of the spectrophotometer, 2.0 mL of the ferrioxalate solution was placed in a cuvette and irradiated for 90.0 seconds at λ = 436 nm with an emission slit width at 10.0 nm. After irradiation completed, 0.35 mL of the phenanthroline solution was added to the cuvette. Then the solution was allowed to rest in dark for 1 h. The absorbance of the solution was measured at 510 nm. A non-irradiated sample was also prepared and the absorbance at 510 nm measured. Conversion was calculated using equation 1:

$$\text{mol } Fe^{2+} = \frac{V \bullet \Delta A}{l \bullet \varepsilon} \quad (\textbf{Equation 1})$$

In the equation 1, V is the total volume (0.00235 L) of the solution after addition of phenanthroline, ΔA is the difference in absorbance at 510 nm between the irradiated and non-irradiated solutions, l is the path length (1.000 cm), and ε is the molar absorptivity at 510 nm (11,100 L mol^{-1} cm^{-1}). The photon flux can be calculated using equation 2:

$$\text{Photon flux} = \frac{\text{mol Fe}^{2+}}{\Phi \bullet t \bullet f} \quad \textbf{(Equation 2)}$$

In the equation 2, Φ is the quantum yield for the ferrioxalate actinometer (1.01 for a 0.15 M solution at λ = 436 nm), t is the time (90.0 s), and f is the fraction of light absorbed at λ = 436 nm (0.99990, *vide infra*). The photon flux was calculated (average of three experiments) to be 4.29 $\times 10^{-10}$ einstein s^{-1}.

Sample calculation:

$$\text{mol Fe}^{2+} = \frac{V \bullet \Delta A}{l \bullet \varepsilon} = \frac{0.00235\,\text{L} \bullet 0.184}{1\,\text{cm} \bullet 11100\,\text{L mol}^{-1}\,\text{cm}^{-1}} = 3.90 \text{ x } 10^{-8}\,\text{mol}$$

$$\text{Photon flux} = \frac{\text{mol Fe}^{2+}}{\Phi \bullet t \bullet f} = \frac{3.90 \text{ x } 10^{-8}}{1.01 \bullet 90\,\text{s} \bullet 0.99990} = 4.29 \text{ x } 10^{-10} \text{ einstein s}^{-1}$$

11. 1. 2 Determination of fraction of light absorbed at 436 nm for the ferrioxalate solution:

The absorbance of the above ferrioxalate solution at 436 nm was measured to be 4.009. The fraction of light absorbed (f) was calculated using equation 3, where A (absorbance) is the measured absorbance at 436 nm.

$$f = 1 - 10^{-A} \quad \textbf{(Equation 3)}$$

$$f = 1 - 10^{-A} = 1\text{-}10^{-4.009} = 0.99990$$

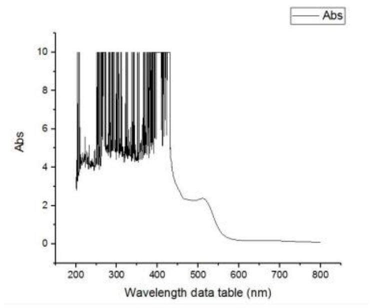

Figure S1. Absorbance of the ferrioxalate actinometer solution.

11. 2 Determination of quantum yield:

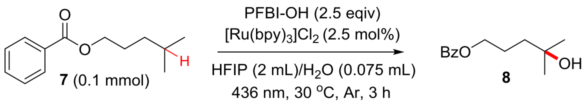

Scheme S22

A cuvette was charged with compound **7** (0.1 mmol, 1.0 equiv), PFBI-OH (0.25 mmol, 2.5 equiv) and $[Ru(bpy)_3]Cl_2$ (0.0025 mmol, 0.025 equiv). HFIP (2 mL) and water (0.075 mL) (Note: HFIP and water were bubbled with argon gas for 10 minutes to remove oxygen), The reaction cuvette was purged with Ar for 1 min, then sealed with PTEF cap. The sample was stirred to make a clear solution (about 5 min.) and irradiated (λ = 436 nm, slit width = 10.0 nm) for 10800 s (3 h). After irradiation, the solvent was removed under reduced pressure. The yield of product formed was determined by ^{1}H NMR based on a TCE (1,1,2,2-tetrachloroethane, ~20 μL) standard to be 3.9%. The quantum yield was determined using equation 4:

$$\Phi = \frac{\text{mol product}}{\text{flux} \bullet \text{t} \bullet \text{f}} \quad \textbf{(Equation 4)}$$

$$\Phi = \frac{3.93 \text{ x } 10^{-6} \text{ mol}}{4.29 \text{ x } 10^{-10} \text{ einstein s}^{-1} \bullet 10800 \text{ s} \bullet 1.00} = 0.85$$

The quantum yield was calculated to be Φ (3.9%) = 0.85, indicating that a chain reaction mechanism can be excluded.

11. 3 Absorbance of catalyst:

The A (absorbance) of [Ru(bpy)3] Cl2 in HFIP was measured at the reaction concentration of 1.25×10^{-3} M and also at a substantially more dilute concentration of 1.25×10^{-5} M. The A at 436 nm for a 1.25×10^{-3} M solution is >10, while the A for 1.25×10^{-5} M solution is 0.188. This result indicated that the fraction of light absorbed (f) is about 1.00.

$$f = 1 - 10^{-A} = 1\text{-}10^{-10} = 1.00$$

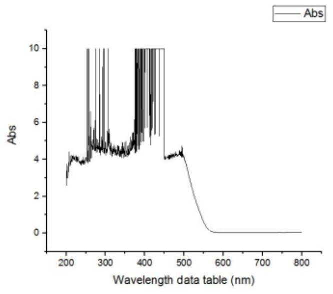

Figure S2. Absorbance of a 1.25 × 10^{-3} M solution of [Ru(bpy)3] Cl2 in HFIP.

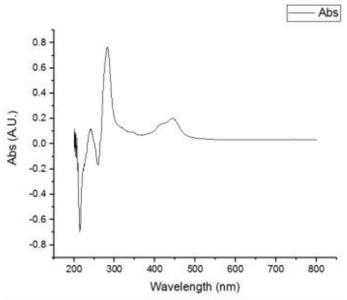

Figure S3. Absorbance of a 1.25 × 10^{-5} M solution of [Ru(bpy)3] Cl2 in HFIP.

12. The luminescence quenching (Stern-Volmer) experiments

The luminescence quenching experiments were carried out on a fluorescence spectrophotometer. To a glass cuvette with a PTEF cap, photocatalyst $[Ru(bpy)_3]Cl_2$, quencher PFBI-OH (compound **17**) or compound **7**, and HFIP were added to obtain a total volume of 200 μL. Before determination, the solution was degassed by three freeze-pump-thaw cycles and backfilled with argon. The concentration of $[Ru(bpy)_3]Cl_2$ was 1.0 × 10^{-4} M. All samples were irradiated at 452 nm, and emission was determined at 567 nm. It should be noted that all samples were measured within 1 minute after preparation.

The result showed that the excited state of Ru (Ⅱ) * can be quenched by PFBI-OH, while no obvious change of Ru (Ⅱ) * luminescence in the presence of variable concentrations of compound **7** was observed.

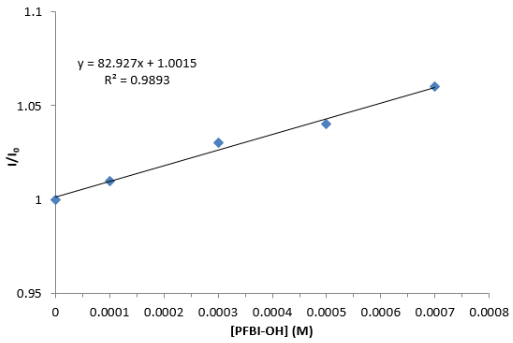

Figure S4. Stern-Volmer quenching studies for PFBI-OH.

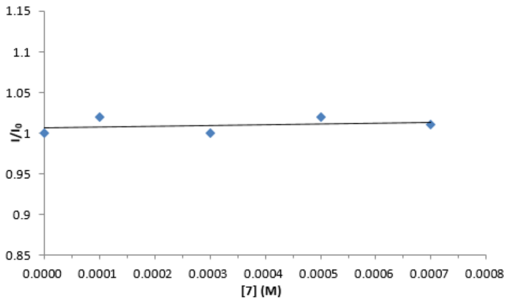

Figure S5. Stern-Volmer quenching studies for **7**.

13. DFT calculations

13.1 Computational details

All DFT calculations were performed with the Gaussian 09 software package.[29] Geometries were optimized using the M06-2X functional and the 6-31+G(d) basis set in the gas phase. Single point energies were calculated using M06-2X and 6-311++G(d,p) and the SMD solvation model in HFIP.[30] Since the solvent parameters for HFIP are not available in Gaussian 09. The parameters of isopropanol were used and the dielectric constant of the solvent was modified to the dielectric constant of HFIP[31] ($\varepsilon = 16.7$) by using the "scrf=(smd,solvent=2-propanol,read)" keywords in the Gaussian 09 calculations. The reported Gibbs free energies and enthalpies include zero-point vibrational energies and thermal corrections computed at 298 K.

The experimental redox potentials of $E^{\ominus}_{\mathrm{Ru(bpy)_3^{3+/2+*}}}$ (−0.81 V vs SCE in MeCN) and $E^{\ominus}_{\mathrm{Ru(bpy)_3^{3+/2+}}}$ (+1.29 V vs SCE in MeCN)[32] were used in the computation of the SET

reaction energies with Ru(II)* and Ru(III)m including the initial SET reduction with Ru(II) and in the subsequent SET oxidation with Ru(III).[6]

13.2 Potential energy profile of C(sp^3)−H hydroxylation with BI-OH

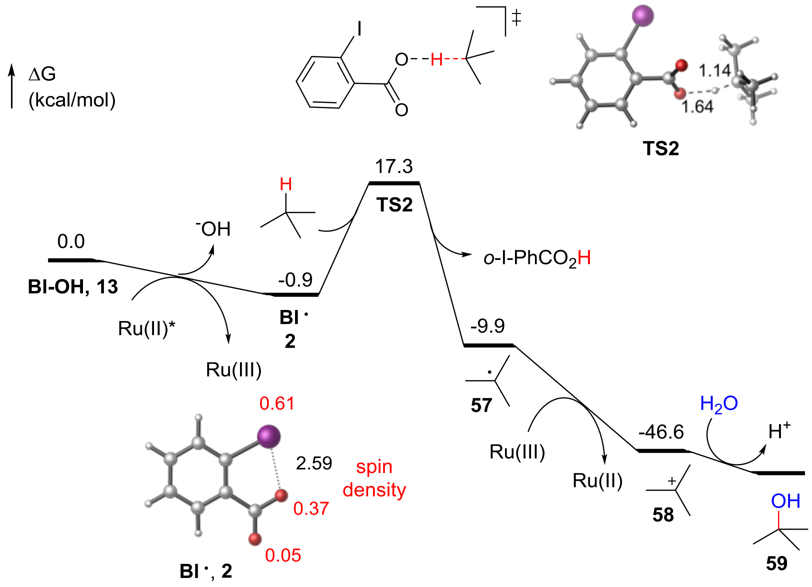

Scheme S23

13.3 H-atom abstraction of *tert*-butane with PFBI• and BI• through the I-centered pathway

Although the computed spin densities of **PFBI•** and **BI•** are delocalized over the O and I atoms, the H-abstraction occurs exclusively via the *O*-centered pathway to form *ortho*-iodo benzoic acid and its perfluorinated derivative. This *O*-centered pathway is exergonic by 13.7 kcal/mol and 9.0 kcal/mol for the reactions with **PFBI•** and **BI•**, respectively. In contrast, the H-abstraction via the *I*-centered pathway to form **60** and **61** is highly endergonic.

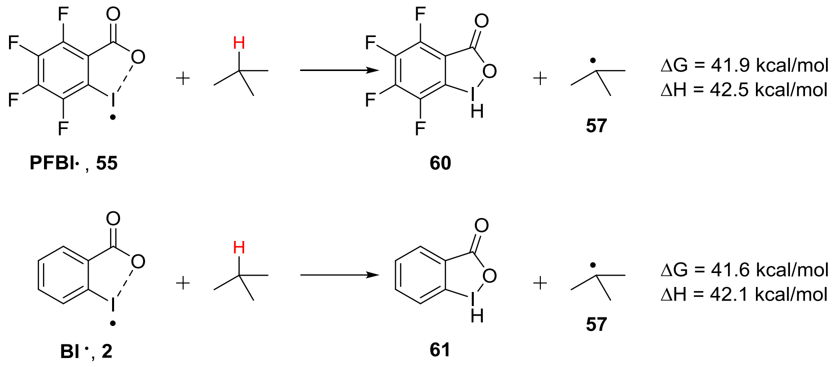

Scheme S24

The transition states for these processes could not be located by calculations. Scan of the H-abstraction reaction coordinates to form the H-I bond indicated a highly endothermic and barrierless transformation with both PFBI• and BI•. These results indicate the H-atom abstraction takes place via the *O*-centered pathway exclusively.

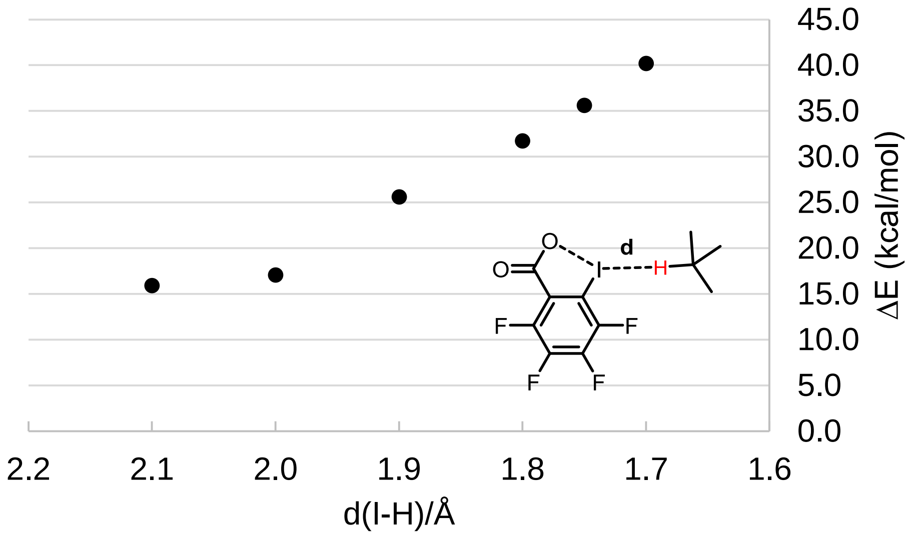

Chart 1

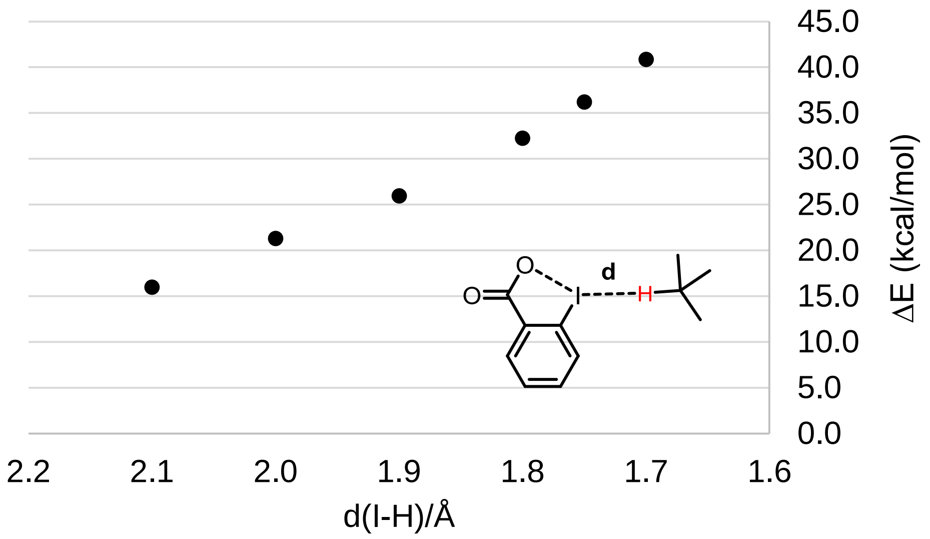
45.0
40.0
35.0
30.0
25.0
20.0
15.0
10.0
5.0
0.0
ΔE (kcal/mol)
2.2
2.1
2.0
1.9
1.8
1.7
1.6
d(I-H)/Å
O
O
I
H
d

Chart 2

13.4 Cartesian coordinates and energies of optimized structures

PFBI-OH
M06-2X/6-31+G(d) SCF energy: -903.31356630 a.u.
M06-2X/6-31+G(d) enthalpy: -903.223347 a.u.
M06-2X/6-31+G(d) free energy: -903.278767 a.u.
M06-2X/6-311++G(d,p) SCF energy in solution: -903.59971492 a.u.
M06-2X/6-311++G(d,p) enthalpy: -903.509496 a.u.
M06-2X/6-311++G(d,p) free energy: -903.564916 a.u.

Cartesian coordinates

ATOM	X	Y	Z
C	-0.704924	0.991232	0.012661
C	-0.049290	-0.234725	-0.015761
C	-0.739258	-1.432811	-0.050404
C	-2.132182	-1.404469	-0.019237
C	-2.808355	-0.195764	0.036731
C	-2.096440	1.000864	0.044450
C	0.121651	2.267728	-0.045013
O	1.400567	2.014839	-0.169365
O	-0.385090	3.361103	-0.000702
I	2.078588	-0.024863	0.004282
O	2.376022	-1.999664	0.309679
H	2.256397	-2.535499	-0.490713
F	-0.159598	-2.625831	-0.151212
F	-2.810831	-2.542565	-0.055974
F	-4.133559	-0.187965	0.062844
F	-2.796097	2.121991	0.073934

55
M06-2X/6-31+G(d) SCF energy: -827.54891344 a.u.
M06-2X/6-31+G(d) enthalpy: -827.475533 a.u.
M06-2X/6-31+G(d) free energy: -827.529063 a.u.
M06-2X/6-311++G(d,p) SCF energy in solution: -827.79532614 a.u.
M06-2X/6-311++G(d,p) enthalpy: -827.721946 a.u.
M06-2X/6-311++G(d,p) free energy: -827.775476 a.u.

Cartesian coordinates

ATOM	X	Y	Z
C	-0.490788	0.981398	-0.000037
C	0.093871	-0.285450	-0.000006
C	-0.670247	-1.437062	0.000030

C	-2.058074	-1.345042	0.000027
C	-2.662398	-0.096924	-0.000024
C	-1.884536	1.058416	-0.000028
C	0.357247	2.227600	0.000014
O	1.658595	2.045384	-0.000021
O	-0.103467	3.347442	0.000091
F	-0.109030	-2.645384	0.000088
F	-2.800132	-2.442593	0.000020
F	-3.985564	-0.009900	-0.000091
F	-2.526483	2.214391	0.000035
I	2.193196	-0.449223	-0.000017

Isobutane

M06-2X/6-31+G(d) SCF energy: -158.36179771 a.u.

M06-2X/6-31+G(d) enthalpy: -158.222321 a.u.

M06-2X/6-31+G(d) free energy: -158.256686 a.u.

M06-2X/6-311++G(d,p) SCF energy in solution: -158.40978520 a.u.

M06-2X/6-311++G(d,p) enthalpy: -158.270308 a.u.

M06-2X/6-311++G(d,p) free energy: -158.304673 a.u.

Cartesian coordinates

ATOM	X	Y	Z
C	-0.000082	0.000024	-0.381074
H	-0.000129	-0.000025	-1.480296
C	-0.029637	-1.452044	0.096502
H	-0.030007	-1.493040	1.193314
H	-0.927262	-1.970727	-0.257930
H	0.845669	-2.006965	-0.258778
C	-1.242864	0.751720	0.096506
H	-1.278782	0.771472	1.193335
H	-1.242751	1.788769	-0.256955
H	-2.161172	0.271921	-0.259739
C	1.272470	0.700305	0.096451
H	2.170479	0.182723	-0.258742
H	1.315882	1.736188	-0.257818
H	1.308755	0.719654	1.193296

TS1

M06-2X/6-31+G(d) SCF energy: -985.91034576 a.u.

M06-2X/6-31+G(d) enthalpy: -985.698697 a.u.

M06-2X/6-31+G(d) free energy: -985.770350 a.u.
M06-2X/6-311++G(d,p) SCF energy in solution: -986.19376847 a.u.
M06-2X/6-311++G(d,p) enthalpy: -985.982120 a.u.
M06-2X/6-311++G(d,p) free energy: -986.053773 a.u.
Imaginary frequency: -188.3224 cm-1

Cartesian coordinates

ATOM	X	Y	Z
C	-2.728307	1.896208	0.052610
C	-1.352459	1.976484	-0.117270
C	-0.571888	0.828657	-0.181985
C	-1.169341	-0.428110	-0.040978
C	-2.542277	-0.505633	0.132668
C	-3.321873	0.648263	0.178042
C	0.920434	0.981516	-0.423054
O	1.459172	0.870166	-1.492742
I	-0.035993	-2.203505	-0.077023
O	1.455178	1.238183	0.746772
C	4.157921	0.796504	0.272176
C	3.908166	-0.696320	0.106263
H	3.314636	-0.897935	-0.791633
H	4.865411	-1.226180	0.010940
H	3.378405	-1.107715	0.972909
C	4.925763	1.112892	1.549572
H	5.923461	0.655487	1.504765
H	5.056470	2.191769	1.682153
H	4.412952	0.715075	2.431531
C	4.794811	1.413829	-0.965952
H	4.925851	2.494922	-0.852082
H	5.786444	0.969554	-1.128852
H	4.183406	1.227771	-1.853561
H	3.142642	1.281107	0.389145
F	-3.161604	-1.675575	0.266983
F	-4.633750	0.555867	0.344503
F	-3.472011	2.994012	0.098679
F	-0.790695	3.177847	-0.231041

56

M06-2X/6-31+G(d) SCF energy: -828.22716198 a.u.
M06-2X/6-31+G(d) enthalpy: -828.140268 a.u.
M06-2X/6-31+G(d) free energy: -828.194685 a.u.
M06-2X/6-311++G(d,p) SCF energy in solution: -828.47380042 a.u.

M06-2X/6-311++G(d,p) enthalpy: -828.386906 a.u.
M06-2X/6-311++G(d,p) free energy: -828.441323 a.u.

Cartesian coordinates

ATOM	X	Y	Z
C	-0.506351	0.956738	0.000206
C	0.126608	-0.289936	0.015356
C	-0.645280	-1.443545	0.023499
C	-2.034864	-1.373688	0.023416
C	-2.665271	-0.139272	0.008812
C	-1.894032	1.016085	-0.008405
C	0.243595	2.258954	-0.044151
O	0.221907	3.012680	-0.979357
F	-0.087356	-2.652249	0.025896
F	-2.758017	-2.486417	0.036147
F	-3.990558	-0.067935	0.015646
F	-2.517901	2.192748	-0.007122
I	2.224942	-0.491682	-0.049778
O	0.917789	2.480914	1.091822
H	1.398553	3.323061	0.991018

57

M06-2X/6-31+G(d) SCF energy: -157.70081077 a.u.
M06-2X/6-31+G(d) enthalpy: -157.575321 a.u.
M06-2X/6-31+G(d) free energy: -157.611634 a.u.
M06-2X/6-311++G(d,p) SCF energy in solution: -157.74850228 a.u.
M06-2X/6-311++G(d,p) enthalpy: -157.623013 a.u.
M06-2X/6-311++G(d,p) free energy: -157.659326 a.u.

Cartesian coordinates

ATOM	X	Y	Z
C	0.000150	-0.000077	-0.188259
C	1.439547	-0.346451	0.019683
H	1.691368	-0.400229	1.094475
H	1.686765	-1.322688	-0.412386
H	2.104195	0.403825	-0.423442
C	-1.019864	-1.073152	0.019669
H	-1.199138	-1.258129	1.094416
H	-1.986572	-0.802478	-0.419990
H	-0.698683	-2.025937	-0.416117
C	-0.419655	1.419634	0.019619
H	0.298472	2.122089	-0.418170

H	-1.404871	1.617628	-0.417563
H	-0.492608	1.666192	1.094508

58

M06-2X/6-31+G(d) SCF energy: -157.45073926 a.u.
M06-2X/6-31+G(d) enthalpy: -157.325618 a.u.
M06-2X/6-31+G(d) free energy: -157.362308 a.u.
M06-2X/6-311++G(d,p) SCF energy in solution: -157.59472172 a.u.
M06-2X/6-311++G(d,p) enthalpy: -157.469600 a.u.
M06-2X/6-311++G(d,p) free energy: -157.506290 a.u.

Cartesian coordinates

ATOM	X	Y	Z
C	-0.002960	0.001259	0.008702
C	-1.408991	0.401326	0.011228
H	-1.592008	0.823086	-0.997031
H	-2.106416	-0.421148	0.172397
H	-1.587475	1.237156	0.697475
C	0.353320	-1.415836	-0.014658
H	-0.348271	-2.005719	-0.614734
H	1.393749	-1.611490	-0.277080
H	0.191229	-1.757103	1.027942
C	1.052629	1.014789	0.008884
H	0.702491	2.029458	0.195539
H	1.857552	0.729930	0.700171
H	1.525164	0.966594	-0.989618

59

M06-2X/6-31+G(d) SCF energy: -233.55853272 a.u.
M06-2X/6-31+G(d) enthalpy: -233.413903 a.u.
M06-2X/6-31+G(d) free energy: -233.450696 a.u.
M06-2X/6-311++G(d,p) SCF energy in solution: -233.64296474 a.u.
M06-2X/6-311++G(d,p) enthalpy: -233.498335 a.u.
M06-2X/6-311++G(d,p) free energy: -233.535128 a.u.

Cartesian coordinates

ATOM	X	Y	Z
C	0.682400	1.258692	-0.512776
H	0.631241	1.306413	-1.605900
H	0.202908	2.151308	-0.099271

H	1.741638	1.271920	-0.226292
C	-1.484749	-0.001192	-0.347041
H	-1.616025	-0.000966	-1.433730
H	-1.972196	-0.889052	0.067310
H	-1.973736	0.885563	0.067865
C	0.684502	-1.257434	-0.513029
H	1.743797	-1.268902	-0.226663
H	0.206584	-2.150949	-0.099640
H	0.633329	-1.305065	-1.606155
C	-0.005242	-0.000003	0.016259
O	0.024122	-0.000111	1.445552
H	0.948022	0.000238	1.737575

BI-OH

M06-2X/6-31+G(d) SCF energy: -506.51406153 a.u.
M06-2X/6-31+G(d) enthalpy: -506.394811 a.u.
M06-2X/6-31+G(d) free energy: -506.441483 a.u.
M06-2X/6-311++G(d,p) SCF energy in solution: -506.68248934 a.u.
M06-2X/6-311++G(d,p) enthalpy: -506.563239 a.u.
M06-2X/6-311++G(d,p) free energy: -506.609911 a.u.

Cartesian coordinates

ATOM	X	Y	Z
C	-1.483420	0.446000	-0.015623
C	-0.565543	-0.584021	-0.002860
C	-0.892175	-1.926239	0.029705
C	-2.254746	-2.234497	0.025923
C	-3.218230	-1.222878	-0.002684
C	-2.838761	0.116482	-0.020518
C	-0.991579	1.873773	-0.012062
H	-0.119375	-2.686367	0.070603
H	-2.561717	-3.275661	0.051217
H	-4.271600	-1.484830	-0.004308
H	-3.564518	0.924327	-0.033884
O	0.319473	1.962361	0.020619
O	-1.756625	2.812034	-0.033267
I	1.443989	0.118637	0.013608
O	2.082121	-1.829842	0.020237
H	2.292735	-2.133363	-0.876845

2

M06-2X/6-31+G(d) SCF energy: -430.74156239 a.u.
M06-2X/6-31+G(d) enthalpy: -430.639154 a.u.
M06-2X/6-31+G(d) free energy: -430.684411 a.u.
M06-2X/6-311++G(d,p) SCF energy in solution: -430.87128459 a.u.
M06-2X/6-311++G(d,p) enthalpy: -430.768876 a.u.
M06-2X/6-311++G(d,p) free energy: -430.814133 a.u.

Cartesian coordinates

ATOM	X	Y	Z
C	1.261548	0.516621	-0.000005
C	0.459383	-0.617658	-0.000047
C	0.995309	-1.899332	0.000029
C	2.382684	-2.039015	0.000175
C	3.208614	-0.914240	0.000272
C	2.648845	0.358264	0.000190
C	0.700006	1.909792	-0.000153
H	0.352160	-2.773122	0.000015
H	2.814777	-3.035214	0.000251
H	4.287237	-1.033062	0.000405
H	3.260865	1.255478	0.000238
O	-0.606247	2.036954	-0.000780
O	1.410723	2.897249	0.000386
I	-1.643192	-0.335364	-0.000010

TS2

M06-2X/6-31+G(d) SCF energy: -589.09544762 a.u.
M06-2X/6-31+G(d) enthalpy: -588.855364 a.u.
M06-2X/6-31+G(d) free energy: -588.920283 a.u.
M06-2X/6-311++G(d,p) SCF energy in solution: -589.26501924 a.u.
M06-2X/6-311++G(d,p) enthalpy: -589.024936 a.u.
M06-2X/6-311++G(d,p) free energy: -589.089855 a.u.
Imaginary frequency: -238.1348 cm-1

Cartesian coordinates

ATOM	X	Y	Z
C	2.583013	3.006222	-0.118467
C	1.246090	2.669031	0.059677
C	0.861815	1.326209	0.129259
C	1.818606	0.321584	-0.027326
C	3.157165	0.656056	-0.211966
C	3.535596	1.997777	-0.252007

H	2.879196	4.049474	-0.157678
H	0.488279	3.440567	0.159227
H	3.901939	-0.124332	-0.327943
H	4.581877	2.249749	-0.396419
C	-0.591284	1.007387	0.409975
O	-1.018556	0.586011	1.457417
I	1.264906	-1.727498	-0.013748
O	-1.267900	1.291623	-0.680286
C	-3.738806	0.220432	-0.170000
C	-3.247871	-1.219840	-0.160348
H	-2.577663	-1.398517	0.686158
H	-4.103527	-1.903722	-0.075227
H	-2.711652	-1.462295	-1.084475
C	-4.614566	0.530924	-1.376058
H	-5.529738	-0.075679	-1.336352
H	-4.911010	1.584754	-1.395852
H	-4.097833	0.298641	-2.313006
C	-4.378830	0.625468	1.149773
H	-4.679224	1.678609	1.142726
H	-5.279800	0.020432	1.323671
H	-3.687475	0.464511	1.981593
H	-2.807303	0.866620	-0.289909

o-I-PhCO2H

M06-2X/6-31+G(d) SCF energy: -431.41442373 a.u.

M06-2X/6-31+G(d) enthalpy: -431.298633 a.u.

M06-2X/6-31+G(d) free energy: -431.344367 a.u.

M06-2X/6-311++G(d,p) SCF energy in solution: -431.54393562 a.u.

M06-2X/6-311++G(d,p) enthalpy: -431.428145 a.u.

M06-2X/6-311++G(d,p) free energy: -431.473879 a.u.

Cartesian coordinates

ATOM	X	Y	Z
C	-1.342260	0.359581	0.008533
C	-0.330068	-0.607949	0.049395
C	-0.666237	-1.962230	0.086626
C	-2.000632	-2.358901	0.088657
C	-3.014528	-1.405997	0.028350
C	-2.679520	-0.060309	-0.020434
C	-1.163600	1.845143	-0.005538
O	-1.964621	2.606006	-0.496199
I	1.756308	-0.188413	-0.060807

O	-0.063849	2.271254	0.630687
H	-0.050339	3.242665	0.560833
H	0.118948	-2.710359	0.108417
H	-2.240676	-3.417296	0.126575
H	-4.056626	-1.708404	0.016836
H	-3.446827	0.705175	-0.079333

60

M06-2X/6-31+G(d) SCF energy: -828.11136219 a.u.
M06-2X/6-31+G(d) enthalpy: -828.029603 a.u.
M06-2X/6-31+G(d) free energy: -828.082158 a.u.
M06-2X/6-311++G(d,p) SCF energy in solution: -828.38325054 a.u.
M06-2X/6-311++G(d,p) enthalpy: -828.301491 a.u.
M06-2X/6-311++G(d,p) free energy: -828.354046 a.u.

Cartesian coordinates

ATOM	X	Y	Z
C	2.684865	-0.017591	0.000042
C	1.861428	1.107003	0.000077
C	0.473217	0.977722	0.000107
C	-0.037268	-0.309962	0.000053
C	0.753784	-1.443030	0.000044
C	2.135143	-1.291351	0.000053
C	-0.486734	2.192076	0.000218
O	-1.718409	1.828966	0.000641
I	-2.171326	-0.426089	-0.000056
O	-0.022778	3.312163	-0.000833
H	-2.015586	-2.116610	-0.000467
F	0.244835	-2.678667	0.000013
F	2.922377	-2.359060	0.000036
F	4.003761	0.119706	0.000026
F	2.464443	2.282584	0.000081

61

M06-2X/6-31+G(d) SCF energy: -431.30576710 a.u.
M06-2X/6-31+G(d) enthalpy: -431.195268 a.u.
M06-2X/6-31+G(d) free energy: -431.239359 a.u.
M06-2X/6-311++G(d,p) SCF energy in solution: -431.45960752 a.u.
M06-2X/6-311++G(d,p) enthalpy: -431.349108 a.u.
M06-2X/6-311++G(d,p) free energy: -431.393199 a.u.

Cartesian coordinates

ATOM	X	Y	Z
C	3.269818	-0.745402	0.000059
C	2.640053	0.495263	0.000062
C	1.245570	0.575829	0.000011
C	0.546708	-0.610531	-0.000015
C	1.122823	-1.868873	-0.000022
C	2.517094	-1.922154	0.000008
H	4.353827	-0.802172	0.000088
H	3.197349	1.427265	0.000086
H	0.527172	-2.776790	-0.000049
H	3.010959	-2.889075	0.000002
C	0.523878	1.924526	-0.000027
O	-0.759502	1.811996	-0.000271
I	-1.585054	-0.340841	-0.000001
O	1.188690	2.945749	0.000176
H	-1.710600	-2.048594	0.000249

14. References

[1] M. V. Vita and J. Waser, *Org. Lett.*, 2013, **15,** 3246.

[2] M. Chen, Z.-T. Huang and Q.-Y. Zheng, *Org. Biomol. Chem.*, 2015, **13**, 8812.

[3] D. M. Schultz, J. W. Sawicki and T. P. Yoon, *Beilstein J. Org. Chem.*, 2015**, 11**, 61.

[4] R. D. Richardson, J. M. Zayed, S. Altermann, D. Smith and T. Wirth, *Angew. Chem., Int. Ed.*, 2007, **46**, 6529.

[5] E. McNeill and J. Du Bois, *Chem. Sci.* 2012, **3**, 1810.

[6] Y. Wang, G.-X. Li, G. Yang, G. He and G. Chen, *Chem. Sci.*, 2016, **7**, 2679.

[7] S. D. Dreher, S.-E Lim, D. L. Sandrock and G. A. Molander, *J. Org. Chem.*, 2009, **74**, 3626

[8] V. A. Schmidt, R. K. Quinn, A. T. Brusoe and E. J. Alexanian, *J. Am. Chem. Soc.* 2014, **136**, 14389.

[9] M. S. Wolfe, *Synth. Commun.,*1997, **27**, 2975.

[10] D. M. Shendage, R. Fröhlich and G. Haufe, *Org. Lett.*, 2004, **6**, 3675.

[11] D. Uraguchi, N. Kinoshita and T. Ooi, *J. Am. Chem. Soc.,* 2010, **132**, 12240.

[12] M. Y. Jiang and D. Dolphin, *J. Am. Chem. Soc.*, 2008, **130**, 4236.

[13] X. Li, X. Che, G.-H. Chen, J. Zhang, J.-L. Yan, Y.-F.Zhang, L.-S. Zhang, C.-P. Hsu, Y. Q. Gao and Z.-J. Shi, *Org. Lett.*, 2016, **18**, 1234.

[14] N. D. Litvinas, B. H. Brodsky and J. Du Bois, *Angew. Chem., Int. Ed.* 2009, **48**, 4513.

[15] I. Okada, K. Chiba and Y. Kitano, *Synthesis*, 2013, **45**, 1069.

[16] C. J. Easton, C. A. Hutton, E. W.Tan and E. R. T. Tiekink, *Tetrahedron Lett.,* 1990, **31**, 7059.

[17] T. Saito, Y. Nishimoto, M. Yasuda and A. Baba, *J. Org. Chem.,* 2006, **71**, 8516.

[18] L. Malet-Sanz, J. Madrzak, R. S. Holvey and T. Underwood, *Tetrahedron Lett.,* 2009, **50**, 7263.

[19] L.Cao, J. Ding, M. Gao, Z. Wang, J. Li and A. Wu, *Org. Lett.*, 2009, **11**, 3810.

[20] A. Cunningham, V. Mokal-Parekh, C. Wilson and S. Woodward, *Org. Biomol. Chem.*, 2004, **2**, 741.

[21] J. Wettergren, A. Bogevig, M. Portier and H. Adolfssona, *Adv. Synth. Catal.*, 2006, **348**, 1277.

[22] L. Adak, S. Bhadra and B. C. Ranu, *Tetrahedron Lett.*, 2010, **51**, 3811.

[23] L. H. Andrade and T. Barcellos, *Org. Lett.*, 2009, **11**, 3052.

[24] A. Rentmeister, F. H. Arnold and R. Fasan, *Nat. Chem. Biol.*, 2009, **5**, 26.

[25] S. Kamijo, Y. Amaoka and M. Inoue, *Synthesis*, 2010, **14**, 2475.

[26] K. Kiyokawa, K. Takemoto and S. Minakata, *Chem. Commun.*, 2016, **52**, 13082.

[27] T. Maegawa, A. Akashi, K. Yaguchi, Y. Iwasaki, M. Shigetsura, Y. Monguchi and H. Sajiki, *Chem. Eur. J.*, 2009, **15**, 6953.

[28] M. A. Cismesia and T. P. Yoon, *Chem. Sci.*, 2015, **6**, 5426.

[29] Gaussian 09, Revision D.01, M. J. Frisch, G. W. Trucks, H. B. Schlegel, G. E. Scuseria, M. A. Robb, J. R. Cheeseman, G. Scalmani, V. Barone, B. Mennucci, G. A. Petersson, H. Nakatsuji, M. Caricato, X. Li, H. P. Hratchian, A. F. Izmaylov, J. Bloino, G. Zheng, J. L. Sonnenberg, M. Hada, M. Ehara, K. Toyota, R. Fukuda, J. Hasegawa, M. Ishida, T. Nakajima, Y. Honda, O. Kitao, H. Nakai, T. Vreven, J. A. Montgomery, Jr., J. E. Peralta, F. Ogliaro, M. Bearpark, J. J. Heyd, E. Brothers, K. N. Kudin, V. N. Staroverov, R. Kobayashi, J. Normand, K. Raghavachari, A. Rendell, J. C. Burant, S. S. Iyengar, J. Tomasi, M. Cossi, N. Rega, J. M. Millam, M. Klene, J. E. Knox, J. B. Cross, V. Bakken, C. Adamo, J. Jaramillo, R. Gomperts, R. E. Stratmann, O. Yazyev, A. J. Austin, R. Cammi, C. Pomelli, J. W. Ochterski, R. L. Martin, K. Morokuma, V. G. Zakrzewski, G. A. Voth, P. Salvador, J. J. Dannenberg, S. Dapprich, A. D. Daniels, Ö. Farkas, J. B. Foresman, J. V. Ortiz, J. Cioslowski, and D. J. Fox, Gaussian, Inc., Wallingford CT, 2009.

[30] For recent computational studies on photoredox mediated C–C bond formation reactions, see: (a) O. Gutierrez, J. C. Tellis, D. N. Primer, G. A. Molander and M. C. Kozlowski, *J. Am. Chem. Soc.*, 2015, **137**, 4896; (b) T. B. Demissie, K. Ruud and J. H. Hansen, *Organometallics*, 2015, **34**, 4218; (c) T. B. Demissie, K. Ruud and J. H. Hansen, *J. Org. Chem.*, 2016, **81**, 7110.

[31] Handbook of Chemistry and Physics. 90th edition, D. R. Lide, *CRC Press*, 2009-

2010.

[32] For experimental redox potentials, see: C. R. Bock, T. J. Meyer and D. G. Whitten, *J. Am. Chem. Soc.*, 1975, **97**, 2909. For DFT calculations of redox potentials, see: ref. 6 and (a) P. Winget, C. J. Cramer and D. G. Truhlar, *Theor. Chem. Acc.*, 2004, **112**, 217; (b) A. A. Isse, C. Y. Lin, M. L. Coote and A. Gennaro *J. Phys. Chem. B*, 2011, **115**, 678; (c) H. G. Roth, N. A. Romero and D. A. Nicewicz, *Synlett*, 2016, **27**, 714.

15. ^{1}H-NMR, ^{13}C-NMR and ^{19}F-NMRspectra

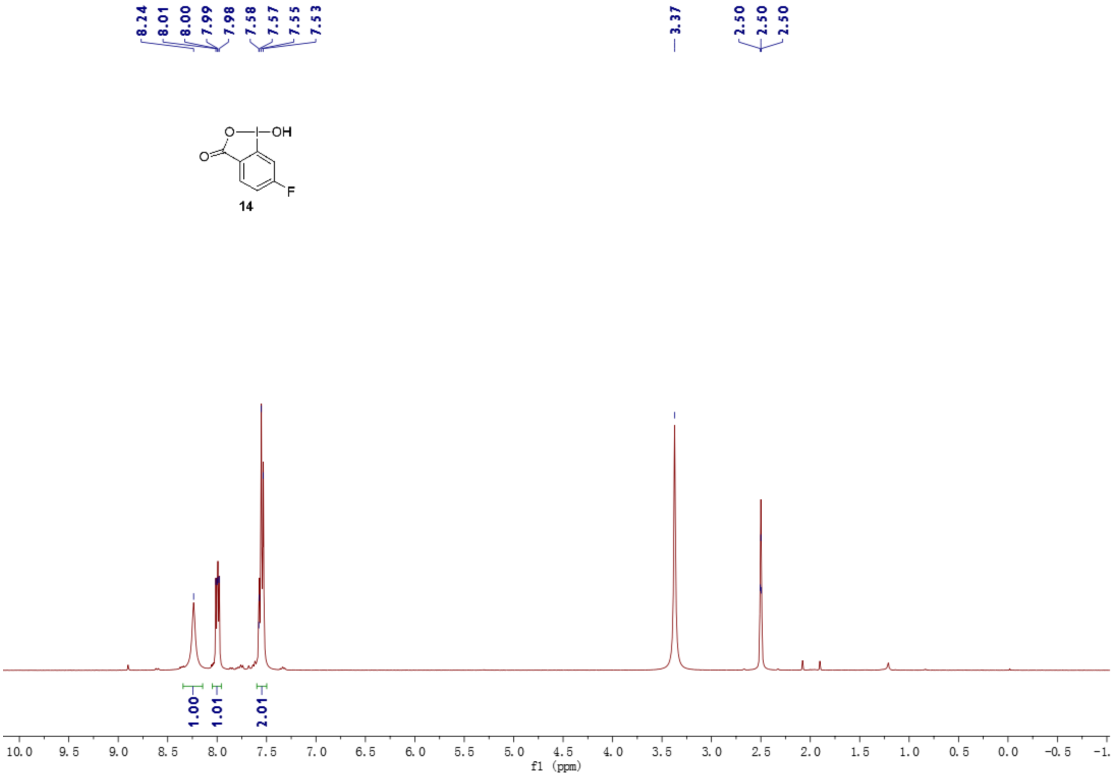

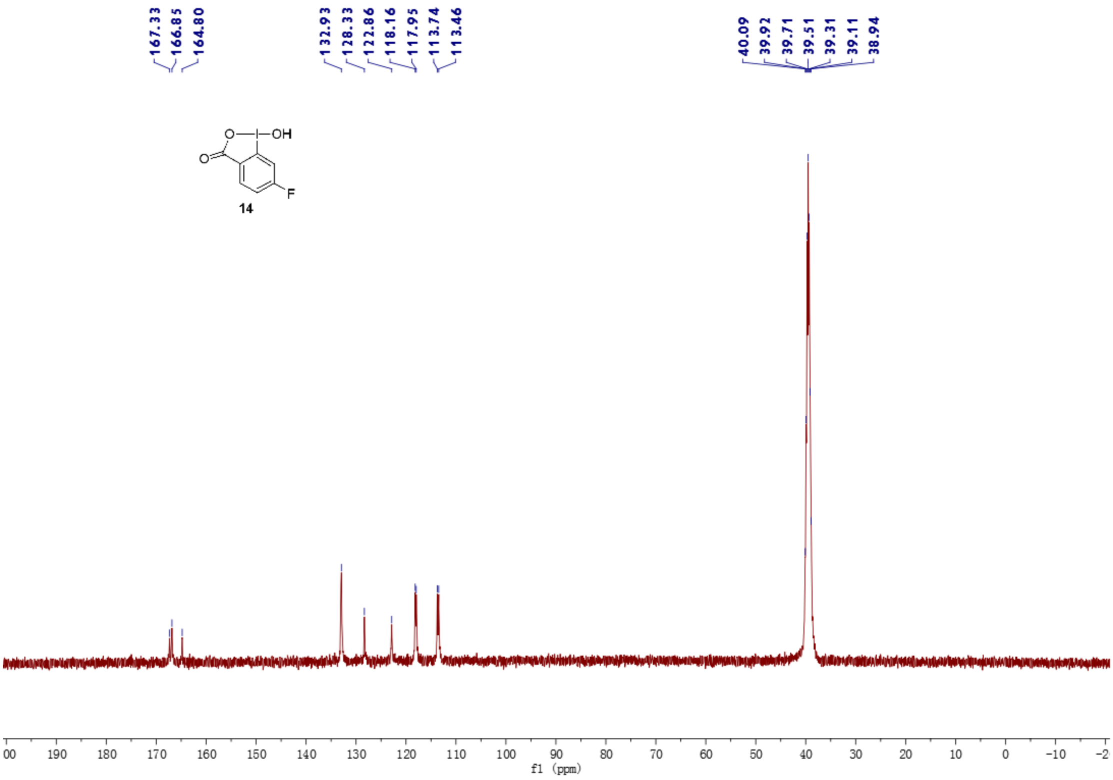

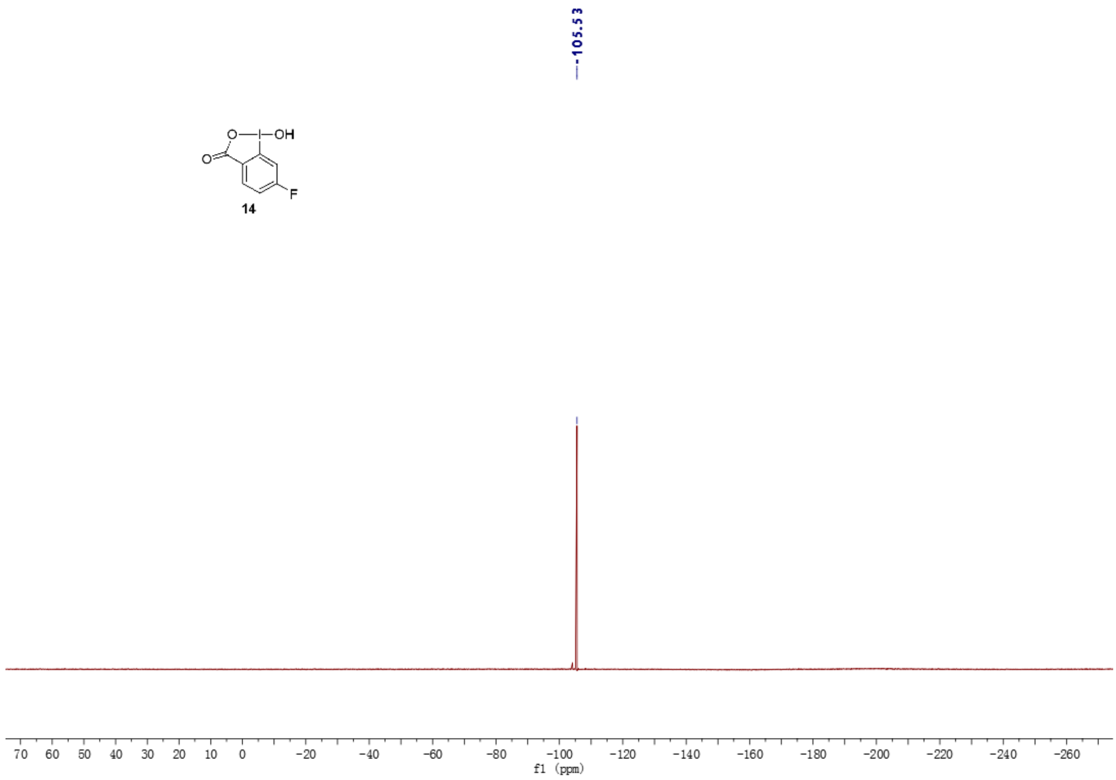

-105.53
O
I
OH
F
14
70 60 50 40 30 20 10 0 -20 -40 -60 -80 -100 -120 -140 -160 -180 -200 -220 -240 -260
f1 (ppm)

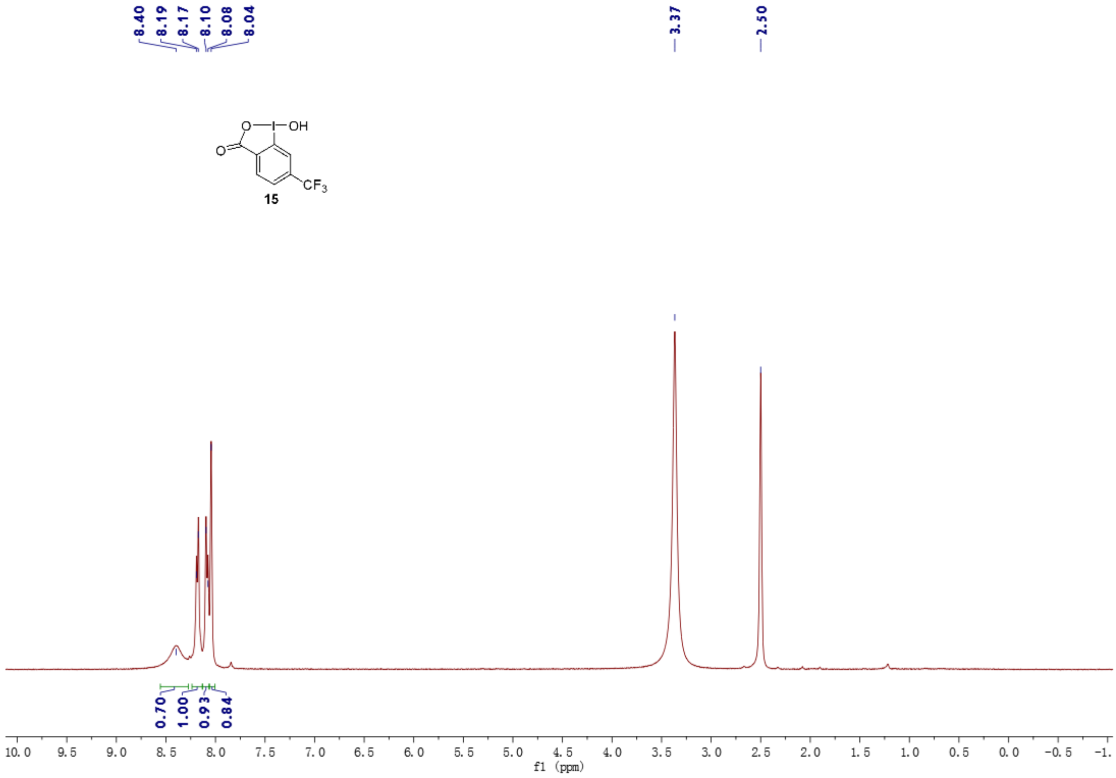

8.40
8.19
8.17
8.10
8.08
8.04
3.37
2.50
O
I
OH
CF3
15
0.70
1.00
0.93
0.84
10.0 9.5 9.0 8.5 8.0 7.5 7.0 6.5 6.0 5.5 5.0 4.5 4.0 3.5 3.0 2.5 2.0 1.5 1.0 0.5 0.0 -0.5 -1.
f1 (ppm)

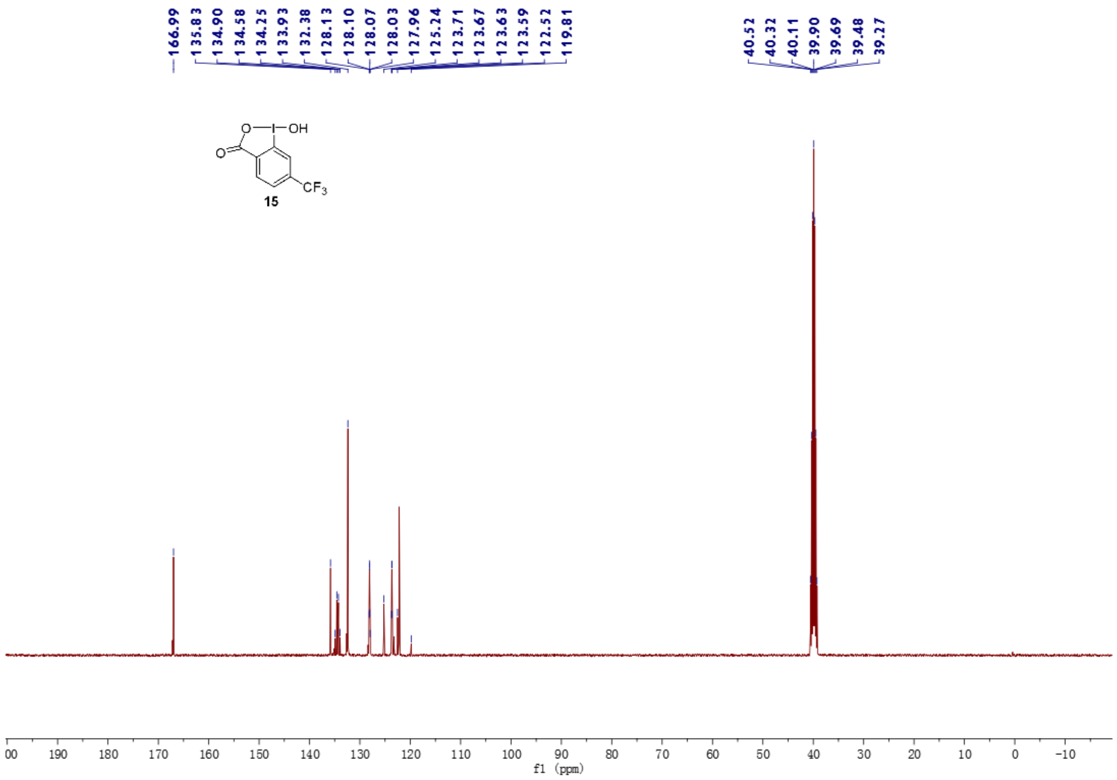

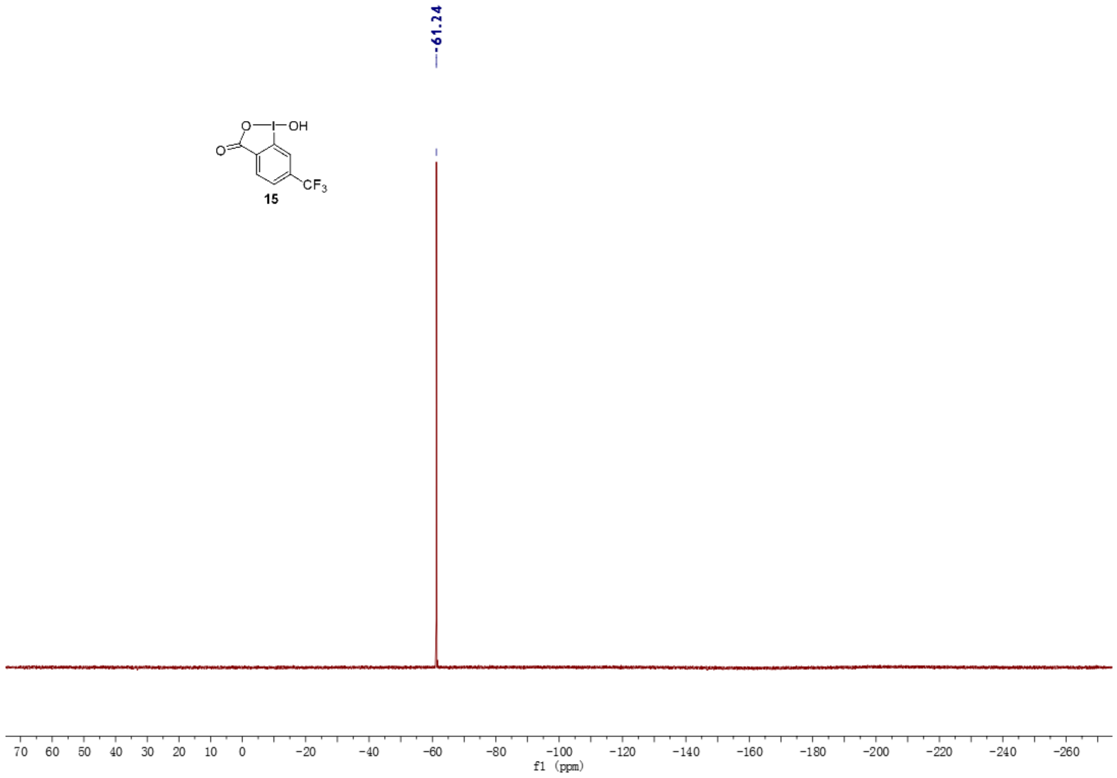

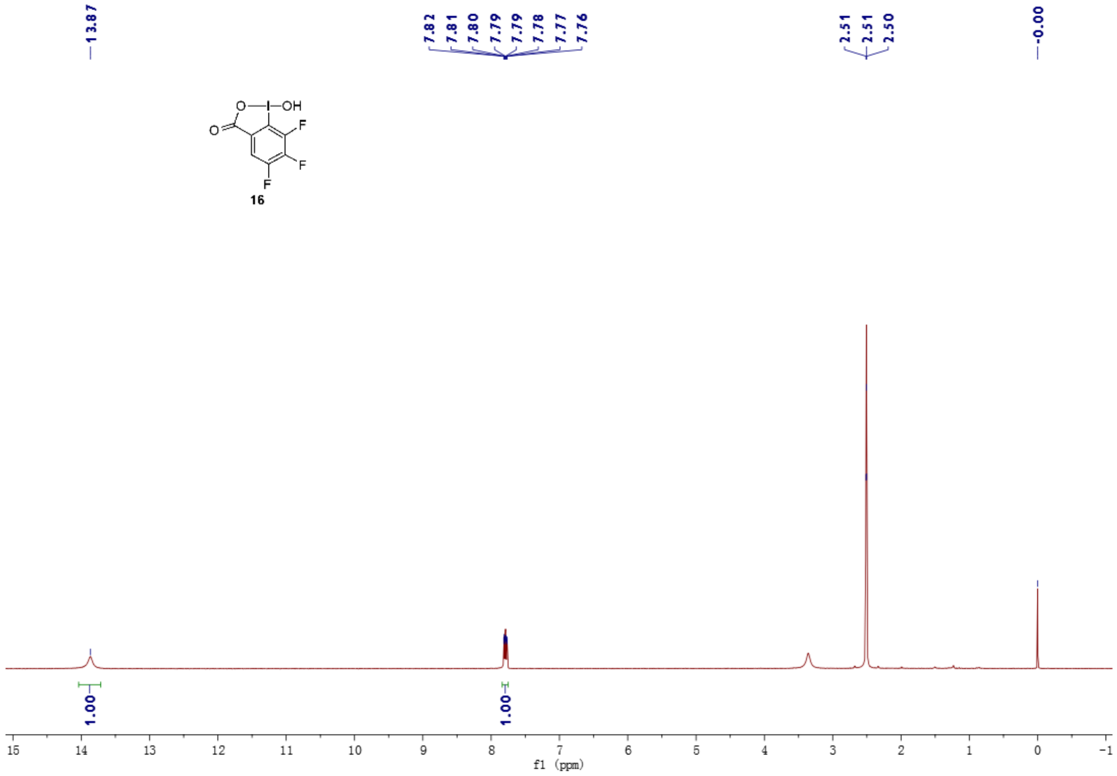
13.87
7.82
7.81
7.80
7.79
7.79
7.78
7.77
7.76
2.51
2.51
2.50
-0.00
O—I—OH
O
F
F
F
16
1.00
1.00
15 14 13 12 11 10 9 8 7 6 5 4 3 2 1 0 -1
f1 (ppm)

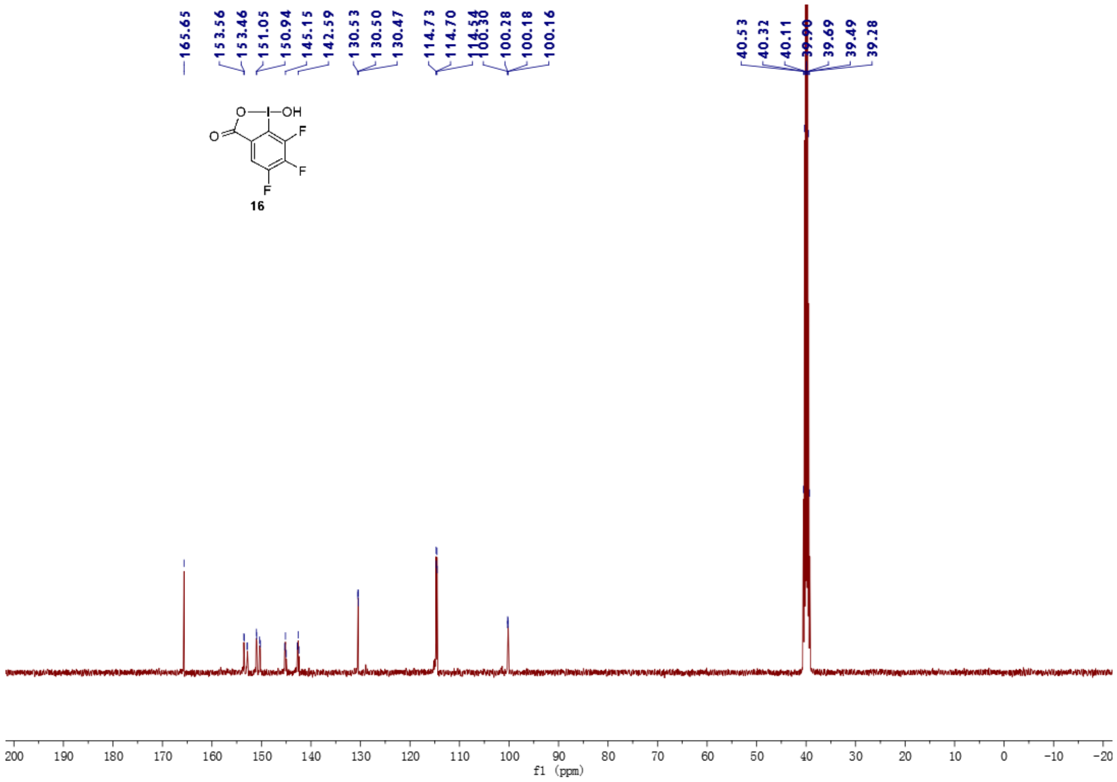
165.65
153.56
153.46
151.05
150.94
145.15
142.59
130.53
130.50
130.47
114.73
114.70
100.28
100.18
100.16
40.53
40.32
40.11
39.69
39.49
39.28
O—I—OH
O
F
F
F
16
200 190 180 170 160 150 140 130 120 110 100 90 80 70 60 50 40 30 20 10 0 -10 -20
f1 (ppm)

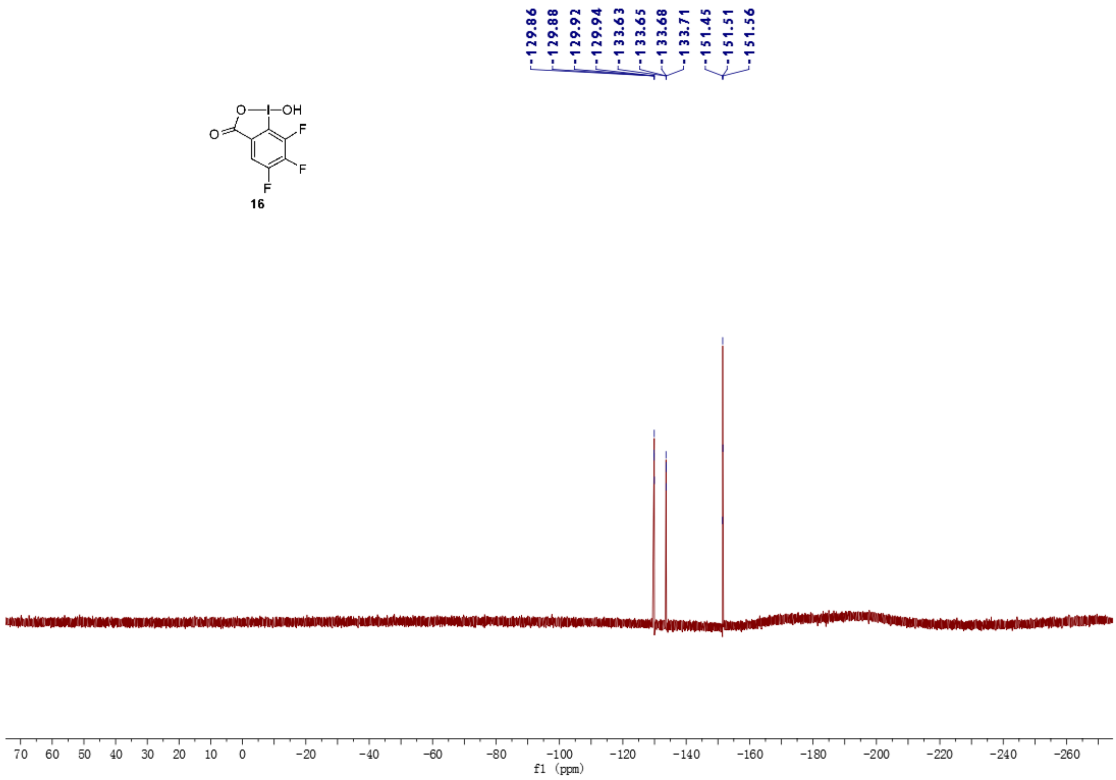
-129.86
-129.88
-129.92
-129.94
-133.63
-133.65
-133.68
-133.71
-151.45
-151.51
-151.56
16
f1 (ppm)

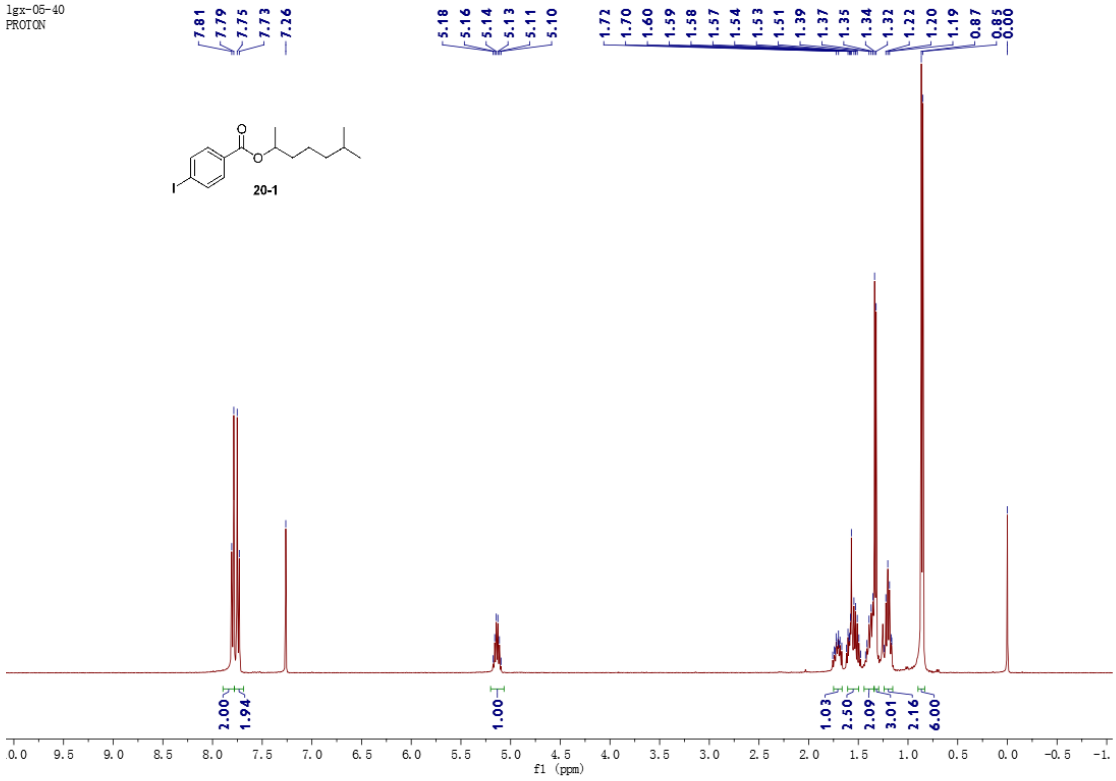
lgx-05-40
PROTON
7.81
7.79
7.75
7.73
7.26
5.18
5.16
5.14
5.13
5.11
5.10
1.72
1.70
1.60
1.59
1.58
1.57
1.54
1.53
1.51
1.39
1.37
1.35
1.34
1.32
1.22
1.20
1.19
0.87
0.85
0.00
20-1
2.00
1.94
1.00
1.03
2.50
2.09
3.01
2.16
6.00
f1 (ppm)

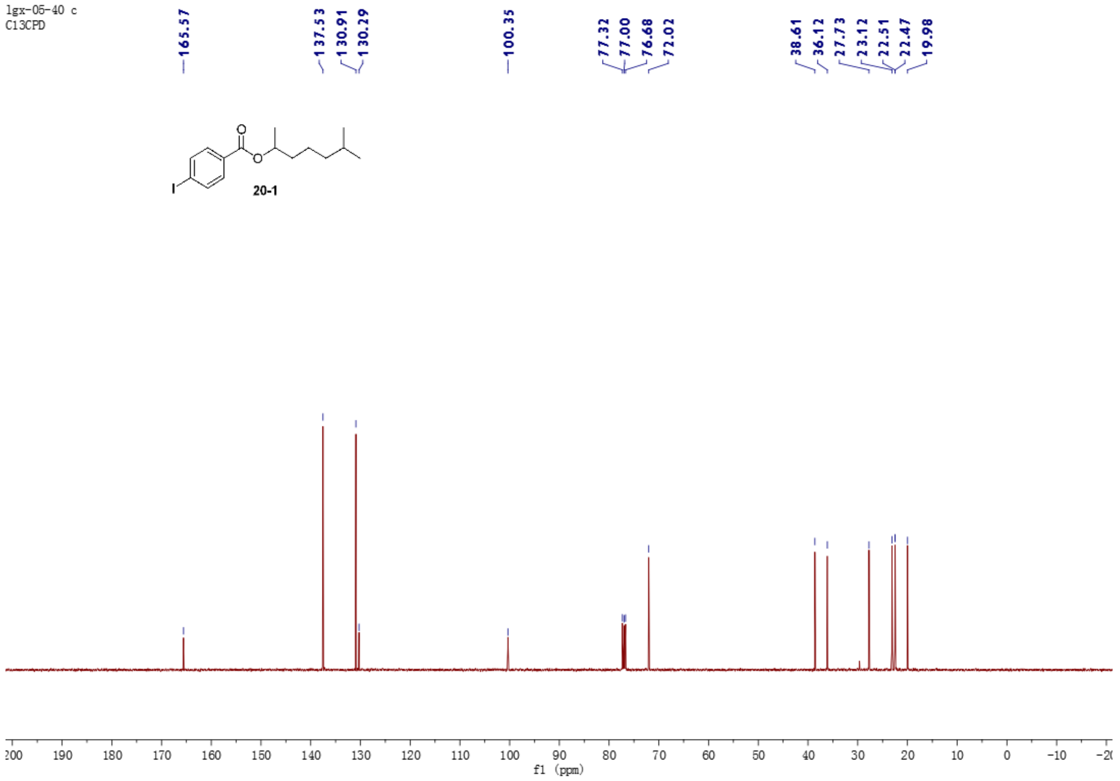
lgx-05-40 c
C13CPD
165.57
137.53
130.91
130.29
100.35
77.32
77.00
76.68
72.02
38.61
36.12
27.73
23.12
22.51
22.47
19.98
20-1
f1 (ppm)

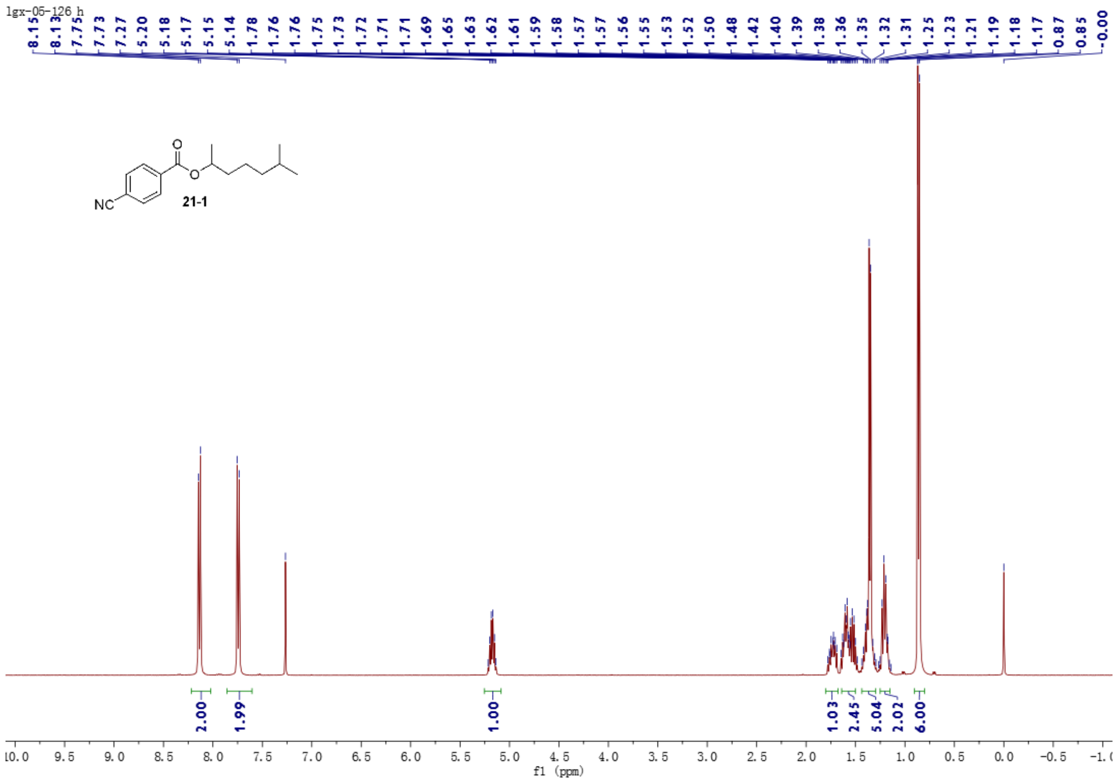
lgx-05-126 h
NC
21-1
f1 (ppm)

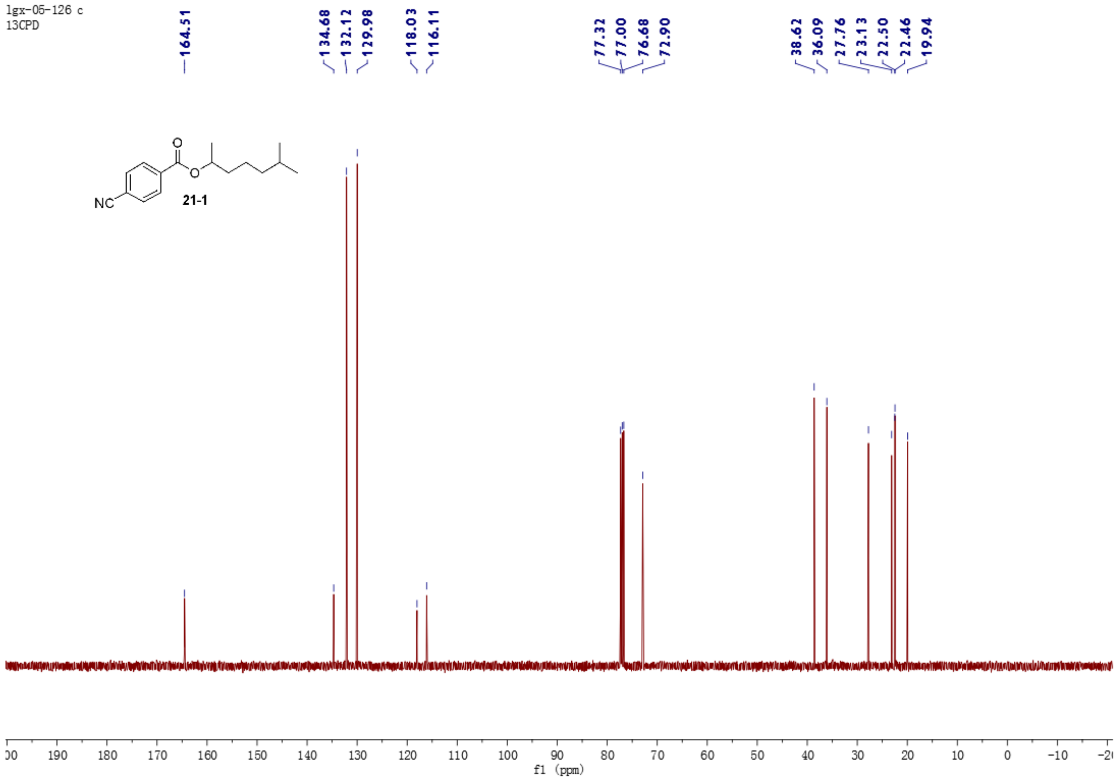
lgx-05-126 c
13CPD
164.51
134.68
132.12
129.98
118.03
116.11
77.32
77.00
76.68
72.90
38.62
36.09
27.76
23.13
22.50
22.46
19.94
NC
O
O
21-1
f1 (ppm)

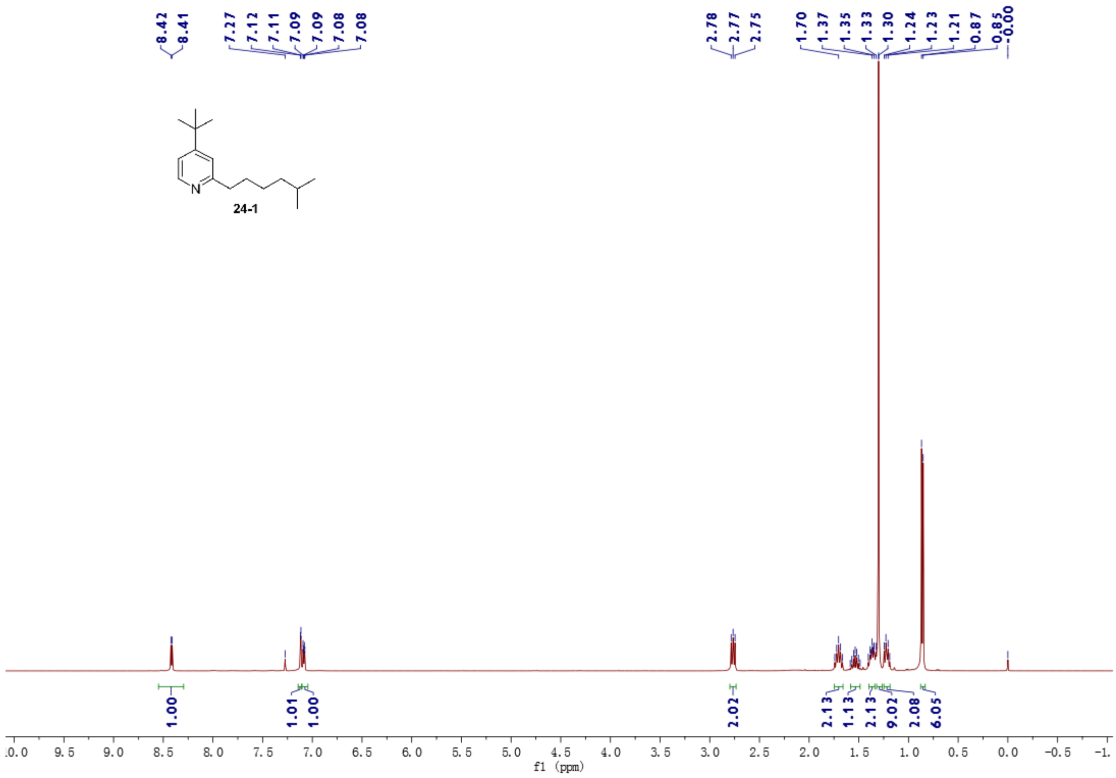
8.42
8.41
7.27
7.12
7.11
7.09
7.09
7.08
7.08
2.78
2.77
2.75
1.70
1.37
1.35
1.33
1.30
1.24
1.23
1.21
0.87
0.85
0.00
N
24-1
1.00
1.01
1.00
2.02
2.13
1.13
2.13
9.02
2.08
6.05
f1 (ppm)

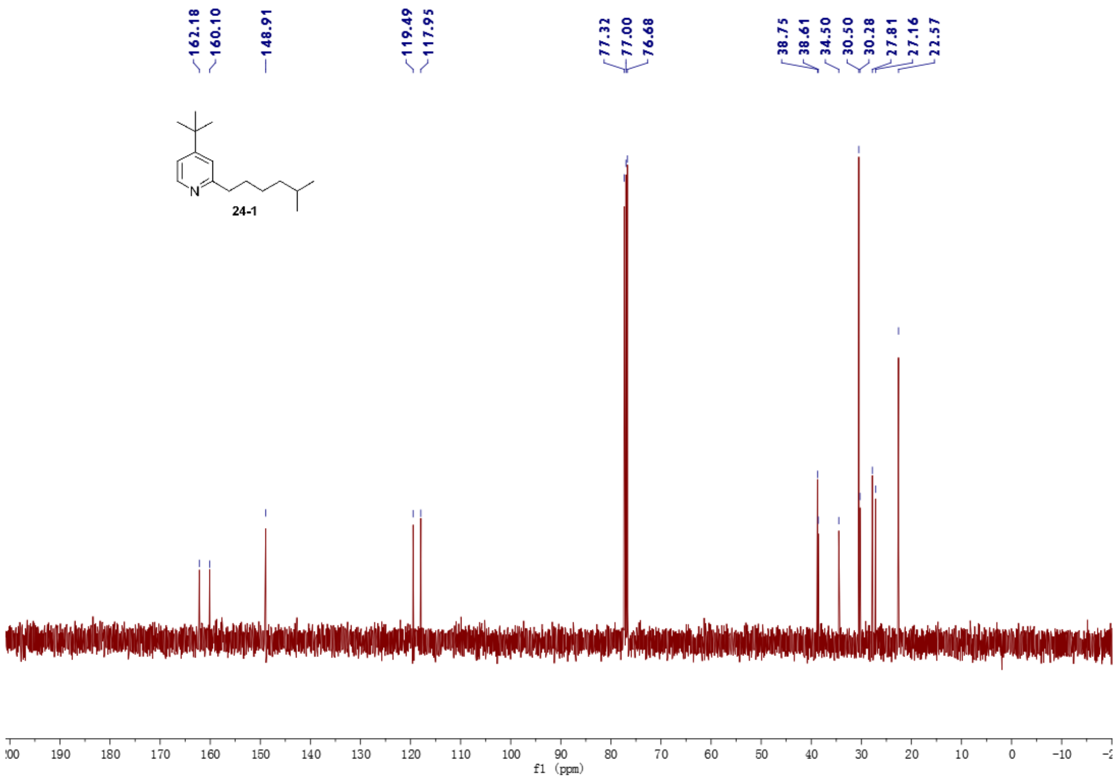
162.18
160.10
148.91
119.49
117.95
77.32
77.00
76.68
38.75
38.61
34.50
30.50
30.28
27.81
27.16
22.57
N
24-1
f1 (ppm)

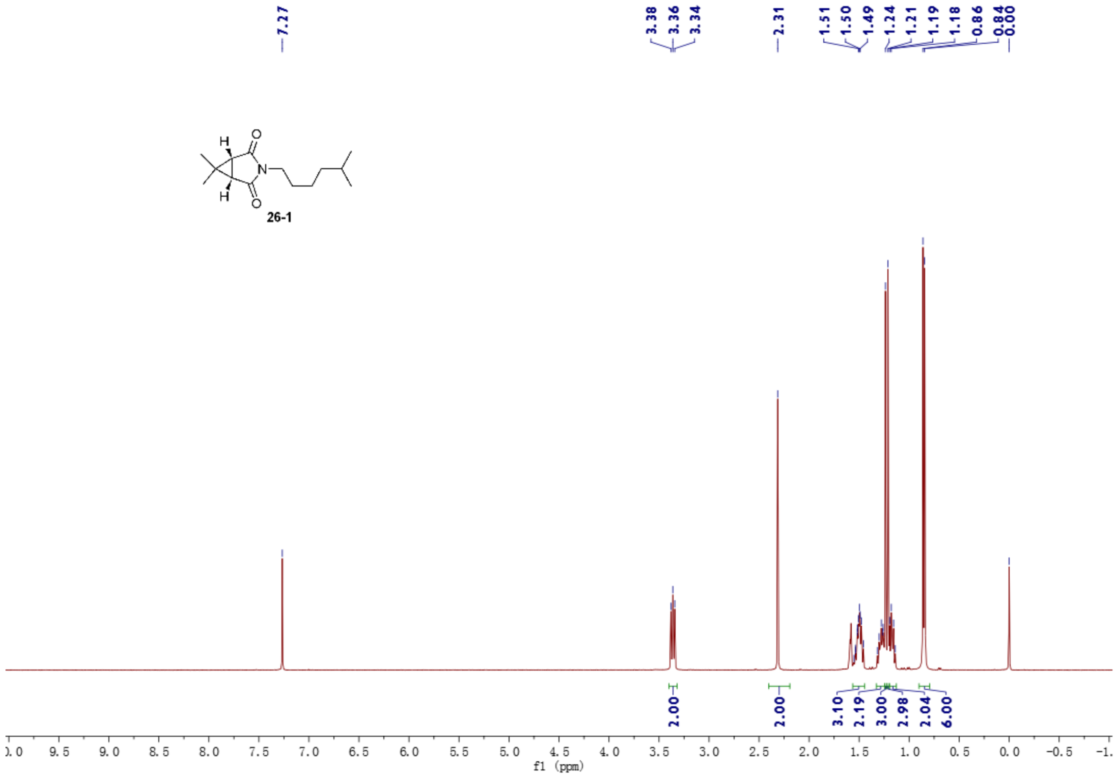
7.27
3.38
3.36
3.34
2.31
1.51
1.50
1.49
1.24
1.21
1.19
1.18
0.86
0.84
0.00
H
O
N
H
O
26-1
2.00
2.00
3.10
2.19
3.00
2.98
2.04
6.00
f1 (ppm)

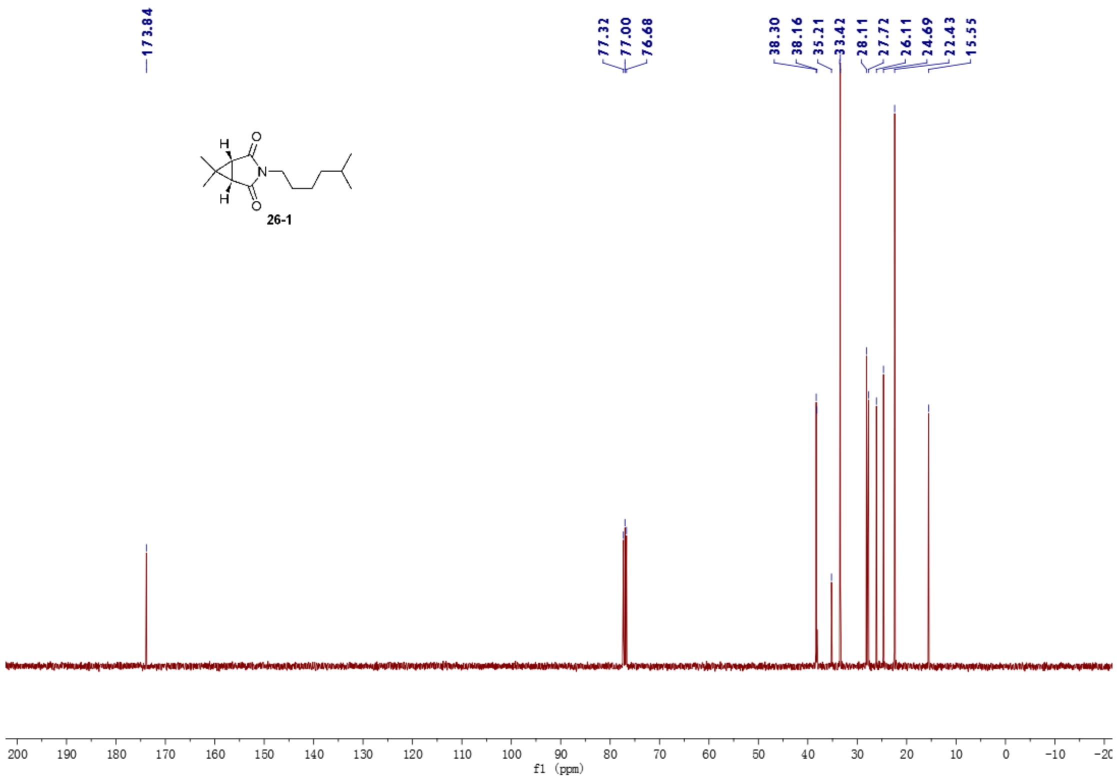
173.84
77.32
77.00
76.68
38.30
38.16
35.21
33.42
28.11
27.72
26.11
24.69
22.43
15.55
26-1
f1 (ppm)

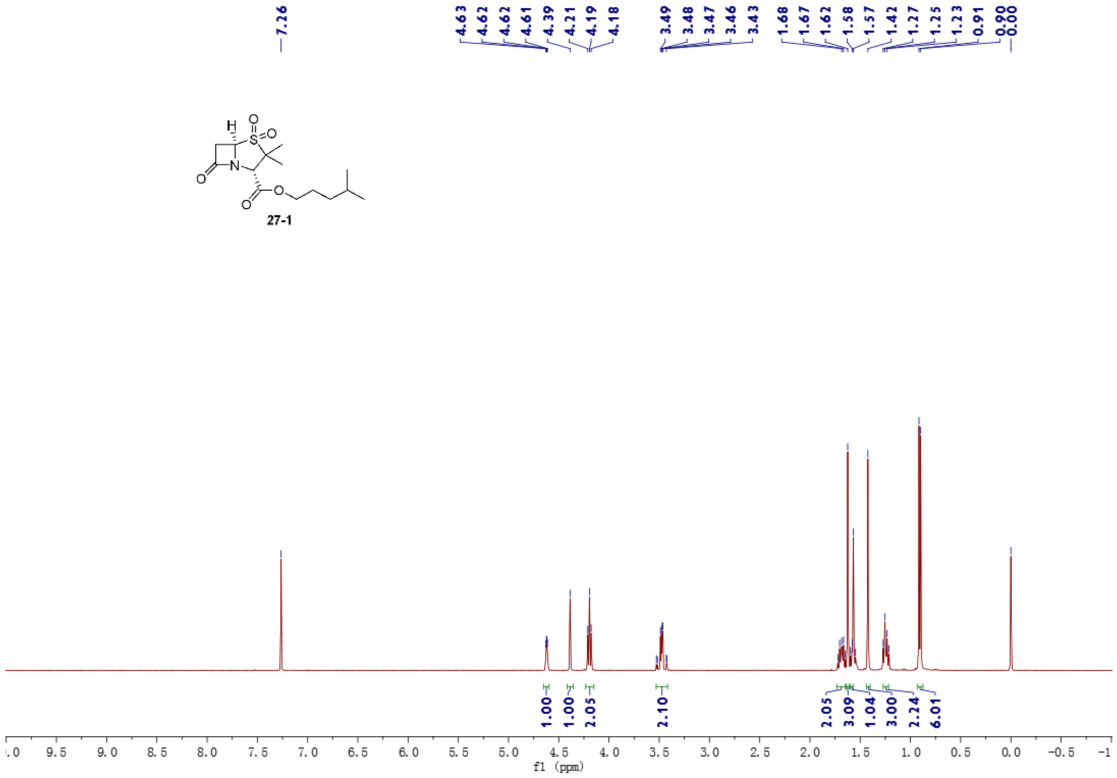
7.26
4.63
4.62
4.62
4.61
4.39
4.21
4.19
4.18
3.49
3.48
3.47
3.46
3.43
1.68
1.67
1.62
1.58
1.57
1.42
1.27
1.25
1.23
0.91
0.90
0.00
27-1
1.00
1.00
2.05
2.10
2.05
3.09
1.04
3.00
2.24
6.01
f1 (ppm)

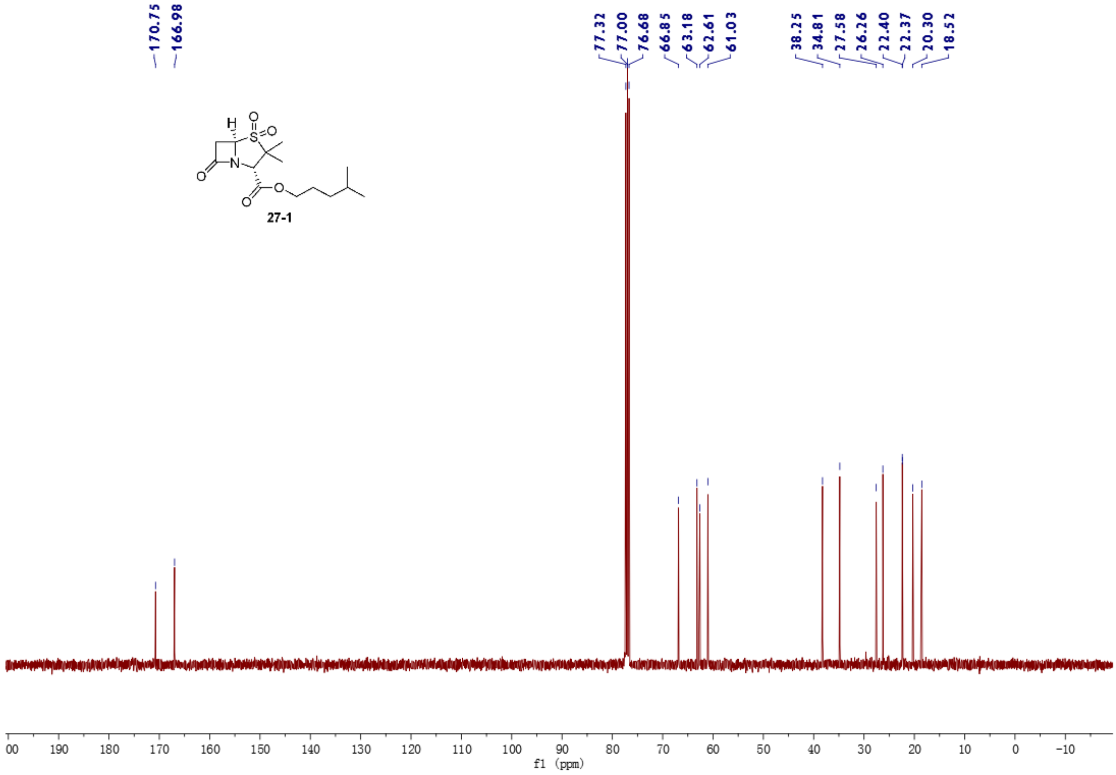
170.75
166.98
77.32
77.00
76.68
66.85
63.18
62.61
61.03
38.25
34.81
27.58
26.26
22.40
22.37
20.30
18.52
27-1
f1 (ppm)

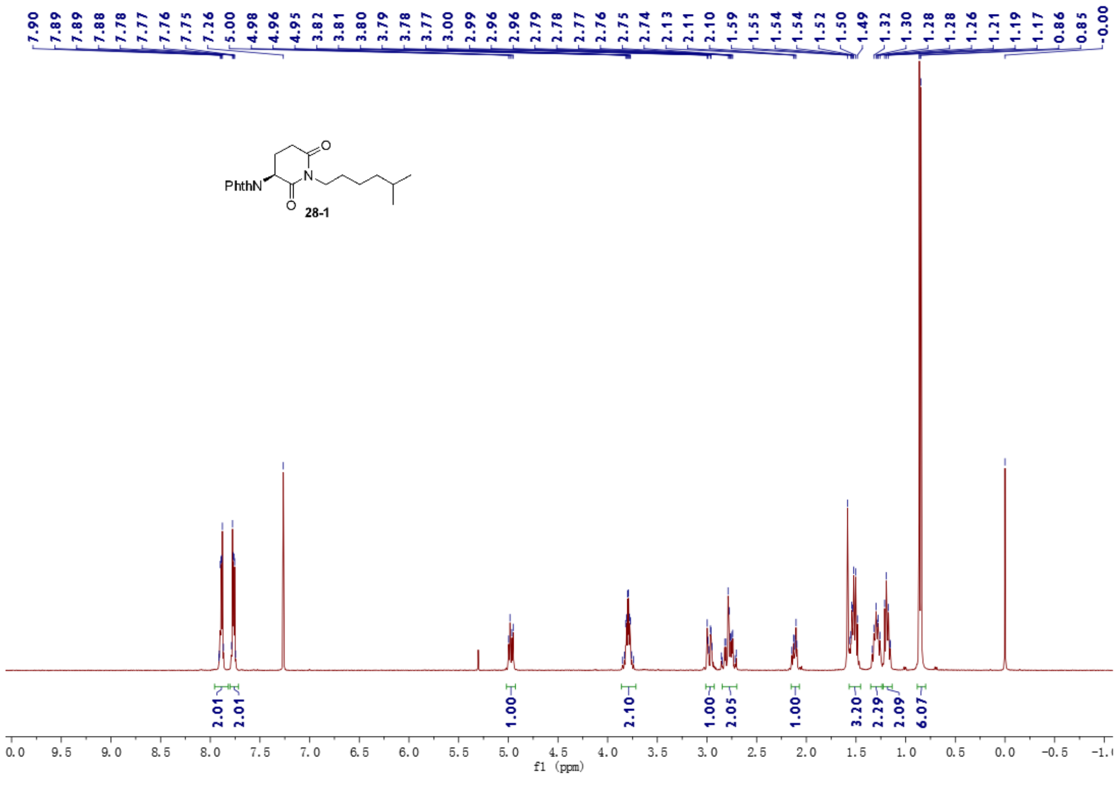
7.90
7.89
7.89
7.88
7.78
7.77
7.76
7.75
7.26
5.00
4.98
4.96
4.95
3.82
3.81
3.80
3.79
3.78
3.77
3.00
2.99
2.96
2.96
2.79
2.78
2.77
2.76
2.75
2.74
2.13
2.11
2.10
1.59
1.55
1.54
1.52
1.50
1.49
1.32
1.30
1.28
1.28
1.26
1.21
1.19
1.17
0.86
0.85
-0.00
PhthN
28-1
2.01
2.01
1.00
2.10
1.00
2.05
1.00
3.20
2.29
2.09
6.07
f1 (ppm)

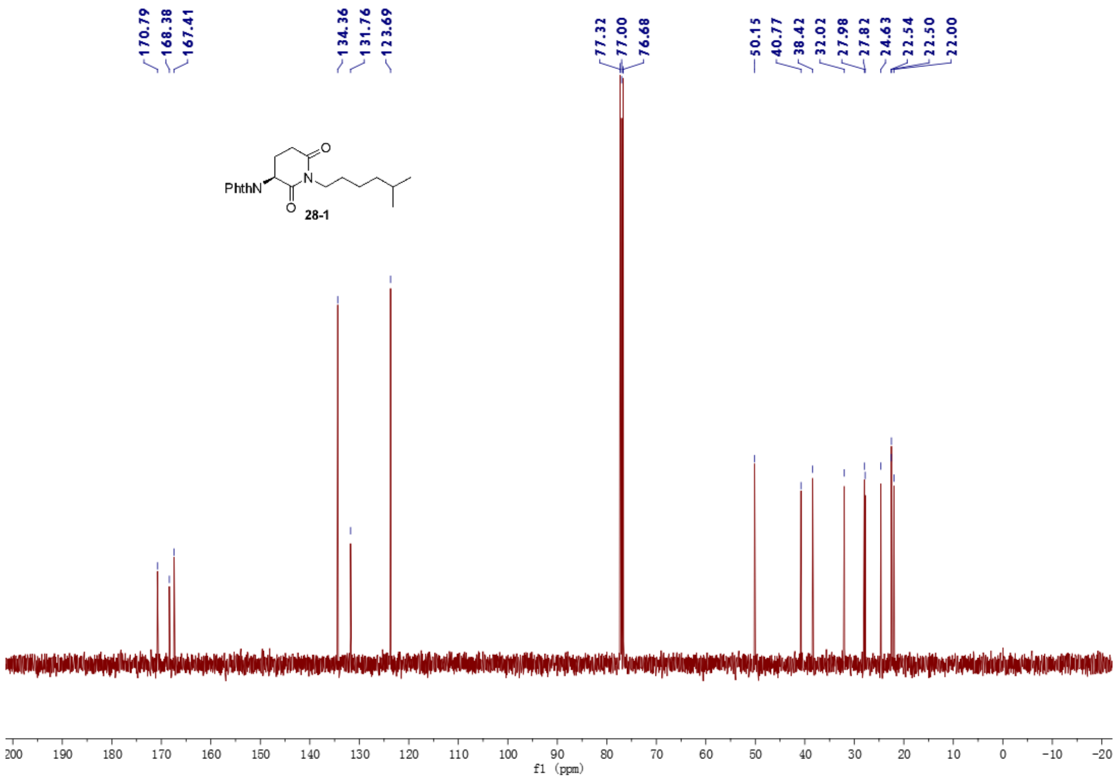
170.79
168.38
167.41
134.36
131.76
123.69
77.32
77.00
76.68
50.15
40.77
38.42
32.02
27.98
27.82
24.63
22.54
22.50
22.00
PhthN
28-1
f1 (ppm)

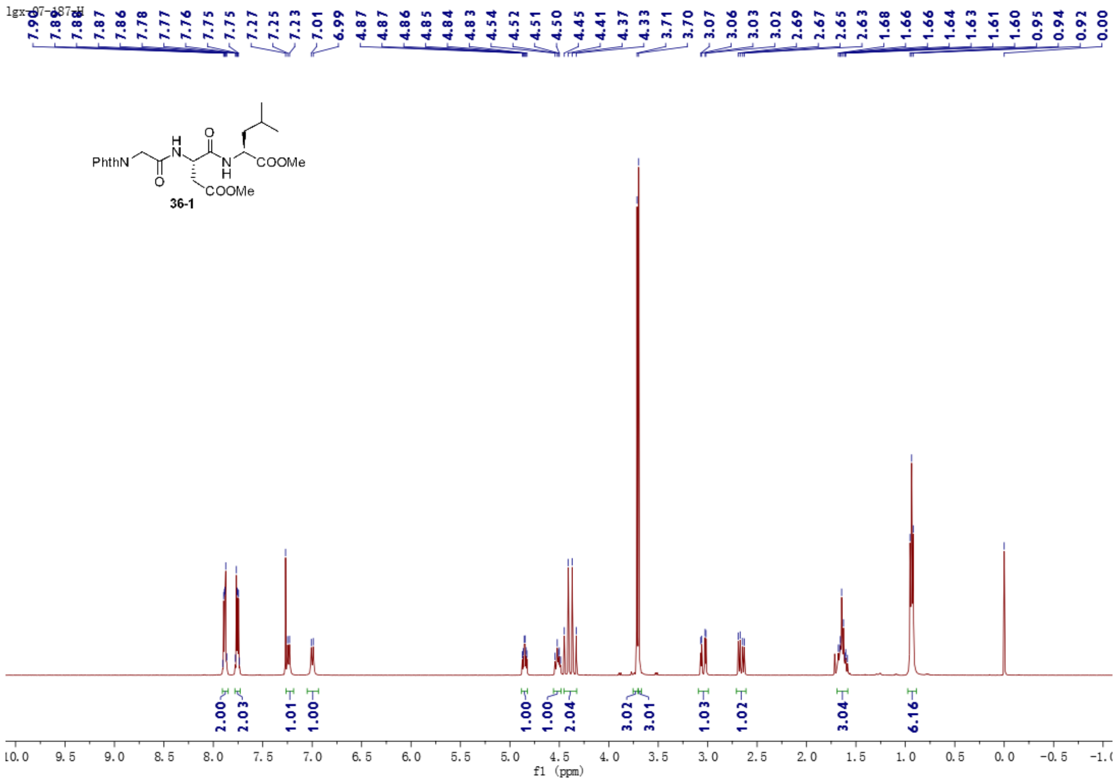
PhthN
COOMe
COOMe
36-1
2.00
2.03
1.01
1.00
1.00
1.00
2.04
3.02
3.01
1.03
1.02
3.04
6.16
f1 (ppm)

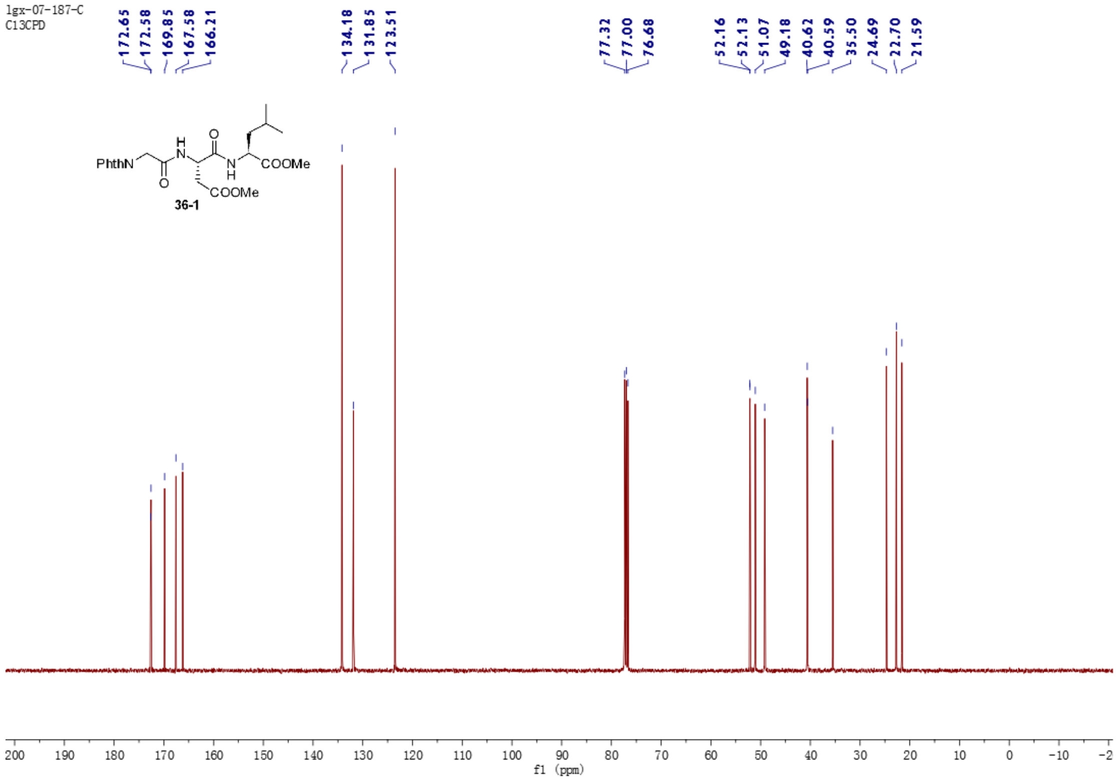

lgx-07-187-C
C13CPD
172.65
172.58
169.85
167.58
166.21
134.18
131.85
123.51
77.32
77.00
76.68
52.16
52.13
51.07
49.18
40.62
40.59
35.50
24.69
22.70
21.59
PhthN
H
N
O
COOMe
COOMe
36-1
f1 (ppm)

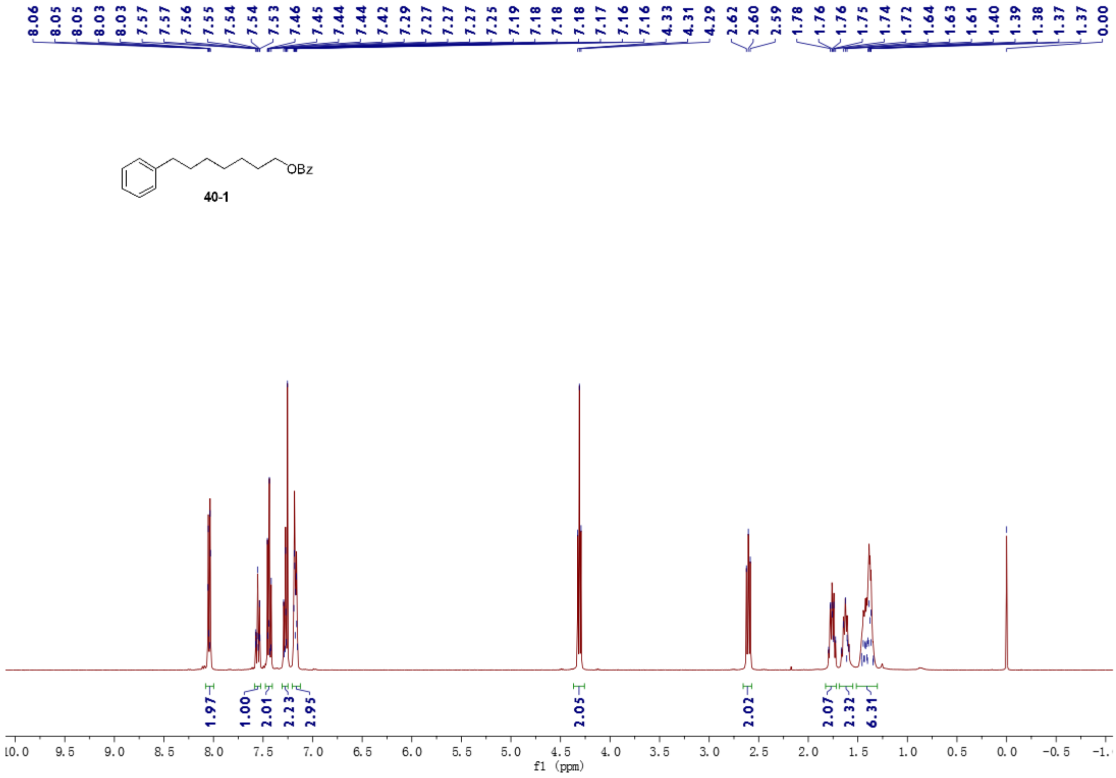

8.06
8.05
8.05
8.03
8.03
7.57
7.56
7.55
7.54
7.54
7.53
7.46
7.45
7.44
7.44
7.42
7.29
7.27
7.27
7.27
7.25
7.19
7.18
7.18
7.17
7.16
7.16
4.33
4.31
4.29
2.62
2.60
2.59
1.78
1.76
1.76
1.75
1.74
1.72
1.64
1.63
1.61
1.40
1.39
1.38
1.37
1.37
0.00
OBz
40-1
1.97
1.00
2.01
2.23
2.95
2.05
2.02
2.07
2.32
6.31
f1 (ppm)

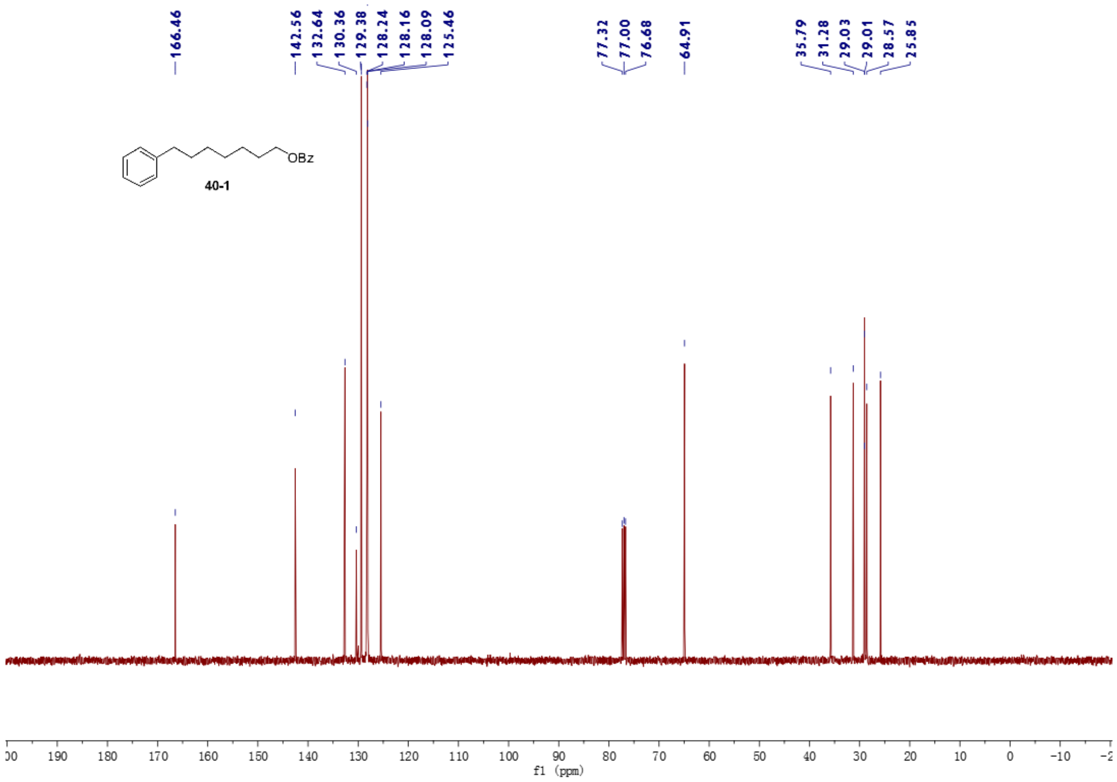

166.46
142.56
132.64
130.36
129.38
128.24
128.16
128.09
125.46
77.32
77.00
76.68
64.91
35.79
31.28
29.03
29.01
28.57
25.85
OBz
40-1
f1 (ppm)

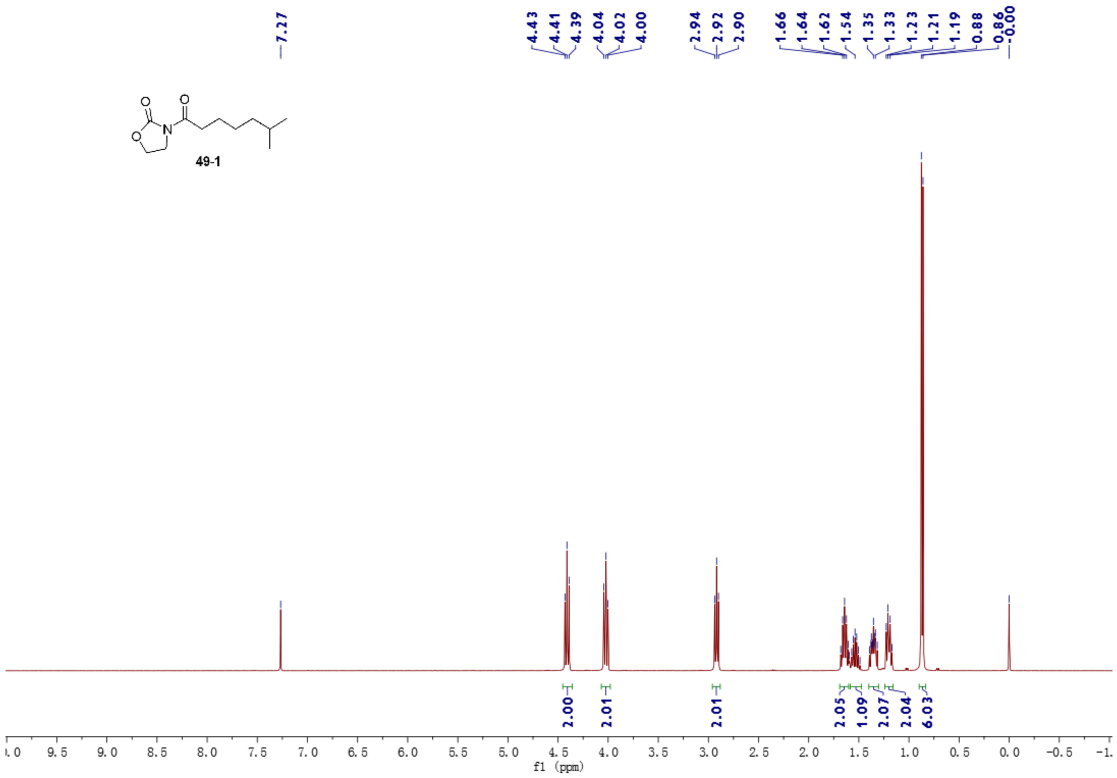

7.27
4.43
4.41
4.39
4.04
4.02
4.00
2.94
2.92
2.90
1.66
1.64
1.62
1.54
1.35
1.33
1.23
1.21
1.19
0.88
0.86
0.00
O
O
N
O
49-1
2.00
2.01
2.01
2.05
1.09
2.07
2.04
6.03
f1 (ppm)

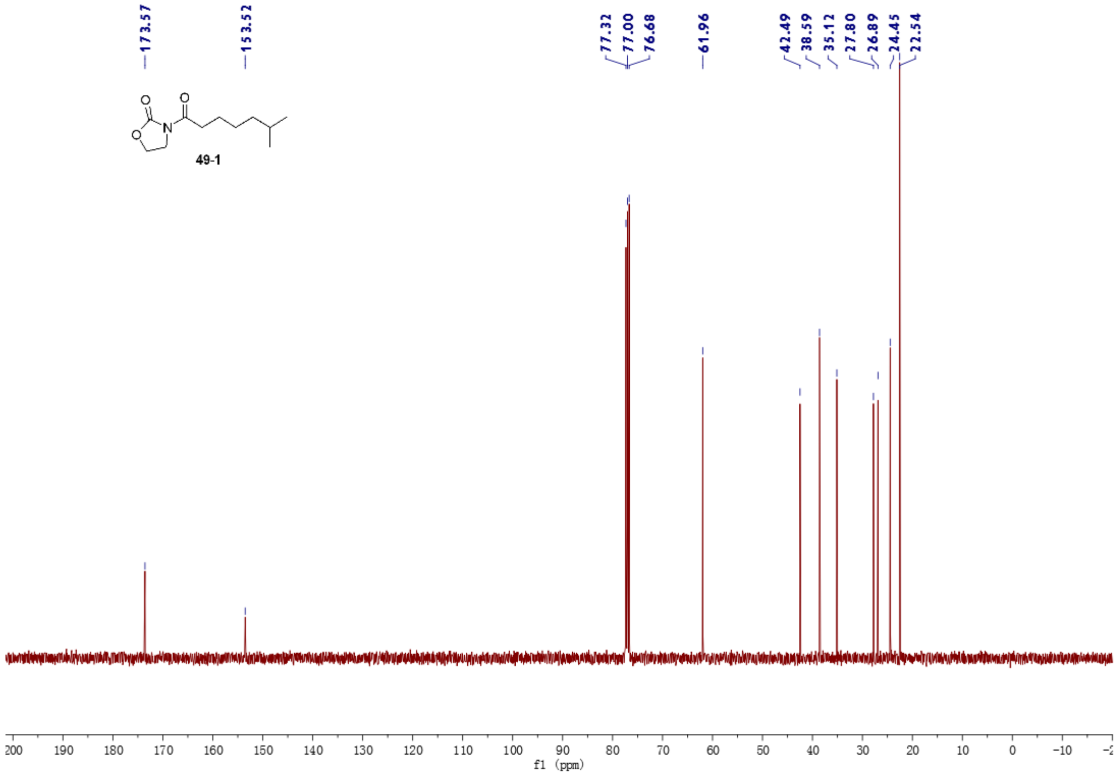
173.57
153.52
77.32
77.00
76.68
61.96
42.49
38.59
35.12
27.80
26.89
24.45
22.54
49-1
f1 (ppm)

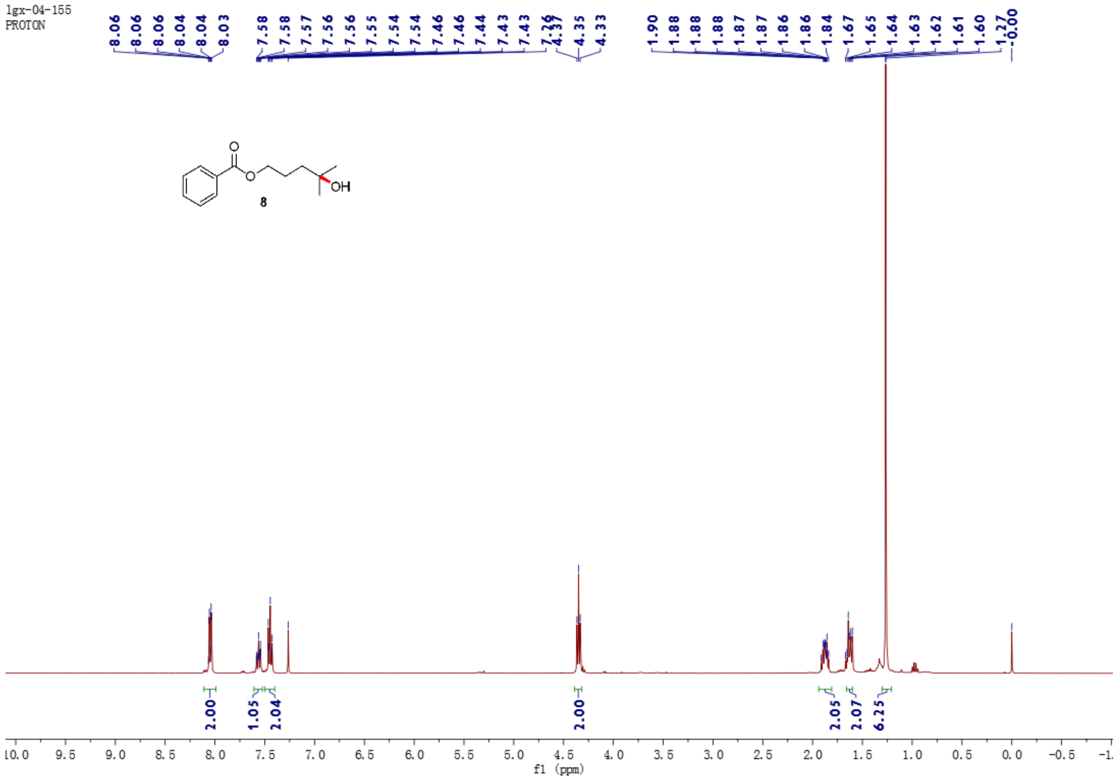
lgx-04-155
PROTON
8.06 8.06 8.06 8.04 8.04 8.03
7.58 7.58 7.57 7.56 7.56 7.55 7.54 7.46 7.46 7.44 7.43 7.43 7.26
4.37 4.35 4.33
1.90 1.88 1.88 1.87 1.87 1.86 1.86 1.84
1.67 1.65 1.64 1.63 1.62 1.61 1.60
1.27
0.00
OH
8
2.00
1.05
2.04
2.00
2.05
2.07
6.25
f1 (ppm)

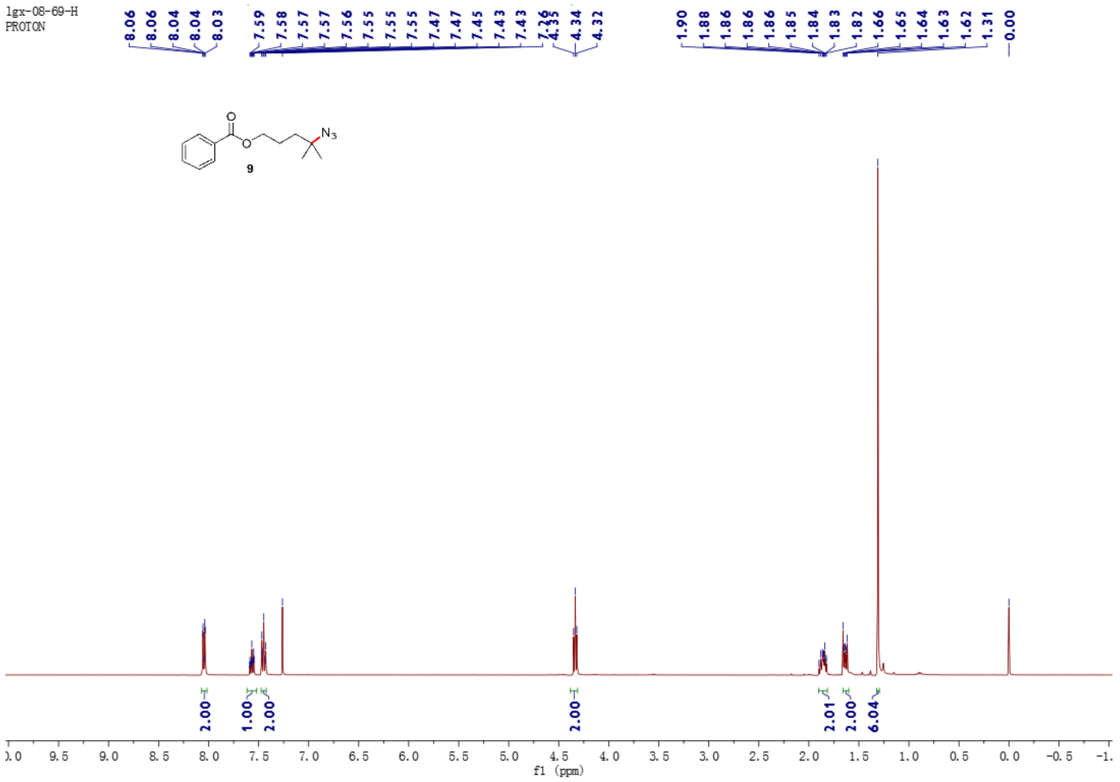
lgx-08-69-H
PROTON
8.06 8.06 8.04 8.04 8.03 7.59 7.58 7.57 7.57 7.56 7.55 7.55 7.55 7.47 7.47 7.45 7.43 7.43 7.26 4.35 4.34 4.32 1.90 1.88 1.86 1.86 1.86 1.85 1.84 1.83 1.82 1.66 1.65 1.64 1.63 1.62 1.31 0.00
N3
9
2.00 1.00 2.00 2.00 2.01 2.00 6.04
f1 (ppm)

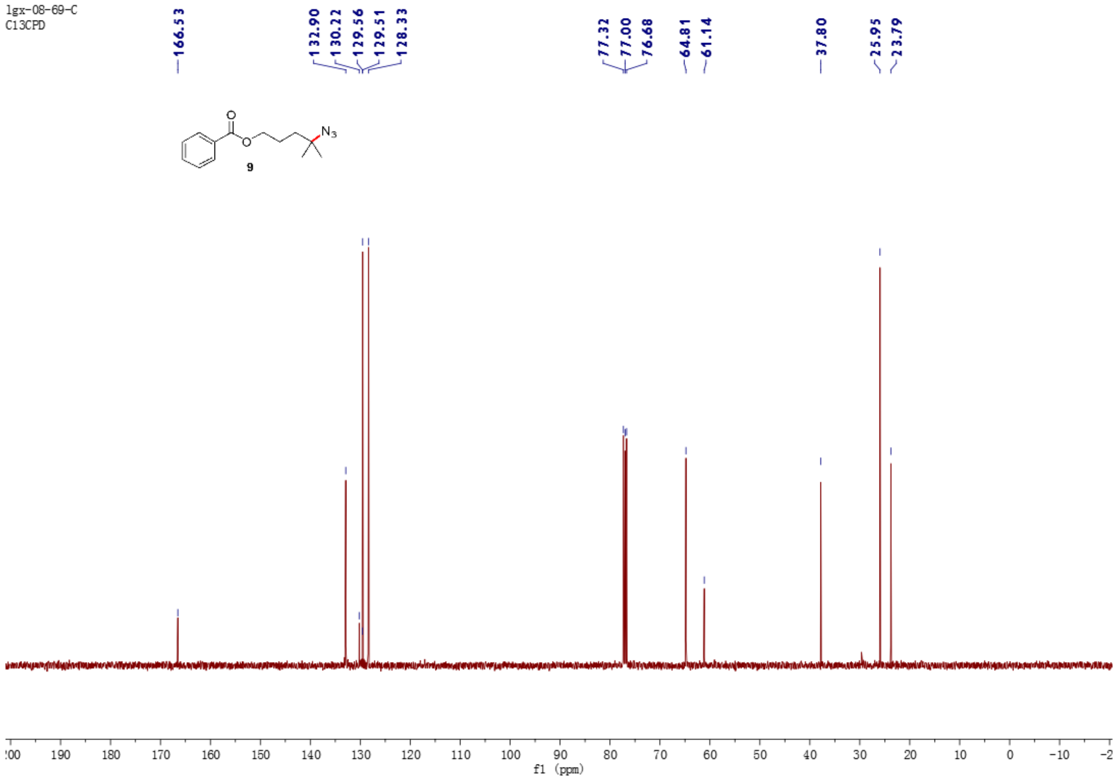
lgx-08-69-C
C13CPD
166.53 132.90 130.22 129.56 129.51 128.33 77.32 77.00 76.68 64.81 61.14 37.80 25.95 23.79
N3
9
f1 (ppm)

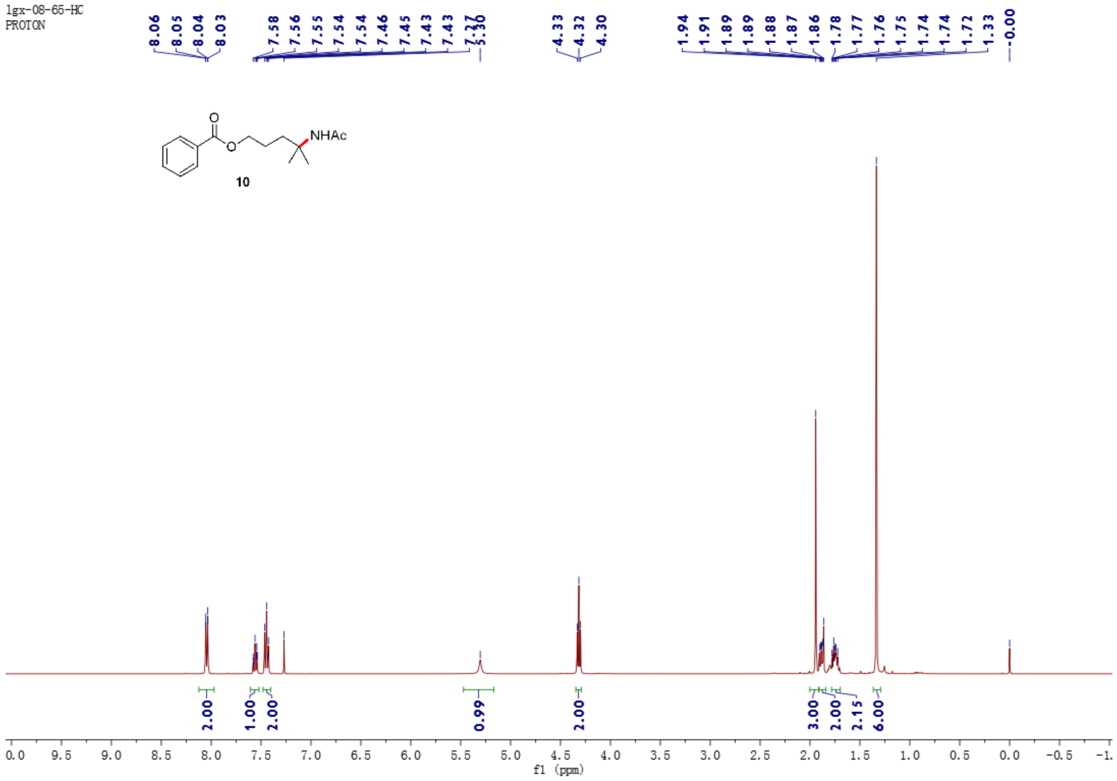
lgx-08-65-HC
PROTON
8.06 8.05 8.04 8.03 7.58 7.56 7.55 7.54 7.54 7.46 7.45 7.43 7.43 7.27 5.30
4.33 4.32 4.30
1.94 1.91 1.89 1.89 1.88 1.87 1.86 1.78 1.77 1.76 1.75 1.74 1.74 1.72 1.33 -0.00
NHAc
10
2.00 1.00 2.00 0.99 2.00 3.00 2.00 2.15 6.00
f1 (ppm)

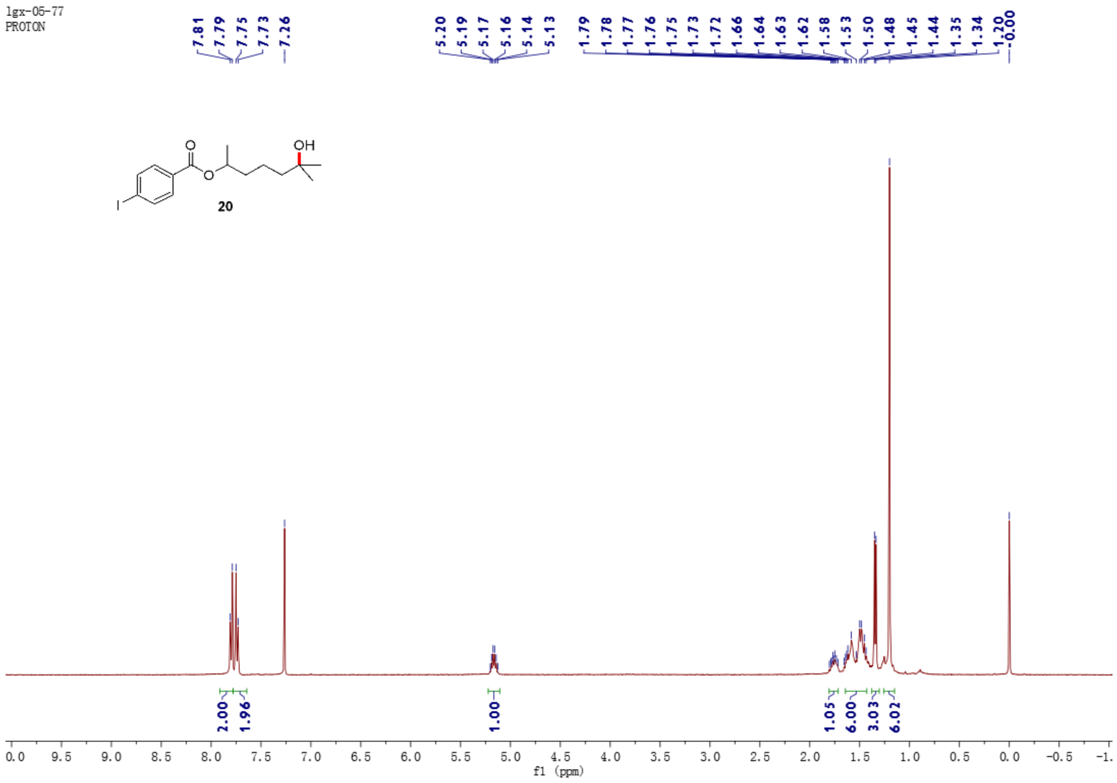
lgx-05-77
PROTON
7.81 7.79 7.75 7.73 7.26
5.20 5.19 5.17 5.16 5.14 5.13
1.79 1.78 1.77 1.76 1.75 1.73 1.72 1.66 1.64 1.63 1.62 1.58 1.53 1.50 1.48 1.45 1.44 1.35 1.34 1.20 0.00
OH
I
20
2.00 1.96 1.00 1.05 6.00 3.03 6.02
f1 (ppm)

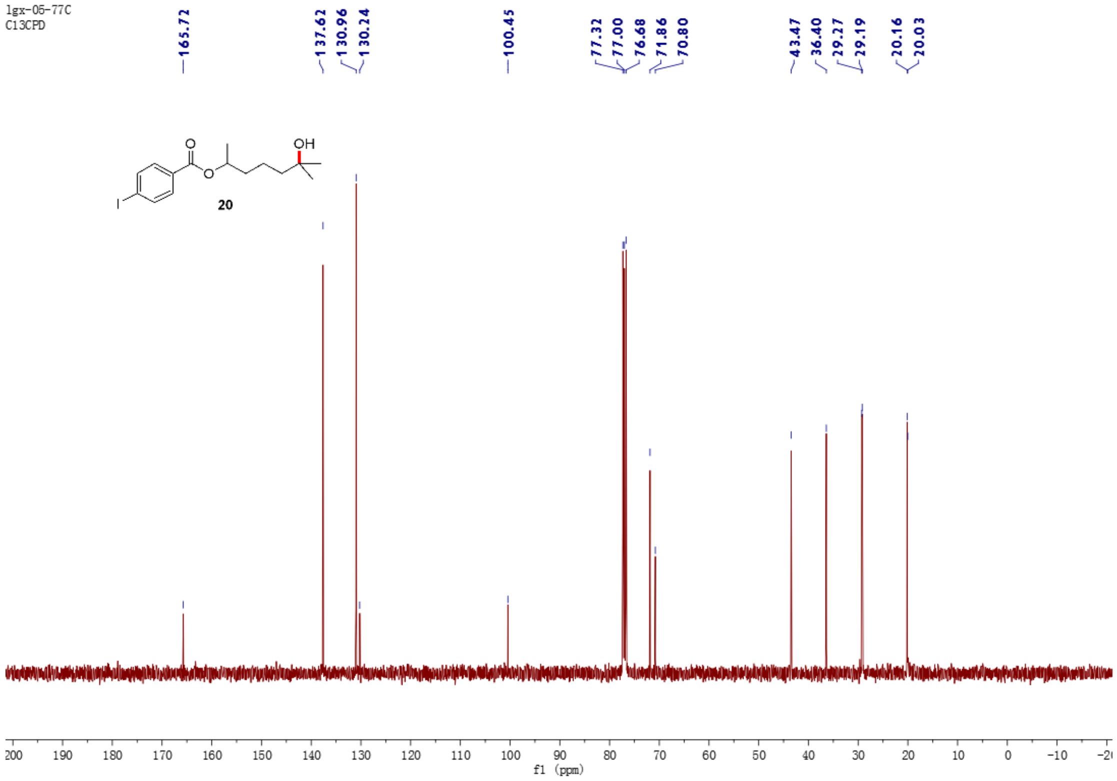
lgx-05-77C
C13CPD
165.72
137.62
130.96
130.24
100.45
77.32
77.00
76.68
71.86
70.80
43.47
36.40
29.27
29.19
20.16
20.03
OH
O
O
I
20
f1 (ppm)

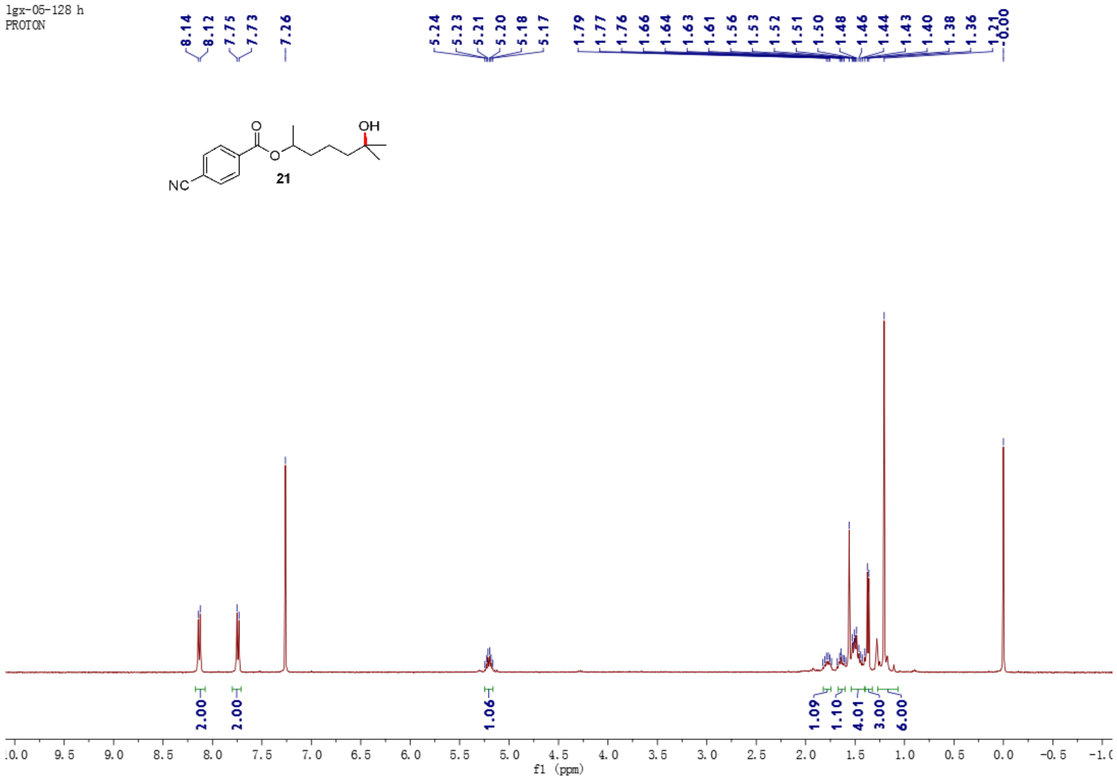
lgx-05-128 h
PROTON
8.14
8.12
7.75
7.73
7.26
5.24
5.23
5.21
5.20
5.18
5.17
1.79
1.77
1.76
1.66
1.64
1.63
1.61
1.56
1.53
1.52
1.51
1.50
1.48
1.46
1.44
1.43
1.40
1.38
1.36
1.21
0.00
OH
O
O
NC
21
2.00
2.00
1.06
1.09
1.10
4.01
3.00
6.00
f1 (ppm)

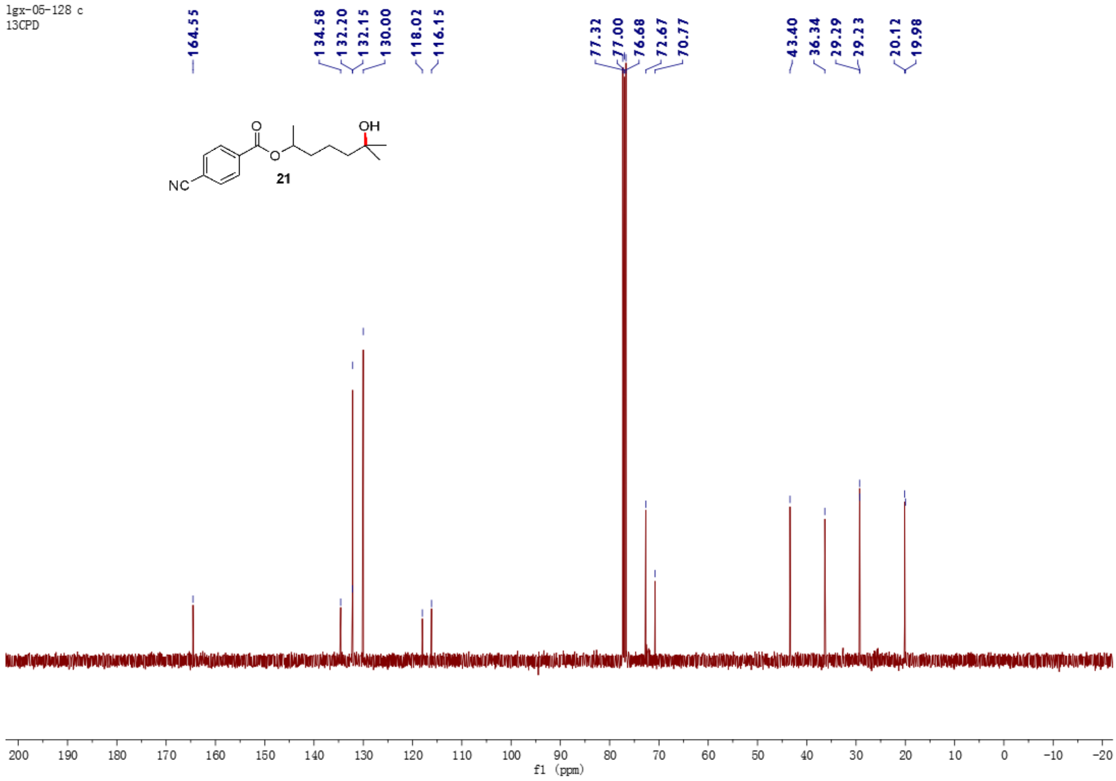
lgx-05-128 c
13CPD
164.55
134.58
132.20
132.15
130.00
118.02
116.15
77.32
77.00
76.68
72.67
70.77
43.40
36.34
29.29
29.23
20.12
19.98
OH
O
O
NC
21
200 190 180 170 160 150 140 130 120 110 100 90 80 70 60 50 40 30 20 10 0 -10 -20
f1 (ppm)

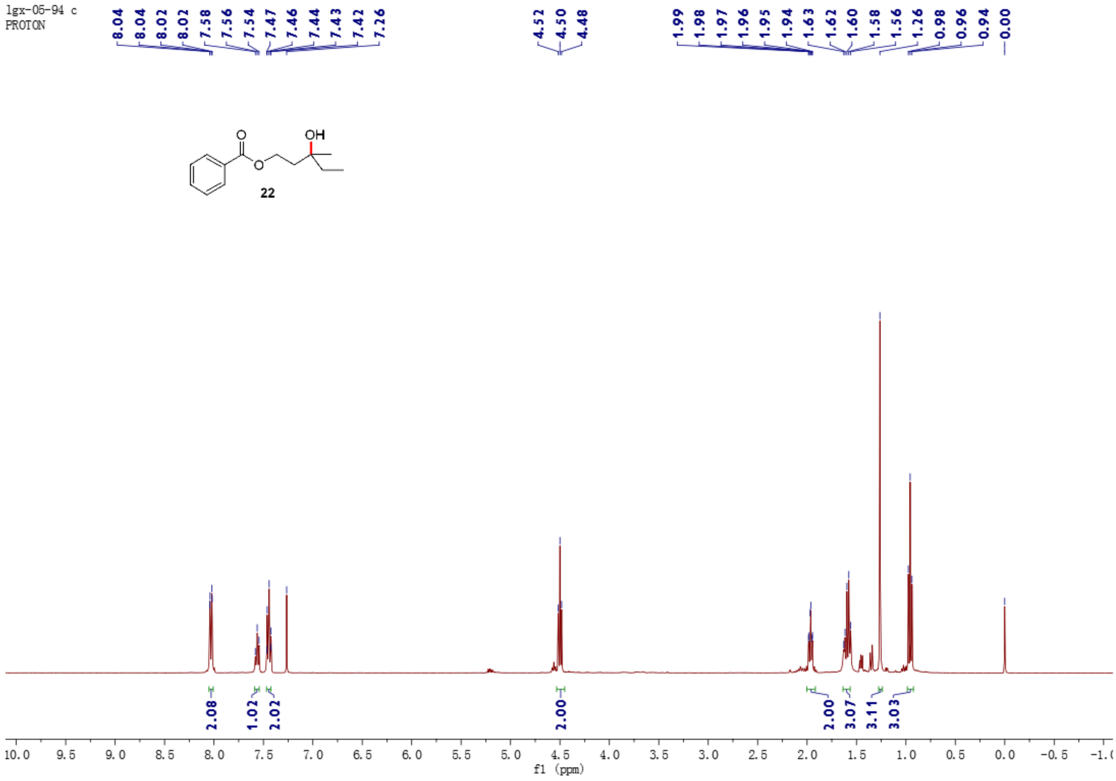
lgx-05-94 c
PROTON
8.04
8.04
8.02
8.02
7.58
7.56
7.54
7.47
7.46
7.44
7.43
7.42
7.26
4.52
4.50
4.48
1.99
1.98
1.97
1.96
1.95
1.94
1.63
1.62
1.60
1.58
1.56
1.26
0.98
0.96
0.94
0.00
O
OH
O
22
2.08
1.02
2.02
2.00
2.00
3.07
3.11
3.03
10.0 9.5 9.0 8.5 8.0 7.5 7.0 6.5 6.0 5.5 5.0 4.5 4.0 3.5 3.0 2.5 2.0 1.5 1.0 0.5 0.0 -0.5
f1 (ppm)

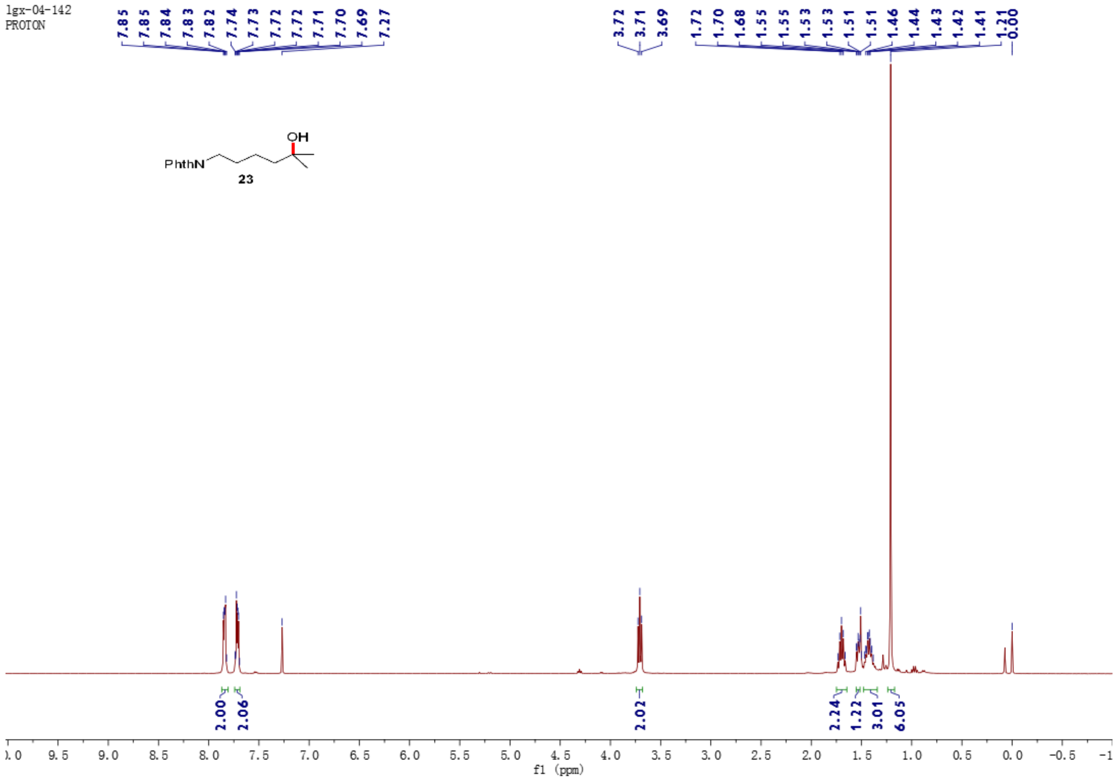
lgx-04-142
PROTON
PhthN
OH
23
f1 (ppm)

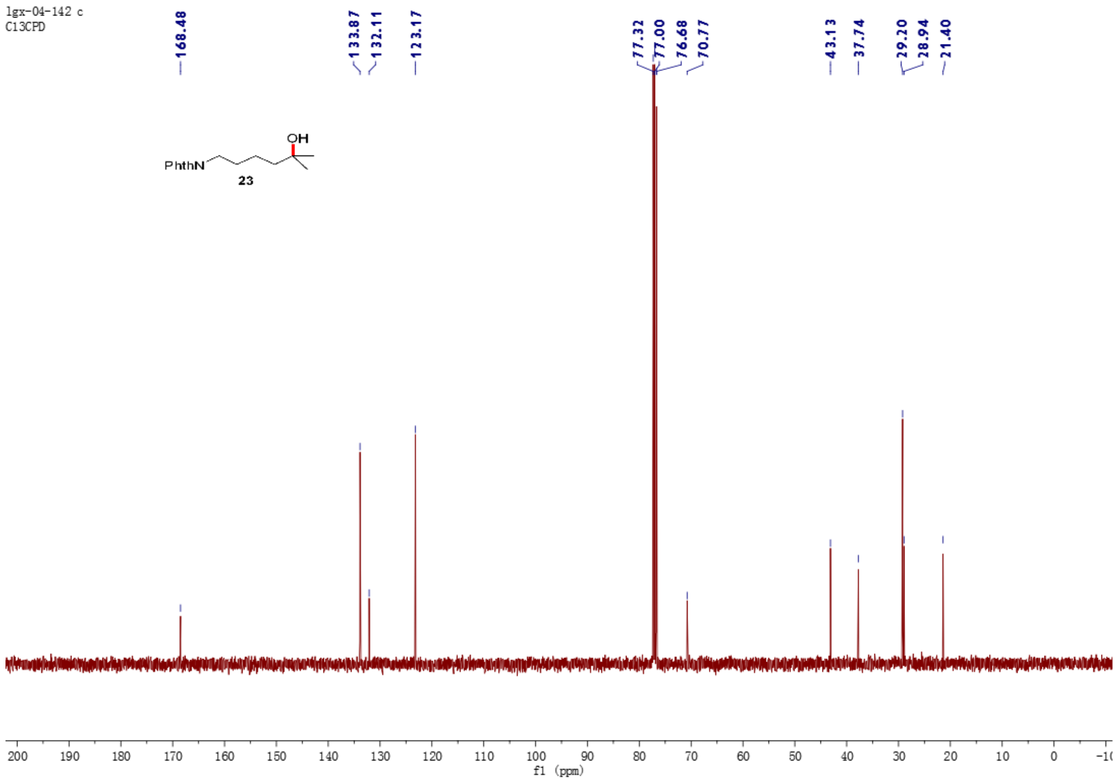
lgx-04-142 c
C13CPD
PhthN
OH
23
f1 (ppm)

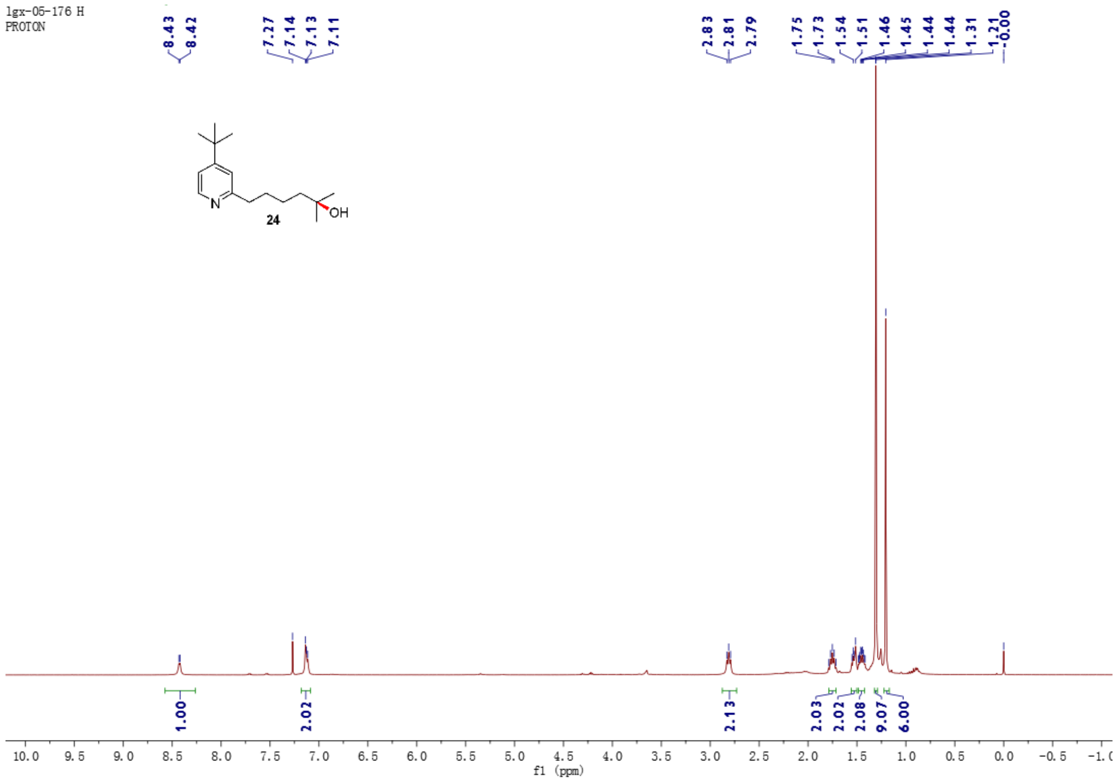
lgx-05-176 H
PROTON
8.43
8.42
7.27
7.14
7.13
7.11
2.83
2.81
2.79
1.75
1.73
1.54
1.51
1.46
1.45
1.44
1.44
1.31
1.21
0.00
N
OH
24
1.00
2.02
2.13
2.03
2.02
2.08
9.07
6.00
f1 (ppm)

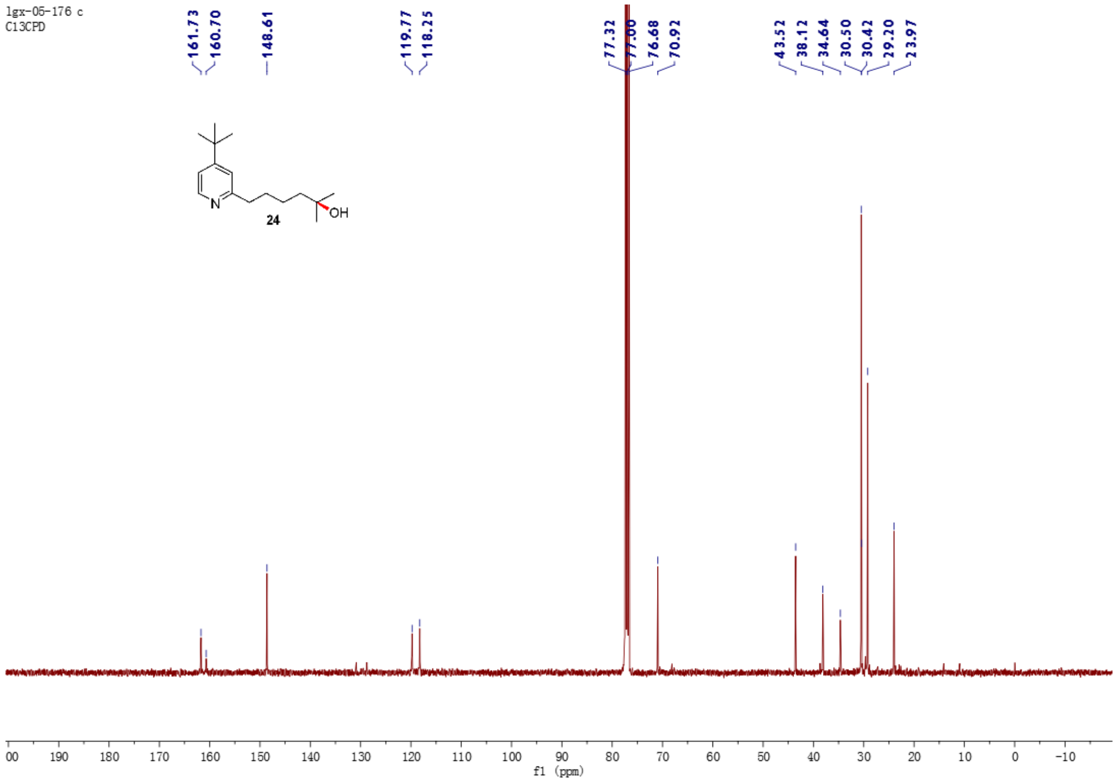
lgx-05-176 c
C13CPD
161.73
160.70
148.61
119.77
118.25
77.32
77.00
76.68
70.92
43.52
38.12
34.64
30.50
30.42
29.20
23.97
N
OH
24
f1 (ppm)

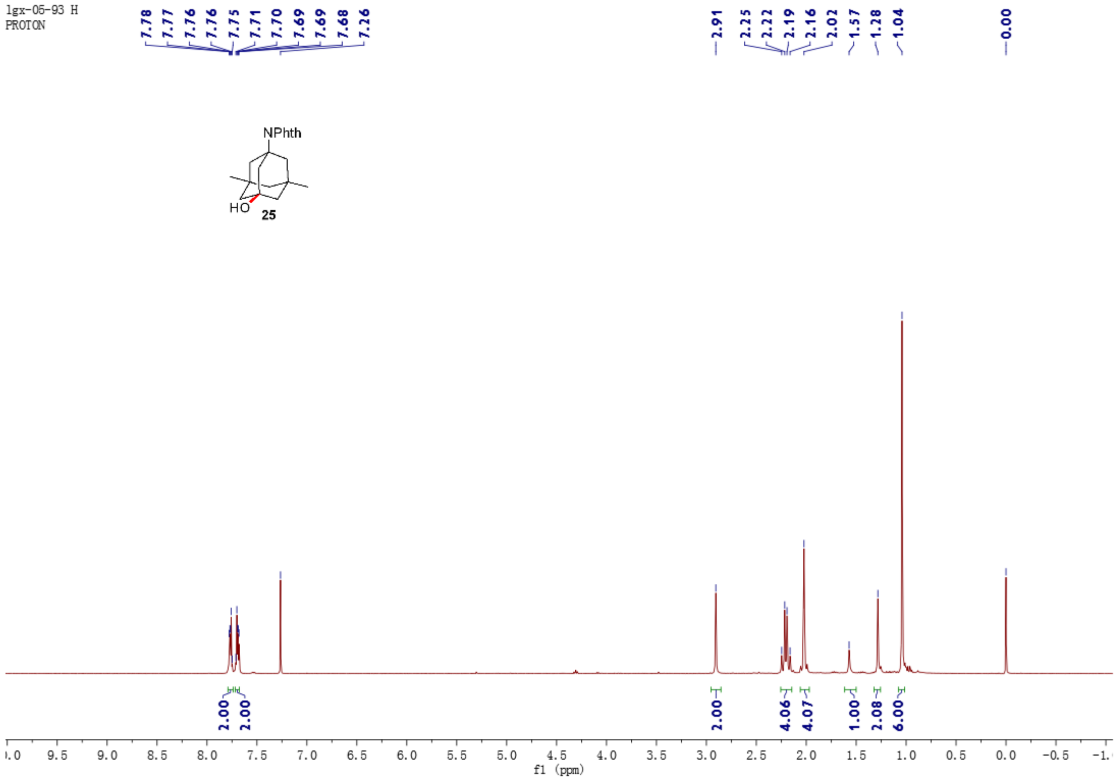

lgx-05-93 H
PROTON
7.78
7.77
7.76
7.76
7.75
7.71
7.70
7.69
7.69
7.68
7.26
2.91
2.25
2.22
2.19
2.16
2.02
1.57
1.28
1.04
0.00
NPhth
HO
25
2.00
2.00
2.00
4.06
4.07
1.00
2.08
6.00
f1 (ppm)

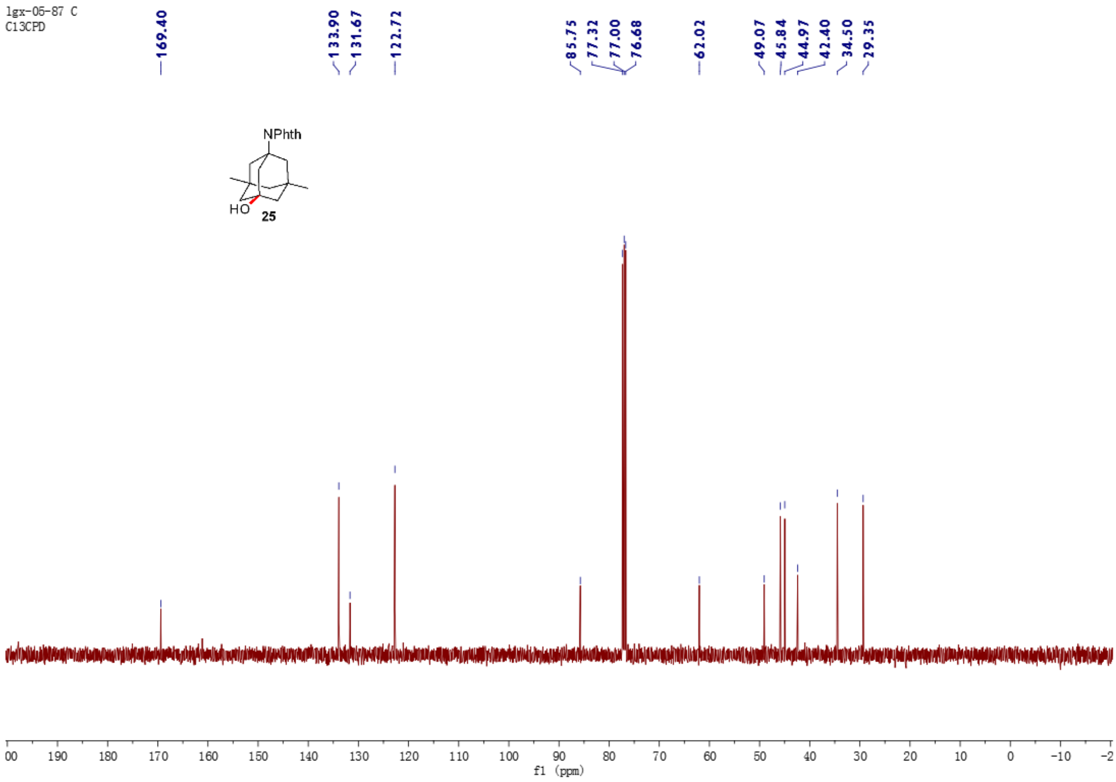

lgx-05-87 C
C13CPD
169.40
133.90
131.67
122.72
85.75
77.32
77.00
76.68
62.02
49.07
45.84
44.97
42.40
34.50
29.35
NPhth
HO
25
f1 (ppm)

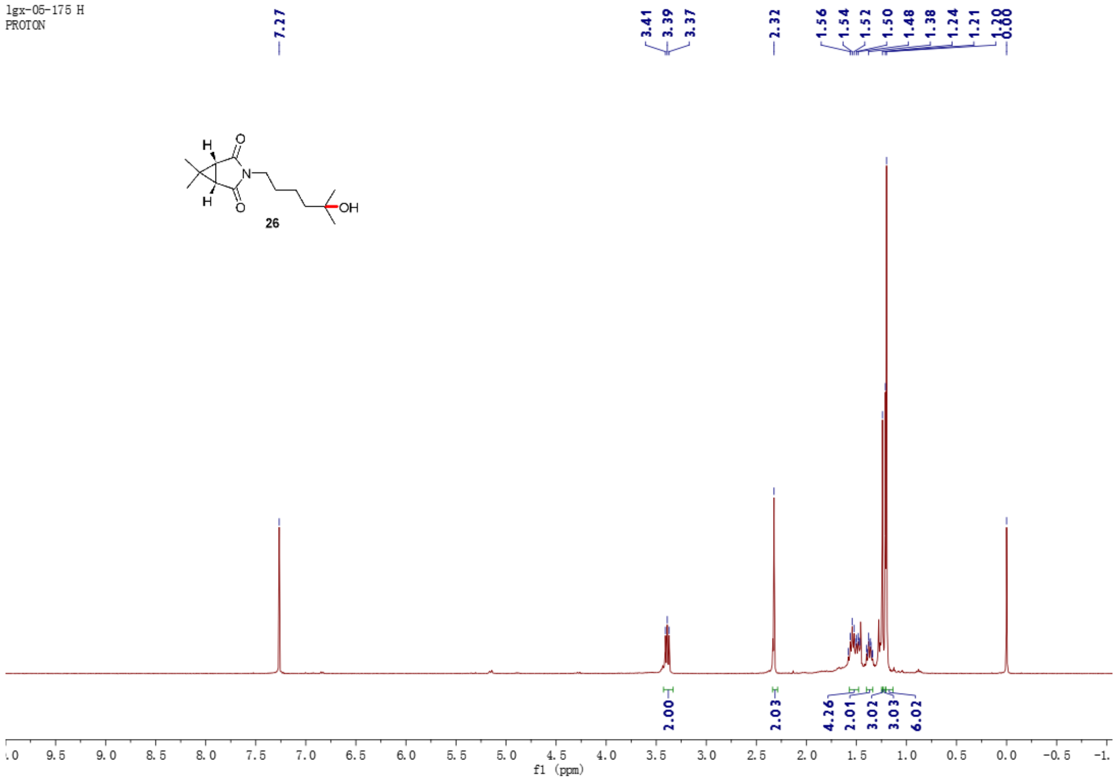
lgx-05-175 H
PROTON
7.27
3.41
3.39
3.37
2.32
1.56
1.54
1.52
1.50
1.48
1.38
1.24
1.21
1.20
0.00
26
2.00
2.03
4.26
2.01
3.02
3.03
6.02
f1 (ppm)

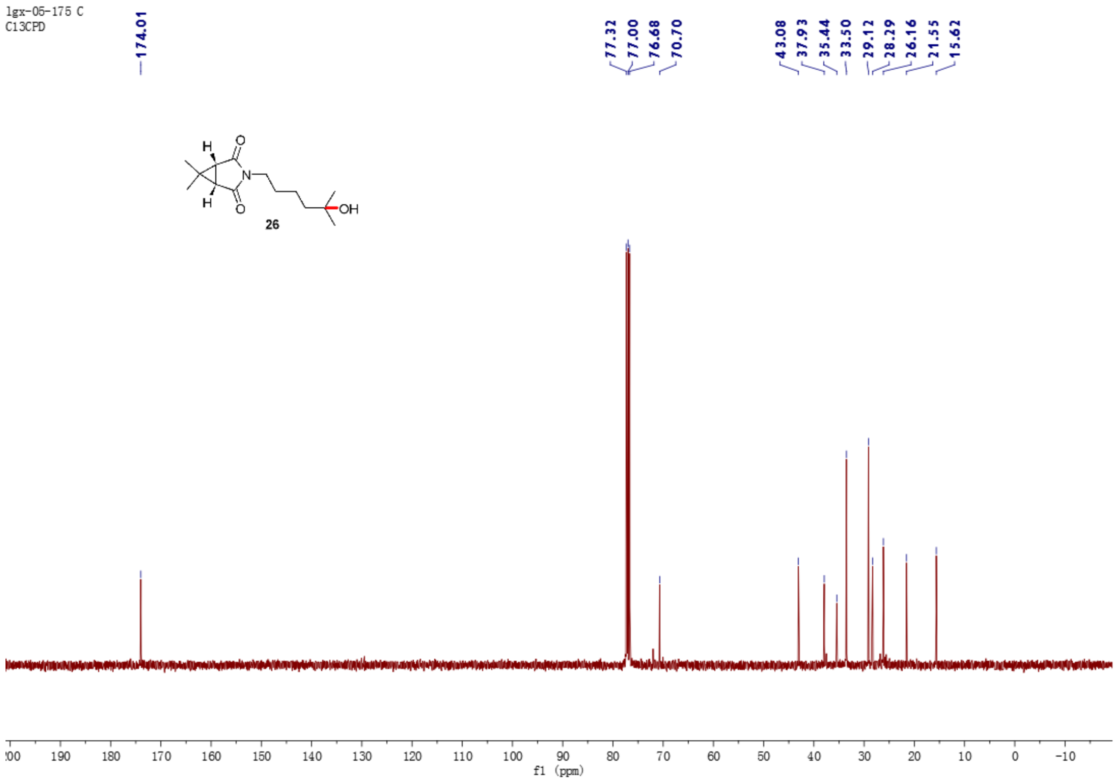
lgx-05-175 C
C13CPD
174.01
77.32
77.00
76.68
70.70
43.08
37.93
35.44
33.50
29.12
28.29
26.16
21.55
15.62
26
f1 (ppm)

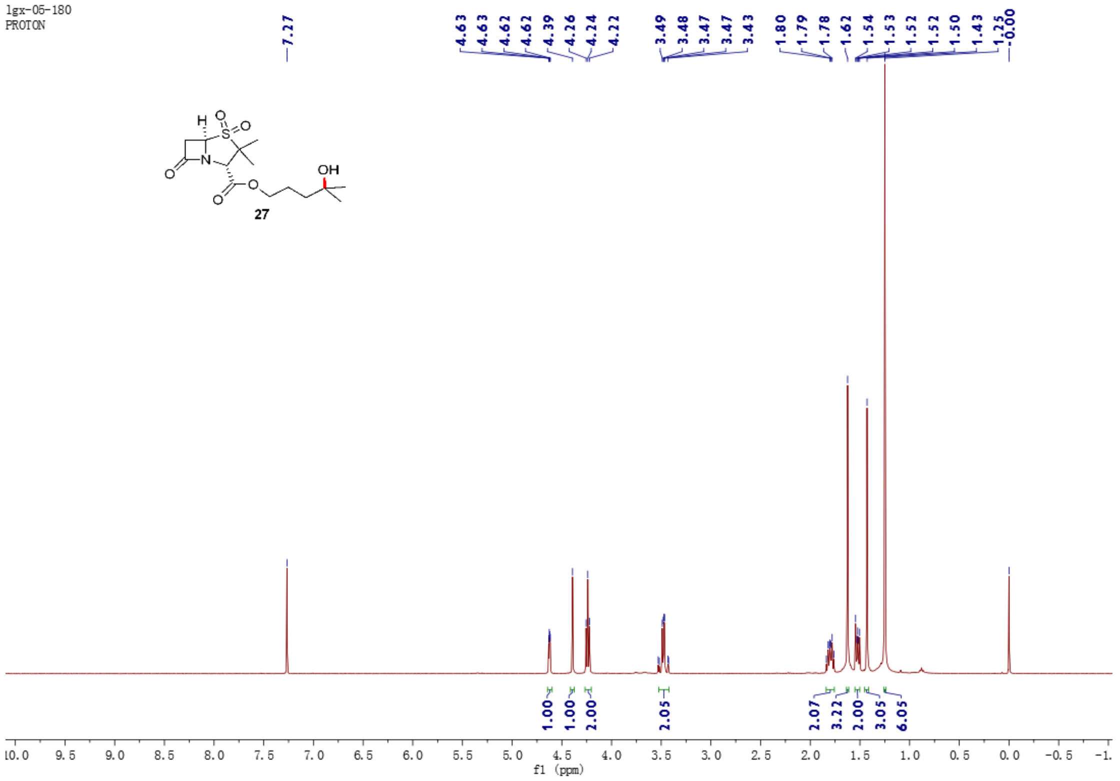

lgx-05-180
PROTON
7.27
4.63
4.63
4.62
4.62
4.39
4.26
4.24
4.22
3.49
3.48
3.47
3.47
3.43
1.80
1.79
1.78
1.62
1.54
1.53
1.52
1.52
1.50
1.43
1.25
0.00
OH
27
1.00
1.00
2.00
2.05
2.07
3.22
2.00
3.05
6.05
f1 (ppm)

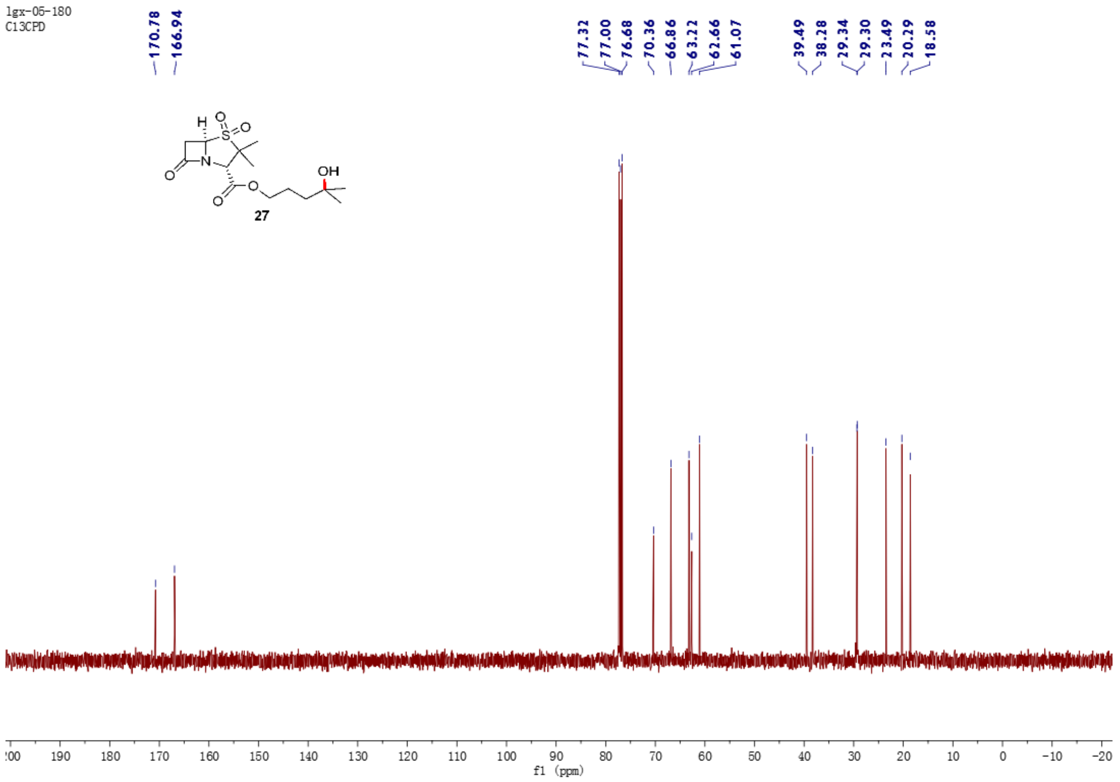

lgx-05-180
C13CPD
170.78
166.94
77.32
77.00
76.68
70.36
66.86
63.22
62.66
61.07
39.49
38.28
29.34
29.30
23.49
20.29
18.58
OH
27
f1 (ppm)

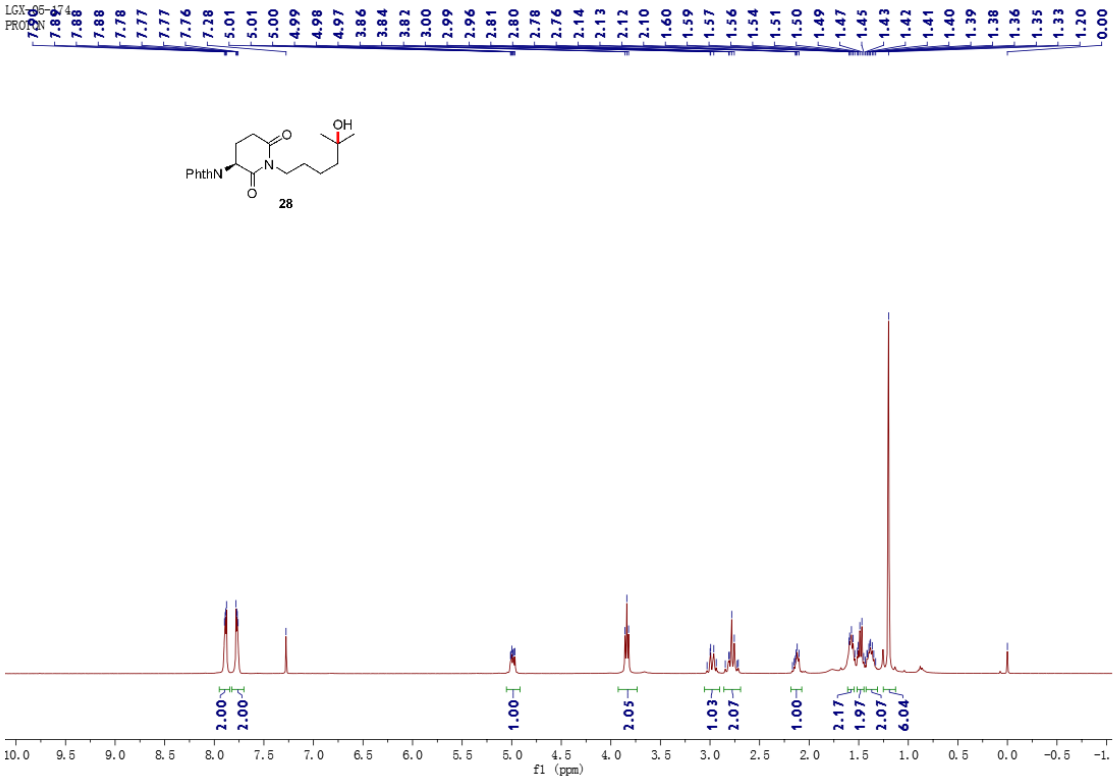

LGX-05-174
PROTON
7.89
7.88
7.88
7.78
7.77
7.77
7.76
7.28
5.01
5.01
5.00
4.99
4.98
4.97
3.86
3.84
3.82
3.00
2.99
2.96
2.81
2.80
2.78
2.76
2.14
2.13
2.12
2.10
1.60
1.59
1.57
1.56
1.54
1.51
1.50
1.49
1.47
1.45
1.43
1.42
1.41
1.40
1.39
1.38
1.36
1.35
1.33
1.20
0.00
OH
PhthN
N
O
28
2.00
2.00
1.00
2.05
1.03
2.07
1.00
2.17
1.97
2.07
6.04
f1 (ppm)

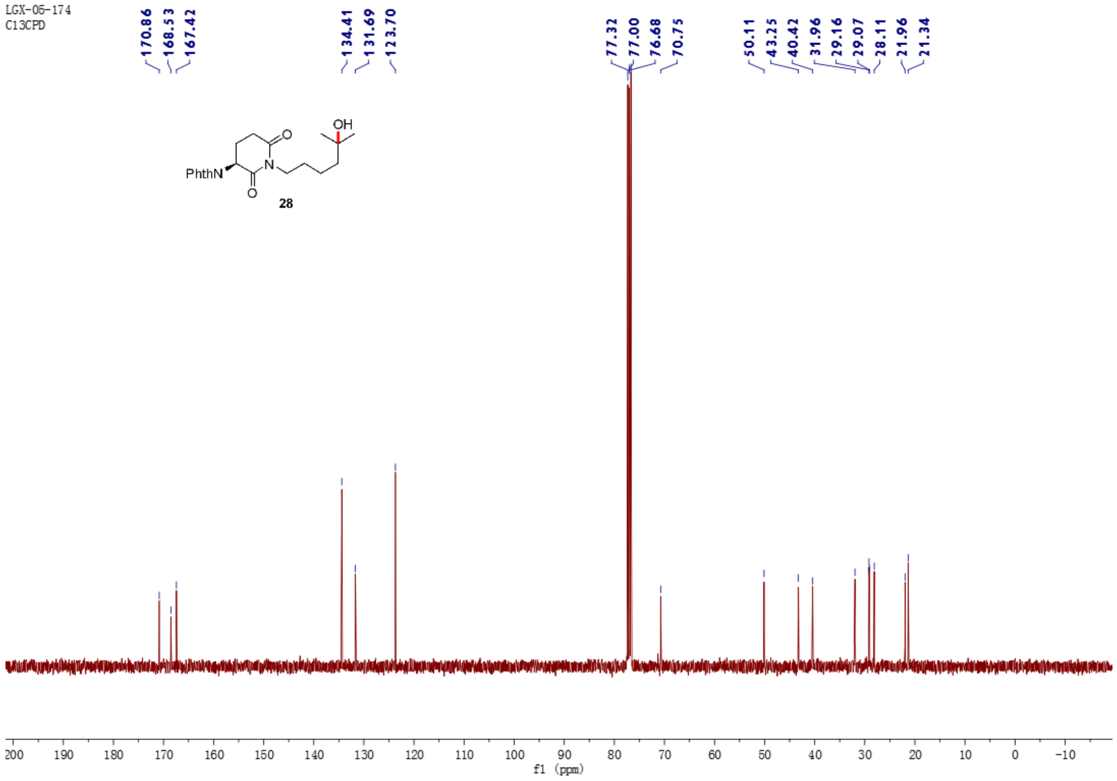

LGX-05-174
C13CPD
170.86
168.53
167.42
134.41
131.69
123.70
77.32
77.00
76.68
70.75
50.11
43.25
40.42
31.96
29.16
29.07
28.11
21.96
21.34
OH
PhthN
N
O
28
f1 (ppm)

lgx-05-166 H
PROTON

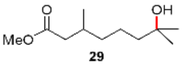

29

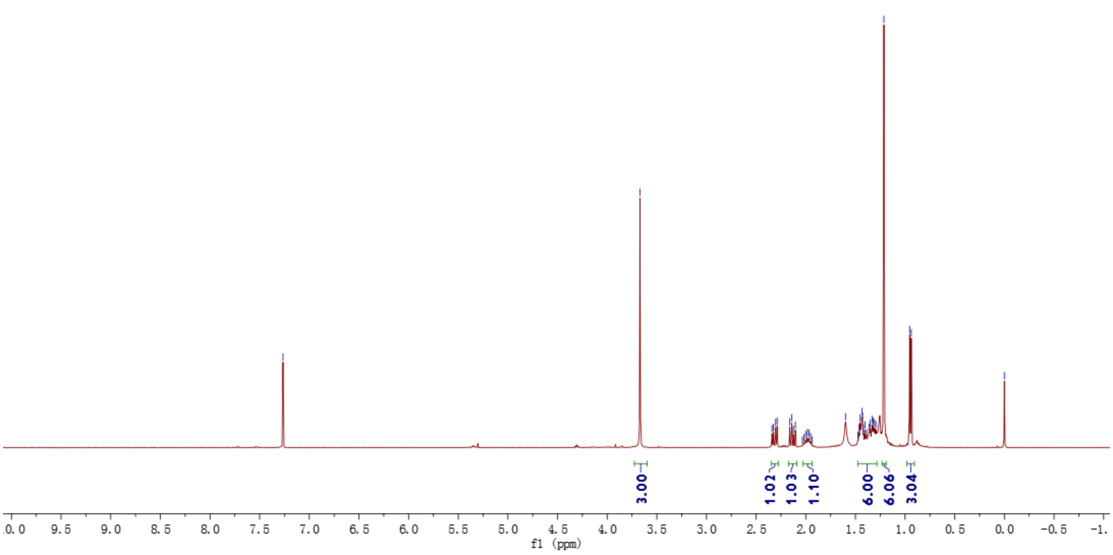

lgx-04-157

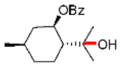

30

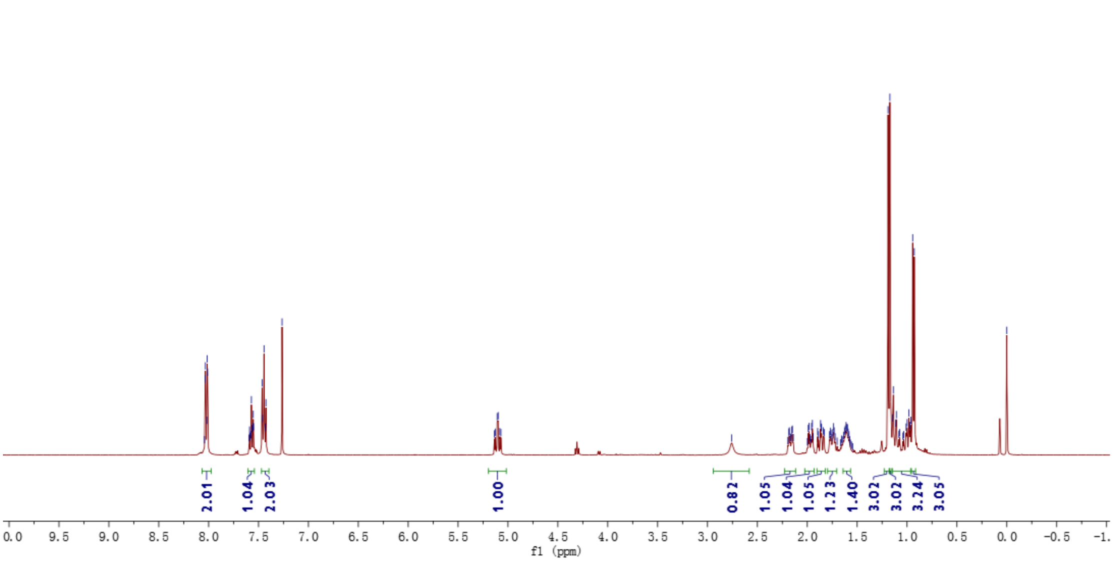

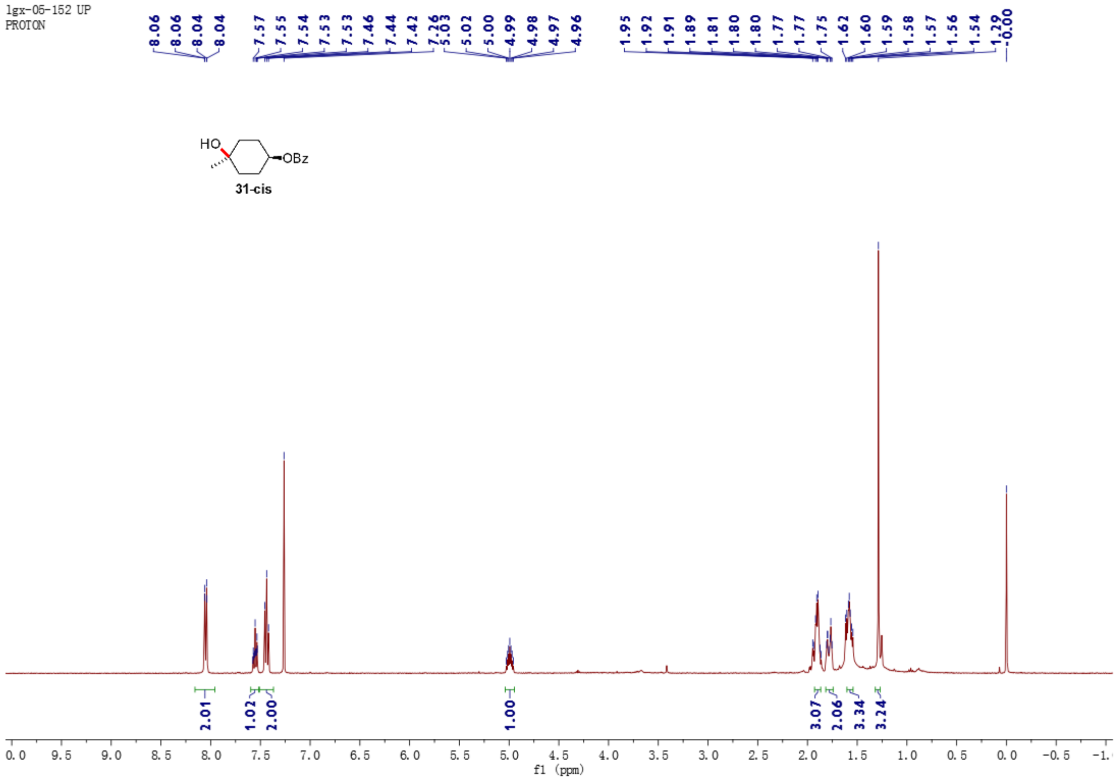
lgx-05-152 UP
PROTON
f1 (ppm)

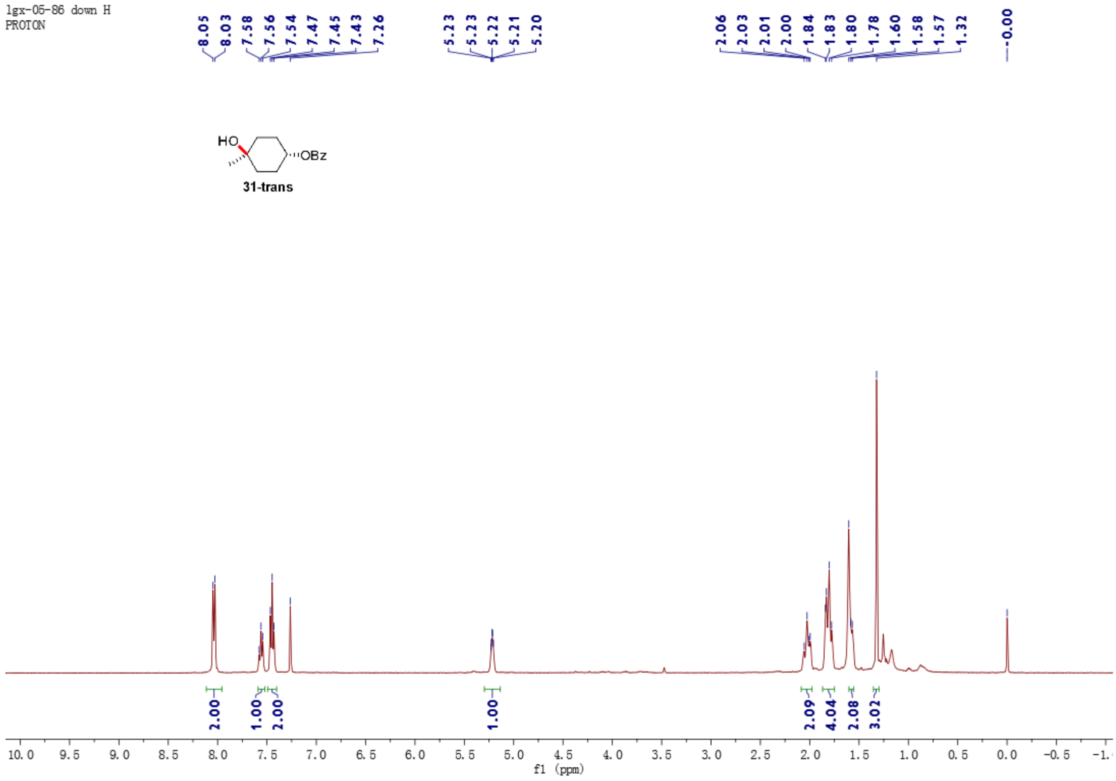
lgx-05-86 down H
PROTON
f1 (ppm)

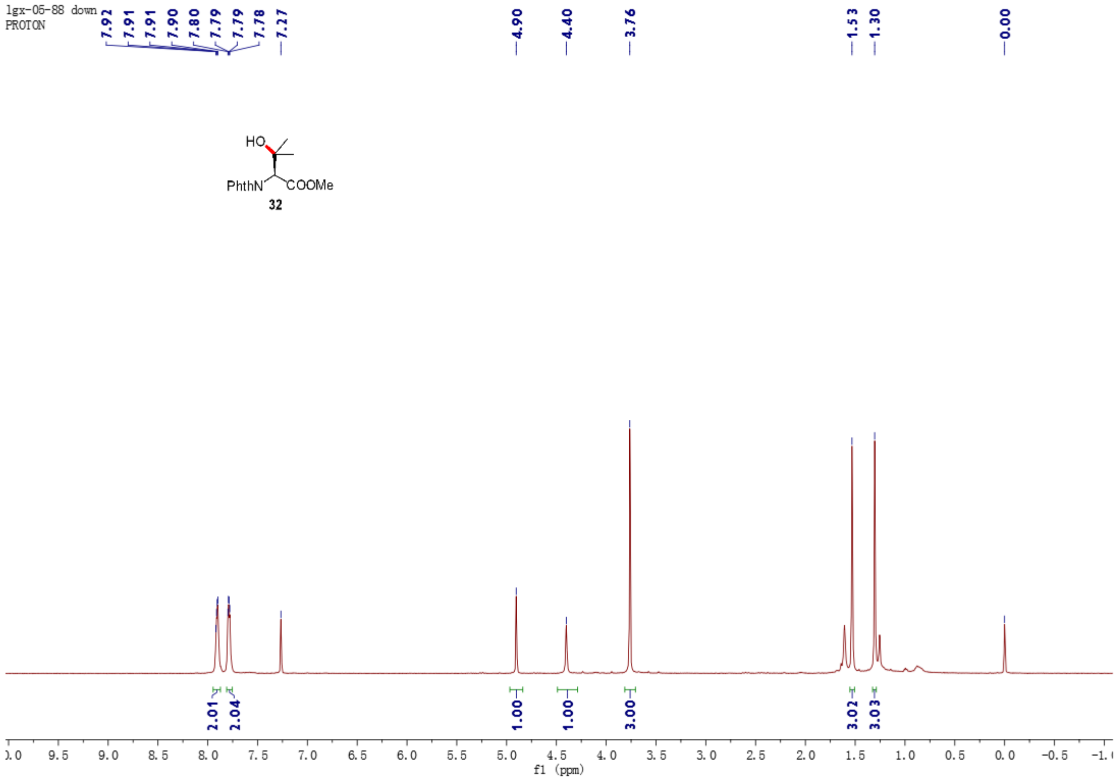
lgx-05-88 down
PROTON
7.92
7.91
7.91
7.90
7.80
7.79
7.79
7.78
7.27
4.90
4.40
3.76
1.53
1.30
0.00
HO
PhthN
COOMe
32
2.01
2.04
1.00
1.00
3.00
3.02
3.03
f1 (ppm)

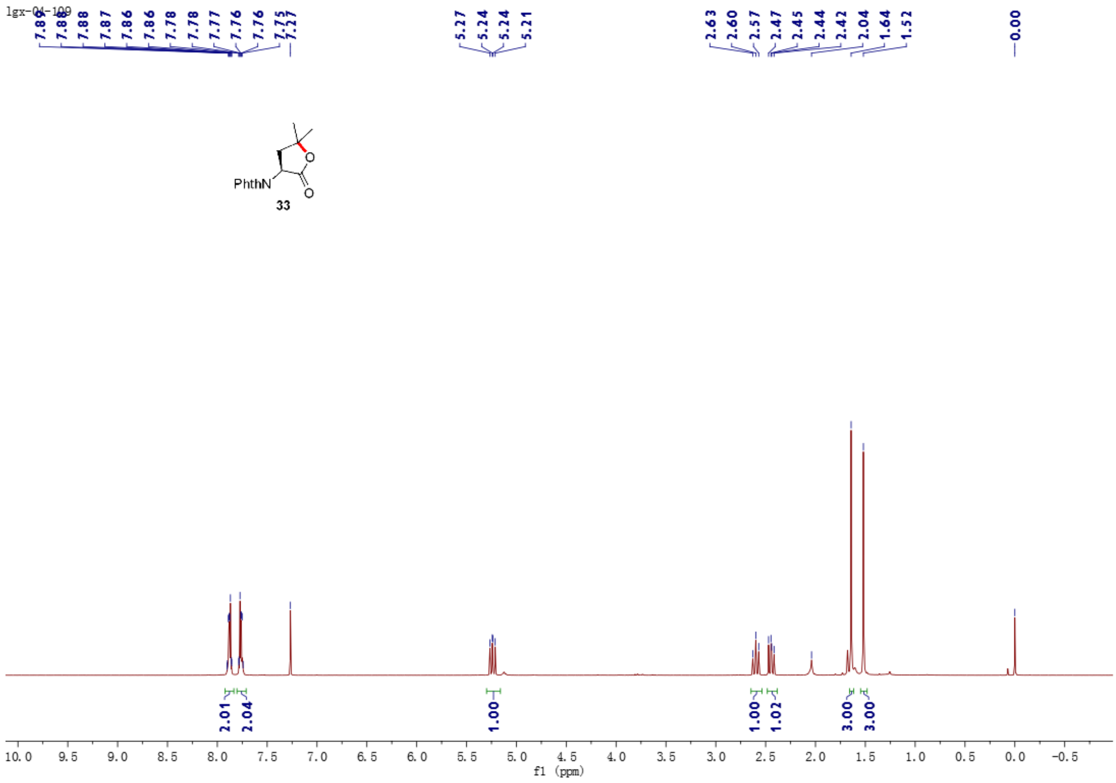
lgx-04-109
7.88
7.87
7.86
7.86
7.78
7.78
7.77
7.76
7.76
7.75
7.27
5.27
5.24
5.24
5.21
2.63
2.60
2.57
2.47
2.45
2.44
2.42
2.04
1.64
1.52
0.00
O
PhthN
O
33
2.01
2.04
1.00
1.00
1.02
3.00
3.00
f1 (ppm)

lgx-07-158-H

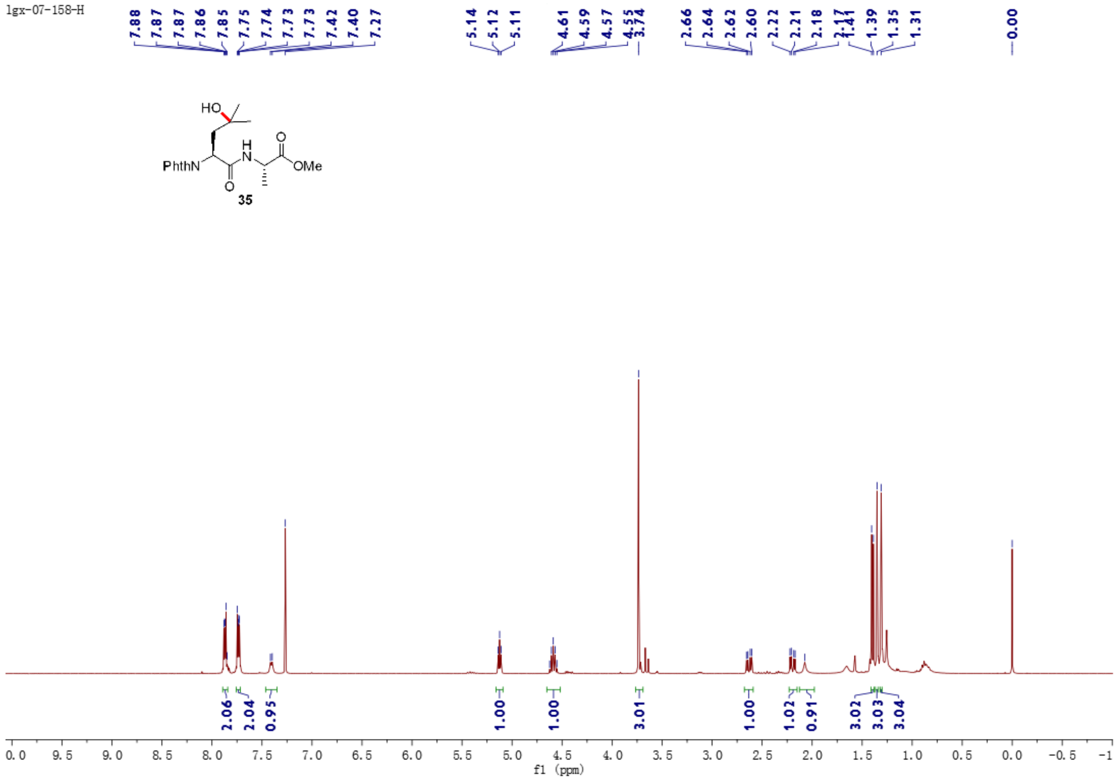

35

lgx-07-197-1-pure-H401
PROTON

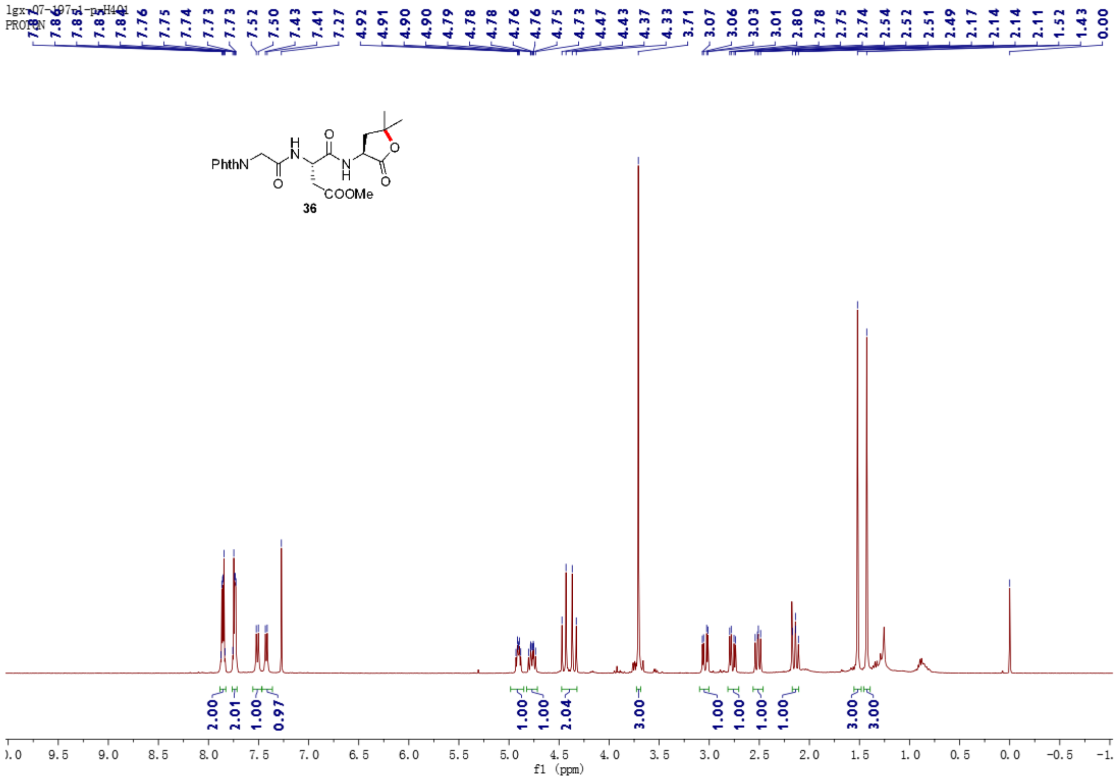

36

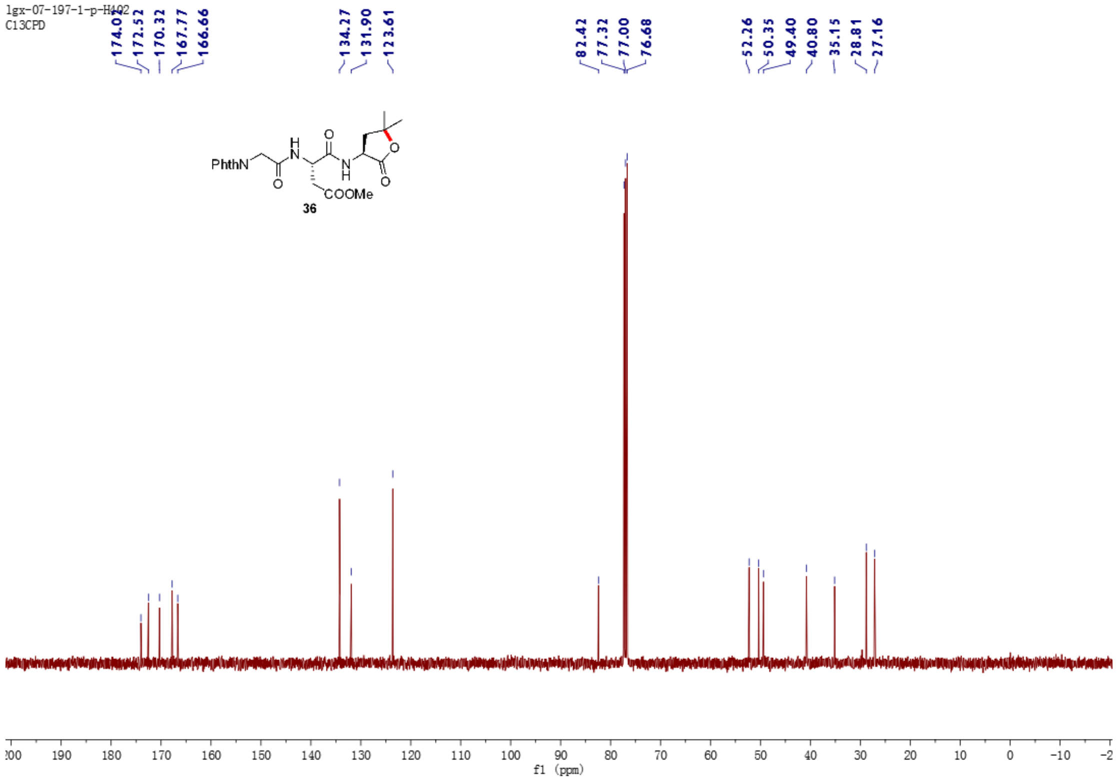

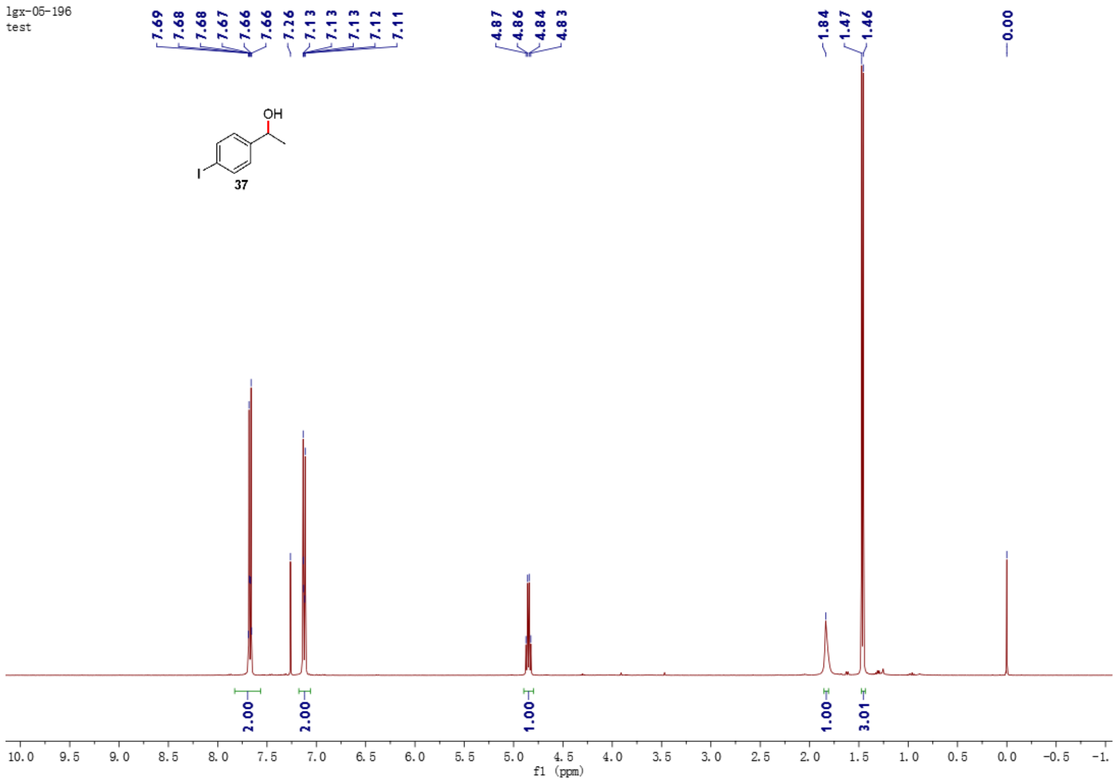

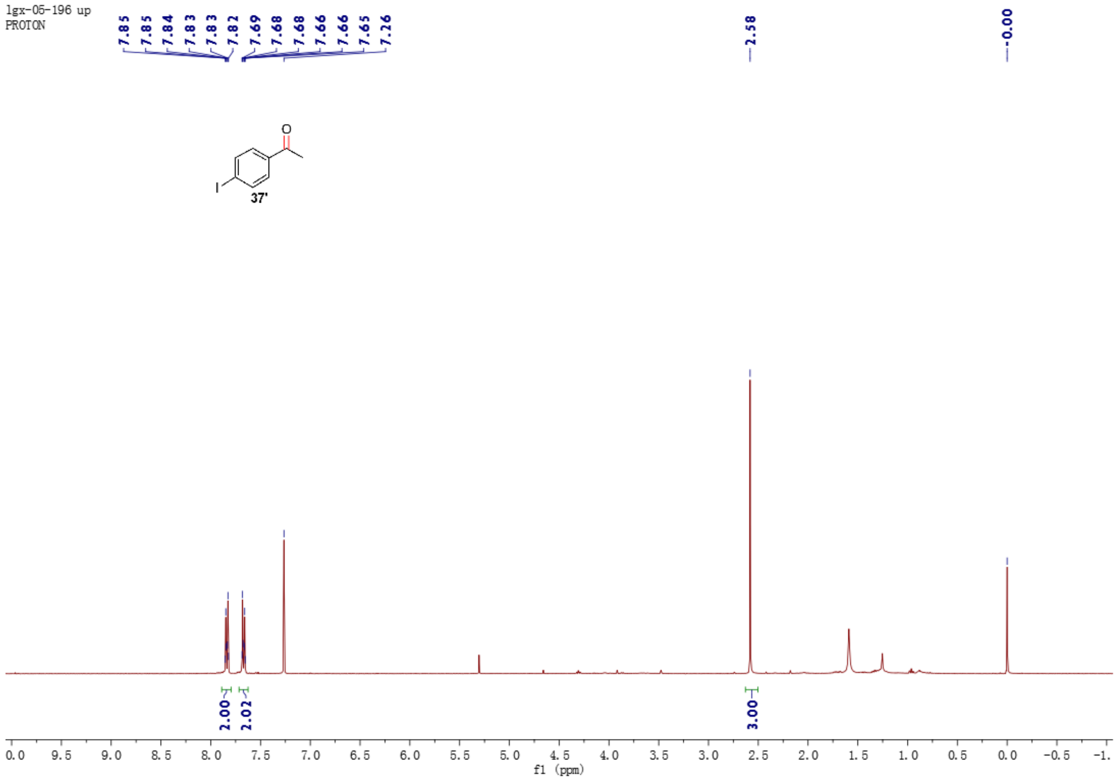
lgx-05-196 up
PROTON
7.85 7.85 7.84 7.83 7.83 7.82 7.69 7.68 7.68 7.66 7.66 7.65 7.26
2.58
-0.00
O
I
37'
2.00 2.02
3.00
0.0 9.5 9.0 8.5 8.0 7.5 7.0 6.5 6.0 5.5 5.0 4.5 4.0 3.5 3.0 2.5 2.0 1.5 1.0 0.5 0.0 -0.5 -1.
f1 (ppm)

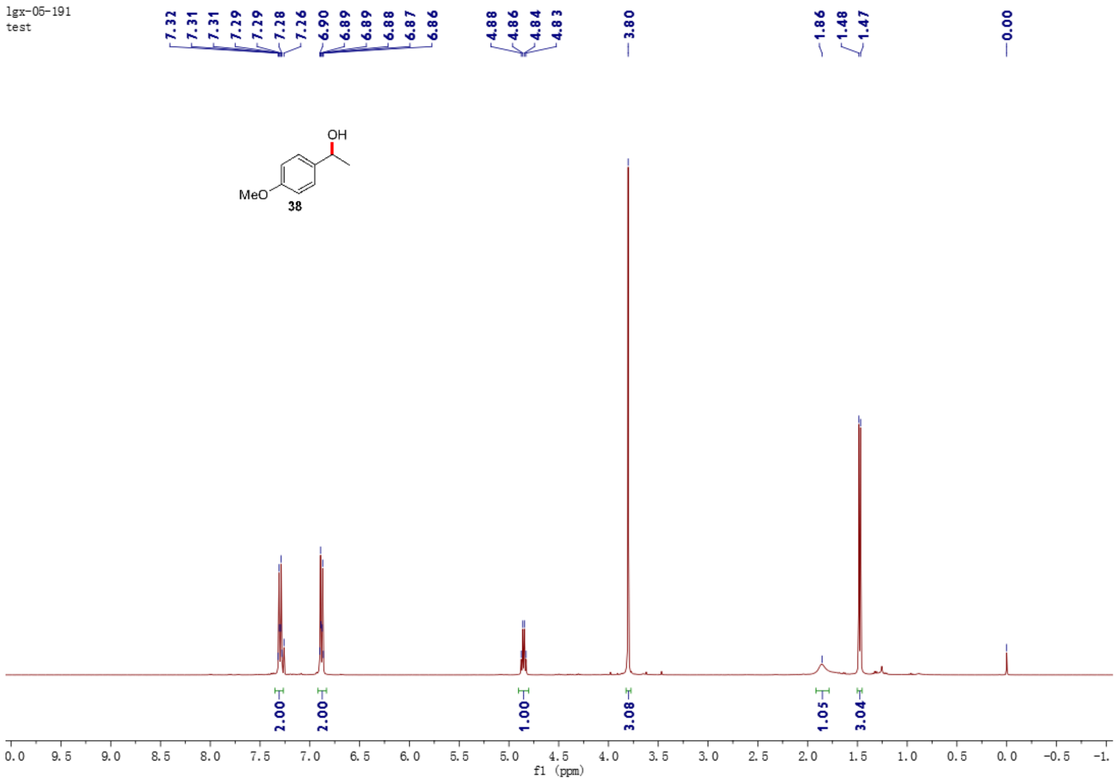
lgx-05-191
test
7.32 7.31 7.31 7.29 7.29 7.28 7.26 6.90 6.89 6.89 6.88 6.87 6.86
4.88 4.86 4.84 4.83
3.80
1.86 1.48 1.47
0.00
OH
MeO
38
2.00 2.00 1.00 3.08 1.05 3.04
0.0 9.5 9.0 8.5 8.0 7.5 7.0 6.5 6.0 5.5 5.0 4.5 4.0 3.5 3.0 2.5 2.0 1.5 1.0 0.5 0.0 -0.5 -1.
f1 (ppm)

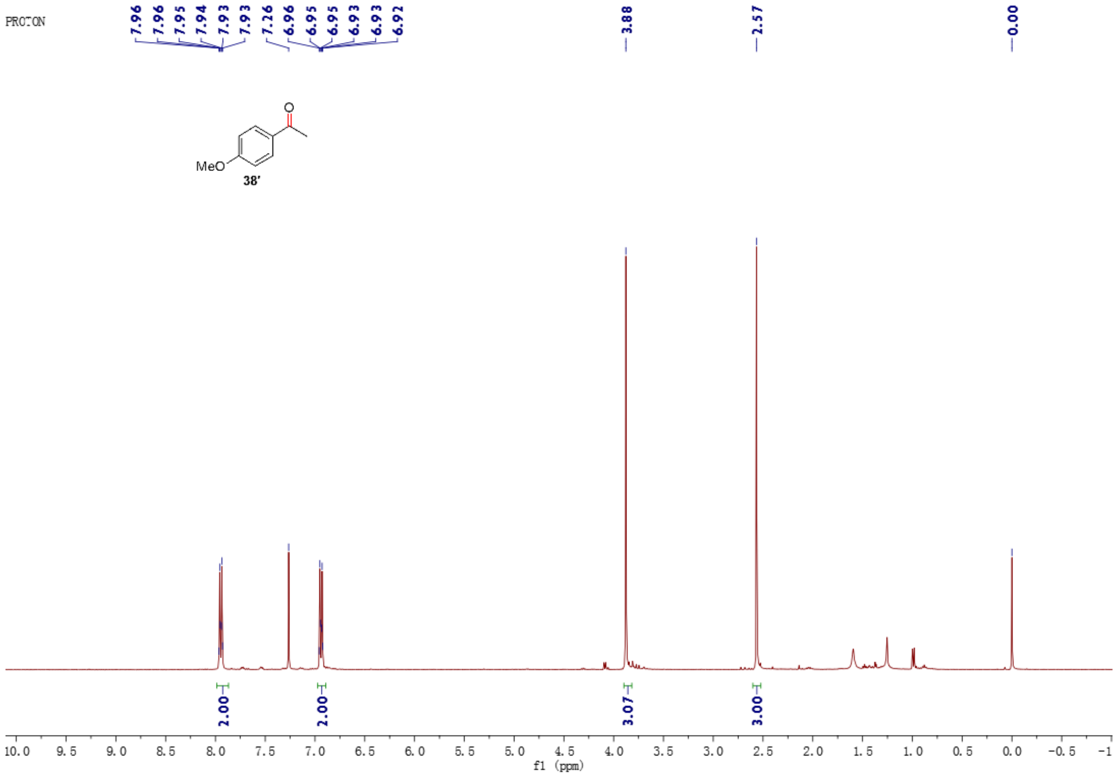
PROTON
7.96
7.96
7.95
7.94
7.93
7.93
7.26
6.96
6.95
6.95
6.93
6.93
6.92
3.88
2.57
0.00
MeO
O
38'
2.00
2.00
3.07
3.00
f1 (ppm)

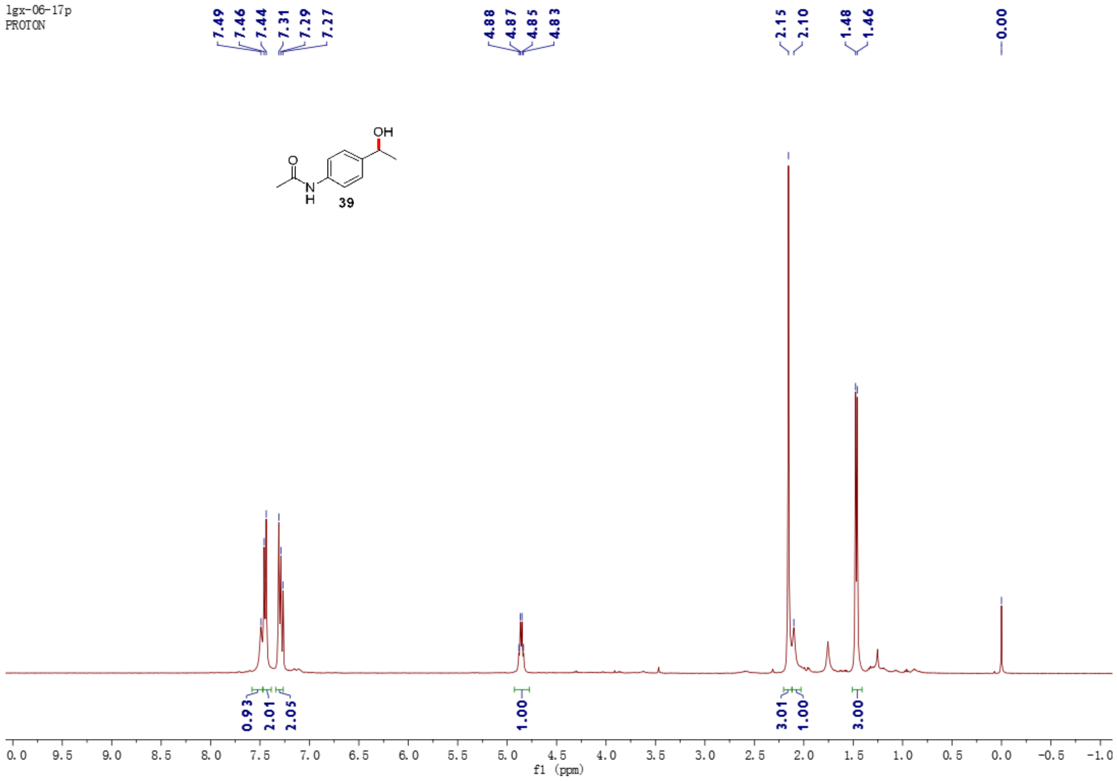
lgx-06-17p
PROTON
7.49
7.46
7.44
7.31
7.29
7.27
4.88
4.87
4.85
4.83
2.15
2.10
1.48
1.46
0.00
OH
O
N
H
39
0.93
2.01
2.05
1.00
3.01
1.00
3.00
f1 (ppm)

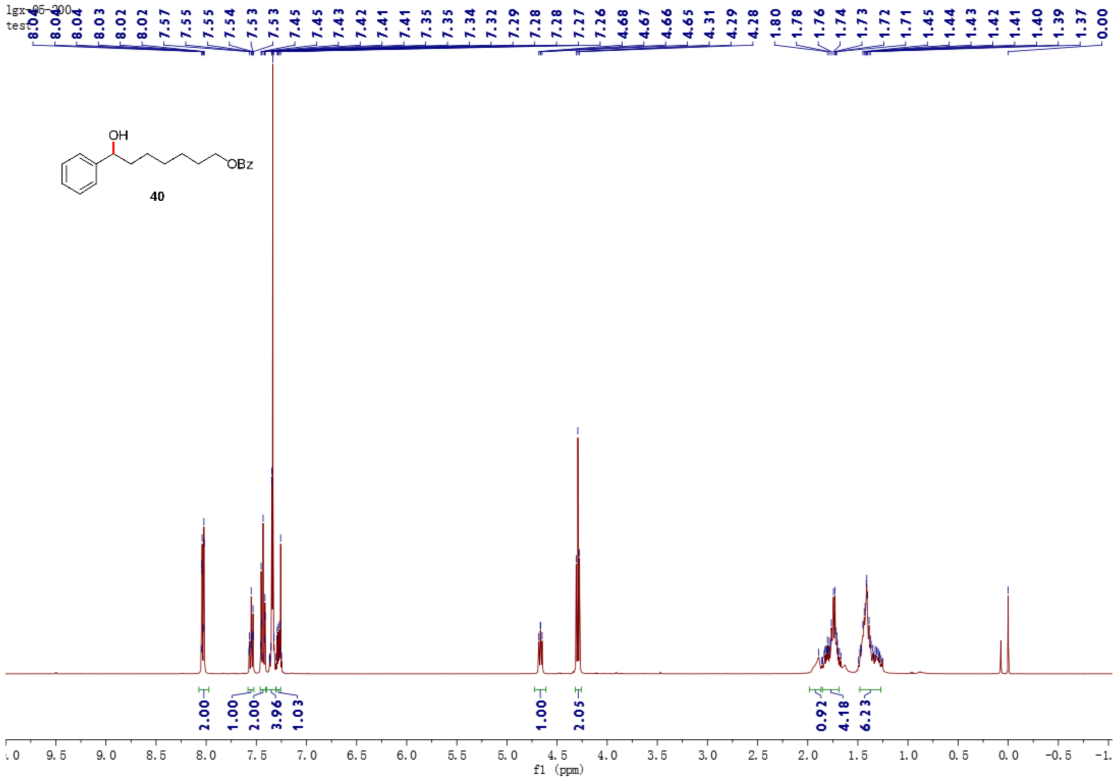

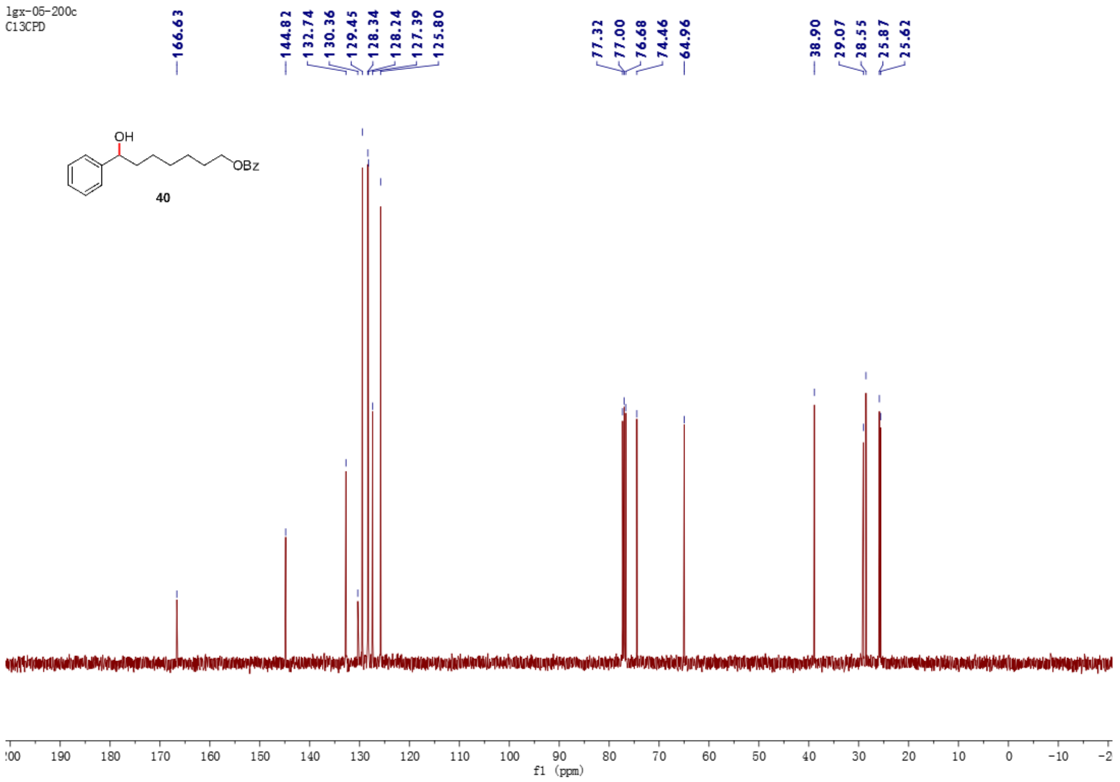

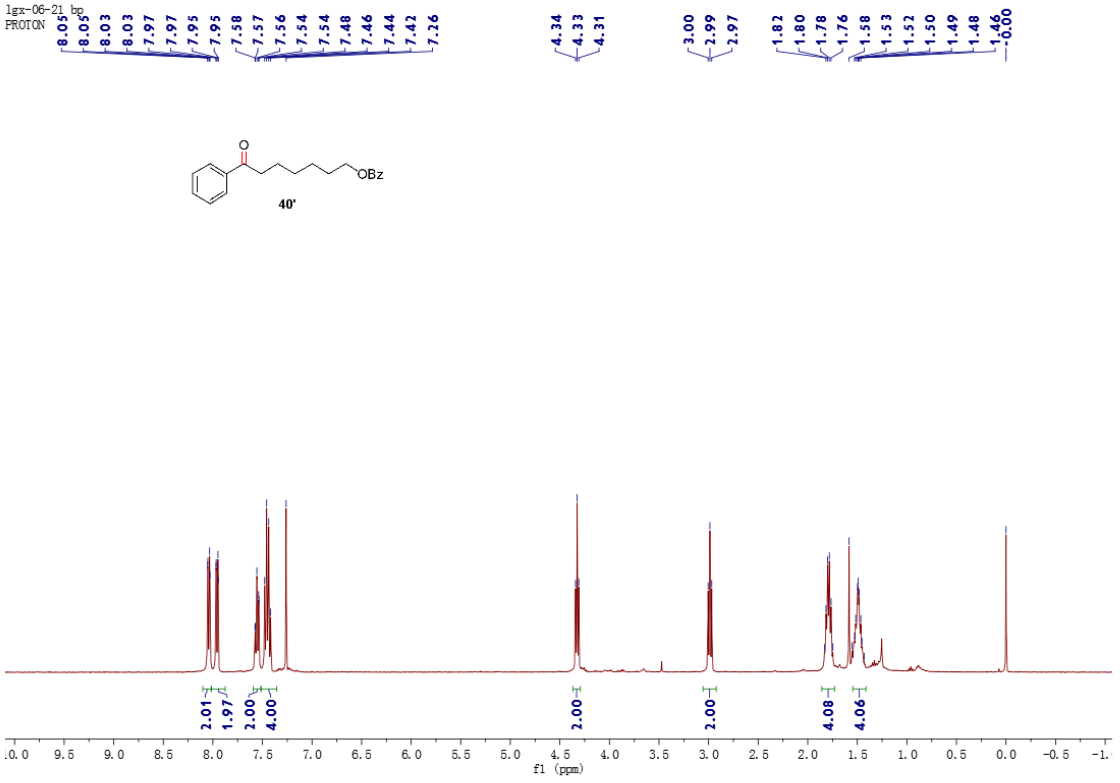

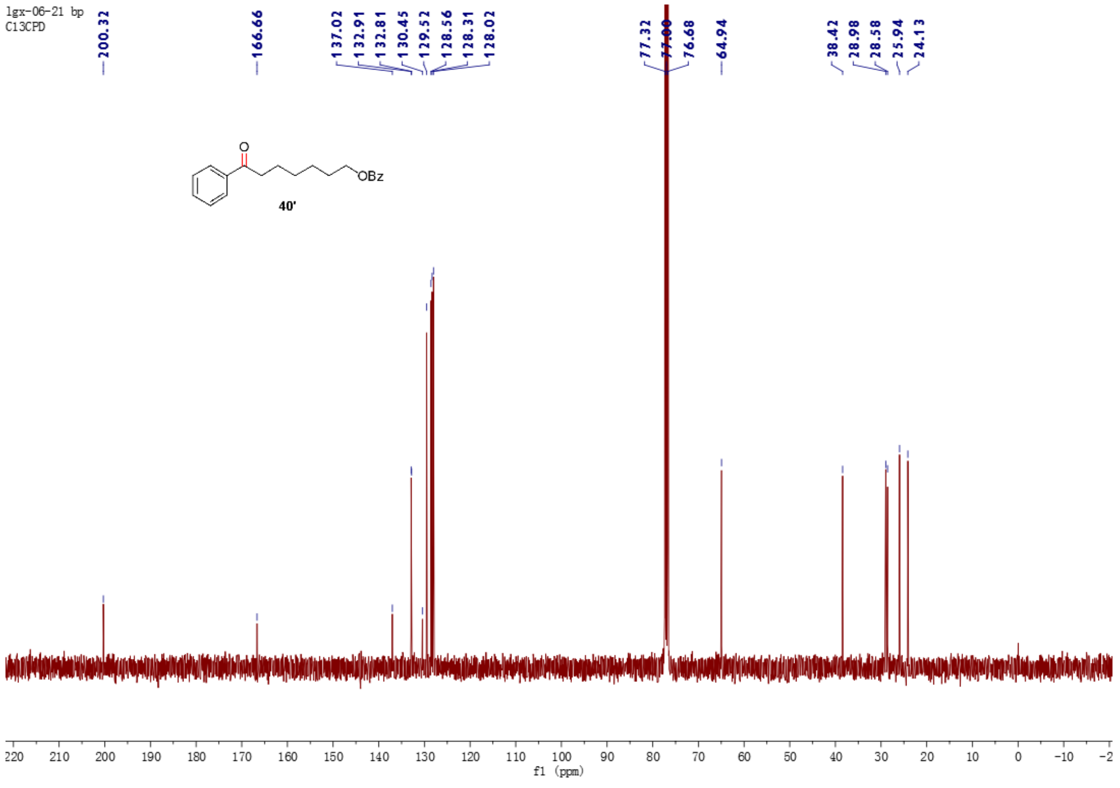

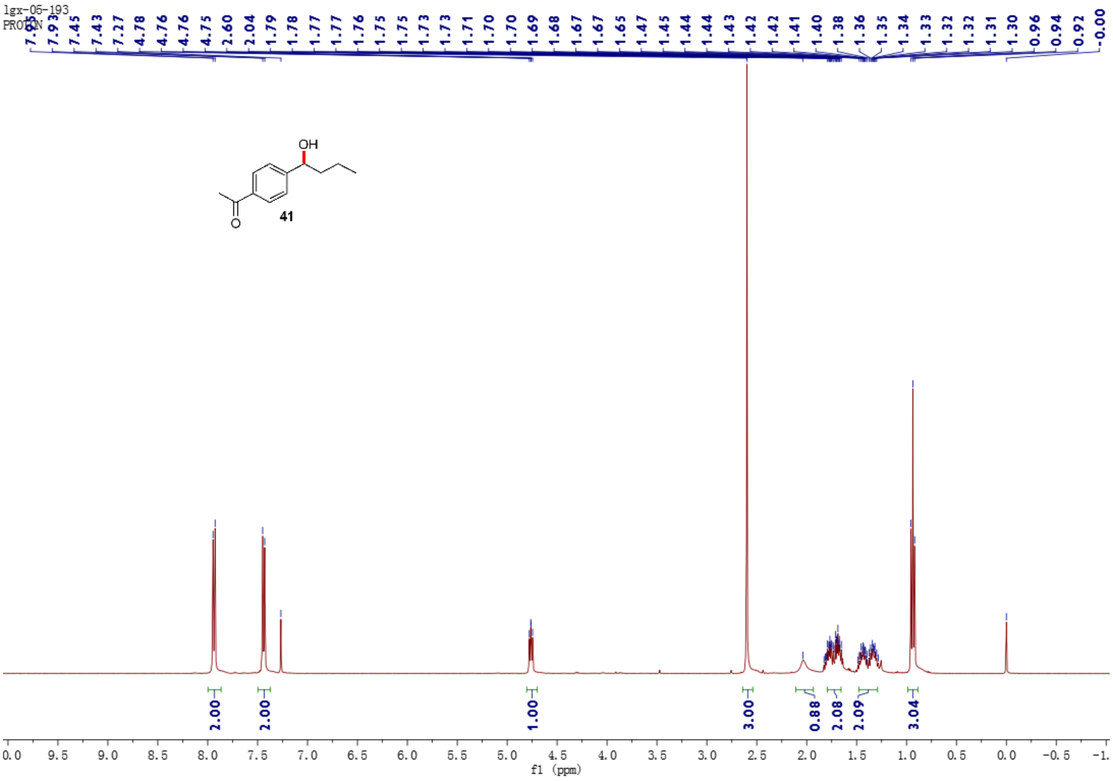
lgx-05-193
PROTON
7.93
7.45
7.43
7.27
4.78
4.76
4.76
4.75
2.60
2.04
1.79
1.78
1.77
1.77
1.76
1.75
1.75
1.73
1.73
1.71
1.70
1.70
1.69
1.68
1.67
1.67
1.65
1.47
1.45
1.44
1.44
1.43
1.42
1.42
1.41
1.40
1.38
1.36
1.35
1.34
1.33
1.32
1.32
1.31
1.30
0.96
0.94
0.92
-0.00
OH
O
41
2.00
2.00
1.00
3.00
0.88
2.08
2.09
3.04
f1 (ppm)

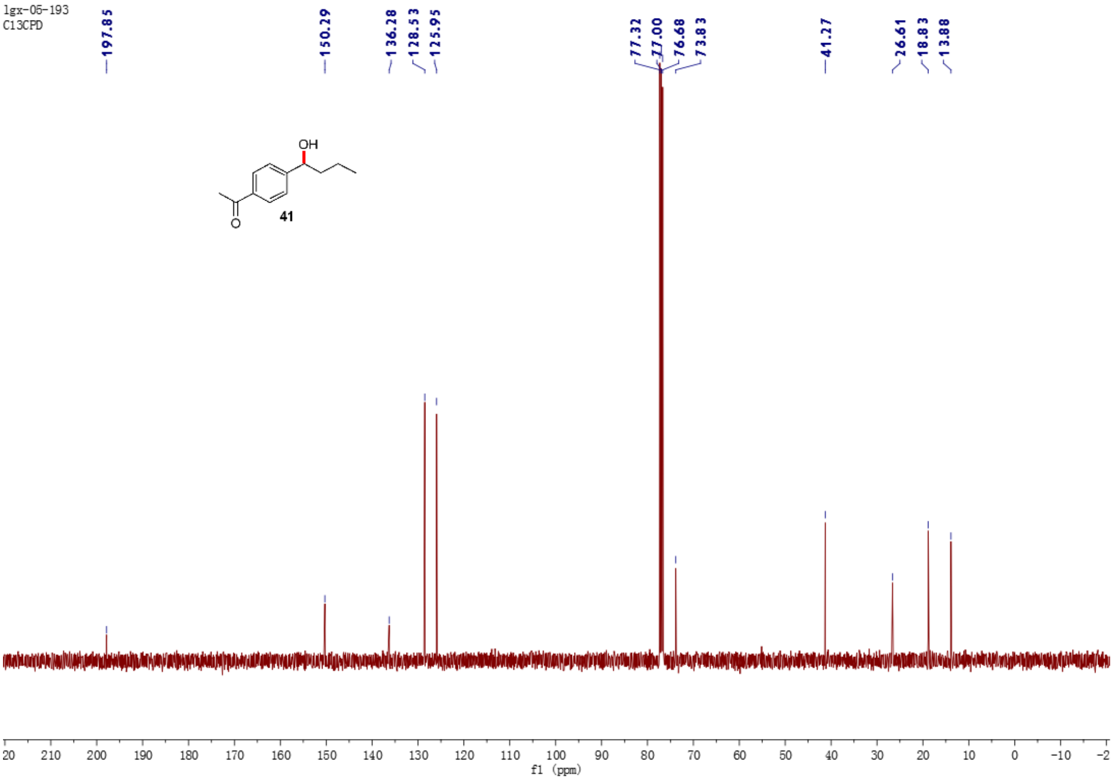
lgx-05-193
C13CPD
197.85
150.29
136.28
128.53
125.95
77.32
77.00
76.68
73.83
41.27
26.61
18.83
13.88
OH
O
41
f1 (ppm)

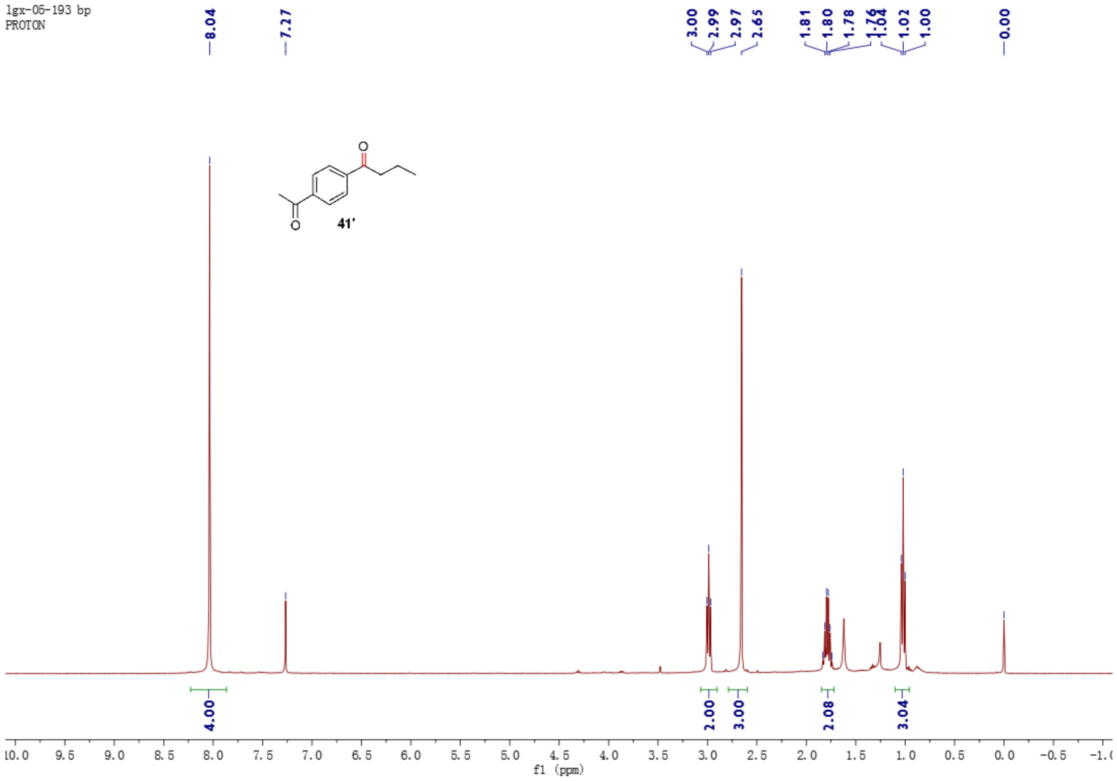
lgx-05-193 bp
PROTON
8.04
7.27
3.00
2.99
2.97
2.65
1.81
1.80
1.78
1.76
1.04
1.02
1.00
0.00
O
O
41'
4.00
2.00
3.00
2.08
3.04
f1 (ppm)

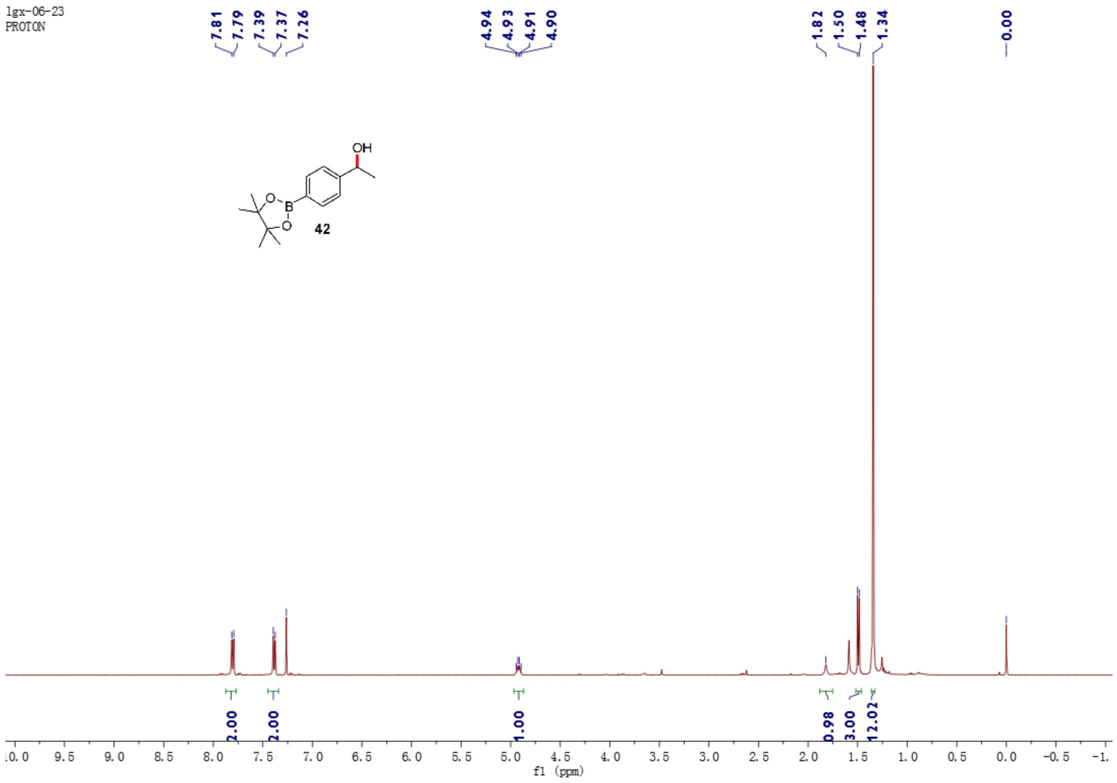
lgx-06-23
PROTON
7.81
7.79
7.39
7.37
7.26
4.94
4.93
4.91
4.90
1.82
1.50
1.48
1.34
0.00
OH
O
B
O
42
2.00
2.00
1.00
0.98
3.00
12.02
f1 (ppm)

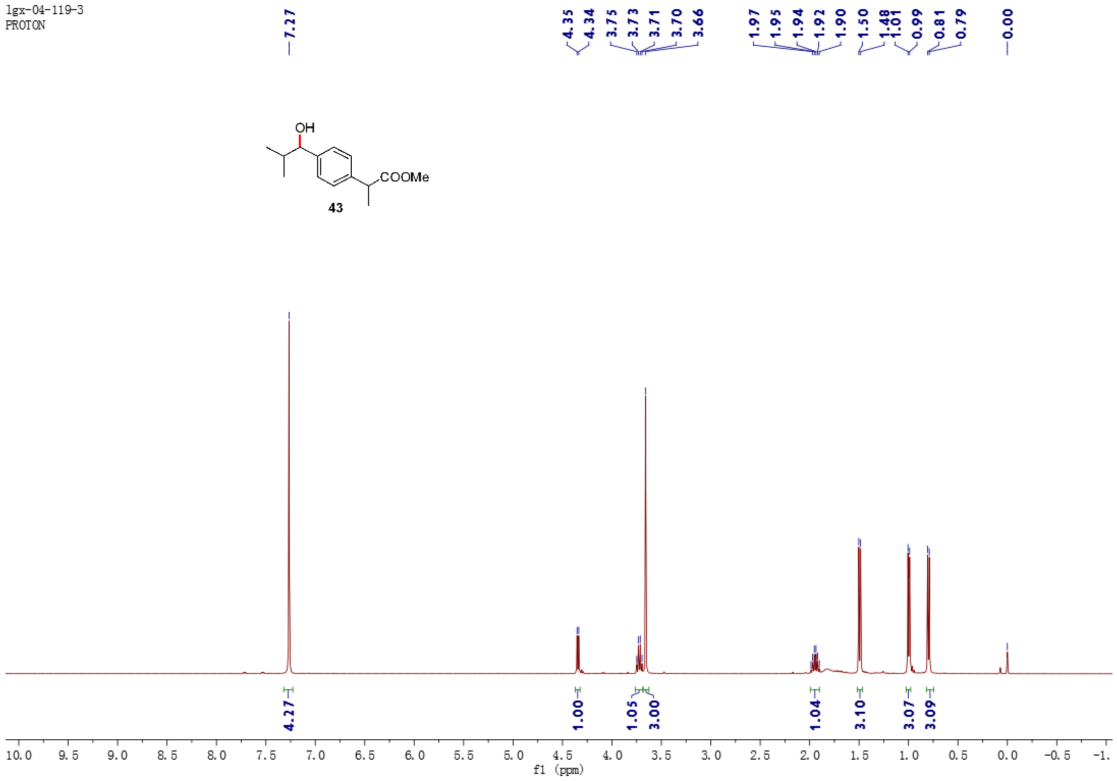
lgx-04-119-3
PROTON
OH
COOMe
43
f1 (ppm)

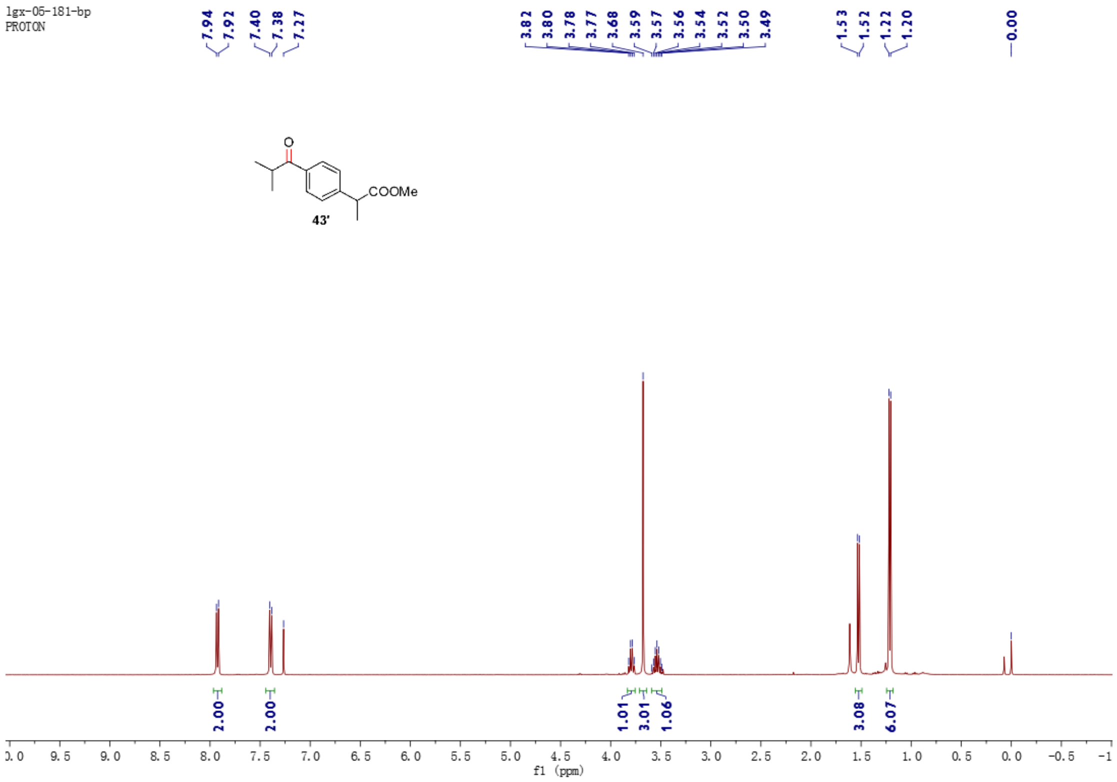
lgx-05-181-bp
PROTON
O
COOMe
43'
f1 (ppm)

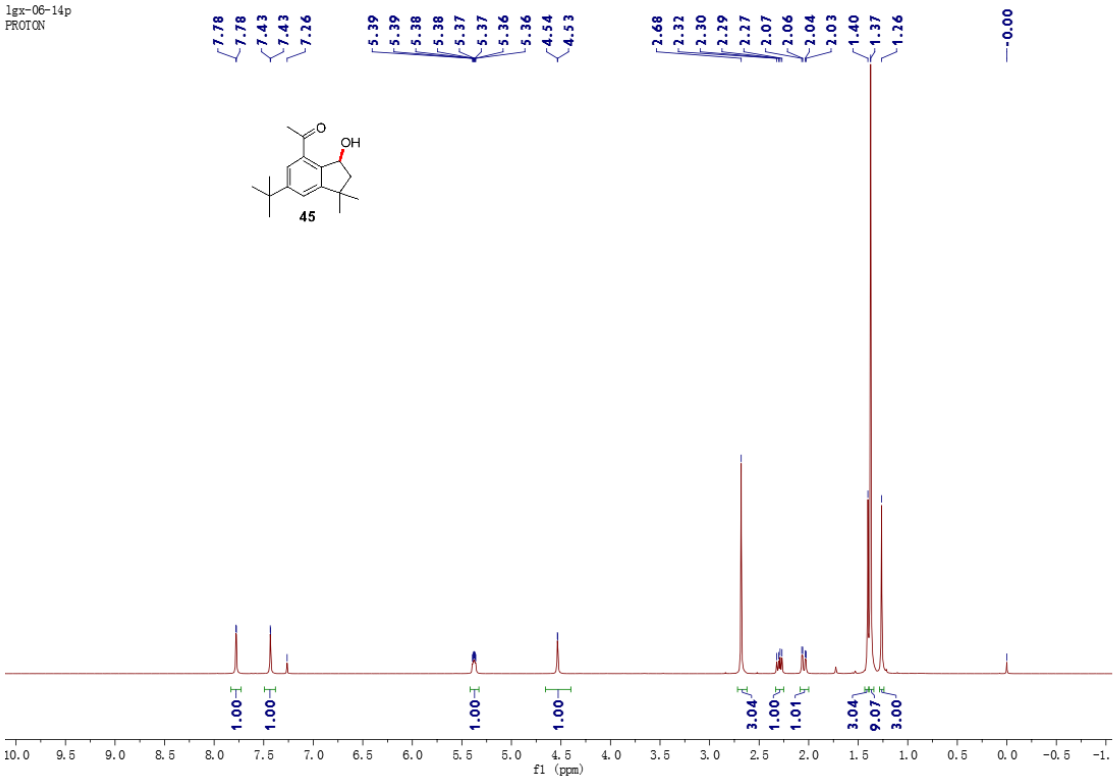
lgx-06-14p
PROTON
7.78
7.78
7.43
7.43
7.26
5.39
5.39
5.38
5.38
5.37
5.37
5.36
5.36
4.54
4.53
2.68
2.32
2.30
2.29
2.27
2.07
2.06
2.04
2.03
1.40
1.37
1.26
-0.00
OH
45
1.00
1.00
1.00
1.00
3.04
1.00
1.01
3.04
9.07
3.00
f1 (ppm)

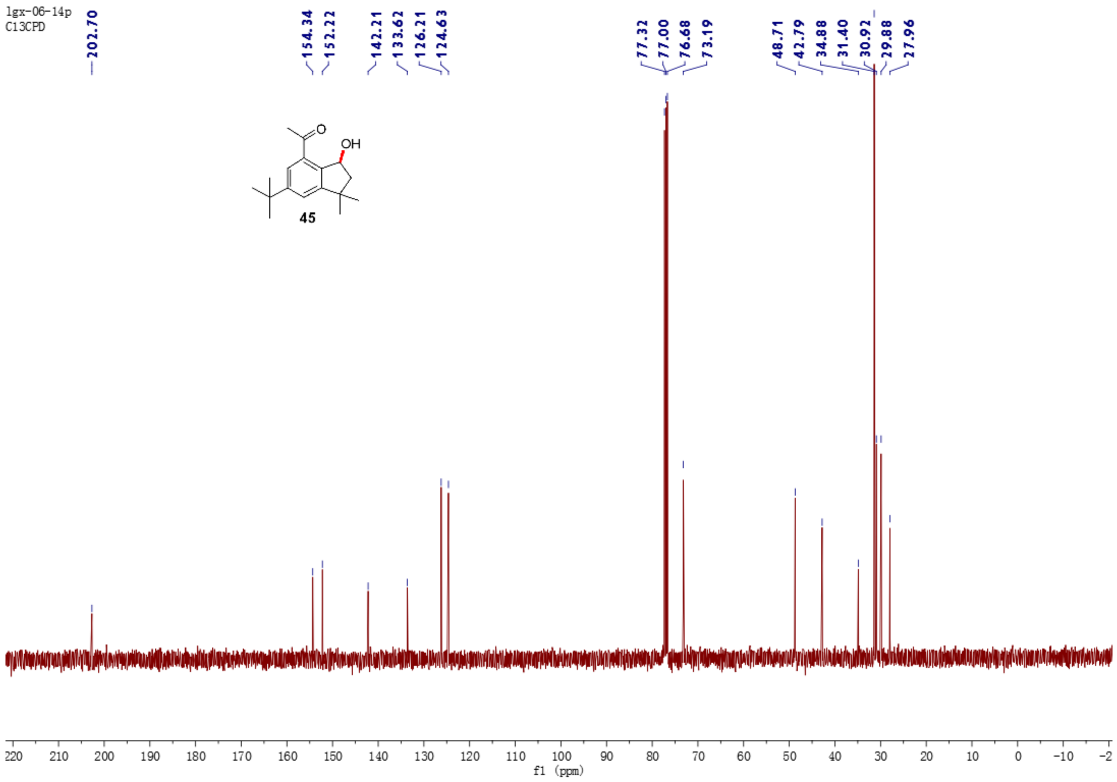
lgx-06-14p
C13CPD
202.70
154.34
152.22
142.21
133.62
126.21
124.63
77.32
77.00
76.68
73.19
48.71
42.79
34.88
31.40
30.92
29.88
27.96
OH
45
f1 (ppm)

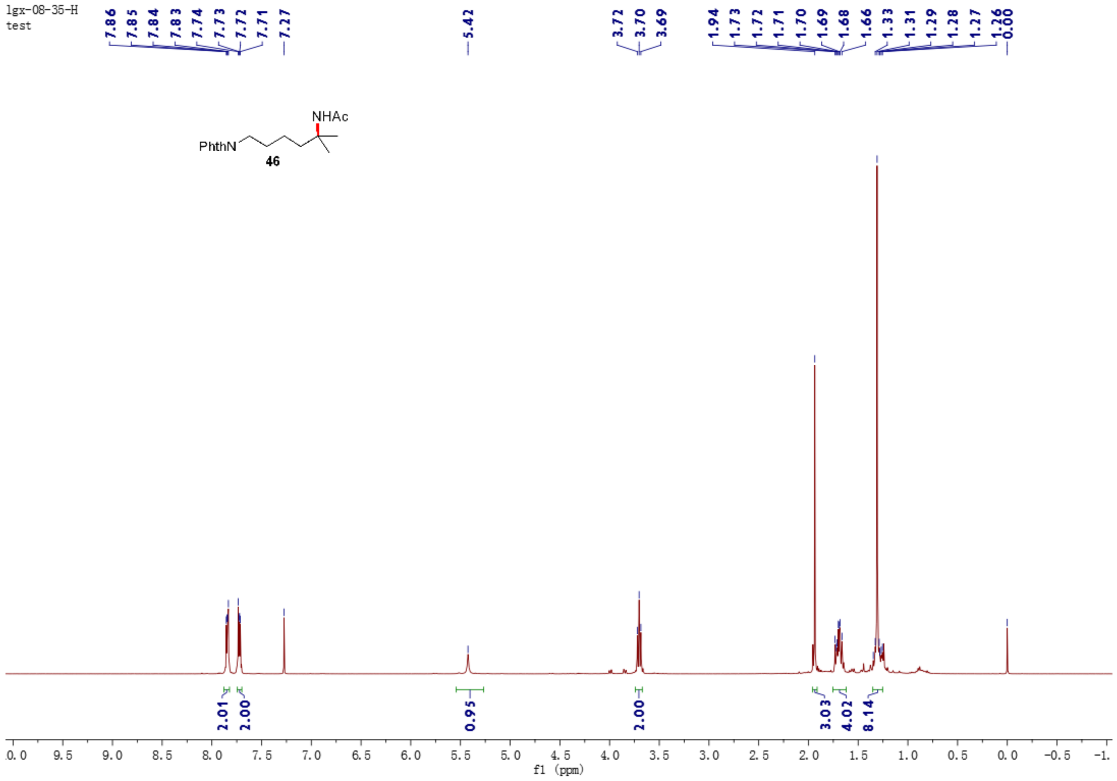
lgx-08-35-H
test
PhthN
NHAc
46
f1 (ppm)

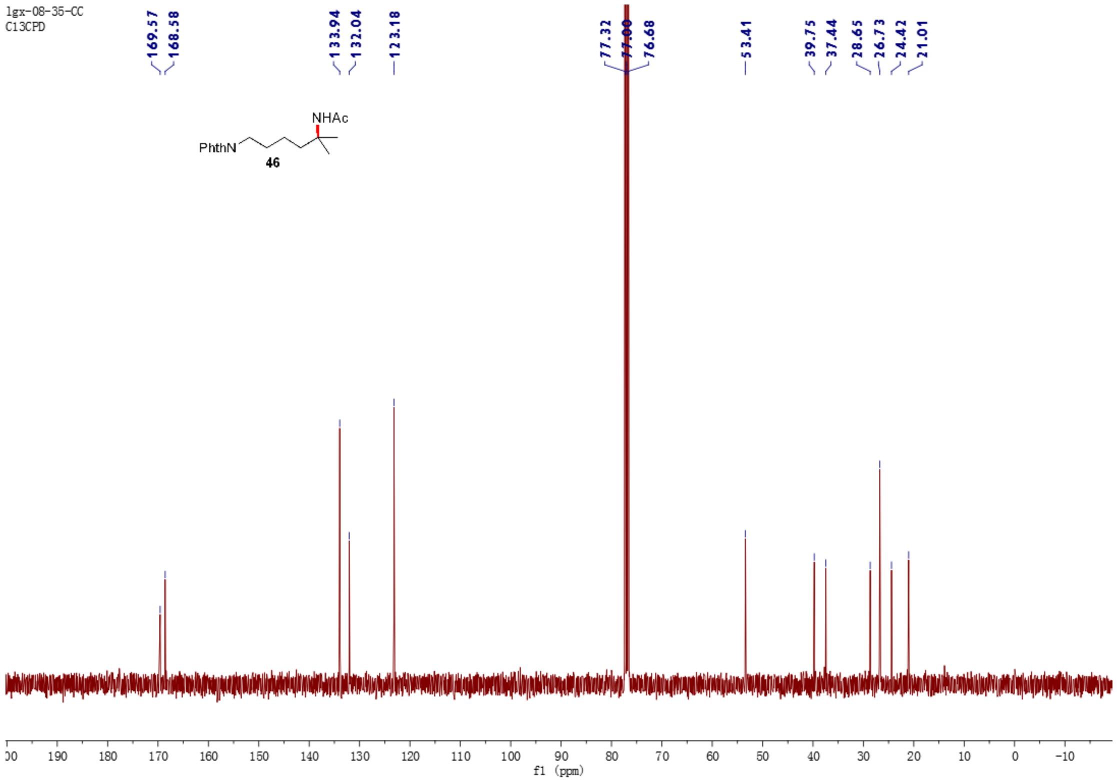
lgx-08-35-CC
C13CPD
PhthN
NHAc
46
f1 (ppm)

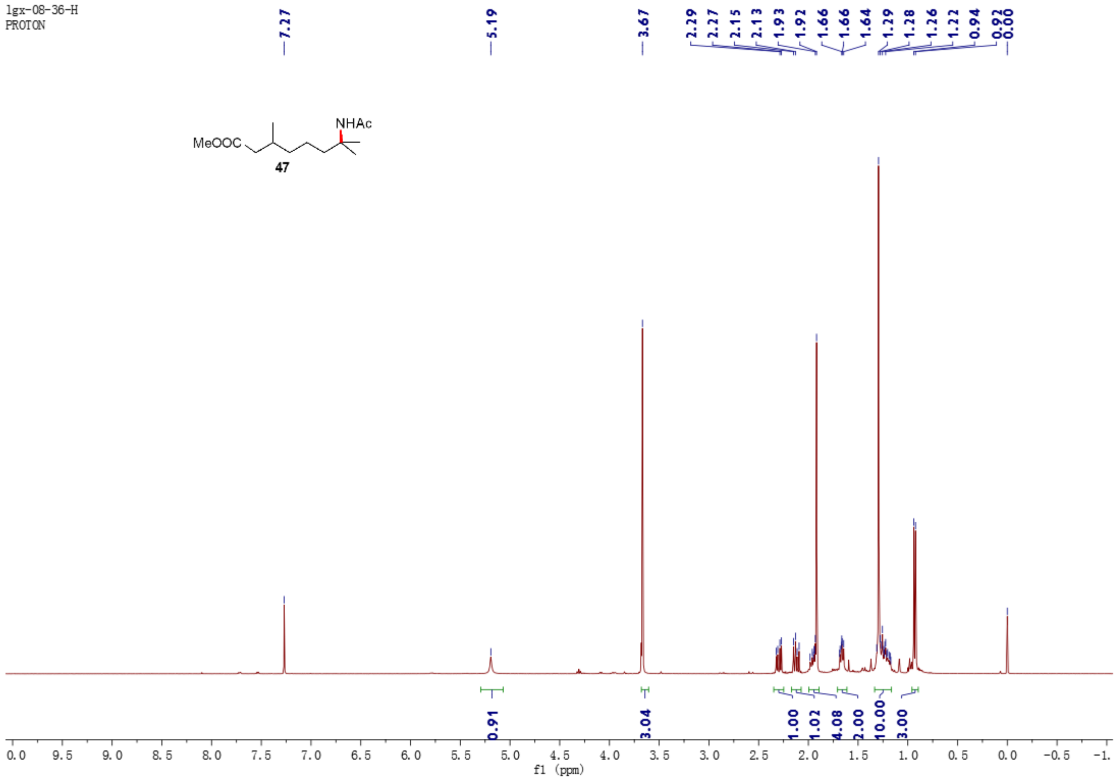
lgx-08-36-H
PROTON
7.27
5.19
3.67
2.29
2.27
2.15
2.13
1.93
1.92
1.66
1.66
1.64
1.29
1.28
1.26
1.22
0.94
0.92
0.00
NHAc
MeOOC
47
0.91
3.04
1.00
1.02
4.08
2.00
10.00
3.00
f1 (ppm)

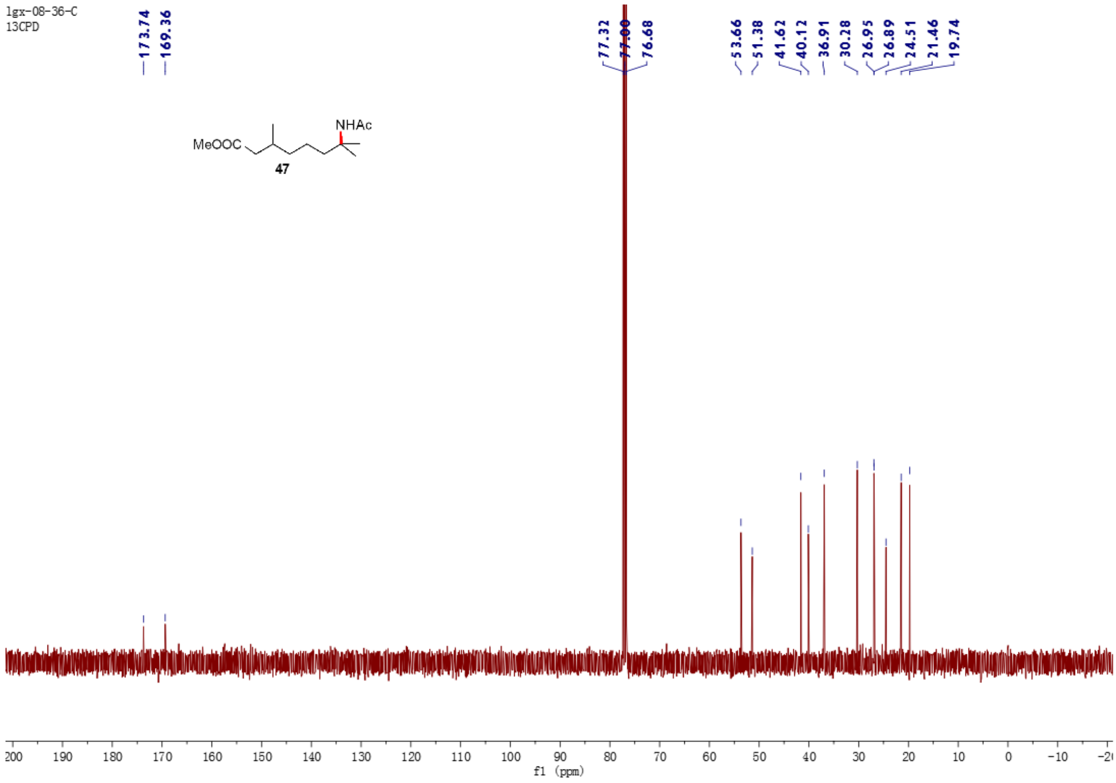
lgx-08-36-C
13CPD
173.74
169.36
77.32
77.00
76.68
53.66
51.38
41.62
40.12
36.91
30.28
26.95
26.89
24.51
21.46
19.74
NHAc
MeOOC
47
f1 (ppm)

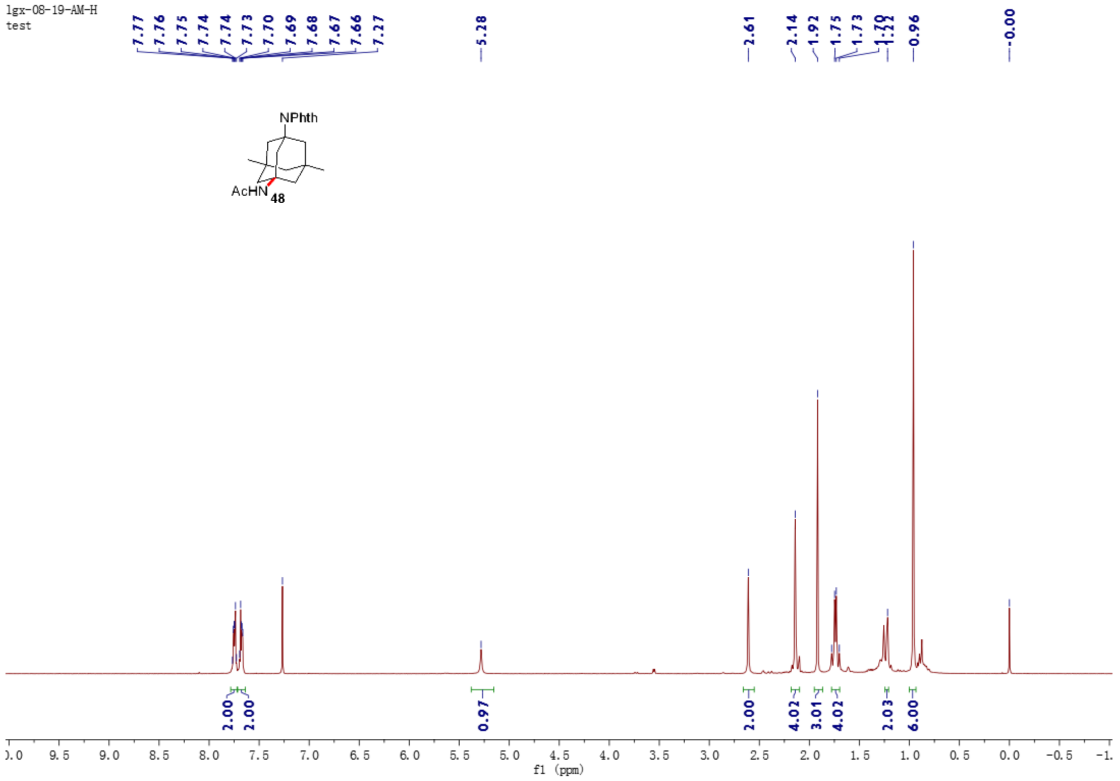

lgx-08-19-AM-H
test
7.77
7.76
7.75
7.74
7.74
7.73
7.70
7.69
7.68
7.67
7.66
7.27
5.28
2.61
2.14
1.92
1.75
1.73
1.70
1.21
0.96
-0.00
NPhth
AcHN
48
2.00
2.00
0.97
2.00
4.02
3.01
4.02
2.03
6.00
f1 (ppm)

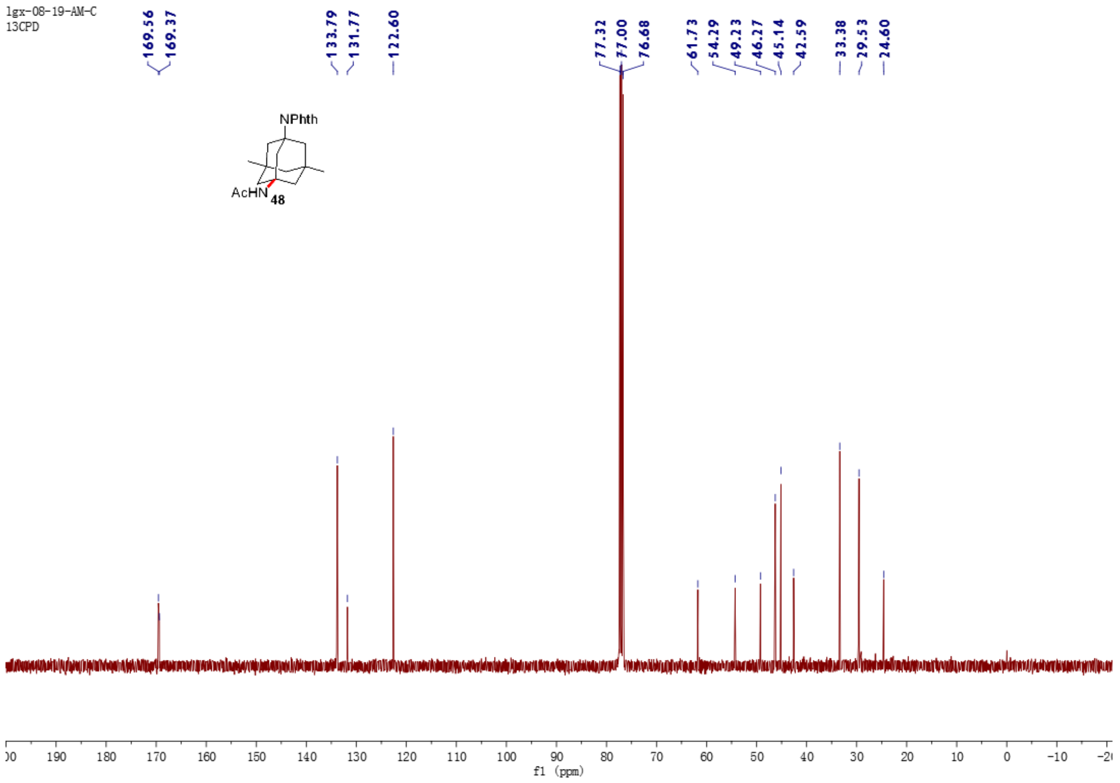

lgx-08-19-AM-C
13CPD
169.56
169.37
133.79
131.77
122.60
77.32
77.00
76.68
61.73
54.29
49.23
46.27
45.14
42.59
33.38
29.53
24.60
NPhth
AcHN
48
f1 (ppm)

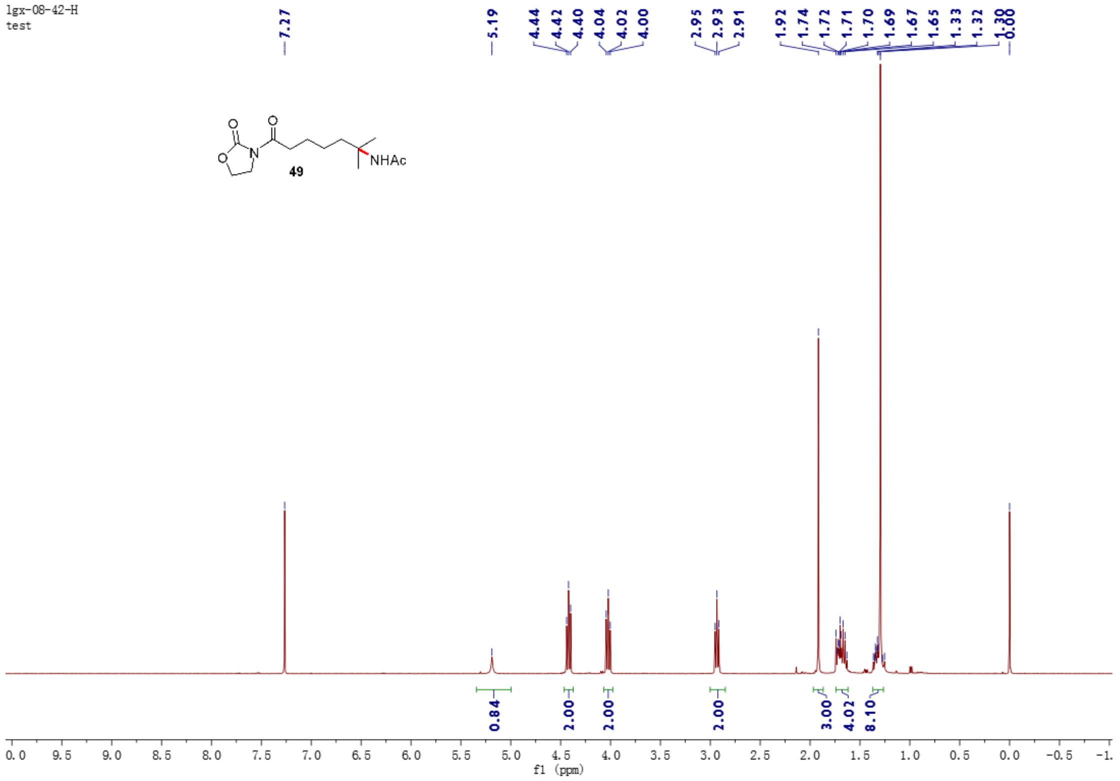
lgx-08-42-H
test
7.27
5.19
4.44
4.42
4.40
4.04
4.02
4.00
2.95
2.93
2.91
1.92
1.74
1.72
1.71
1.70
1.69
1.67
1.65
1.33
1.32
1.30
0.00
NHAc
49
0.84
2.00
2.00
2.00
3.00
4.02
8.10
f1 (ppm)

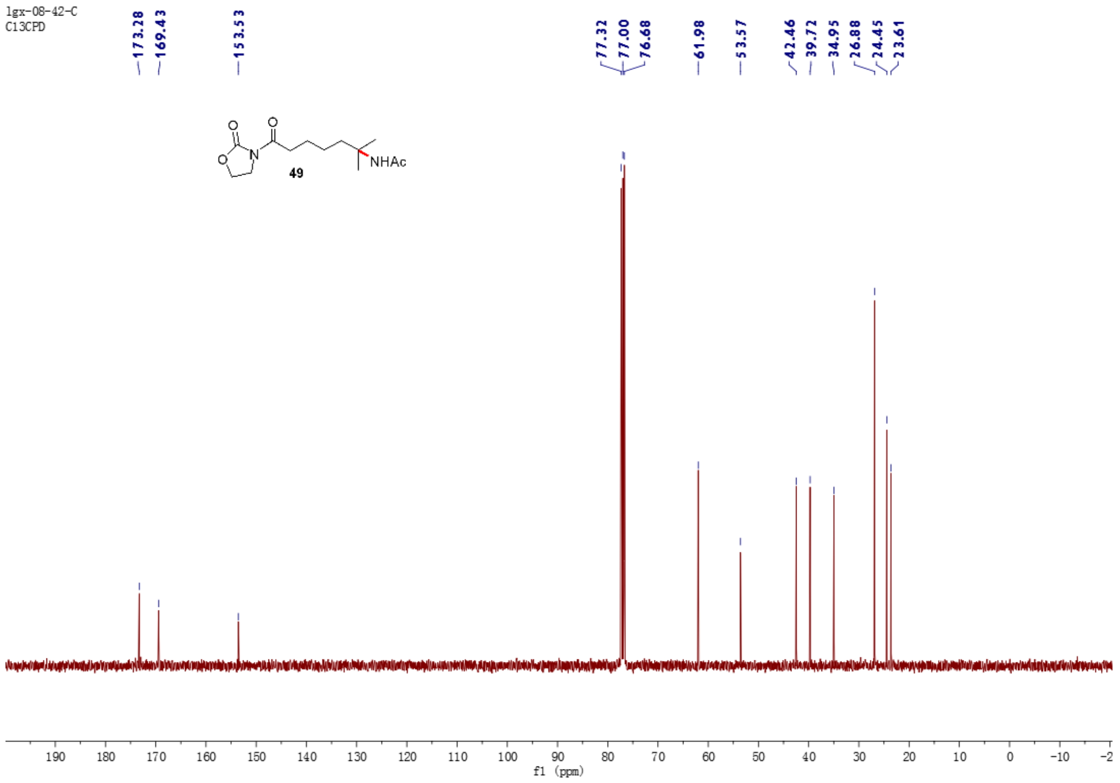
lgx-08-42-C
C13CPD
173.28
169.43
153.53
77.32
77.00
76.68
61.98
53.57
42.46
39.72
34.95
26.88
24.45
23.61
NHAc
49
f1 (ppm)

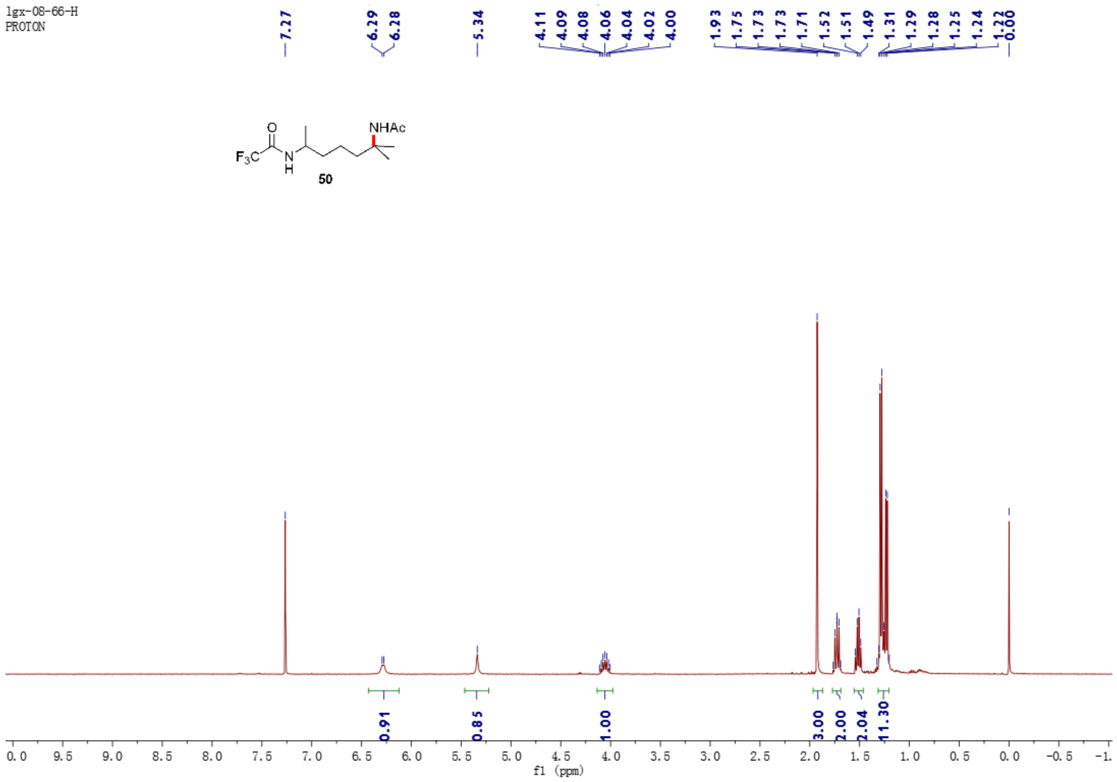
lgx-08-66-H
PROTON
7.27
6.29
6.28
5.34
4.11
4.09
4.08
4.06
4.04
4.02
4.00
1.93
1.75
1.73
1.73
1.71
1.52
1.51
1.49
1.31
1.29
1.28
1.25
1.24
1.22
0.00
NHAc
F_3C
O
N
H
50
0.91
0.85
1.00
3.00
2.00
2.04
11.30
f1 (ppm)

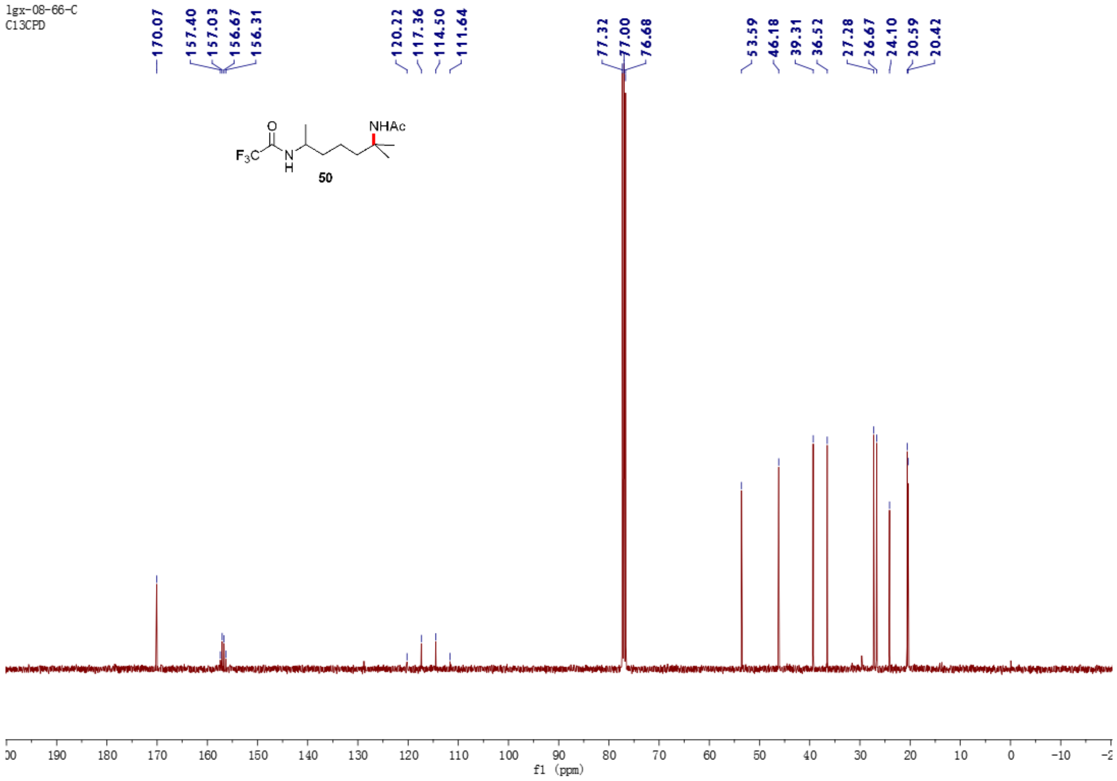
lgx-08-66-C
C13CPD
170.07
157.40
157.03
156.67
156.31
120.22
117.36
114.50
111.64
77.32
77.00
76.68
53.59
46.18
39.31
36.52
27.28
26.67
24.10
20.59
20.42
NHAc
F_3C
O
N
H
50
f1 (ppm)

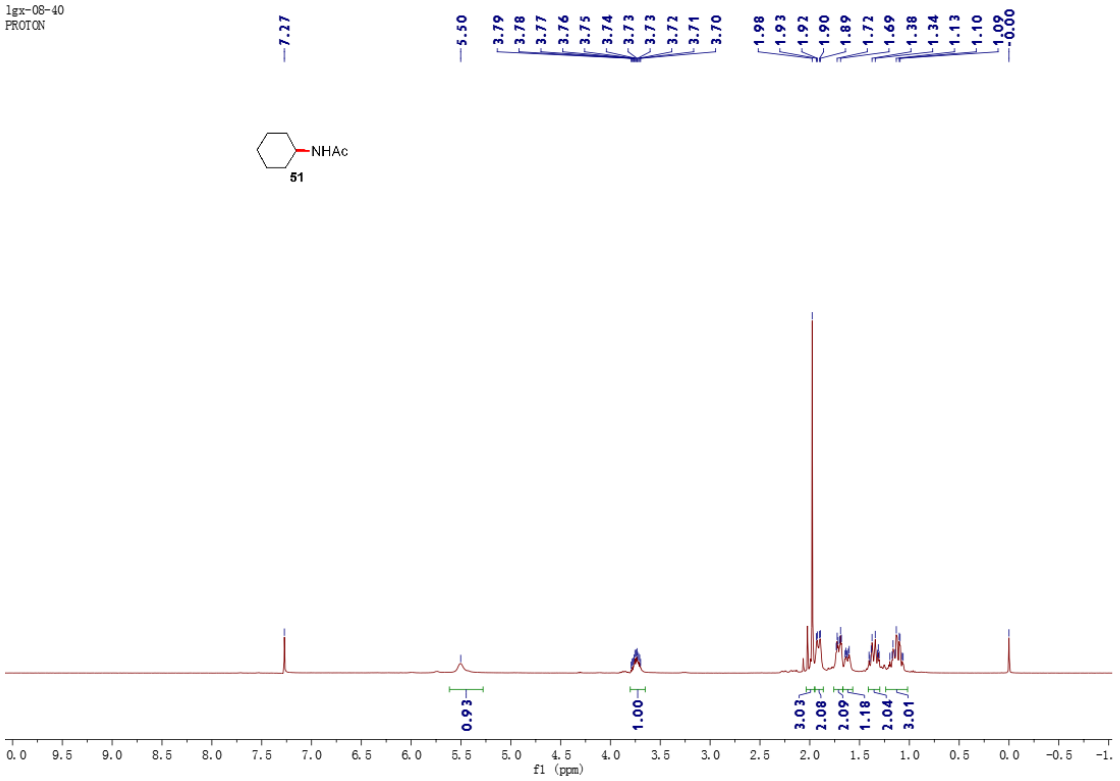
lgx-08-40
PROTON
NHAc
51
f1 (ppm)

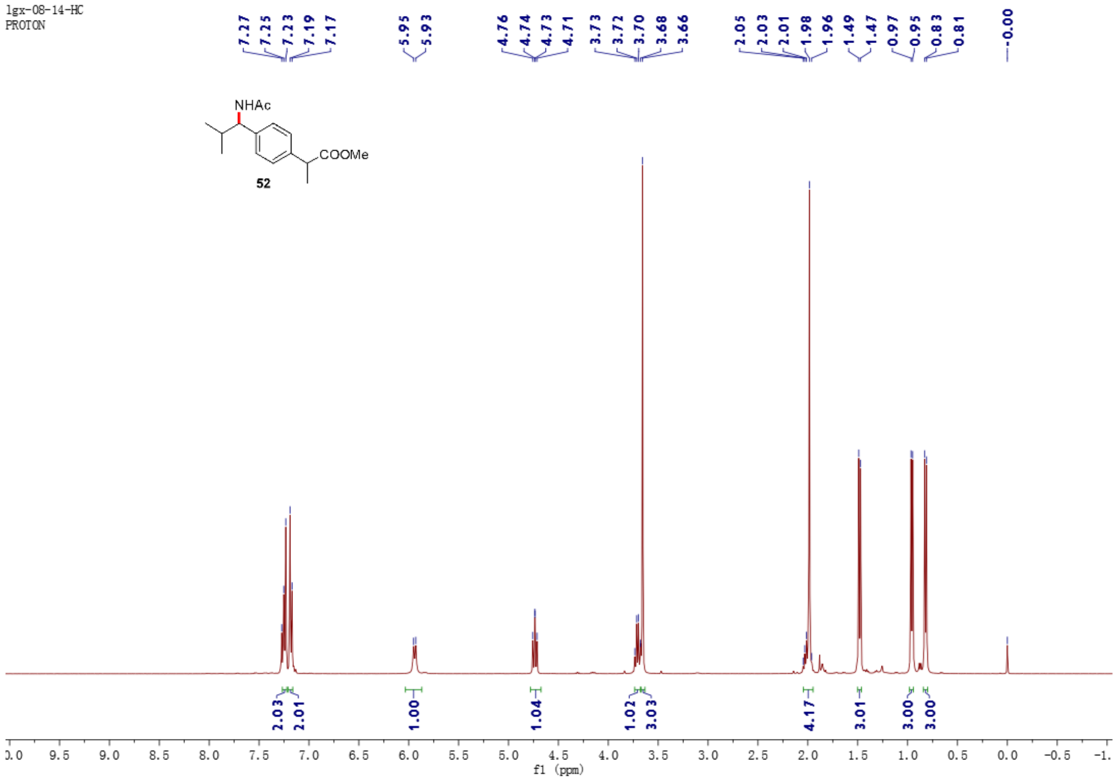
lgx-08-14-HC
PROTON
NHAc
COOMe
52
f1 (ppm)

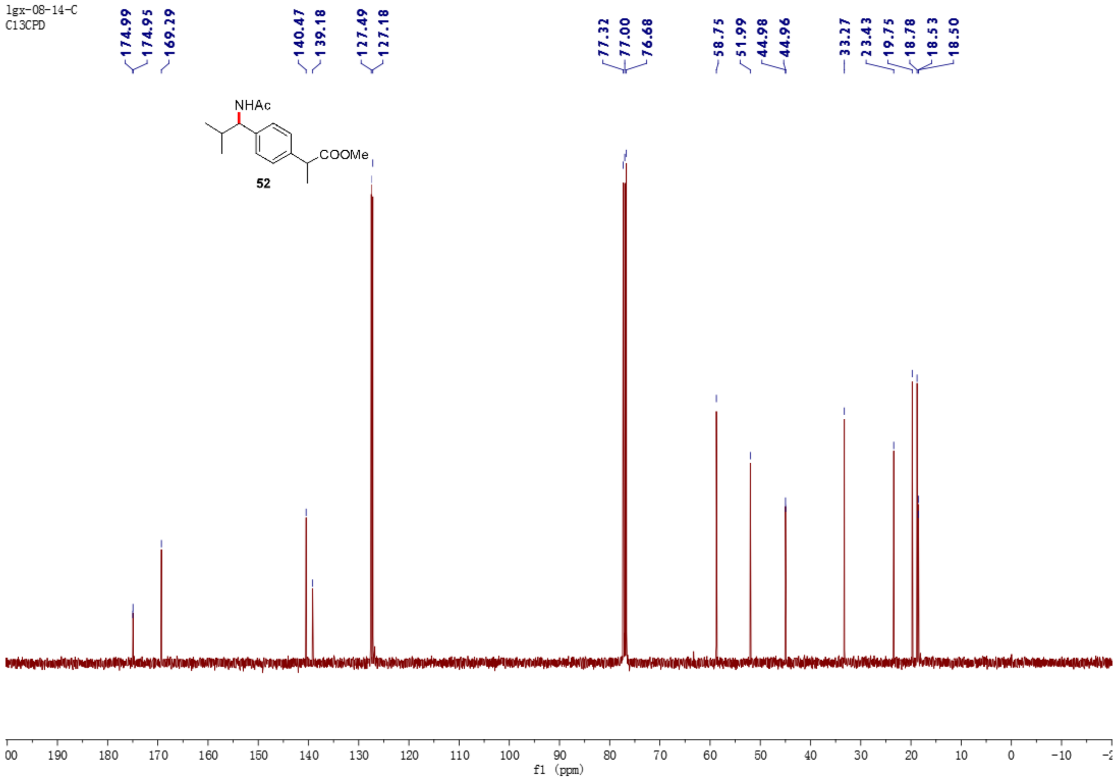
lgx-08-14-C
C13CPD
174.99
174.95
169.29
140.47
139.18
127.49
127.18
77.32
77.00
76.68
58.75
51.99
44.98
44.96
33.27
23.43
19.75
18.78
18.53
18.50
NHAc
COOMe
52
f1 (ppm)

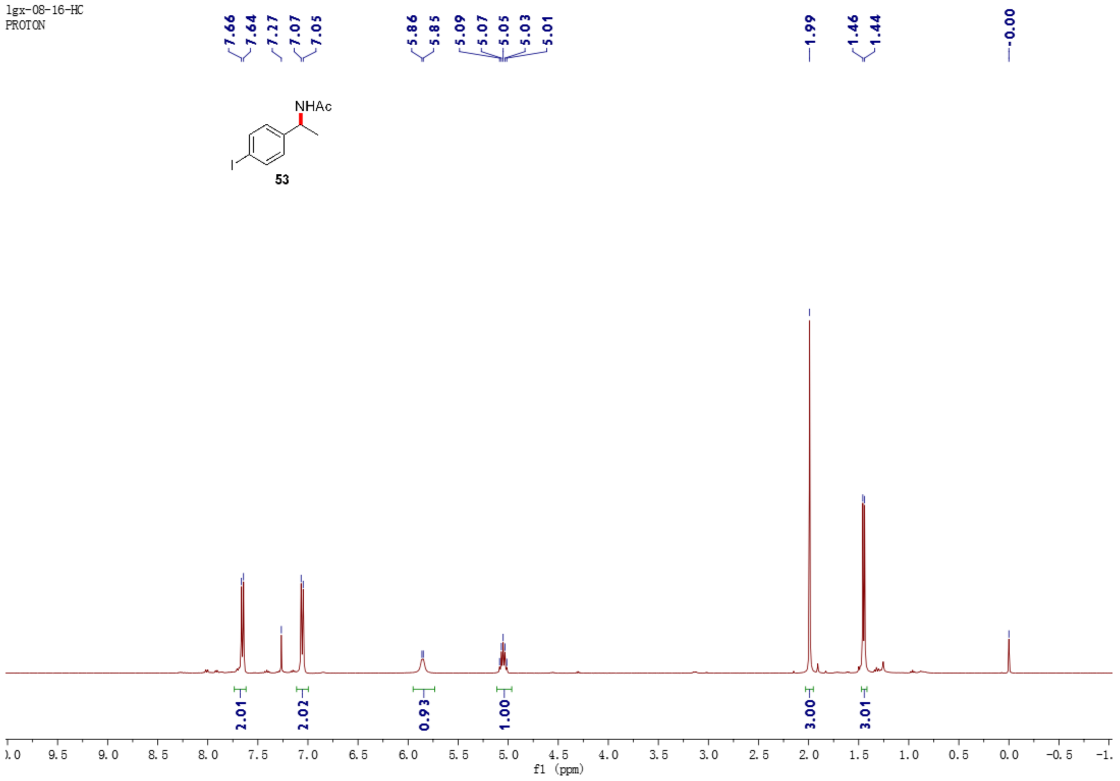
lgx-08-16-HC
PROTON
7.66
7.64
7.27
7.07
7.05
5.86
5.85
5.09
5.07
5.05
5.03
5.01
1.99
1.46
1.44
0.00
NHAc
I
53
2.01
2.02
0.93
1.00
3.00
3.01
f1 (ppm)

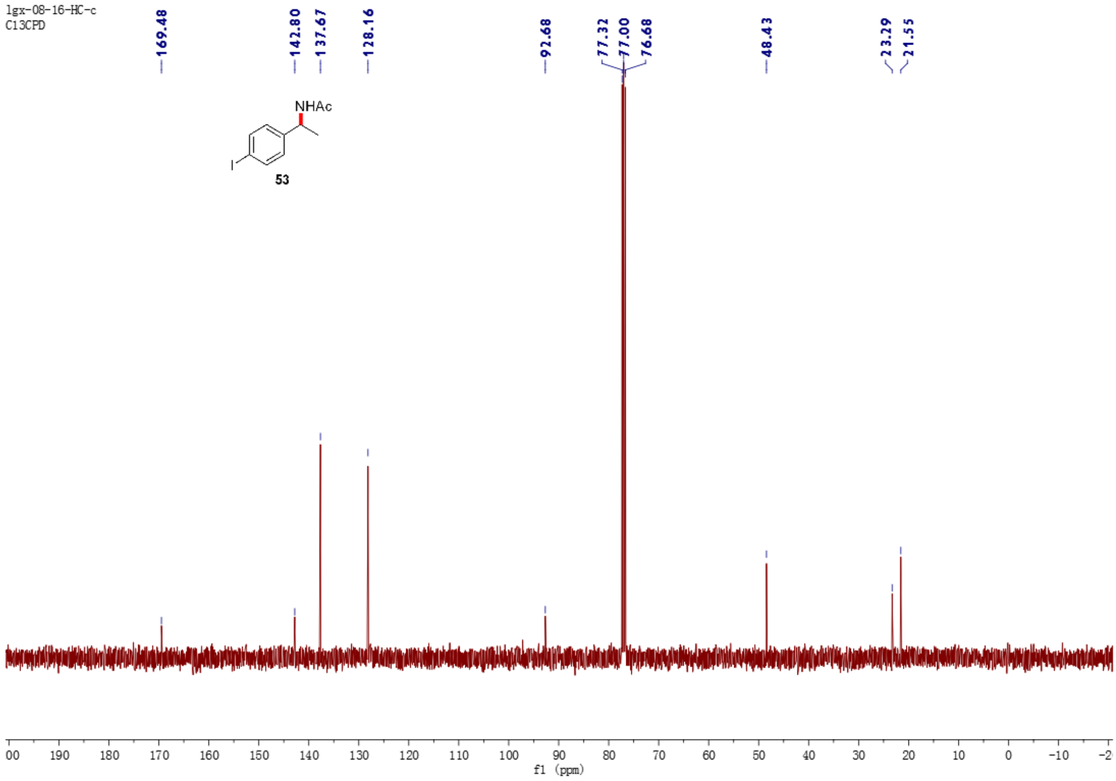
lgx-08-16-HC-c
C13CPD
169.48
142.80
137.67
128.16
92.68
77.32
77.00
76.68
48.43
23.29
21.55
NHAc
I
53
f1 (ppm)

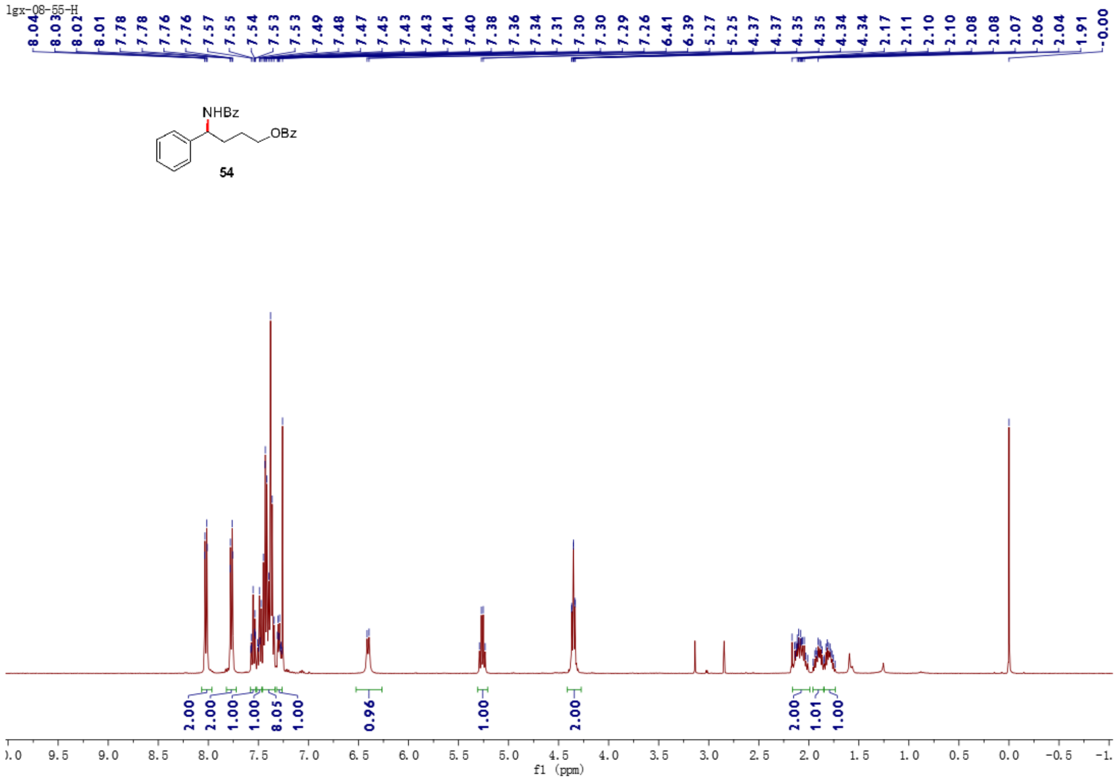
lgx-08-55-H
8.04 8.03 8.02 8.01 7.78 7.78 7.76 7.76 7.57 7.55 7.54 7.53 7.53 7.49 7.48 7.47 7.45 7.43 7.43 7.41 7.40 7.38 7.36 7.34 7.31 7.30 7.29 7.26 6.41 6.39 5.27 5.25 4.37 4.37 4.35 4.35 4.34 4.34 2.17 2.11 2.10 2.10 2.08 2.08 2.07 2.06 2.04 1.91 0.00
NHBz
OBz
54
2.00 2.00 1.00 1.00 8.05 1.00 0.96 1.00 2.00 2.00 1.01 1.00
f1 (ppm)

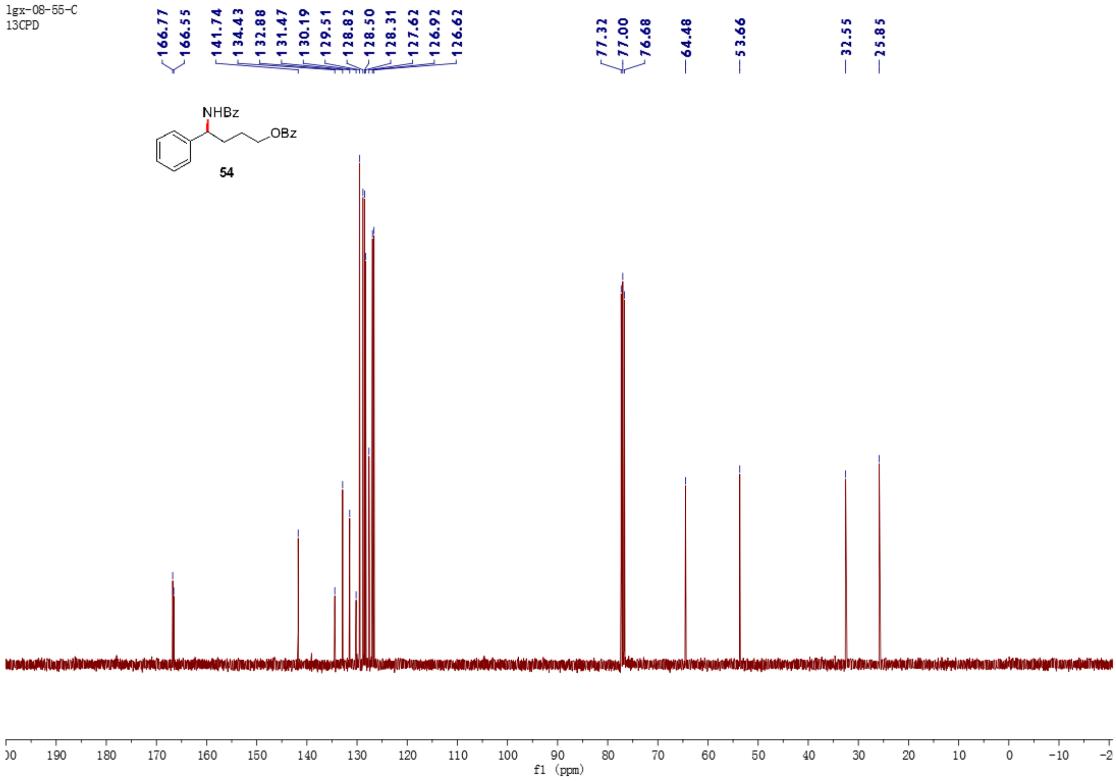

lgx-08-55-C
13CPD
166.77
166.55
141.74
134.43
132.88
131.47
130.19
129.51
128.82
128.50
128.31
127.62
126.92
126.62
77.32
77.00
76.68
64.48
53.66
32.55
25.85
NHBz
OBz
54
f1 (ppm)
